# Screening cell–cell communication in spatial transcriptomics via collective optimal transport

**DOI:** 10.1038/s41592-022-01728-4

**Published:** 2023-01-23

**Authors:** Zixuan Cang, Yanxiang Zhao, Axel A. Almet, Adam Stabell, Raul Ramos, Maksim V. Plikus, Scott X. Atwood, Qing Nie

**Affiliations:** 1 Department of Mathematics and Center for Research in Scientific Computation, North Carolina State University, Raleigh, NC USA; 2 Department of Mathematics, The George Washington University, Washington, DC USA; 3 Department of Mathematics, University of California, Irvine, Irvine, CA USA; 4 The NSF-Simons Center for Multiscale Cell Fate Research, University of California, Irvine, Irvine, CA USA; 5 Department of Developmental and Cell Biology, University of California, Irvine, Irvine, CA USA

**Keywords:** Cellular signalling networks, Computational models, Software, Transcriptomics

## Abstract

Spatial transcriptomic technologies and spatially annotated single-cell RNA sequencing datasets provide unprecedented opportunities to dissect cell–cell communication (CCC). However, incorporation of the spatial information and complex biochemical processes required in the reconstruction of CCC remains a major challenge. Here, we present COMMOT (COMMunication analysis by Optimal Transport) to infer CCC in spatial transcriptomics, which accounts for the competition between different ligand and receptor species as well as spatial distances between cells. A collective optimal transport method is developed to handle complex molecular interactions and spatial constraints. Furthermore, we introduce downstream analysis tools to infer spatial signaling directionality and genes regulated by signaling using machine learning models. We apply COMMOT to simulation data and eight spatial datasets acquired with five different technologies to show its effectiveness and robustness in identifying spatial CCC in data with varying spatial resolutions and gene coverages. Finally, COMMOT identifies new CCCs during skin morphogenesis in a case study of human epidermal development.

## Abstract (web summary)

This work presents a computational framework, COMMOT, to spatially infer cell–cell communication from transcriptomics data based on a variant of optimal transport (OT).

## Main

The complex structures and functions of multicellularity are achieved through the coordinated activities of various cells. Cells make decisions and accomplish their goals by interacting with an environment consisting of external stimuli and other cells. A major form of cell–cell interaction is cell–cell communication (CCC), mainly mediated by biochemical signaling through ligand–receptor binding that induces downstream responses that shape development, structure and function.

Traditionally, CCC studies were restricted to a few cell types and a small number of selected genes at the resolution of cell groups. Recently, the emergence of single-cell transcriptomics (that is, single-cell RNA sequencing, scRNA-seq) has enabled the examination of tissues at single-cell resolution at unprecedented genomic coverage^1^. Computational tools have been developed to estimate CCC activities from scRNA-seq data^2,3^ using signaling databases^4–6^. Most of these methods rely on the expression levels of ligand and receptor pairs and explicitly defined functions. For example, the products of ligand and receptor levels^5,7^ or non-linear Hill function-based models^6^ are used. In addition, these methods emphasize different aspects of CCC. For example, CellPhoneDB^5^, ICELLNET^7^ and CellChat^6^ account for the multi-subunit composition of protein complexes; SoptSC^8^, NicheNet^9^ and CytoTalk^10^ utilize downstream intracellular gene–gene interactions; and scTensor^11^ examines higher-order CCC represented as hypergraphs. These inference methods designed for scRNA-seq data have provided biological insights based on non-spatial transcriptomic data^2,12,13^. However, these non-spatial studies often contain significant false positives given that CCC takes place only within limited spatial distances that are not measured in scRNA-seq datasets. Improvements can be made by filtering the inferred CCC using spatial annotations^14^.

Spatial transcriptomics^15–20^ provides information on the distance between cells or spots containing multiple or fractions of cells. At various cellular resolutions these technologies measure the spatial expression of hundreds to tens of thousands of genes in 2-dimensional (2D) or 3-dimensional tissue (3D) samples^21^. Methods and software^22–24^ developed for non-spatial data analysis have been applied to spatial data, with a small number of methods designed specifically for spatial data. Giotto builds a spatial proximity graph to identify interactions through membrane-bound ligand–receptor pairs^23^; CellPhoneDB v3 restricts interactions to cell clusters in the same microenvironment defined based on spatial information^25^; stLearn relates the co-expression of ligand and receptor genes to the spatial diversity of cell types^24^; SVCA^26^ and MISTy^27^ use probabilistic and machine learning models, respectively, to identify the spatially constrained intercellular gene–gene interactions; and NCEM fits a function to relate cell type and spatial context to gene expression^28^. However, current methods examine CCC locally and on cell pairs independently, and focus on information between cells or in the neighborhoods of individual cells. As a result, collective or global information in CCC, such as competition between cells, is neglected.

Optimal transport has recently been used for transcriptomic data analysis, including batch effect correction^29^, developmental trajectory reconstruction^30^ and spatial annotation of scRNA-seq data^31,32^. Naturally, one can form an optimal transport problem by viewing ligand and receptor expression as two distributions to be coupled with a cost based on spatial distance^31,33,34^. However, when using classical optimal transport, different molecule species with significantly different expression levels are normalized to ensure the same total mass, which renders the units of distributions unable to be compared. Furthermore, multiple ligand species can bind to multiple receptor species, resulting in competition. Of the 1,735 (secreted) ligand–receptor pairs in the Fantom5 database^35^, 72% of ligands (372 of 516) and 60% of receptors (309 of 512) bind to multiple species. Such competition between multiple molecule species is ubiquitous and a critical biophysical process but it is ignored in existing methods. Although recent optimal transport variants such as unbalanced optimal transport and partial optimal transport can deal with unnormalized distributions and avoid certain coupling due to signaling spatial range and simultaneous consideration of multiple species^33,36–38^, they introduce other issues. Specifically, unbalanced optimal transport^38^ in its common form uses Kullback–Leibler divergence as a soft constraint on marginal distribution preservation. This approach may result in the total amount of coupled signaling molecule species significantly exceeding the total amount of either ligand or receptor initially available. By contrast, partial optimal transport^36^ requires an additional parameter, the total coupled mass, which is usually difficult to estimate in the context of CCC inference.

To adapt optimal transport theory for the application of CCC inference, we present a method called collective optimal transport, which is capable of preserving the comparability between distributions, ensuring that the total signal does not exceed the individual species amounts (ligand or receptor), enforcing spatial range limits of signaling, and handling multiple competing species. The collective optimal transport method achieves this by optimizing the total transported mass and the ligand–receptor coupling simultaneously, unlike existing optimal transport methods. By introducing an entropy regularization to enforce the inequalities for marginal distributions, the collective optimal transport can be reformulated as a special case of the general unbalanced optimal transport framework^38^. An efficient algorithm is developed specifically for solving the collective optimal transport problem.

Based on collective optimal transport, we develop COMMunication analysis by Optimal Transport (COMMOT), a package that infers CCC by simultaneously considering numerous ligand–receptor pairs for either spatial transcriptomics data or spatially annotated scRNA-seq data equipped with spatial distances between cells estimated from paired spatial imaging data; summarizes and compares directions of spatial signaling; identifies downstream effects of CCC on gene expressions using ensemble of trees models; and provides visualization utilities for the various analyses.

We show that COMMOT accurately reconstructs CCC on simulated data generated by partial differential equation (PDE) models and outperforms three related optimal transport methods. We then apply COMMOT to analyze scRNA-seq data that have been spatially annotated using paired spatial datasets and five types of spatial transcriptomics data that differ with respect to spatial resolution or gene coverage. Finally, we examine a specific system of human epidermal development and elucidate connections between CCC and skin development.

## Results

### Overview of COMMOT

**Fig. 1:**
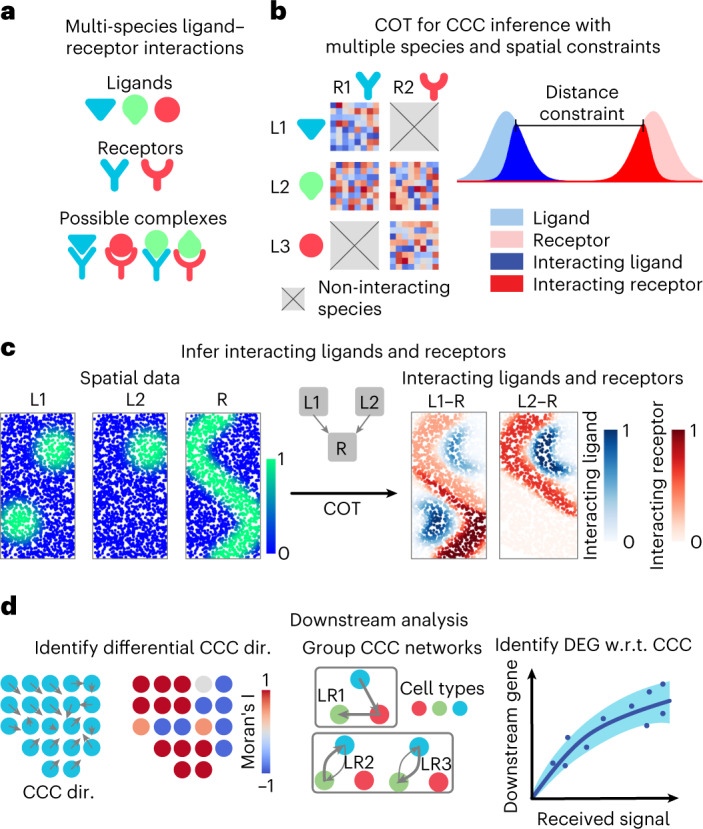
Overview of COMMOT. **a**, COMMOT infers CCC in space while considering the competition between different ligand and receptor species. **b**, Collective optimal transport (COT) infers CCC in space by introducing multi-species distributions and enforcing limited spatial ranges. **c**, An example of inferring CCC for spatial distributions of ligand–receptor complexes from spatial distributions of the ligands and receptor where two ligand species (L1, L2) compete for one receptor species (R). **d**, Three applications of downstream analysis based on the inferred CCC network between cells or spots. DEG, differentially expressed gene; dir., direction; w.r.t., with respect to.

Ligands and receptors often interact with multiple species and within limited spatial ranges (Fig. 1a). Considering this, we present collective optimal transport (Fig. 1b) with three important features: first, the use of non-probability mass distributions to control the marginals of the transport plan to maintain comparability between species; second, enforcement of spatial distance constraints on CCC to avoid connecting cells that are spatially far apart; and last, the transport of multi-species distributions (ligands) to multi-species distributions (receptors) to account for multi-species interactions (Fig. 1c).

Given a spatial transcriptomics dataset of *n*_*s*_ cells or spots and *n*_*l*_ ligand species and *n*_*r*_ receptor species, the collective optimal transport determines an optimal multi-species coupling ${{{\boldsymbol{P}}}}^ \ast \in {\Bbb R}_ + ^{n_l\, \times \,n_r\, \times \,n_s\, \times \,n_s}$ where ${{{\boldsymbol{P}}}}_{i,j,k,l}^ \ast$ scores the signaling strength from sender cell *k* to receiver cell *l* through ligand *i* and receptor *j*. This is achieved by solving a minimization problem, $\mathop{\min}\limits_{{{{\boldsymbol{P}}}} \in \Gamma }\mathop {\sum}\nolimits_{\left( {i,j} \right) \in I} {\alpha _{\left( {i,j} \right)}\left\langle {{{{\boldsymbol{P}}}}_{i,j, \cdot , \cdot },\,{{{\boldsymbol{C}}}}_{\left( {i,j} \right)}} \right\rangle _F}$ where

$$\begin{array}{l}\Gamma = \left\{ {{{{\boldsymbol{P}}}} \in {\Bbb R}_ + ^{n_l\, \times \,n_r\, \times \,n_s\, \times \,n_s}:{{{\boldsymbol{P}}}}_{i,j, \cdot , \cdot } = 0\,{{{\mathrm{for}}}}\,\left( {i,j} \right) \notin I,}\right.\\ \qquad \left.{ \mathop {\sum}\nolimits_{j,l} {{{{\boldsymbol{P}}}}_{i,j,k,l} \le {{{\boldsymbol{X}}}}_{i,k}} ,\mathop {\sum}\nolimits_{i,k}}\right.\left.{\boldsymbol{P}}_{i,j,k,l} \le {\boldsymbol{X}}_{j,l}\right\},\end{array}$$

*I* is the index set for ligand and receptor species that can bind together, and ${{{\boldsymbol{X}}}}_{i,k}$ is the expression level of gene *i* on spot *k*. The species-specific cost matrix ${{{\boldsymbol{C}}}}_{\left( {i,j} \right)}$ is a modified distance matrix for between-spot distance that replaces distances exceeding the spatial range of ligand *i* by infinity. The competitions between molecule species and cells are considered by assuming that a given receptor species or cell has limited capacity for interactions, such that a stronger inferred interaction with one ligand species or cell reduces the potential of interaction with other ligand species or cells (see the Methods and Supplementary Note for detailed formulations and algorithm derivations).

**Extended Data Fig. 1:**
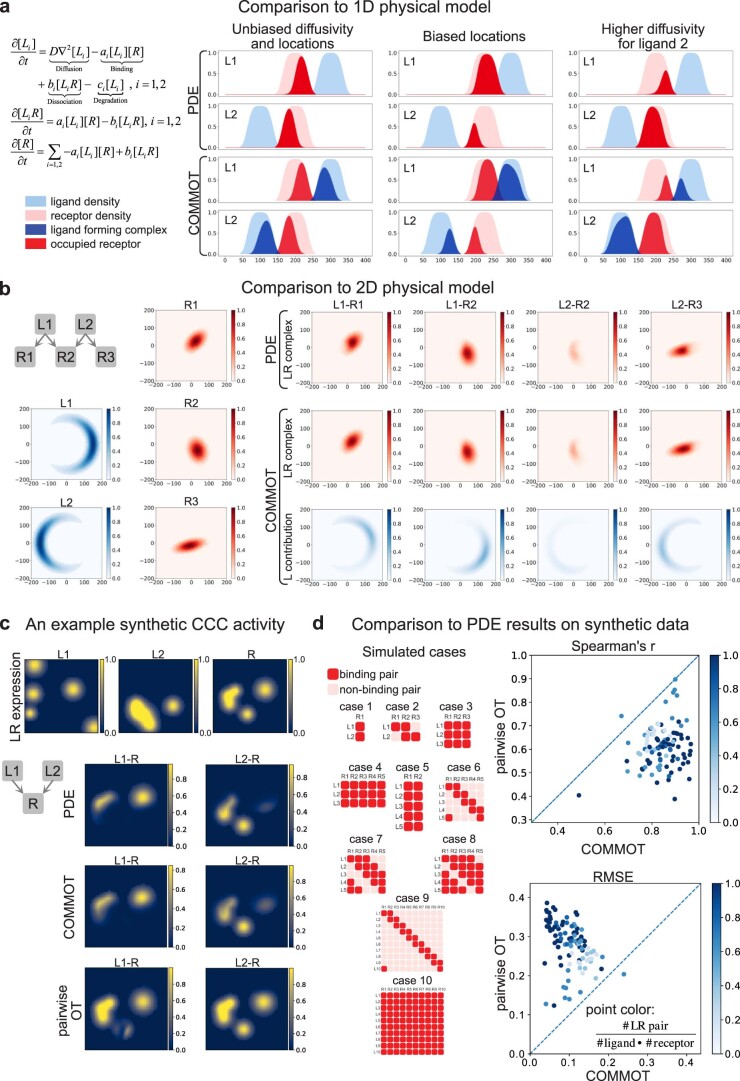
Validation using simulated data by partial differential equations (PDE) model. The example PDE model where two ligand species can bind to the same receptor. The inference by COMMOT is compared to the simulation results in several 1-dimensional cases. **b** Comparison to simulated results in a 2-dimensional case with three ligand species and two receptor species. **c** An example of randomly generated 2-dimensional benchmark with two ligand species that binds to the same receptor. The simulated result, inference by COMMOT, and inference by pairwise method are shown. **d** Ten different cases of ligand–receptor binding and the performance of COMMOT and pairwise OT (with the same spatial limit as COMMOT but each LR pair examined separately) obtained by comparing to simulated results.

Direct validation of CCC inference methods for spatial data is difficult due to a lack of spatial co-localization measurements of ligand and receptor proteins. Here, we built PDE models to simulate CCC in space (Extended Data Fig. 1). Simulating various numbers of ligand and receptor species and diverse competition patterns, COMMOT accurately reconstructs the CCC connections from the resulting synthetic data (Extended Data Fig. 1d and Supplementary Figs. 1–4). COMMOT outperformed, and is significantly different from, two related optimal transport variants: unbalanced optimal transport and partial optimal transport (Supplementary Figs. 5–9). COMMOT’s characteristics of enforcing spatial limits and not requiring probability distributions are further illustrated with other real spatial transcriptomics datasets (Supplementary Figs. 10 and11).

For each ligand–receptor pair and each pair of cells or spots, the CCC inference quantifies the ligand contributed by one spot to the ligand–receptor complex in another spot. We then perform several downstream analyses: first, interpolation of the spatial signaling direction and identification of the differences between CCC regions; second, summarization and grouping of CCC at the spatial cluster level; and last, identification of the downstream genes affected by the CCC (Fig. 1d). The spatial signaling direction is obtained by interpolating the cell-by-cell CCC matrix to a vector field to identify the direction from which the signal is received or sent. For downstream analysis we first identify genes that are differentially expressed with the received signal, then quantify the CCC effect on these genes while considering the effect of other genes by incorporating a machine learning model that predicts a target gene level using both the received signal and other correlated genes. See Methods for the algorithms that perform the downstream tasks.

### The roles of CCC in human epidermal development

**Fig. 2:**
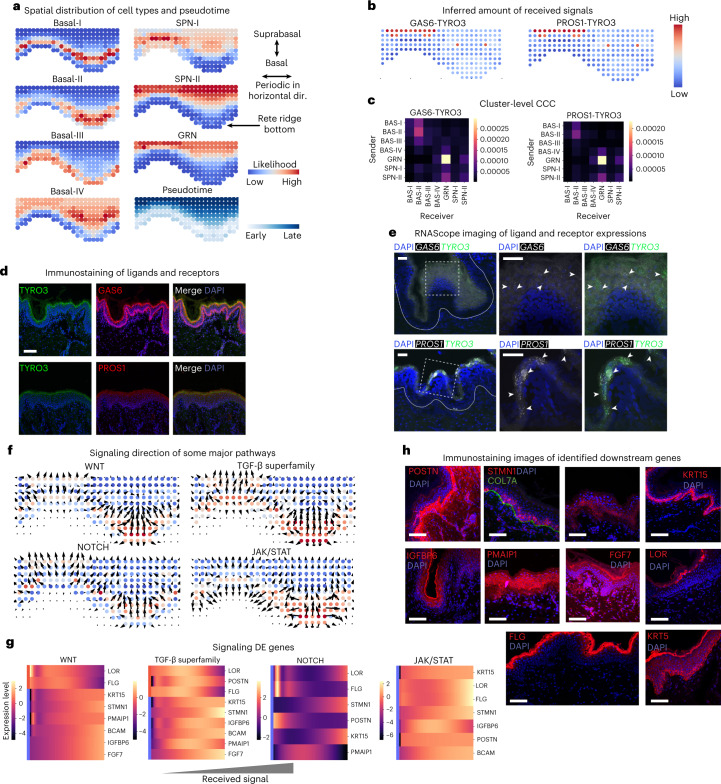
Role of CCC in human skin development. **a**, Predicted spatial origin of the skin subtypes of cells in intact tissue and the pseudotime projected to space. GRN, granular cell cluster; SPN, spinous cell cluster. **b**,**c**, The inferred amount of received signals of two example ligand–receptor pairs, GAS6-TYRO3 and PROS1-TYRO3 at the cell level (**b**) and cluster level (**c**). **d**, Immunostaining of proteins for GAS6, TYRO3 and PROS1. **e**, Fluorescent in situ hybridization against RNA molecules for predicted ligand–receptor interactions in human epidermis (solid white outline; regions of interest are marked by a white dashed square). The top row shows expression patterns of *GAS6* (white) and *TYRO3* (green); the bottom row shows expression patterns for *PROS1* (white) and *TYRO3* (green). In both cases, the middle and right panels show ligand–receptor signals, some of which colocalize to the stratum granulosum (white arrowheads). In merged images, the brightness of the *GAS6* channel was increased to improve clarity against the prominent *TYRO3* (green) signal. Experiments were repeated four times independently with consistent results. **f**, The signaling directions of four major signaling pathways. **g**, Heatmaps of selected signaling differentially expressed genes of the four signaling pathways, respectively. **h**, Immunofluorescence staining images of the identified signaling differentially expressed genes supporting the identified correlation between WNT signaling and the expression of these genes. Scale bars: **d**,**e**,**h**, 100 μm. The immunostaining experiments in **d** and **h** were repeated three times independently with consistent results.

We applied COMMOT to examine the development of epidermis in human skin. Our recent work profiled neonatal human epidermis using scRNA-seq and identified four stem cell clusters (basal I, II, III and IV) found in different regions of the innermost basal layer of the epidermis, a differentiating spinous cell cluster in the intermediate layer, and a granular cell cluster in the outermost living layers^39^. A refined in situ spatial transcriptomic map was constructed using SpaOTsc^31^ by integrating scRNA-seq data with spatial data digitized from immunofluorescence staining images. The integrated dataset correctly identified previously known locations of the epidermal cell types and agreed with a known developmental path by epidermal cells from basal to suprabasal layers (Fig. 2a). This result was further validated by leave-one-out validation (Supplementary Fig. 12).

The spatial signaling between epidermal cells was inferred in the integrated dataset by considering ligand–receptor pairs annotated in the database CellChatDB. For example, our computational analysis predicted that molecular interactions between the ligands GAS6 and PROS1 with their receptor TYRO3 (GAS6-TYRO3 and PROS1-TYRO3) are significant in granular cells and moderately present in basal cells (Fig. 2b). This prediction was confirmed by both immunostaining for proteins (Fig. 2d) and using RNAscope to stain for RNA (Fig. 2e).

At the signaling pathway level we examined four specific pathways with known important roles in epidermal homeostasis, namely the WNT, TGF-β (transforming growth factor-β), NOTCH and JAK/STAT (Janus kinase/signal transducers and activators of transcription) pathways^39^ (Fig. 2f and Supplementary Figs. 13–16). For all four pathways we observed mainly upward-directed signaling, with some downward signaling to the basal layers at the bottom of the ridges (Fig. 2f). WNT signaling is known to promote basal stem cell proliferation^40^, whereas TGF-β suppresses it^41,42^. Thus, this observed directional signaling from the suprabasal layers may be regulating the communications to basal cells on proliferation.

Based on the inferred signaling activities, we further identified differentially expressed genes corresponding to each signaling pathway and modeled their expression level changes with increasing received signal without further considering spatial information (Fig. 2g). For the WNT pathway, increasing signal results in higher expression of the known basal cell markers *KRT15* and *KRT5*, as well as lower expression of the known terminally differentiated granular cell markers *LOR* and *FLG*, reinforcing the WNT pathway’s known role in stem cell proliferation^40^. The analysis also predicted that higher WNT signaling would increase the expression of *BCAM*, *POSTN* and *STMN1*, the expression localization of which we confirmed by immunostaining on human epidermis (Fig. 2h). Interestingly, computational results predicted that *IGFBP6*, *PMAIP1* and *FGF7* would correlate positively with WNT signaling, but we observed their expression mainly in the spinous and granular layers, possibly due to predicted WNT signaling in both directions in basal-IV (Fig. 2h). TGF-β signaling had a similar profile to that of the WNT pathway, with NOTCH and JAK/STAT signaling having a more complex response (Fig. 2g). These results suggest how testable hypotheses can be derived from inferred signaling activities.

### Signaling analysis in spatial transcriptomics data with high spatial resolution

**Fig. 3:**
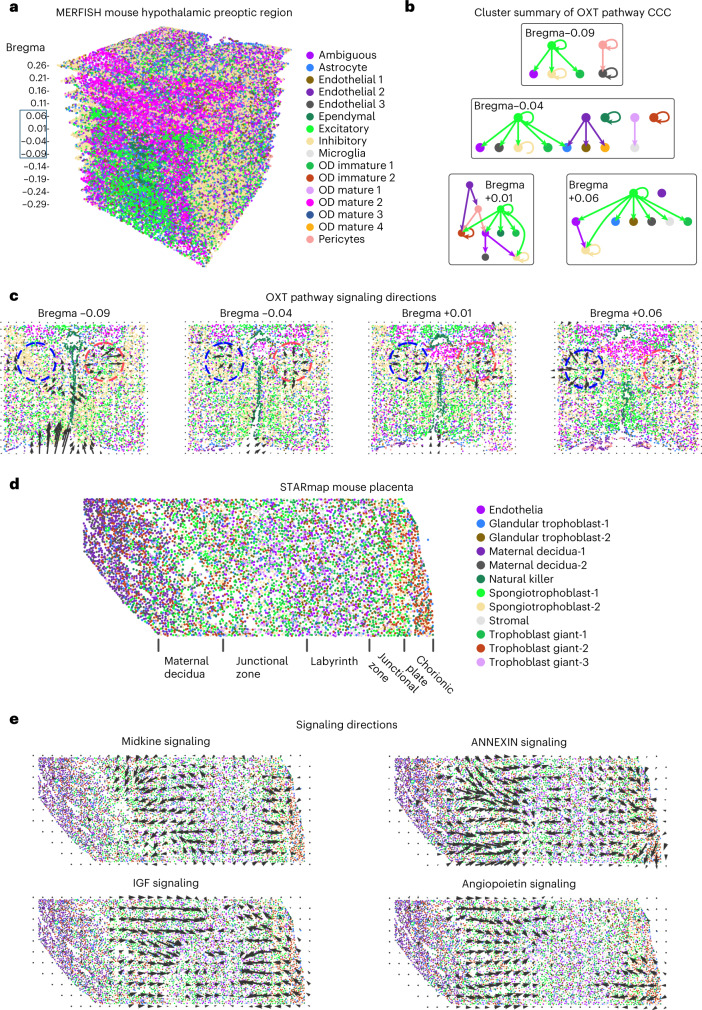
Inference of signaling direction in single-cell resolution spatial transcriptomics data. **a**, MERFISH data of the mouse hypothalamic preoptic region with multiple slices across the anterior–posterior axis^44^. **b**, Cluster-level summary of CCC through the OXT signaling pathway. **c**, Signaling directions of the OXT pathway. **d**, STARmap data of the mouse placenta^46^. **e**, Signaling directions of the midkine, IGF, annexin and angiopoietin pathways.

**Extended Data Fig. 2:**
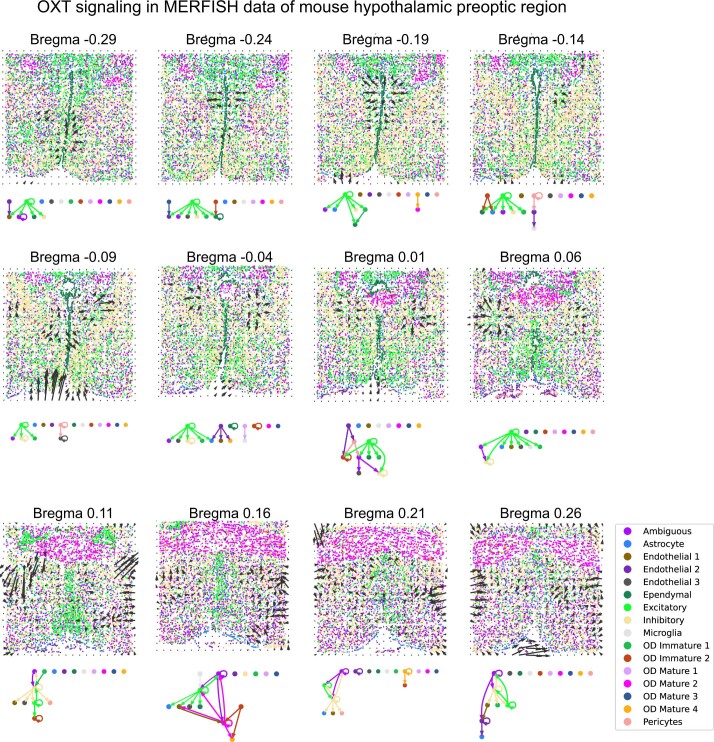
OXT CCC in MERFISH mouse hypothalamic preoptic region. The inferred signaling directions and cluster-level CCC of OXT signaling in each of the slice of the MERFISH data.

We first studied CCC in spatial transcriptomics data with high spatial resolution using the CellChatDB^6^. We analyzed MERFISH (multiplexed error-robust fluorescence in situ hybridization) data of the mouse hypothalamic preoptic region with 161 genes and 73,655 cells across 12 slices along the anterior–posterior axis^43^ (Fig. 3a–c). Of the signaling pathways available in the data, oxytocin (OXT) signaling, an important pathway that modulates social behaviors, was found to be most active. Self-modulation of excitatory neurons and modulation of inhibitory neurons by excitatory neurons through OXT signaling were identified across all of the slices (Fig. 3b, Extended Data Fig. 2 and Supplementary Fig. 17), a result consistent with the known major functions of OXT signaling^44^. Further analysis identified the local regions of high OXT signaling activity and the spatial direction of OXT signaling (Fig. 3c), which agreed with the results of protein staining of OXT and its receptor^45^. A gradual change of predicted signaling direction and high-activity regions was observed through adjacent slices (Fig. 3c and Extended Data Fig. 2).

We then analyzed STARmap (spatially-resolved transcript amplicon readout mapping) data of mouse placenta with 903 genes and 7,203 cells^46^ (Fig. 3d). Midkine and insulin-like growth factor (IGF) signaling were found to be active in the same regions but with opposing directions (Fig. 3e), suggesting a potential feedback loop^47^. In addition, it was found that IGF signaling is active in the labyrinth region and in endothelial cells, both of which were consistent with our predictions^48^. Midkine signaling was inferred to be active in trophoblast cells, consistent with previous findings on the role of SDC1 and SDC4 in trophoblast cells^49,50^ (Supplementary Fig. 18). We also found that the annexin and the angiopoietin signaling pathways were active in similar regions with similar directions, suggesting that they may function cooperatively (Fig. 3e).

**Fig. 4:**
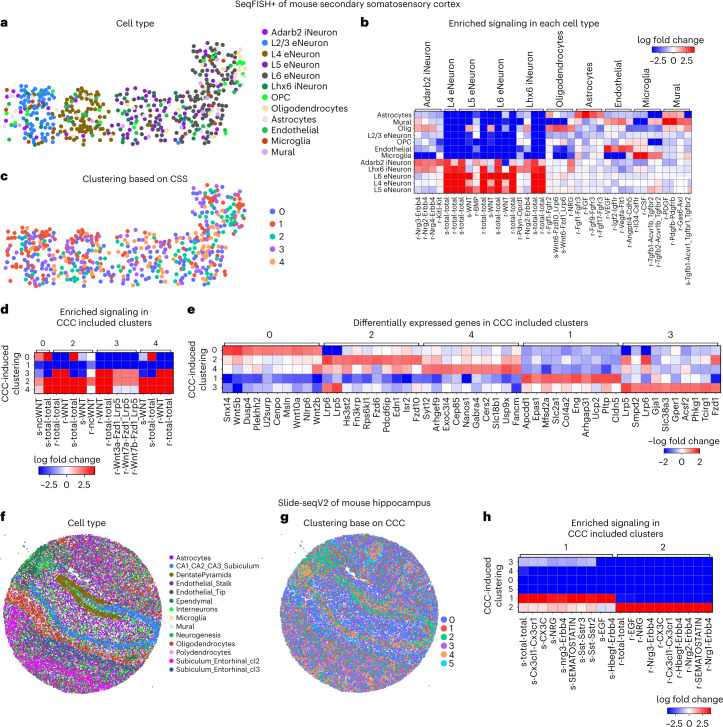
Downstream analysis of inferred CCC in single-cell resolution spatial transcriptomics data. **a**–**e**, CCC analysis of seqFISH+ data of mouse secondary somatosensory cortex. **a**, Clustering of cell type based on gene expression. OPC, oligodendrocyte precursor cells. **b**, Enriched signaling in each cell type. **c**, Clustering based on inferred CCC. **d**, Enriched signaling in CCC-induced clusters. **e**, Differentially expressed genes in the CCC-induced clusters. **f**–**h**, CCC analysis of Slide-seq (v2) data of mouse hippocampus. **f**, Clustering of cell type based on gene expression. **g**, Clustering based on inferred CCC. **h**, Enriched signaling in CCC-induced clusters.

To demonstrate downstream analyses of CCC, we first studied seqFISH+ (sequential fluorescence in situ hybridization) data of mouse secondary somatosensory cortex with 10,000 genes measured in 523 individual cells^18^ (Fig. 4a–e). Using the inferred CCC, each cell was assigned a CCC profile quantifying the amount of signal sent or received through each ligand–receptor pair to assemble a (*n*_*s*_ × 2*n*_*lr*_) CCC profile matrix for the *n*_*s*_ cells and *n*_*lr*_ ligand–receptor pairs. Differential expression analysis of the cell types and CCC profile found neuron cells to be most active through various ligand–receptor pairs, and distinct CCC activities for relatively rare cell types (Fig. 4b). Predicted significant WNT signaling in neurons (Supplementary Fig. 19) correlated well with known critical roles of WNT signaling in neuronal migration and activity in the somatosensory cortex^51^.

After clustering with respect to CCC activities, cells in the same group are expected to have similar signaling activities (Fig. 4c). Clusters 2 and 4 showed hyperactive signaling while clusters 0 and 3 were significant signal senders and receivers, respectively (Fig. 4d). We next identified differentially expressed genes that matched the signaling patterns of each CCC-induced cluster (Fig. 4e). This analysis identified both known signaling components in the relevant pathways and regulators of each pathway. For example, the positive differentially expressed genes associated with cluster 0 (WNT signal senders) included the known WNT ligands *Wnt5b*, *Wnt10a* and *Wnt2b*, while the differentially expressed genes in cluster 3 (WNT signal receivers) included known target genes of the WNT signaling pathway such as *Gja1* and *Acsf2* and the known corresponding intracellular signaling transductors *Lrp5* and *Lrp6* (Fig. 4e).

**Extended Data Fig. 3:**
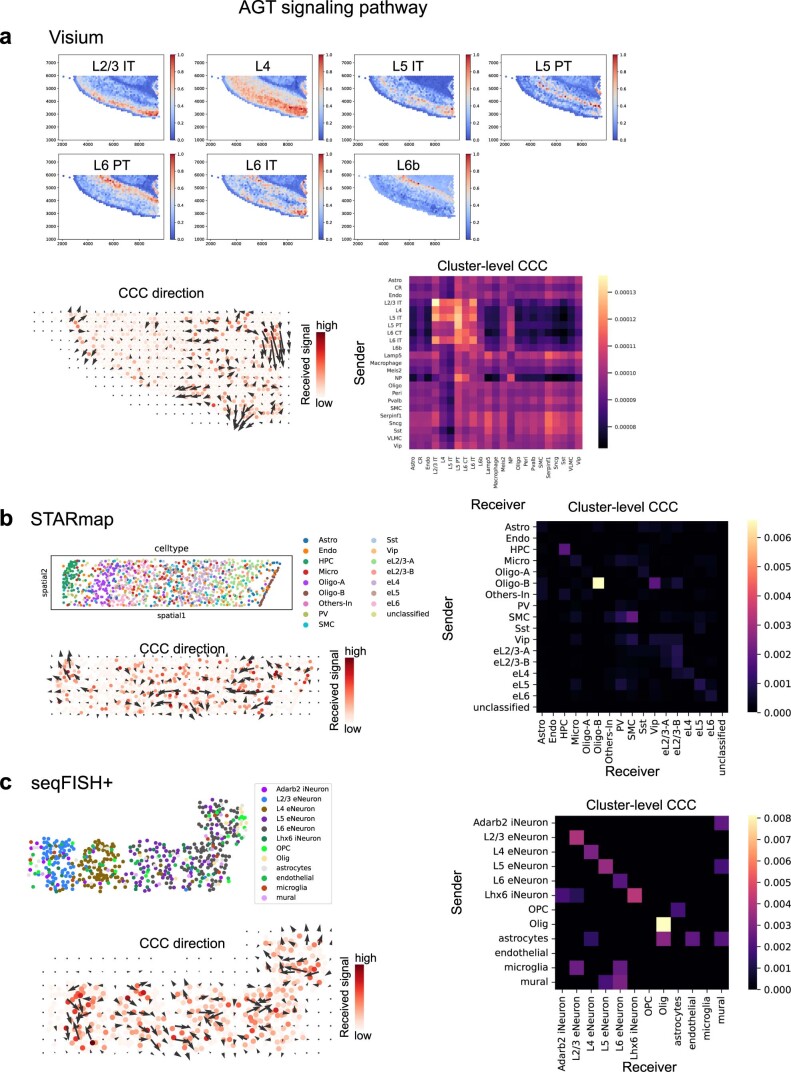
AGT signaling pathway in mouse cortex. 1) Cell type plots, 2) spatial directions of CCC, and 3) heatmaps of cluster-level CCC of the AGT signaling pathway in **a** Visium, **b** STARmap, and **c** seqFISH+ mouse cortex data. Across these three datasets, AGT signaling was identified in neurons. Spatially, neurons in the L2-3 region were identified as strong receivers of AGT ligands across the three datasets. Interestingly, a striped signaling pattern was observed, wherein strong signals within individual layers form stripes, while weak signals form inter-stripe regions. Strong AGT signaling activity among oligodendrocytes was also identified in both STARmap and seqFISH+ datasets.

**Extended Data Fig. 4:**
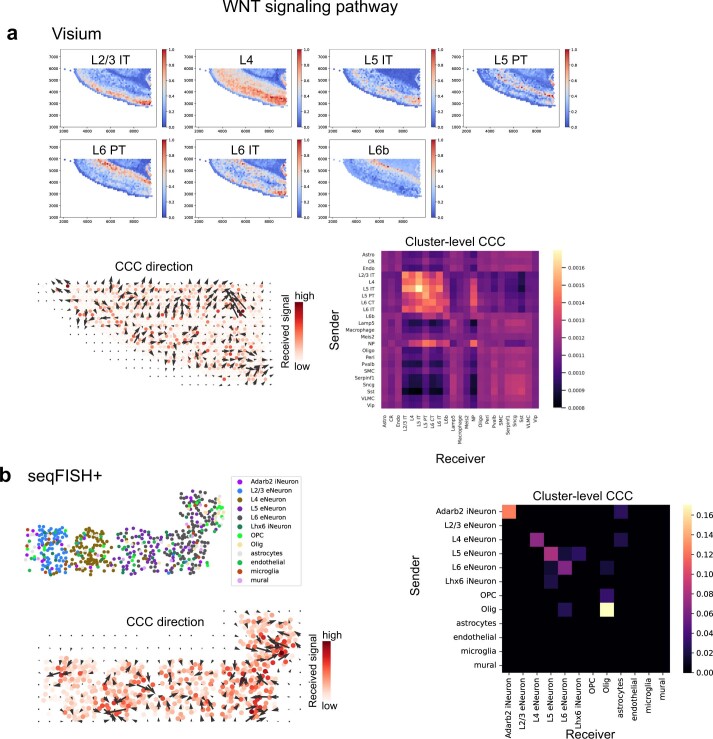
WNT signaling pathway in mouse cortex. 1) Cell type plots, 2) spatial directions of CCC, and 3) heatmaps of cluster-level CCC of the WNT signaling pathway in **a** Visium and **b** seqFISH+ mouse cortex data. In both Visium and seqFISH+ cortex datasets, we inferred WNT signaling to be active across different cortical layers. In both datasets, we identified WNT signaling to be relatively low in layer 5, compared to other layers.

**Extended Data Fig. 5:**
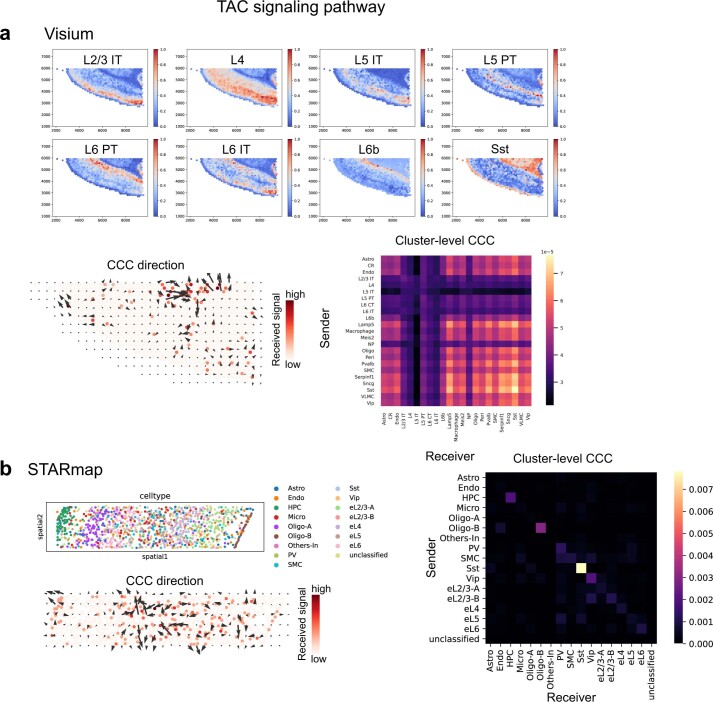
TAC signaling pathway in mouse cortex. 1) Cell type plots, 2) spatial directions of CCC, and 3) heatmaps of cluster-level CCC of the TAC signaling pathway in **a** Visium and **b** STARmap mouse cortex data. TAC (tachykinin neuropeptide family) signaling activity was consistently found in both Visium and STARmap cortex datasets to be active in non-neuronal cells and in inhibitory neurons, especially in somatostatin-expressing neurons (Sst).

We further jointly analyzed CCC in mouse cortex datasets generated with three different technologies: Visium, seqFISH+ and STARmap. We found CCC patterns across the datasets that were consistent with existing knowledge, demonstrating the robustness of COMMOT (Extended Data Figs. 3–5). Details of the findings are given in the Supplementary Note. We also applied COMMOT to a large-scale spatial transcriptomics dataset, that is, Slide-seqV2 data of mouse hippocampus, containing expression of 23,264 genes in 53,173 beads (spatial spots), which are similar in size to individual cells^52^ (Fig. 4f–h). Clustering based on CCC activities separated the spots into six clusters, of which clusters 1 and 2, consisting mostly of DentatePyramid, CA1_CA2_CA3_Subiculum, and interneuron cells, are generally active in CCC (Fig. 4f–h).

### Signaling analysis in multi-cell resolution spatial transcriptomics data

**Fig. 5:**
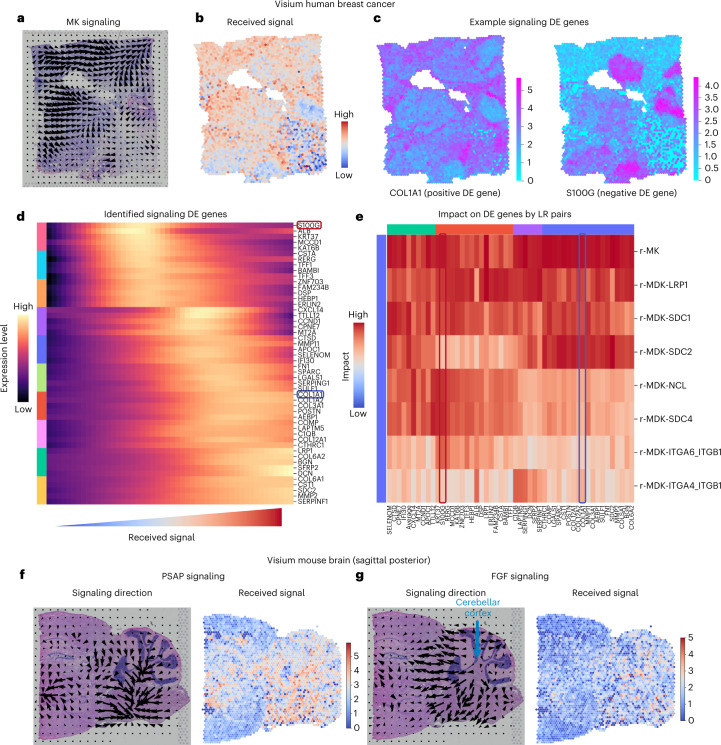
CCC inference using Visium spatial transcriptomics data. **a**–**e**, Midkine (MK) signaling in human breast cancer tissue. **a**, Spatial signaling direction. **b**, Amount of received signal by each spot. **c**, Two examples of differentially expressed (DE) genes due to signaling. **d**, Identification of the differentially expressed genes due to the total amount of received signal in the MK signaling pathway. **e**, Unique impact on the identified differentially expressed genes by the individual ligand–receptor pairs. **f**,**g**, Signaling in mouse brain tissue. The signaling direction (left) and the level of received signal (right) are shown for PSAP signaling (**f**) and FGF signaling (**g**).

Finally, we applied COMMOT to signaling analysis with Visium^16^ spatial transcriptomics data, in which each spatial spot contains multiple cells. By analyzing the breast cancer data with 3,798 spots and 36,601 genes, we found clear spatial signaling directionality of midkine signaling, which was identified to be the most active (Fig. 5a), and the regions receiving such signals (Fig. 5b). To identify the genes that may be regulated by or regulate CCC, we used tradeSeq^53^ to perform a differential expression test, in which the amount of received midkine signaling was used as the cofactor, analogous to a temporal differential expression test in which pseudotime is used as the cofactor (Fig. 5c,d). COL1A1 was identified as a significant positive differentially expressed gene with a distinct spatial pattern, whereas S100G was a significant negative differentially expressed gene with its own unique spatial pattern (Fig. 5c). Furthermore, as the received midkine signaling increases, the level of COL1A1 expression increases while the S100G expression level decreases (Fig. 5d). Adapting temporal differentially expressed gene analysis methods for scRNA-seq data to the signaling differentially expressed gene analysis of spatial transcriptomics data identifies relationships between gene expression and signaling activity, for example, between COL1A1 expression and midkine signaling. In general, good coverage of genes and a large number of cells or spots is preferred for CCC-associated differentially expressed gene analysis of spatial transcriptomics data.

Differential expression tests typically examine the pairwise correlation between a potential target gene and a cofactor. The higher-order interactions between multiple factors (multiple potential upstream genes and the cofactor) are often neglected. To prioritize the genes that are more likely to be regulated by CCC, we used a random forest model^54,55^ in which the potential target gene is the output and the CCC cofactor and the top intracellular correlated genes are the input features. The feature importance of the cofactor in the trained model then served to quantify the unique information provided by the cofactor about the potential target gene, scoring the unique impact of individual ligand–receptor pairs on each of the identified signaling differentially expressed genes. This model showed that COL1A1 and S100G are distinctly impacted by various midkine ligand–receptor pairs (Fig. 5e). Such analysis may be carried out for any ligand–receptor pair expressed in the data, for example, the PD1 signaling pathway related to T-cell functions (Supplementary Fig. 20).

We also analyzed a Visium^16^ dataset of mouse brain tissue with 3,355 spots and 32,285 genes (Fig. 5f,g). We found significant prosaposin signaling activity across the tissue (Fig. 5f), where broad protective roles of prosaposin in the nervous system were discovered^56^, and fibroblast growth factor signaling was identified on the border of the cerebellar cortex (Fig. 5g), consistent with its known role in cerebellum patterning during development^57^.

### Robust identification of CCC direction and downstream target

To assess method robustness and efficiency we next studied the correlation between inferred CCC and the expression of known downstream genes, and compared COMMOT with three existing methods: CellChat^6^, which was designed for scRNA-seq data, and Giotto^23^ and CellPhoneDB v3^25^, which were designed for spatial transcriptomics data.

**Extended Data Fig. 6:**
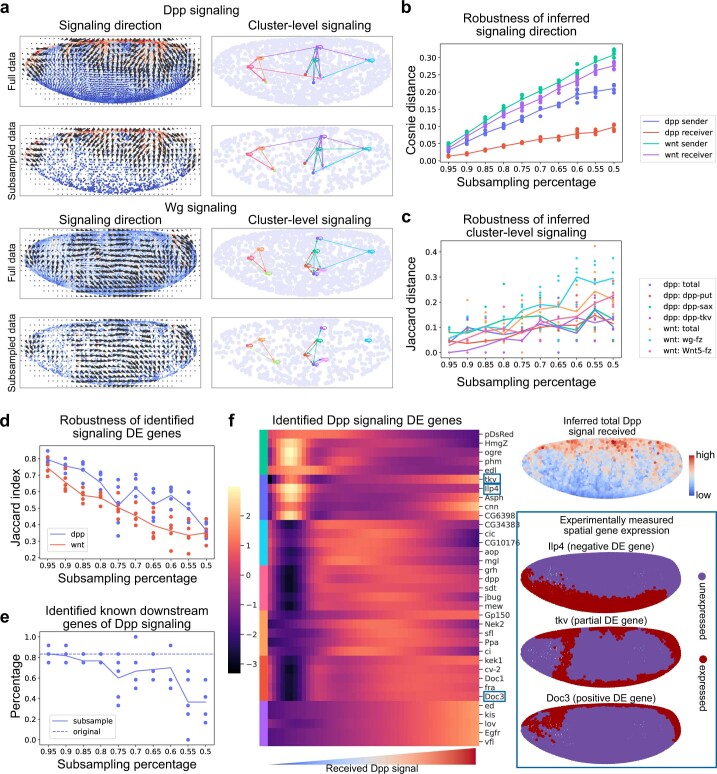
Robustness of CCC analysis on a well-studied drosophila embryo dataset. **a** Spatial signaling direction and signaling among cell clusters for Dpp and Wg signaling pathways. **b** Robustness of inferred signaling direction evaluated by comparing the direction obtained from subsampled dataset to the one from the full dataset using cosine distance. Each point is an independent test and the line shows the average of the tests. **c** Robustness of inferred cluster-level communication evaluated by comparing random subsamples to the full dataset using the Jaccard distance. **d** Robustness of downstream gene identification. **e** Percentage of known downstream genes that are identified as differentially expressed gene due to signaling activity. **f** Examples of the identified positively, negatively, and partially differentially expressed genes associated to Dpp signaling. For panels b–e, the averages of 5 independent random subsampling are plotted.

To test robustness, we used the stage 6 *Drosophila* embryo, an extensively studied system^58,59^. An in situ spatial transcriptomic map was generated by integrating an scRNA-seq dataset with spatial single-cell resolution data^60^ using SpaOTsc^31^. From subsampled data, COMMOT consistently identified CCC directions, cluster-level CCC and the signaling differentially expressed genes (Extended Data Fig. 6). See Methods for evaluation metrics and the Supplementary Note for more details.

Utilizing scSeqComm^61^, a database of known target genes of ligand–receptor pairs combining major resources including Reactome, TTRUST and RegNetwork, we investigated the correlation between the inferred signaling activities and the expression of the corresponding target genes. We used three datasets analyzed in the previous sections with transcriptome or near-transcriptome gene coverage: Visium human breast cancer data, Visium mouse brain data and seqFISH+ mouse somatosensory cortex data. COMMOT was used to quantify all available ligand–receptor pairs in the CellChatDB. At the individual-spot scale, Spearman’s correlation coefficient was computed for each ligand–receptor pair between the received signal and the average expression of the known downstream genes. The median correlations on the three datasets were 0.237, 0.180 and 0.230, respectively (Supplementary Fig. 21). At the cluster scale, we quantified the level of received signal using the average of the spots in the cluster.

We compared COMMOT with three methods that infer cluster-level CCC: CellChat^6^, Giotto^23^ and CellPhoneDB v3^25^. The activity of the downstream genes of a ligand–receptor pair was quantified as the percentage of significant positive differentially expressed genes of a cluster. By studying the correlation between the inferred CCC and the activity of known downstream genes, we found COMMOT to have a stronger correlation than the three methods for most datasets, and a comparable correlation to CellPhoneDB v3 in some cases (Supplementary Figs. 22–24). This evaluation can be further improved if more complete knowledge of gene regulation is available. With such a list, one may also formulate the evaluation as a classification problem. The differences between COMMOT and the three methods are illustrated in Supplementary Figs. 25–30 and discussed in the Supplementary Note. Furthermore, COMMOT can identify localized signaling hotspots compared with cluster-level approaches (Supplementary Figs. 31 and 32). For a specific ligand–receptor pair, COMMOT prioritizes regions containing its high signaling activity with low competition from other pairs (Supplementary Figs. 33 and34), showing its unique strength.

To study algorithm efficiency, we found that COMMOT running time scales linearly with the number of non-zero elements in the CCC (Supplementary Fig. 35). The number of non-zero elements in the CCC matrices scales linearly with the number of locations in spatial transcriptomics data due to the spatial range constraint, and the memory usage also scales linearly with the number of locations given that only the finite values of the cost matrix and the non-zero values of the CCC matrix need to be stored. Thus, COMMOT can effectively handle the existing spatial transcriptomics datasets given that both computing time and memory usage both scale linearly with the number of spatial locations.

## Discussion

To dissect CCC from the emerging spatial transcriptomics data we have developed COMMOT to infer CCC for all ligand and receptor species, simultaneously; visualize spatial CCC at various scales including a vector field visualization of spatial signaling directions; and analyze their downstream effects. This tool is based on collective optimal transport that incorporates both competing marginal distributions and constrained transport plans, two important features that cannot be dealt with using current variants of optimal transport.

We have studied a wide range of data types with different spatial resolutions and gene coverage: in silico spatial transcriptomics data obtained by integrating scRNA-seq and spatial staining data, Visium, Slide-seq, STARmap, MERFISH and seqFISH+ spatial transcriptomics. COMMOT could consistently capture the CCC activities known from the literature. In human skin, COMMOT showed that higher WNT signaling increases the expression of several genes, a result confirmed by immunofluorescence staining. We acknowledge that false positives in our inferred CCC are inherently possible because spatial transcriptomics data do not directly represent protein abundancy and our method cannot capture protein-specific modifications such as protein phosphorylation, glycosylation, proteolytic cleavage into fragments, and dimerization, which certainly affect the signaling functions and, thus, the CCC mechanisms that COMMOT aims to infer. The reliability of CCC predictions is expected to significantly improve as emerging spatial proteomics approaches mature.

The spatial distance constraint used to capture the effect of ligand diffusivity is usually determined by several factors, including protein weight and tortuosity of extracellular space^62^. It is difficult to accurately estimate this parameter for every pair in the database. In our model the local short-range interactions are emphasized even when the spatial distance range is increased (Supplementary Fig. 36). Thus, when screening many ligand–receptor pairs a uniform and relatively large spatial distance limit may be used to avoid missing important interactions. Once the important interactions are identified, an accurate estimation of this parameter would further refine the prediction to remove false-positive CCC links.

Most recently, several methods and packages have been introduced to study CCC with spatial transcriptomics data. SpatialDM^63^ evaluates the co-expression of ligand and receptor genes; SpaTalk^64^ and stMLnet^65^ are focused on signaling target genes; HoloNet^66^ studies the joint impact from different combinations of CCC events; and DeepLinc^67^ constructs de novo cell–cell interaction landscapes without the need for annotated ligand and receptor genes. Although COMMOT has a different focus, these methods arguably complement each other when studying different aspects of CCC.

With the foreseeable availability of temporal sequences of spatial transcriptomics data^68^, CCC dynamics may be elucidated, for example by extending collective optimal transport into a dynamic optimal transport formulation. The PDE model of CCC can be generalized to further incorporate the intracellular gene regulatory network. While traditional optimal transport is powerful at integrating a pair of datasets and multimarginal optimal transport^69^ integrates multiple datasets, the collective optimal transport is able to effectively control the coupling and deal with competing species, which is useful for a broad range of problems beyond CCC inference.

## Methods

Full details of the theoretical background and implementation of COMMOT can be found in the Supplementary Information.

### COMMOT model

COMMOT constructs a collection of CCC networks through various predefined ligand–receptor pairs (user-defined or from aggregated ligand–receptor interaction databases) by solving a global optimization problem that accounts for potential higher-order interactions between the multiple ligand and receptor species. To this end, we introduce collective optimal transport that determines a collection of optimal transport plans for all pairs of species that can be coupled simultaneously. As a result, the coupling between a species pair will affect other couplings and vice versa, which cannot be realized in traditional optimal transport^34^. The collective optimal transport results in a large-scale optimization problem for which new algorithms are needed, and thus we present one based on the efficient Sinkhorn iteration^70^.

For a spatial transcriptomics dataset of *n*_*s*_ spatial locations and a set of *n*_*l*_ ligand species and *n*_*r*_ receptor species, a collective optimal transport problem is formulated as follows:

1 $$\begin{array}{*{20}{c}} {\mathop {{{{{\mathrm{min}}}}}}\limits_{{{{\boldsymbol{P}}}} \in \Gamma } \mathop {\sum}\limits_{\left( {i,j} \right) \in I} {\left\langle {{{{\boldsymbol{P}}}}_{i,j, \cdot , \cdot },{{{\boldsymbol{C}}}}_{\left( {i,j} \right)}} \right\rangle _F} + \mathop {\sum}\limits_i {F\left( {\mu _i} \right)} + \mathop {\sum}\limits_j {F\left( {\nu _j} \right)} ,} \\ {\Gamma = \left\{ {{{{\boldsymbol{P}}}} \in {\Bbb R}_ + ^{n_l \times n_r \times n_s \times n_s}:{{{\boldsymbol{P}}}}_{i,j, \cdot , \cdot } = {{{\mathbf{0}}}}\,{{{\mathrm{for}}}}\,\left( {i,j} \right) \notin I,\mathop {\sum}\limits_{j,l} {{{{\boldsymbol{P}}}}_{i,j,k,l} \le {{{\boldsymbol{X}}}}_{i,k}^L} ,\mathop {\sum}\limits_{i,k} {{{{\boldsymbol{P}}}}_{i,j,k,l} \le {{{\boldsymbol{X}}}}_{j,l}^R} } \right\},} \\ {\mu _i\left( k \right) = {{{\boldsymbol{X}}}}_{i,k}^L - \mathop {\sum}\limits_{j,l} {{{{\boldsymbol{P}}}}_{i,j,k,l},\,\nu _j\left( l \right) = {{{\boldsymbol{X}}}}_{j,l}^R - \mathop {\sum}\limits_{i,k} {{{{\boldsymbol{P}}}}_{i,j,k,l}} } } \end{array}$$

where ${{{\boldsymbol{X}}}}_{i,k}^L$ is the expression level of ligand *i* on spot *k*, ${{{\boldsymbol{X}}}}_{j,l}^R$ is the expression level of receptor *j* on spot *l* and *F* penalizes the untransported mass **μ**_*i*_ and ***v***_*j*_. The coupling matrix ${{{\boldsymbol{P}}}}_{i,j,k,l}$ scores the signaling strength from spot *k* to spot *l* through the pair consisting of the ligand *i* and receptor *j* for $\left( {i,j} \right) \in I$ where *I* is the index set of ligand and receptor species that can bind. The cost matrix **C**_(*i,j*)_ is based on the thresholded distance matrix such that its *kl*-th entry equals *φ*(**D**_*k,l*_) if **D**_*k,l*_ ≤ *T*_(*i,j*)_ and infinity otherwise, where **D** is the Euclidean distance matrix for the distances between the spots, *T*_(*i,j*)_ is the spatial limit of signaling through the pair of ligand *i* and receptor *j*, and *φ* is a scaling function, such as square or exponential. When the ligands or receptors contain heteromeric units, the minimum of units is used by default in the package to represent the amount of ligand or receptor. For example, if receptor species *j* is composed of two subunits, the minimum of them in spot l is used to represent the level of this receptor species $\mathbf{X}^R_{j,l}$.

### Collective optimal transport algorithm

To solve the collective optimal transport problem described above, we rewrite the original problem as:

2 $$\begin{array}{*{20}{c}} {\mathop {{{{{\mathrm{min}}}}}}\limits_{\hat {{{\boldsymbol{P}}}},\hat{\boldsymbol{\mu}} ,\hat{\boldsymbol{\nu}} \ge 0} \langle {\hat {{{\boldsymbol{P}}}},\hat {{{\boldsymbol{C}}}}} \rangle _{{{F}}} + {\it{\epsilon }}_pH( {\hat {{{\boldsymbol{P}}}}} ) + {\it{\epsilon }}_\mu H\left( {\hat{\boldsymbol{\mu}} } \right) + {\it{\epsilon }}_\nu H\left( {\hat{\boldsymbol{\nu}} } \right) + \rho \left( {\left\| {\hat{\boldsymbol{\mu}} } \right\|_1 + \left\| {\hat{\boldsymbol{\nu}} } \right\|_1} \right),} \\ {{\mathrm{s.t.}}\,\hat {{{\boldsymbol{P}}}}{{{\mathbf{1}}}}^n = {{{\boldsymbol{a}}}} - \hat{\boldsymbol{\mu}} ,\,\hat {{{\boldsymbol{P}}}}^T{{{\mathbf{1}}}}^m = {{{\boldsymbol{b}}}} - \hat{\boldsymbol{\nu}} } \end{array}$$

where ${{{\hat{\boldsymbol P}}}}$ is obtained by reshaping $\boldsymbol{P}$ such that ${{{\hat{\boldsymbol P}}}}_{(i-1) \times n_s + k,\,(j-1) \times n_s + l} = {{{\boldsymbol{P}}}}_{i,j,k,l}$. The cost matrix ${{{\hat{\boldsymbol C}}}}$ is obtained similarly and we set ${{{\hat{\boldsymbol C}}}}_{(i-1) \times n_s + k,(j-1) \times n_s + l} = \infty$ for ligand *i* and receptor *j* that cannot bind. The marginal distributions are constructed such that ${{{\boldsymbol{a}}}}_{(i-1) \times n_s + k} = {{{\boldsymbol{X}}}}_{i,k}^L$ and ${{{\boldsymbol{b}}}}_{(j-1) \times n_s + l} = {{{\boldsymbol{X}}}}_{j,l}^R$. Entropy regularization is added to speed up computation and smooth the result with $H\left( {{{\boldsymbol{x}}}} \right) = \mathop {\sum}\nolimits_i {x_i\left( {\ln \left( {x_i} \right) - 1} \right)}$.

When the entropy regularization terms have the same coefficient values, ${\it{\epsilon }} = {\it{\epsilon }}_p = {\it{\epsilon }}_\mu = {\it{\epsilon }}_\nu$, the problem can be efficiently solved with a stabilized Sinkhorn iteraction^70^

3 $$\begin{array}{*{20}{c}} {\begin{array}{*{20}{c}} {{{{\boldsymbol{f}}}}^{\left( {l + 1} \right)} \leftarrow {\it{\epsilon }}\log {{{\boldsymbol{a}}}} + {{{\boldsymbol{f}}}}^{\,\left( l \right)} - {\it{\epsilon }}\log \left( {e^{\frac{{{{{\boldsymbol{f}}}}^{\left( l \right)}}}{{\it{\epsilon }}}} \odot e^{ - \frac{{{{\boldsymbol{C}}}}}{{\it{\epsilon }}}}e^{\frac{{{{{\boldsymbol{g}}}}^{\left( l \right)}}}{{\it{\epsilon }}}} + e^{\frac{{{{{\boldsymbol{f}}}}^{\left( l \right)} - \rho }}{{\it{\epsilon }}}}} \right),} \\ {{{{\boldsymbol{g}}}}^{\left( {l + 1} \right)} \leftarrow {\it{\epsilon }}\log {{{\boldsymbol{b}}}} + {{{\boldsymbol{g}}}}^{\left( l \right)} - {\it{\epsilon }}\log \left( {e^{\frac{{{{{\boldsymbol{g}}}}^{\left( l \right)}}}{{\it{\epsilon }}}} \odot e^{ - \frac{{{{{\boldsymbol{C}}}}^T}}{{\it{\epsilon }}}}e^{\frac{{{{{\boldsymbol{f}}}}^{\left( {l + 1} \right)}}}{{\it{\epsilon }}}} + e^{\frac{{{{{\boldsymbol{g}}}}^{\left( l \right)} - \rho }}{{\it{\epsilon }}}}} \right),} \end{array}} \end{array}$$

for *l* ≥ 0 with arbitrary initial $\boldsymbol{f}^{(0)}$ and $\boldsymbol{g}^{(0)}$. The resulting numerical solution to the optimization problem can be constructed by ${{{\hat{\boldsymbol P}}}}^ \ast = e^{({{{\boldsymbol{f}}}} \oplus {{{\boldsymbol{g}}}} - {{{\boldsymbol{C}}}})/{\it{\epsilon }}}$. The formulation in Eq. (2) solved by the algorithm in Eq. (3) was used to generate the results in this study. The derivation of the algorithm, and that of algorithms for the general case in which the regularization terms have different coefficients, is described in the Supplementary Information.

### Spatial signaling direction

To visualize the spatial signaling directions, we estimate a spatial vector field $V \in {\Bbb R}^{n_s \times d}$ of signaling directions given a CCC matrix ${{{\boldsymbol{S}}}} \in {\Bbb R}_ + ^{n_s \times n_s}$ obtained from collective optimal transport algorithm where $\boldsymbol{S}_{i,j}$ is the strength of the signal sent by spot *i* to spot *j*. The *i*th row of $\boldsymbol{V}$ represents the spatial signaling direction. We construct two vector fields, $\boldsymbol{V}^s$ and $\boldsymbol{V}^r$ describing the direction to/from which the spots are sending/receiving signals, respectively. Specifically, ${{{\boldsymbol{V}}}}_{i}^s = (\mathop {\sum}\nolimits_j\boldsymbol{S}_{i,j})\times\mathcal{N}\left(\mathop {\sum}\nolimits_{j \in N_i^s} {{{{\boldsymbol{S}}}}_{i,j}\mathcal{N}({{{{\boldsymbol{x}}}}_j - {{{\boldsymbol{x}}}}_i}})\right)$, where $\mathcal{N}(\mathbf{x})=\mathbf{x}\|\mathbf{x}$ and $N_i^s$ is the index set of top *k* signal-sending spots with the largest value on the *i*th row of **S**. Similarly, ${{{\boldsymbol{V}}}}_{i}^r = (\mathop {\sum}\nolimits_{j}\boldsymbol{S}_{j,i} )\times\mathcal{N}\left(\mathop {\sum}\nolimits_{j \in N_i^r} {{{{\boldsymbol{S}}}}_{j,i}\mathcal{N}({{{\boldsymbol{x}}}}_i - {{{\boldsymbol{x}}}}_j}) \right)$, where $N_i^r$ is the index set of top *k* signal-receiving spots with the largest value on the *i*th column of **S**.

### Cluster-level CCC

To elucidate CCC among cell states or local groups of spots, we aggregate the spot-by-spot CCC matrix $\boldsymbol{S}$ to a cluster-by-cluster matrix $\boldsymbol{S}^{cl}$. The signaling strength from cluster *i* to cluster *j* is quantified as ${{{\boldsymbol{S}}}}_{i,j}^{cl} = \mathop {\sum}\nolimits_{(k,l) \in I_{i,j}^{cl}} {{{{\boldsymbol{S}}}}_{k,l}}/|I_{i,j}^{cl}|$, where $\boldsymbol{I}_{i,j}^{cl} = \left\{ {\left( {k,l} \right):L_k = i,\,L_l = j} \right\}$ and *L*_*k*_ is the cluster label of spot *k*. The significance (*P* value) of the cluster-level CCC is determined by performing *n* independent permutations of the cluster labels and computing the percentile of the original signaling strength in the signaling strengths resulting from these label permutations. Permuting cluster labels after computing the spot-level CCC matrices may neglect communications between different clusters. To address this limitation, we provide an option that randomly permutes the locations of all spots or the spots within each cluster and then computes the spot-level CCC matrices.

### Evaluation metrics

The spatial signaling direction is described by a vector field defined on a discretized tissue space consisting of *n* grid points and is represented by an array ${{{\boldsymbol{V}}}} \in {\Bbb R}^{n \times d}$. The cosine distance is used to compare the vector field $\boldsymbol{V}_{\mathrm{sub}}$ from subsampled data with the one from the full data $\boldsymbol{V}_{\mathrm{full}}$ and is defined as

$$\begin{array}{l}d_{\mathrm{cos}}\left( {{{{\boldsymbol{V}}}}_{{{{\mathrm{full}}}}},\,{{{\boldsymbol{V}}}}_{{{{\mathrm{sub}}}}}} \right) =\\ \mathop {\sum}\nolimits_i {\left\| {{{{\boldsymbol{V}}}}_{{{{\mathrm{full}}}}}(i)} \right\|\left[ {1 - {{{\boldsymbol{V}}}}_{{{{\mathrm{full}}}}}\left( i \right) \cdot {{{\boldsymbol{V}}}}_{{{{\mathrm{sub}}}}}\left( i \right)/\left( {\left\| {{{{\boldsymbol{V}}}}_{{{{\mathrm{full}}}}}(i)} \right\|\left\| {{{{\boldsymbol{V}}}}_{{{{\mathrm{sub}}}}}(i)} \right\|} \right)} \right]/\mathop {\sum}\nolimits_i {\left\| {{{{\boldsymbol{V}}}}_{{{{\mathrm{full}}}}}\left( i \right)} \right\|.} }\end{array}$$

To compare two cluster-level CCC networks ${{{\boldsymbol{S}}}}_1^{cl}$ and ${{{\boldsymbol{S}}}}_2^{cl}$, we first binarize them such that the edges with *P* < 0.05 are kept in the edge sets ${{{\bar{\boldsymbol S}}}}_1^{cl}$ and ${{{\bar{\boldsymbol S}}}}_2^{cl}$. Then, the Jaccard distance is used for quantitative comparison, $d_{{{{\mathrm{Jaccard}}}}}\left( {{{{\bar{\boldsymbol S}}}}_1^{cl},{{{\bar{\boldsymbol S}}}}_2^{cl}} \right) = 1 - \left| {{{{\bar{\boldsymbol S}}}}_1^{cl} \cap {{{\bar{\boldsymbol S}}}}_2^{cl}} \right|/\left| {{{{\bar{\boldsymbol S}}}}_1^{cl} \cup {{{\bar{\boldsymbol S}}}}_2^{cl}} \right|$.

The Spearman’s correlation coefficient is used to quantify the correlation between the inferred signaling activity and the activity of the known target genes across the cell clusters, defined as ${\mathop{{{\rm{cov}}}}} \left( {{\mathop{\rm R}\nolimits} \left( {{{{\boldsymbol{X}}}}^{LR}} \right),{\mathop{\rm R}\nolimits} \left( {{{{\boldsymbol{X}}}}^{tgt}} \right)} \right)/\left( {\sigma _{{\mathop{\rm R}\nolimits} \left( {{{{\boldsymbol{X}}}}^{LR}} \right)}\sigma _{{\mathop{\rm R}\nolimits} \left( {{{{\boldsymbol{X}}}}^{tgt}} \right)}} \right)$, where ${{{\boldsymbol{X}}}}_i^{LR}$ i s the average received signal through a ligand–receptor pair in cell cluster *i*, and ${{{\boldsymbol{X}}}}_i^{tgt}$ is the activity of the known target genes of this ligand–receptor pair in cell cluster *i* quantified as the percentage of differentially expressed genes. The function R converts the vectors into ranks and *σ* is the standard deviation of the rank variables.

### Downstream gene analysis

After computing the CCC matrix $\boldsymbol{S}$ of a ligand–receptor pair or a signaling pathway, genes that are potential downstream targets of the corresponding CCC can be identified. The amount of signal received by each spot is quantified by ${{{\boldsymbol{r}}}} \in {\Bbb R}^{n_s}$ where ${{{\boldsymbol{r}}}}_i = \mathop {\sum}\nolimits_j {{{{\boldsymbol{S}}}}_{j,i}}$. Then the tradeSeq package^53^ is used to identify the genes that are differentially expressed with respect to $\boldsymbol{r}$, which we call differentially expressed CCC genes.

The identified differentially expressed CCC genes may be regulated by other genes in cells through gene regulation. To further prioritize the downstream genes, the expressions of which are affected by CCC, we train a random forest regression model^54,55^ that takes a potential downstream gene as the output, and $\boldsymbol{r}$ and a collection of highly correlated genes as input features. The unique impact of CCC on this potential downstream gene is quantified by the feature importance (Gini importance computed as the mean of total impurity decrease in each tree) of $\boldsymbol{r}$ in the trained random forest model. The inclusion of highly correlated genes in a cell as input features emphasizes the amount of information of potential target genes explained by inferred CCC, which is unlikely to be explained only by intracellular interactions. If such a dilution of importance is not preferred, the users may choose a smaller number of highly correlated genes as input features. The implementation in the scikit-learn package^55^ is used.

### CellChat, Giotto and CellPhoneDB analysis

For the CellChat analysis the spatial data were treated as non-spatial scRNA-seq data, and the count matrix was first normalized using the normalizeData function. The data were then filtered using the functions identifyOverExpressedGenes and identifyOverExpressedInteractions with the default parameters. The cluster-level communication scores in CellChat were computed using the computeCommunProb function with default parameters, and the results were further filtered using the filterCommunication function with min.cells set to 10. The ligand–receptor pairs categorized under ‘Secreted Signaling’ in the CellChatDB were examined. For Giotto analysis, the count data were first normalized using the normalizeGiotto function with default parameters. A spatial network was then created using the createSpatialNetwork function with the *k*-nearest neighbors method and *k* set to 100 and the maximum distance threshold of 1000 μm for Visium data and 500 μm for seqFISH+ data. The heteromeric ligand–receptor pairs in CellChatDB were converted to pairs of individual subunits. The spatCellCellcom function was then used to generate the cluster-level communication scores with the adjust_method set to fdr. For CellPhoneDB v3 analysis, the distance between clusters was quantified as the average distance between cells from the pair of clusters. The command ‘cellphonedb method statistical_analysis’ was used to generate CellPhoneDB results with the threshold parameter set to 0.1.

### Immunostaining and fluorescence in situ hybridization

Frozen tissue sections (10 μm) were fixed with 4% paraformaldehyde in PBS for 15 min. Ten percent BSA in PBS was used for blocking. Following blocking, 5% BSA and 0.1% Triton X-100 in PBS was used for permeabilization. The following antibodies were used: mouse anti-KRT5 (1:100; Santa Cruz Biotechnology, sc-32721), mouse anti-KRT15 (1:100; Santa Cruz Biotechnology, sc-47697), mouse anti-BCAM (1:100; Santa Cruz Biotechnology, sc-365191), mouse anti-FGF7 (1:100; Santa Cruz Biotechnology, sc-365440), mouse anti-STMN1 (1:100; Santa Cruz Biotechnology, sc-48362); mouse anti-IGFBP6 (1:500; Abgent, AP6764b); mouse anti-PMAIP1 (1:100; Santa Cruz Biotechnology, sc-56169), mouse anti-POSTN (1:100; Santa Cruz Biotechnology, sc-398631); mouse anti-FLG (1:100; Santa Cruz Biotechnology, sc-66192); rabbit anti-LOR (1:1000; abcam, ab85679); mouse anti-TYRO3 (1:100; LSBio, LS-C114523-100); rabbit anti-GAS6 (1:100; abcam, ab227174); and rabbit anti-PROS1 (1:100; Proteintech, 16910-1-AP). Secondary antibodies include Alexa Fluor 488 (1:500; Jackson ImmunoResearch, 715-545-150, 711-545-152) and Cy3 AffiniPure (1:500; Jackson ImmunoResearch, 711-165-152, 111-165-003). Slides were mounted with Prolong Diamond Antifade Mountant containing DAPI (Molecular Probes). Confocal images were acquired at room temperature (22.2 ºC) on a Zeiss LSM700 laser scanning microscope with a Plan-Apochromat ×20 objective or ×40 and ×63 oil immersion objectives.

Frozen neonatal human foreskin tissue sections were used for RNA in situ hybridization using RNAscope kit v2 (323100, Advanced Cell Diagnostics) as per the manufacturer’s instructions. The following *Homo sapiens* probes from Advanced Cell Diagnostics were used: Tyro3 probe (429611), Gas6 (427811-C2) and Pros1 (506991-C2). Confocal images were acquired at room temperature on an Olympus FV3000 confocal microscope with a Plan-Apochromat ×20 objective or ×40 and ×60 oil immersion objectives.

### Reporting summary

Further information on research design is available in the Nature Portfolio Reporting Summary linked to this article.

## Online content

Any methods, additional references, Nature Portfolio reporting summaries, source data, extended data, supplementary information, acknowledgements, peer review information; details of author contributions and competing interests; and statements of data and code availability are available at 10.1038/s41592-022-01728-4.

## Supplementary information

Supplementary Information Supplementary Figs. 1–36, Supplementary Note

Reporting Summary

Peer Review File

## Data Availability

The original public data used in this work can be accessed through the following links: *Drosophila* embryo spatial and scRNA-seq data: Dream Single cell Transcriptomics Challenge through Synapse ID (syn15665609)^60^; human epidermal scRNA-seq data^39^: GEO accession code GSE147482 (protocols involving human skin data were approved by the Institutional Review Board of the University of California, Irvine); mouse hypothalamic preoptic region MERFISH data^43^: original data available at Dryad^71^ at the link 10.5061/dryad.8t8s248 (this work used the preprocessed data through the Squidpy package^22^ with the utility squidpy.datasets.merfish); mouse placenta STARmap data^46^: downloaded from Code Ocean (https://codeocean.com/capsule/9820099/tree/v1) with the 10.24433/CO.6072400.v1; mouse brain STARmap data^20^: processed data were downloaded from the same repository as the mouse placenta STARmap data; mouse somatosensory cortex seqFISH+ data^18^: downloaded through the Giotto package^23^; mouse hippocampus Slide-seqV2 data^52^: downloaded from the Broad Institute Single Cell Portal (https://singlecell.broadinstitute.org/single_cell/study/SCP815/sensitive-spatial-genome-wide-expression-profiling-at-cellular-resolution#study-summary); breast cancer Visium data: downloaded from the 10X Genomics website (https://www.10xgenomics.com/resources/datasets/human-breast-cancer-block-a-section-1-1-standard-1-1-0); mouse brain (sagittal posterior) Visium data: downloaded from the 10X Genomics website (https://www.10xgenomics.com/resources/datasets/mouse-brain-serial-section-1-sagittal-anterior-1-standard-1-1-0). The ligand–receptor pairs with secreted ligands, as categorized in the CellChatDB^6^, were used and can be accessed at http://www.cellchat.org/cellchatdb/. The downstream target genes were taken from scSeqComm^61^ and the target gene libraries TF_TG_TRRUSTv2 and TF_TG_TRRUSTv2_RegNetwork_High_mouse were used for human and mouse, respectively.

## Extended data

are available for this paper at 10.1038/s41592-022-01728-4.

## Code availability

The open-source software is available at https://github.com/zcang/COMMOT. The code for reproducing the presented analysis results is available at 10.5281/zenodo.7272562 (ref. ^72^).
