# Supplementary material for: Screening cell–cell communication in spatial transcriptomics via collective optimal transport
Source: Nat Methods. 2023 Jan 23;20(2):218–28. doi: 10.1038/s41592-022-01728-4 (PMC9911355; doi:10.1038/s41592-022-01728-4)
Supplement: Supplementary file 1 — Supplementary Figs. 1–36, Supplementary Note [file 41592_2022_1728_MOESM1_ESM.pdf]

nature methods

Article

https://doi.org/10.1038/s41592-022-01728-4

Screening cell–cell communication in spatial transcriptomics via collective optimal transport

In the format provided by the authors and unedited

Supplementary Information

Screening cell-cell communication in spatial transcriptomics via collective optimal transport

Zixuan Cang, Yanxiang Zhao, Axel A. Almet, Adam Stabell, Raul Ramos,
Maksim V. Plikus, Scott X. Atwood, Qing Nie

Supplementary Figures

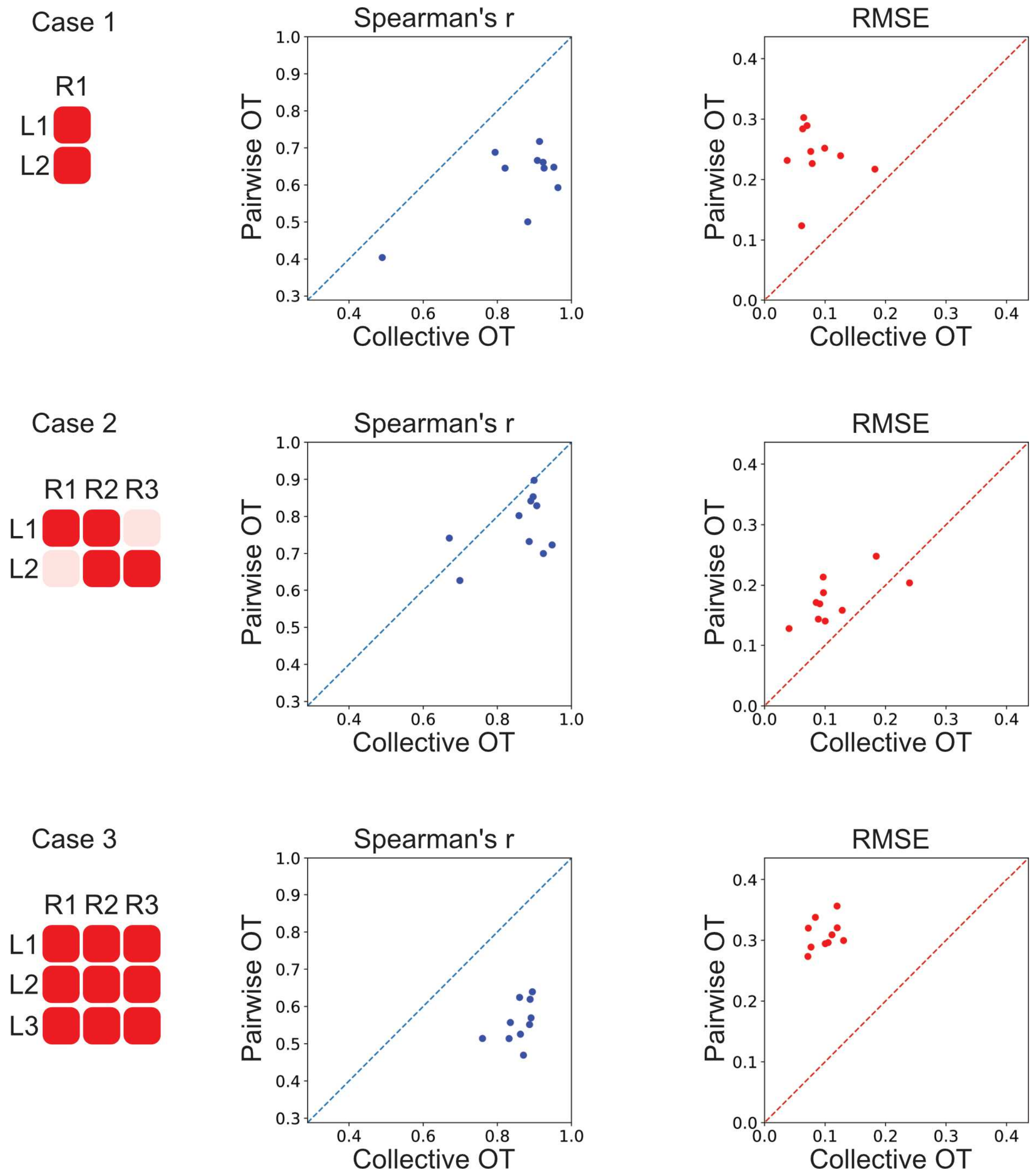

Supplementary Figure 1

Performance of collective optimal transport (benchmark cases 1-3)

The performance of collective optimal transport and traditional pairwise optimal transport obtained by comparison with benchmarks (cases 1-3) from PDE simulations. Each benchmark case has a specified number of ligand species and receptor species and a specific binding pattern among the species. The dark red color annotates the ligand-receptor pairs that can bind. The performance is measured by root-mean-squared error (RMSE) and Spearman’s correlation coefficient among the grid points.

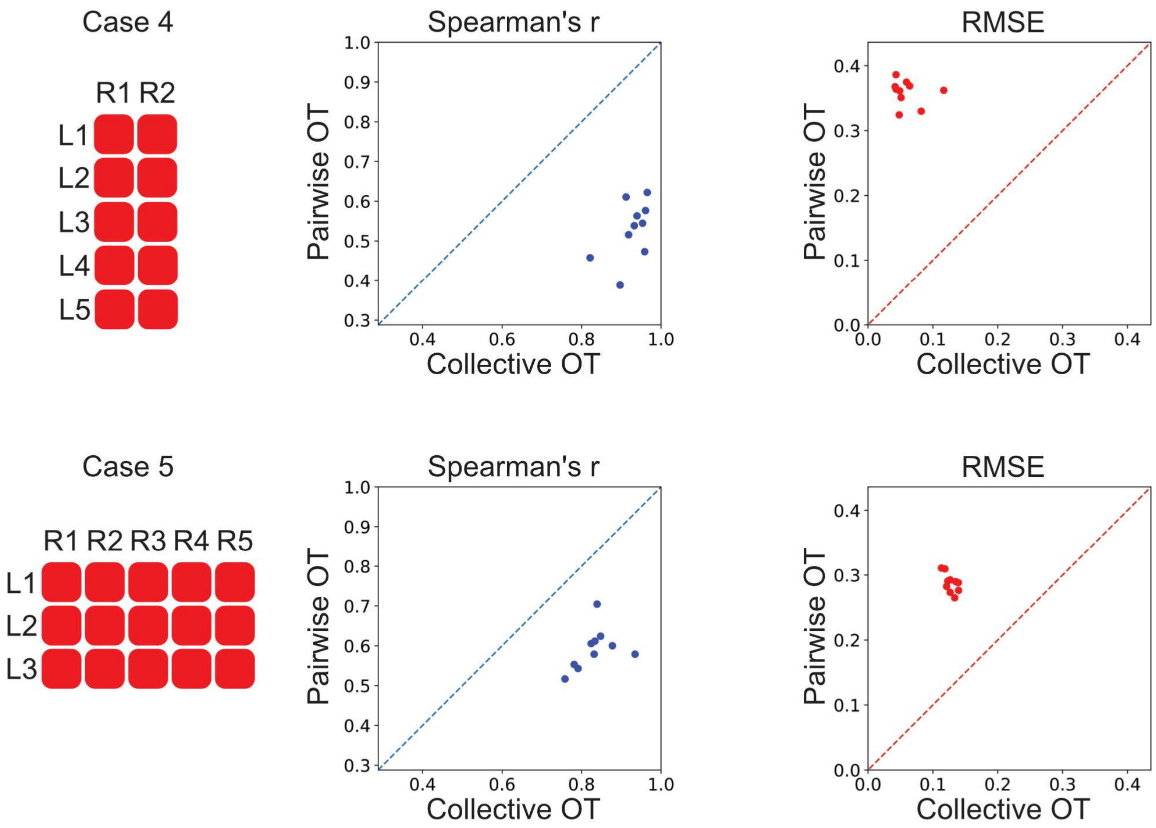

Supplementary Figure 2

Performance of collective optimal transport (benchmark cases 4-5)

The performance of collective optimal transport and traditional pairwise optimal transport obtained by comparison with benchmarks (cases 4-5) from PDE simulations. Each benchmark case has a specified number of ligand species and receptor species and a specific binding pattern among the species. The dark red color annotates the ligand-receptor pairs that can bind. The performance is measured by root-mean-squared error (RMSE) and Spearman's correlation coefficient among the grid points.

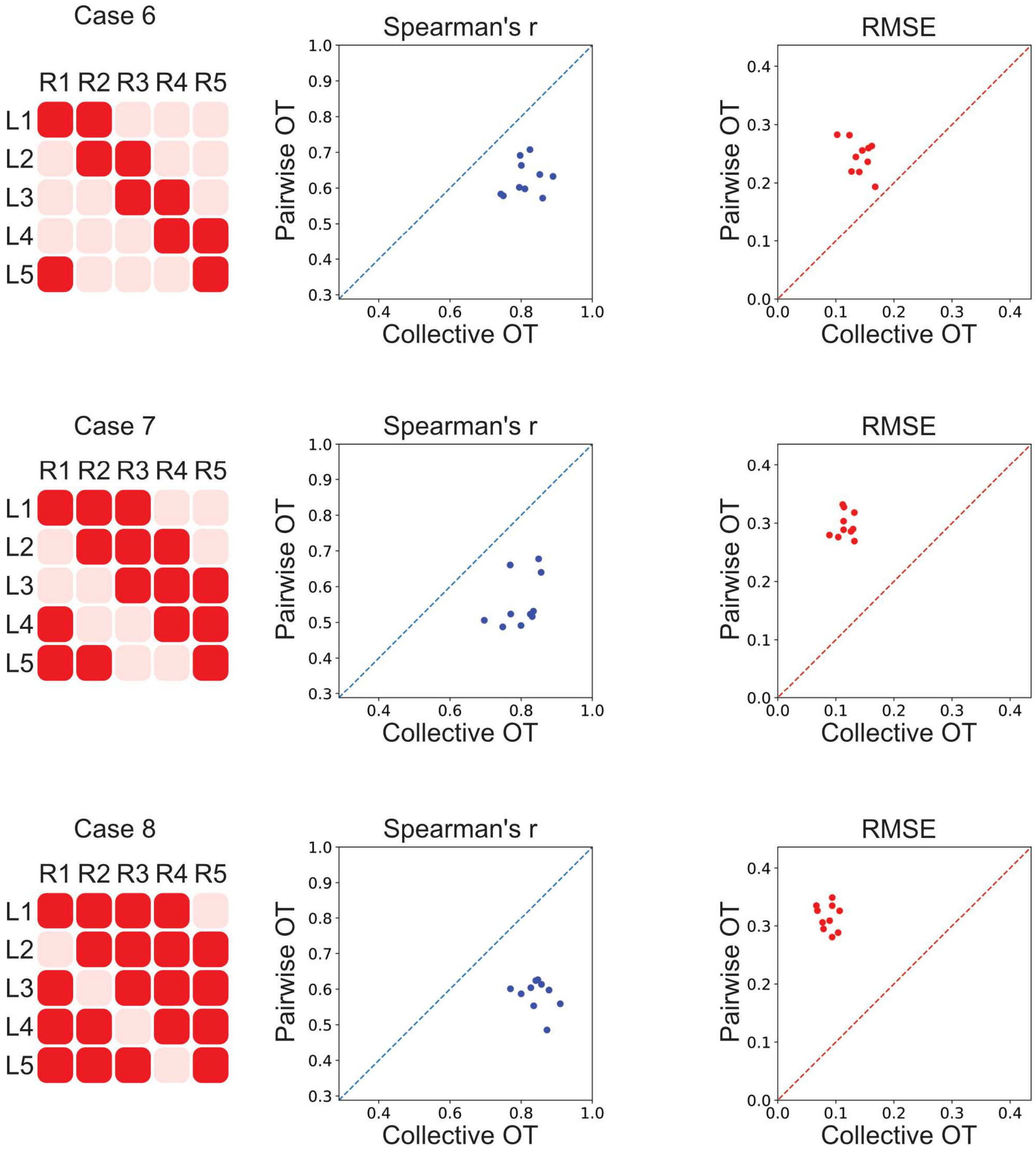

Supplementary Figure 3

Performance of collective optimal transport (benchmark cases 6-8)

The performance of collective optimal transport and traditional pairwise optimal transport obtained by comparison with benchmarks (cases 6-8) from PDE simulations. Each benchmark case has a specified number of ligand species and receptor species and a specific binding pattern among the species. The dark red color annotates the ligand-receptor pairs that can bind. The performance is measured by root-mean-squared error (RMSE) and Spearman's correlation coefficient among the grid points.

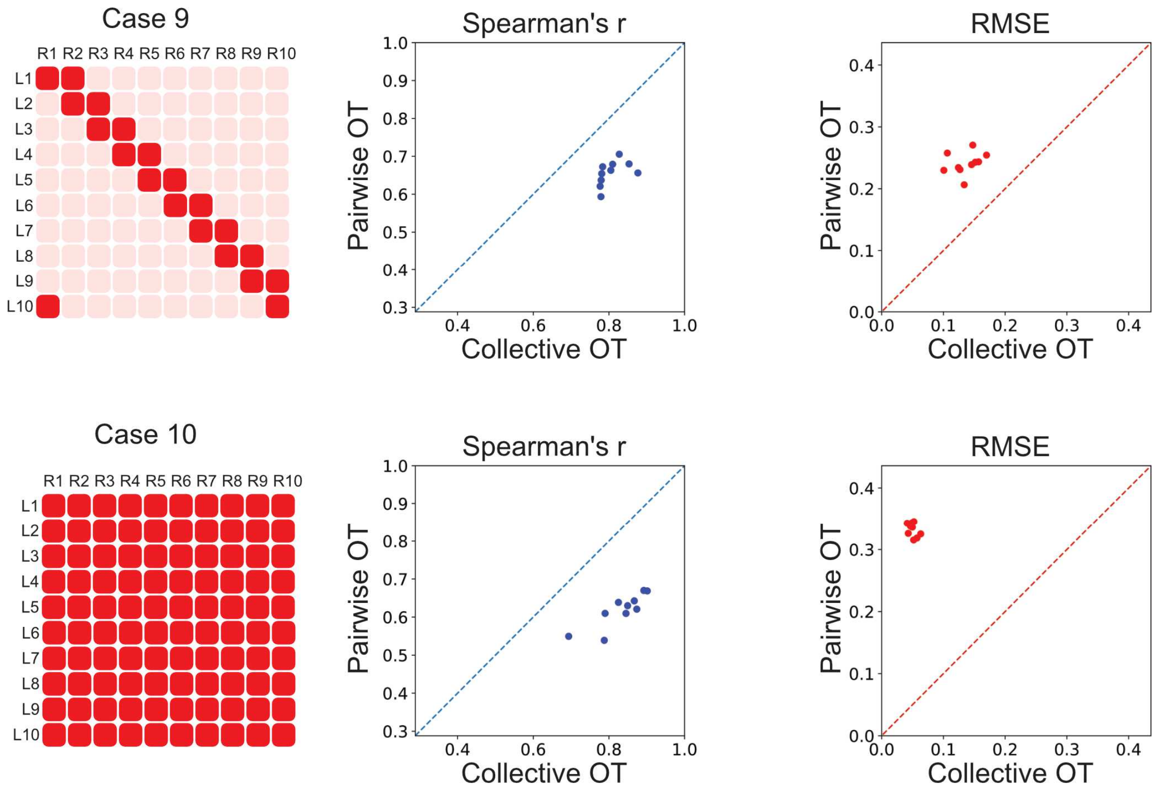

Supplementary Figure 4

Performance of collective optimal transport (benchmark cases 9-10)

The performance of collective optimal transport and traditional pairwise optimal transport obtained by comparison with benchmarks (cases 9-10) from PDE simulations. Each benchmark case has a specified number of ligand species and receptor species and a specific binding pattern among the species. The dark red color annotates the ligand-receptor pairs that can bind. The performance is measured by root-mean-squared error (RMSE) and Spearman's correlation coefficient among the grid points.

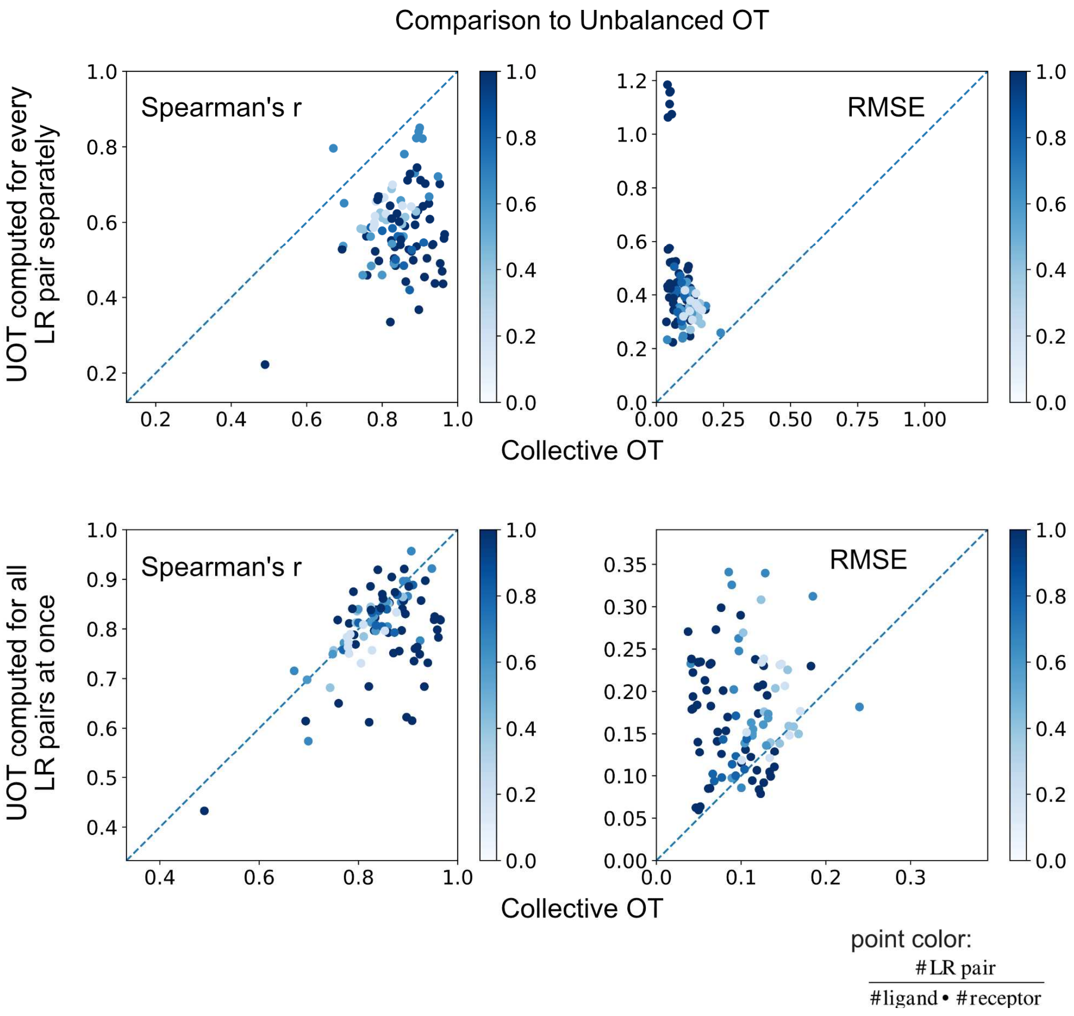

Supplementary Figure 5

Comparison with unbalanced optimal transport

The comparisons with unbalanced optimal transport that examines each ligand-receptor pair separately (top) or all pairs together (bottom). All analyses use the same spatial cutoff such that the transport cost is set to infinity if the distance between a location pair exceeds the distance cutoff. The optimal transport analysis results are evaluated by comparing the inferred amount of received signal to those obtained by simulation using Spearman’s correlation coefficient and root-mean-squared error.

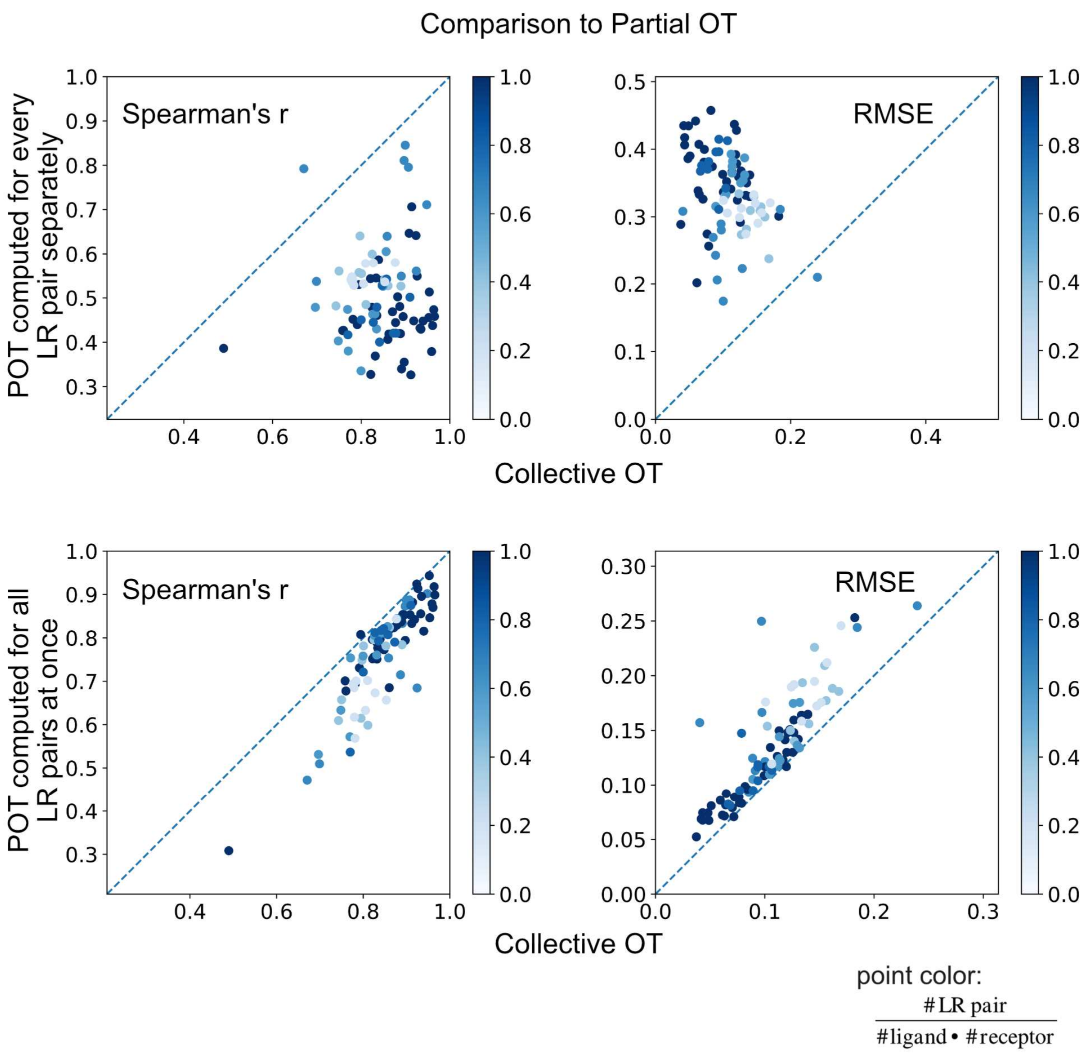

Supplementary Figure 6

Comparison with partial optimal transport

The comparisons with partial optimal transport that examines each ligand-receptor pair separately (top) or all pairs together (bottom). All analyses use the same spatial cutoff such that the transport cost is set to infinity if the distance between a location pair exceeds the distance cutoff. The optimal transport analysis results are evaluated by comparing the inferred amount of received signal to those obtained by simulation using Spearman's correlation coefficient and root-mean-squared error.

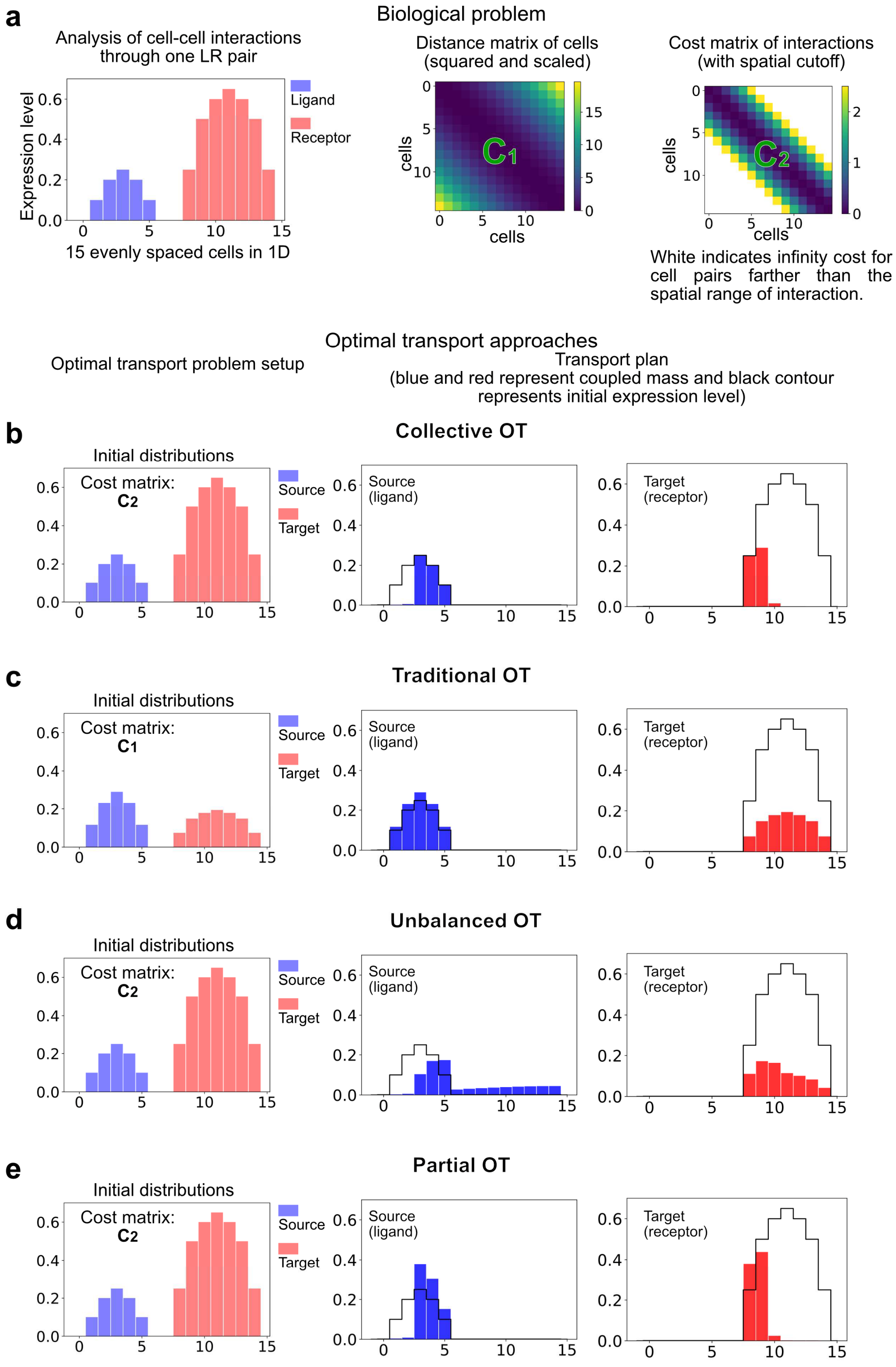

Supplementary Figure 7

Illustration of differences among several optimal transport variants

a The problem of fifteen cells placed one unit apart in 1D through one ligand-receptor pair with a spatial range cutoff of five units. **b-e** The input distributions and cost matrices of each optimal transport approach and the marginal distributions of the optimal transport plans. All three variants (collective OT, unbalanced OT, and partial OT) can take the initial unnormalized distributions and the cost matrix with infinity entries. However, unbalanced OT and partial OT result in artificial extra amount of ligand and receptor exceeding their expression levels. Traditional OT also induces artificial expressions due to normalization and cannot enforce the spatial range of signaling. Collective OT controls the coupled mass by the initial distributions and enforces the spatial range of signaling.

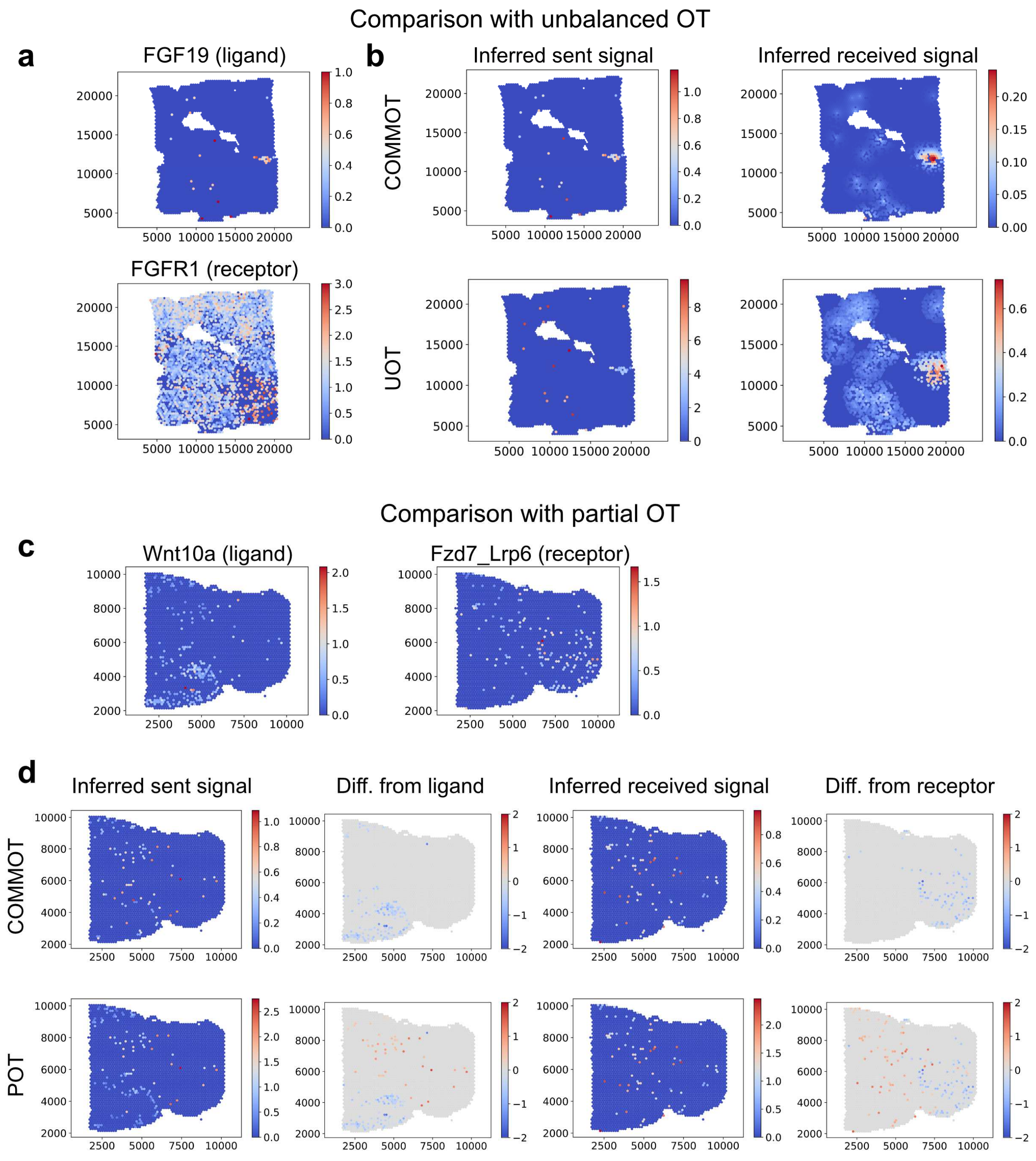

Supplementary Figure 8

Differences between OT variants on example Visium datasets

a The difference between COMMOT and unbalanced OT is demonstrated with a Visium dataset of human breast cancer focusing on the ligand-receptor pair FGF19-FGFR1 whose total expression levels are significantly different. **b** The amount of sent and received signal is plotted. Compared to COMMOT, unbalanced OT overestimates the signaling activity. Specifically, the amount of sent signal inferred using unbalanced OT significantly exceeds the expression level of ligand. **c** The difference between COMMOT and partial OT is demonstrated with a Visium dataset of mouse brain focusing on the ligand-receptor pair Wnt10a-Fzd7_Lrp6 whose total expression levels are comparable but are expressed in spatially distinct regions. **d** The amounts of sent, received signal and their difference to the expression levels of ligand and receptor are plotted. Compared to partial OT, COMMOT further prioritizes potential signal senders and receivers. The difference plots show that the sent and received signal inferred using partial OT exceed ligand and receptor expressions in some regions leading to overestimation of signaling activity.

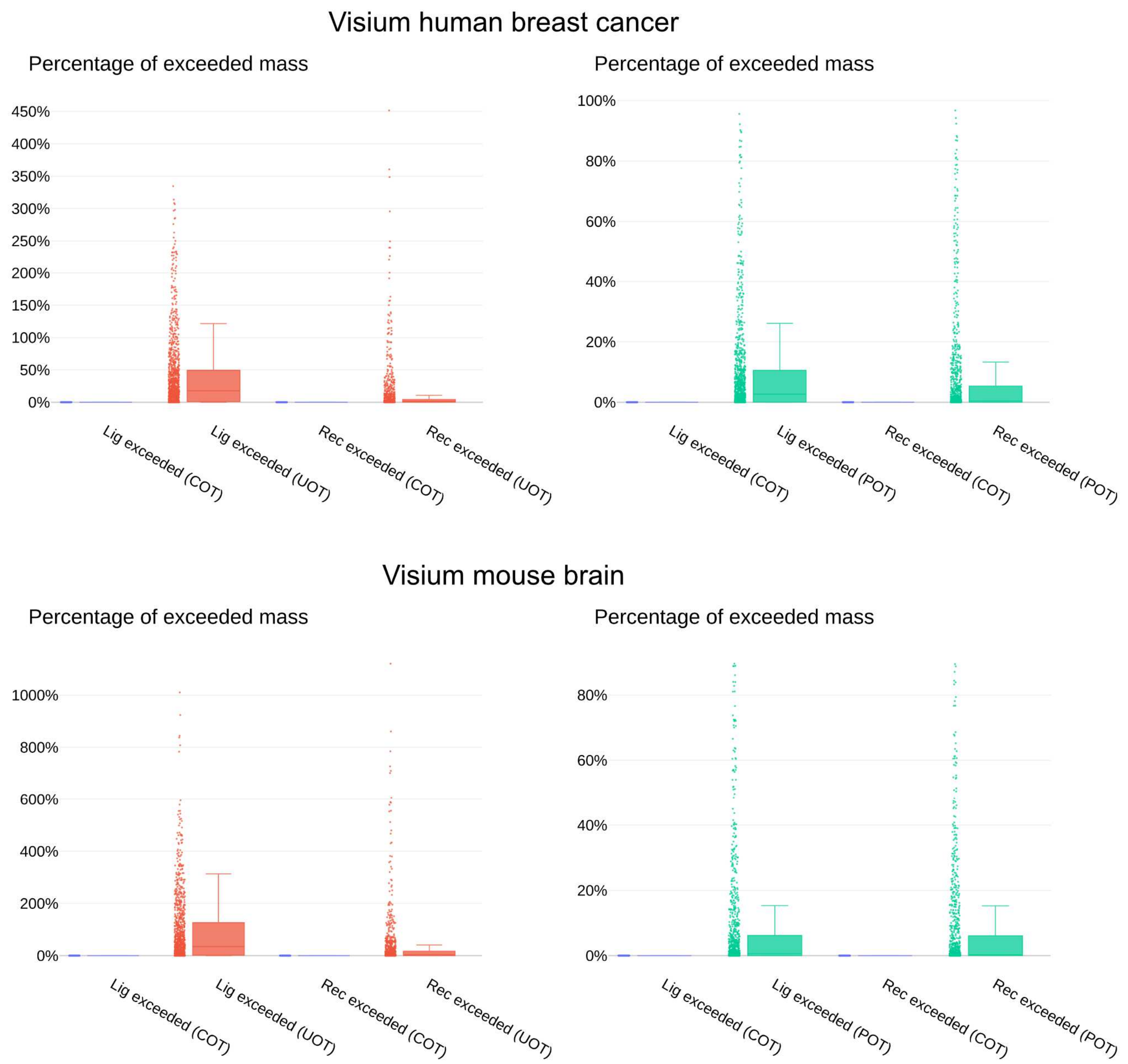

Supplementary Figure 9

Inferred sent or received signal exceeding the amount of ligand or receptors

Each dot represents a ligand-receptor pair. The commonly used KL divergence-based unbalanced optimal transport is used. All box plots show Q1 (25% quantile), Q2 (median), Q3 (75% quantile) as the box and the upper whisker represents Q3+1.5IQR. All data points are 0% for every COT experiments demonstrating no exceeded mass. For the Visium human breast cancer and Visium mouse brain data, the boxplots contain 1026 and 960 ligand-receptor pairs, respectively.

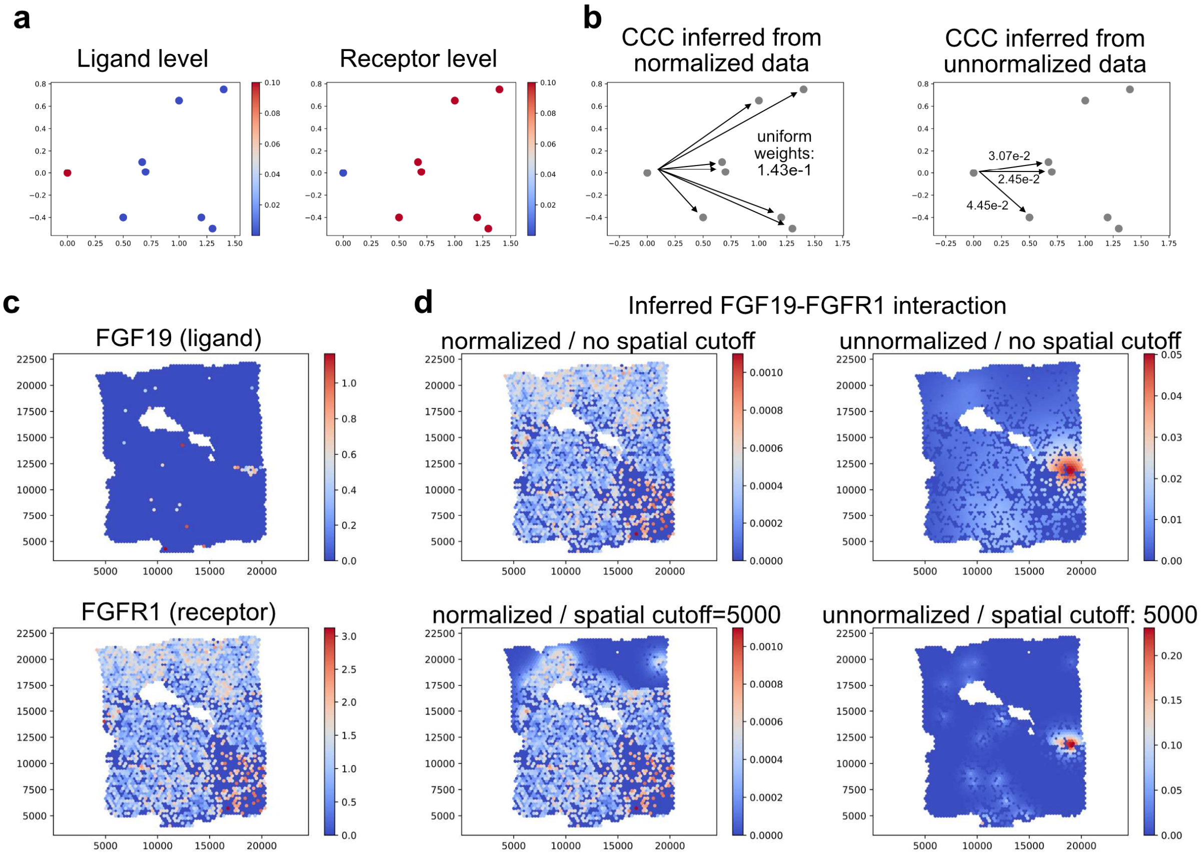

Supplementary Figure 10

Comparison of whether to normalize expression to probability distributions.

a An example with 8 cells where 1 cell expresses the ligand gene and the other 7 cells express the receptor gene. **b** The inferred cell-cell communication (CCC) with or without normalizing the expression levels to probability distributions. **c** An example ligand-receptor pair in a Visium human breast cancer data. **d** The inferred spatial distribution of received signals through the ligand-receptor pair FGF19-FGFR1 with or without normalization to probability distribution and with or without a spatial distance cutoff.

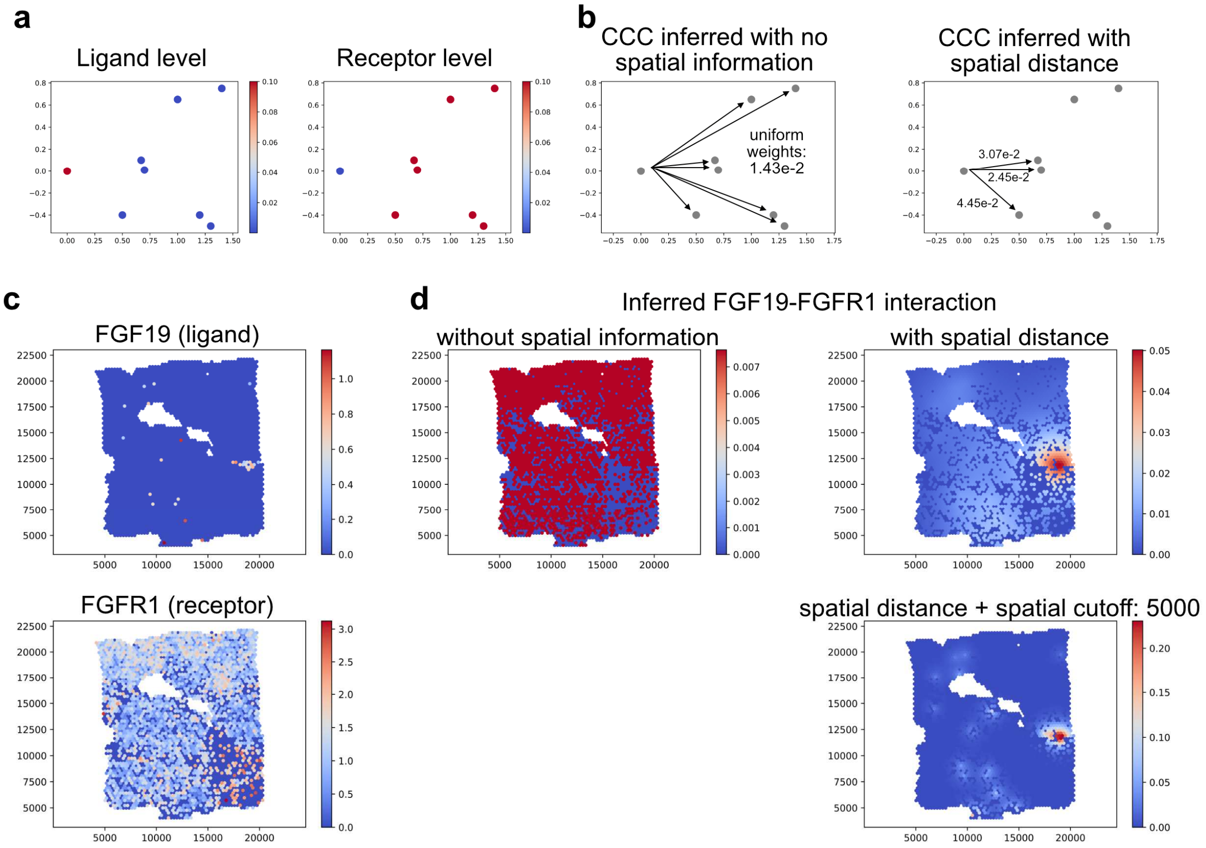

Supplementary Figure 11

Comparison of whether to incorporate spatial distance information.

a An example with 8 cells where 1 cell expresses the ligand gene and the other 7 cells express the receptor gene. **b** The inferred cell-cell communication (CCC) with or without normalizing the expression levels to probability distributions. Both results are from the spatial expression levels without normalizing to probability distributions. **c** An example ligand-receptor pair in a Visium human breast cancer data. **d** The inferred spatial distribution of received signals through the ligand-receptor pair FGF19-FGFR1 with or without incorporating spatial information and if spatial information is used, with or without a spatial distance cutoff.

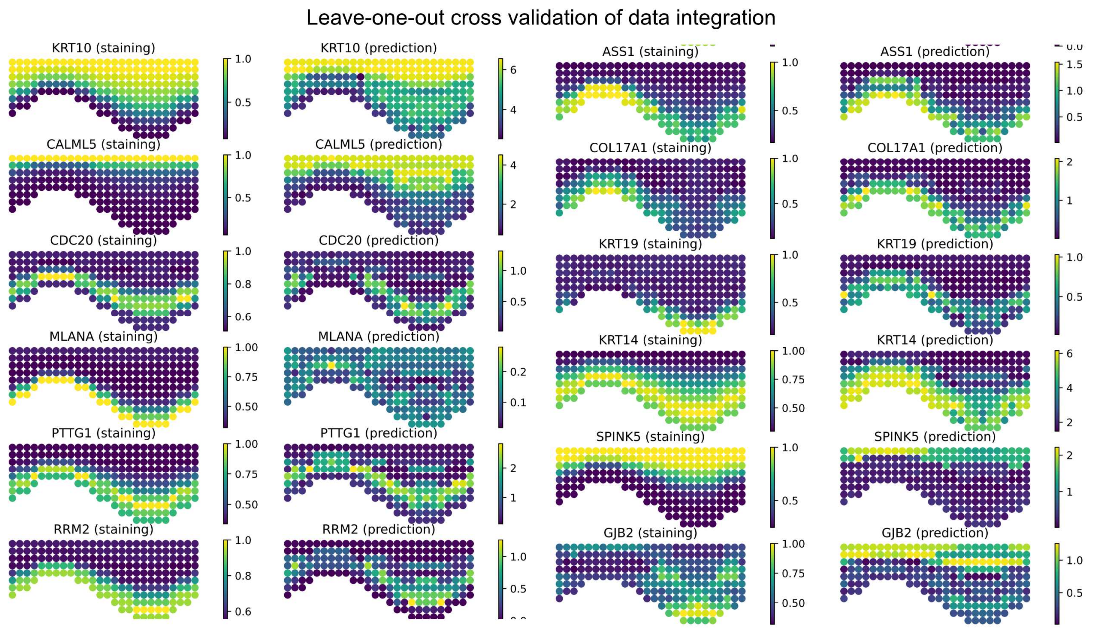

Supplementary Figure 12

Leave-one-out cross validation of scRNA-seq and spatial data integration for the human epidermis data

For each gene in the spatial data, the integration of scRNA-seq and spatial data is performed using the rest of the genes and the predicted spatial expression of this gene is compared to the ground-truth.

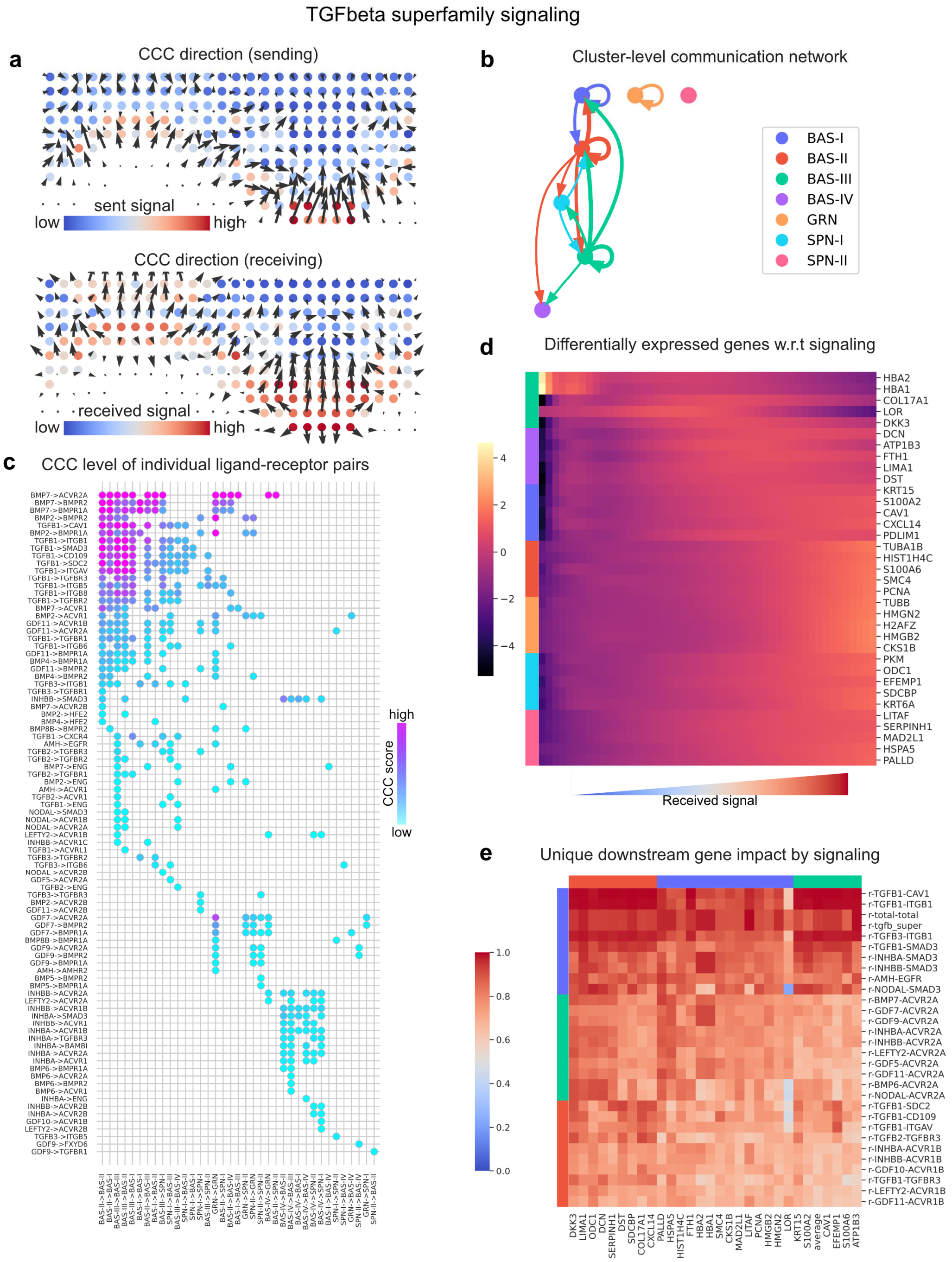

Supplementary Figure 13

TGFβ superfamily signaling in human skin

a Cell-cell communication directions. Sender: the direction shows to where the signal sending cells are sending the signal and color depicts the amount of signal sent by each cell. Receiver: the direction shows from where the signaling receiving cells are receiving the signal and color depicts the amount of signal received by each cell. **b** The cell-cell communication of all ligand-receptor pairs summarized to clusters (clustering identical to the recent NC paper), a thicker arrow means stronger signaling activity. **c** Dotplot showing the detailed signaling strength among the clusters through the individual ligand-receptor pairs. **d** The identified differentially expressed genes due to cell-cell communication activity. This is analogous to pseudotime DE genes but with the horizontal axis describing the amount of received signal. **e** Unique impact of CCC on downstream genes. A high score means the ligand-receptor pair is likely affecting the expression of the downstream gene considering the effects from other genes in the same cell.

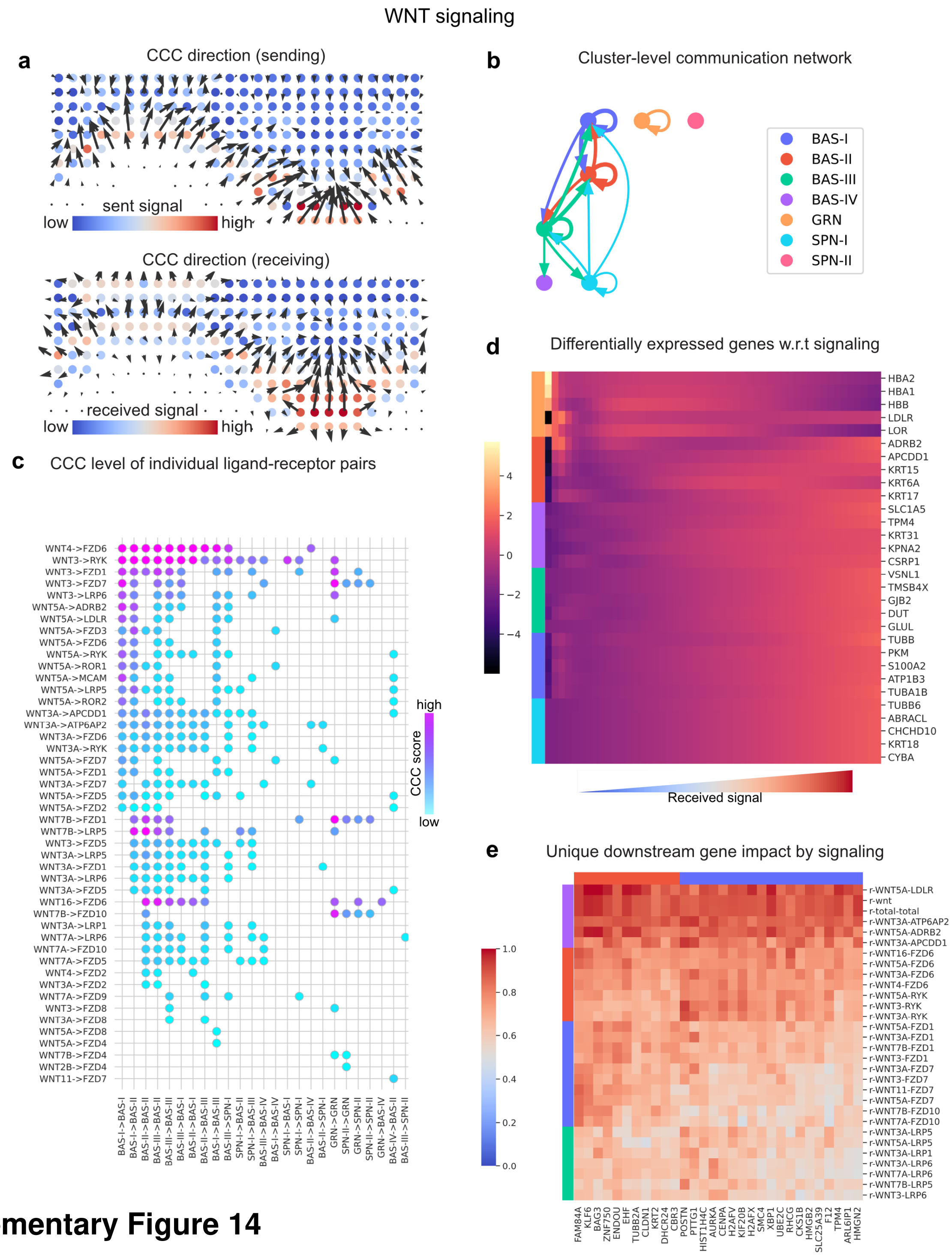

Supplementary Figure 14

WNT signaling in human skin

a Cell-cell communication directions. Sender: the direction shows to where the signal sending cells are sending the signal and color depicts the amount of signal sent by each cell. Receiver: the direction shows from where the signaling receiving cells are receiving the signal and color depicts the amount of signal received by each cell. **b** The cell-cell communication of all ligand-receptor pairs summarized to clusters (clustering identical to the recent NC paper), a thicker arrow means stronger signaling activity. **c** Dotplot showing the detailed signaling strength among the clusters through the individual ligand-receptor pairs. **d** The identified differentially expressed genes due to cell-cell communication activity. This is analogous to pseudotime DE genes but with the horizontal axis describing the amount of received signal. **e** Unique impact of CCC on downstream genes. A high score means the ligand-receptor pair is likely affecting the expression of the downstream gene considering the effects from other genes in the same cell.

JAK-STAT signaling

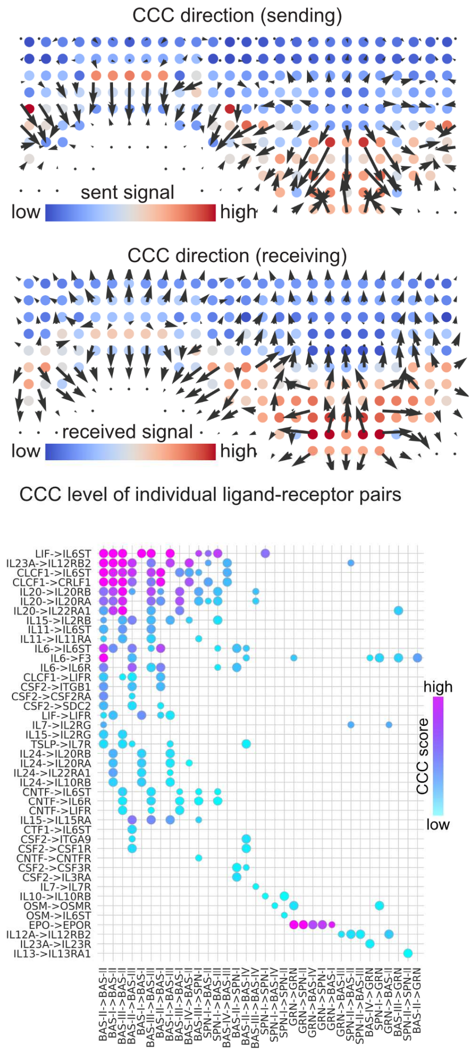

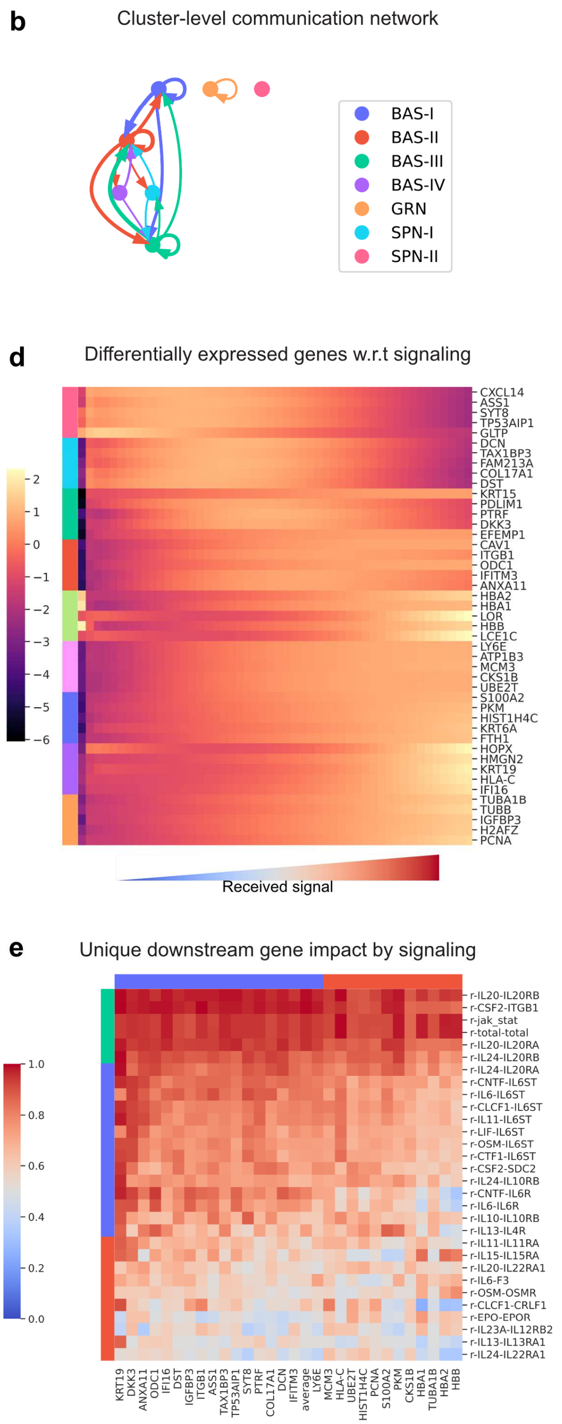

Supplementary Figure 15

JAK-STAT signaling in human skin

a Cell-cell communication directions. Sender: the direction shows to where the signal sending cells are sending the signal and color depicts the amount of signal sent by each cell. Receiver: the direction shows from where the signaling receiving cells are receiving the signal and color depicts the amount of signal received by each cell. **b** The cell-cell communication of all ligand-receptor pairs summarized to clusters (clustering identical to the recent NC paper), a thicker arrow means stronger signaling activity. **c** Dotplot showing the detailed signaling strength among the clusters through the individual ligand-receptor pairs. **d** The identified differentially expressed genes due to cell-cell communication activity. This is analogous to pseudotime DE genes but with the horizontal axis describing the amount of received signal. **e** Unique impact of CCC on downstream genes. A high score means the ligand-receptor pair is likely affecting the expression of the downstream gene considering the effects from other genes in the same cell.

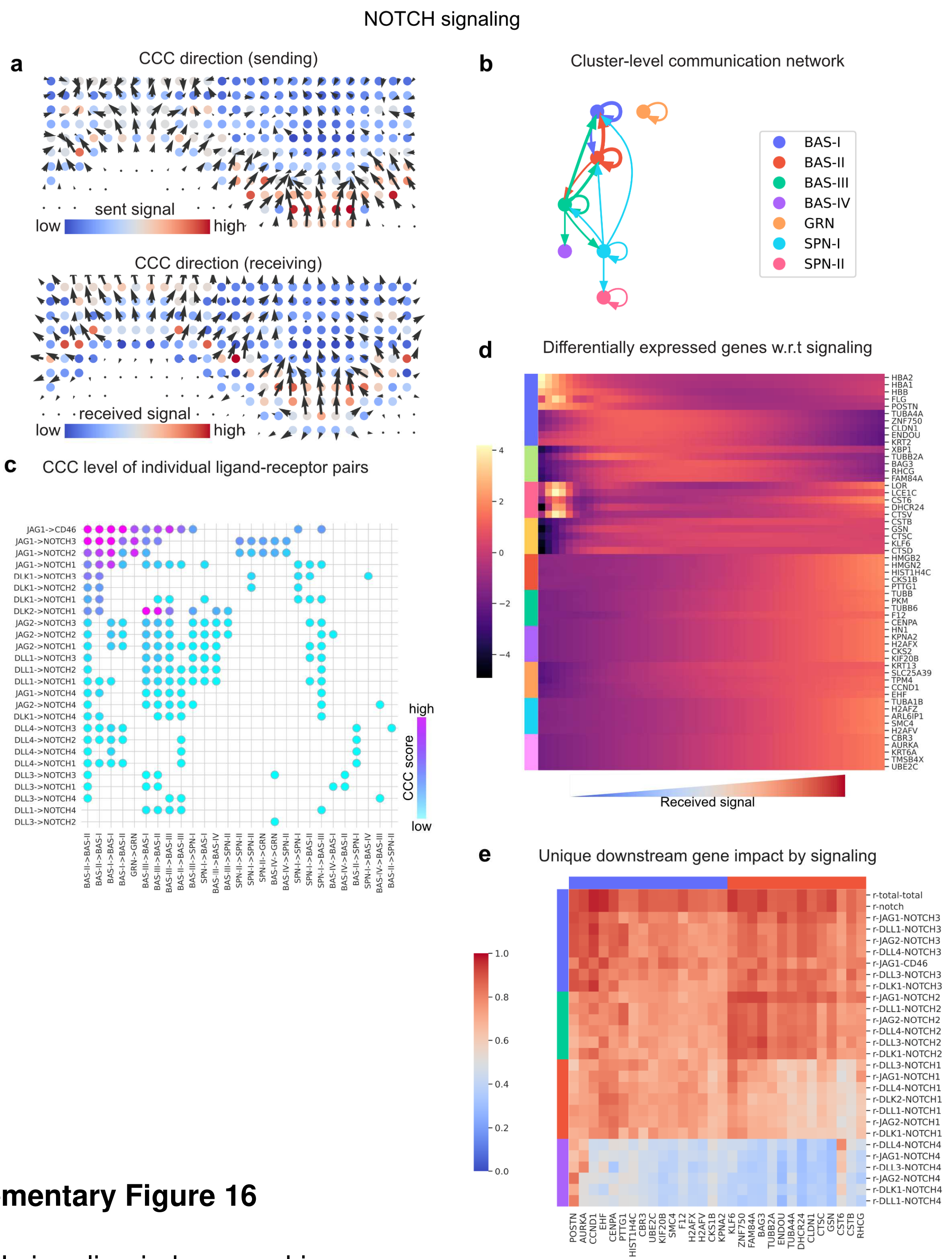

Supplementary Figure 16

NOTCH signaling in human skin

a Cell-cell communication directions. Sender: the direction shows to where the signal sending cells are sending the signal and color depicts the amount of signal sent by each cell. Receiver: the direction shows from where the signaling receiving cells are receiving the signal and color depicts the amount of signal received by each cell. **b** The cell-cell communication of all ligand-receptor pairs summarized to clusters (clustering identical to the recent NC paper), a thicker arrow means stronger signaling activity. **c** Dotplot showing the detailed signaling strength among the clusters through the individual ligand-receptor pairs. **d** The identified differentially expressed genes due to cell-cell communication activity. This is analogous to pseudotime DE genes but with the horizontal axis describing the amount of received signal. **e** Unique impact of CCC on downstream genes. A high score means the ligand-receptor pair is likely affecting the expression of the downstream gene considering the effects from other genes in the same cell.

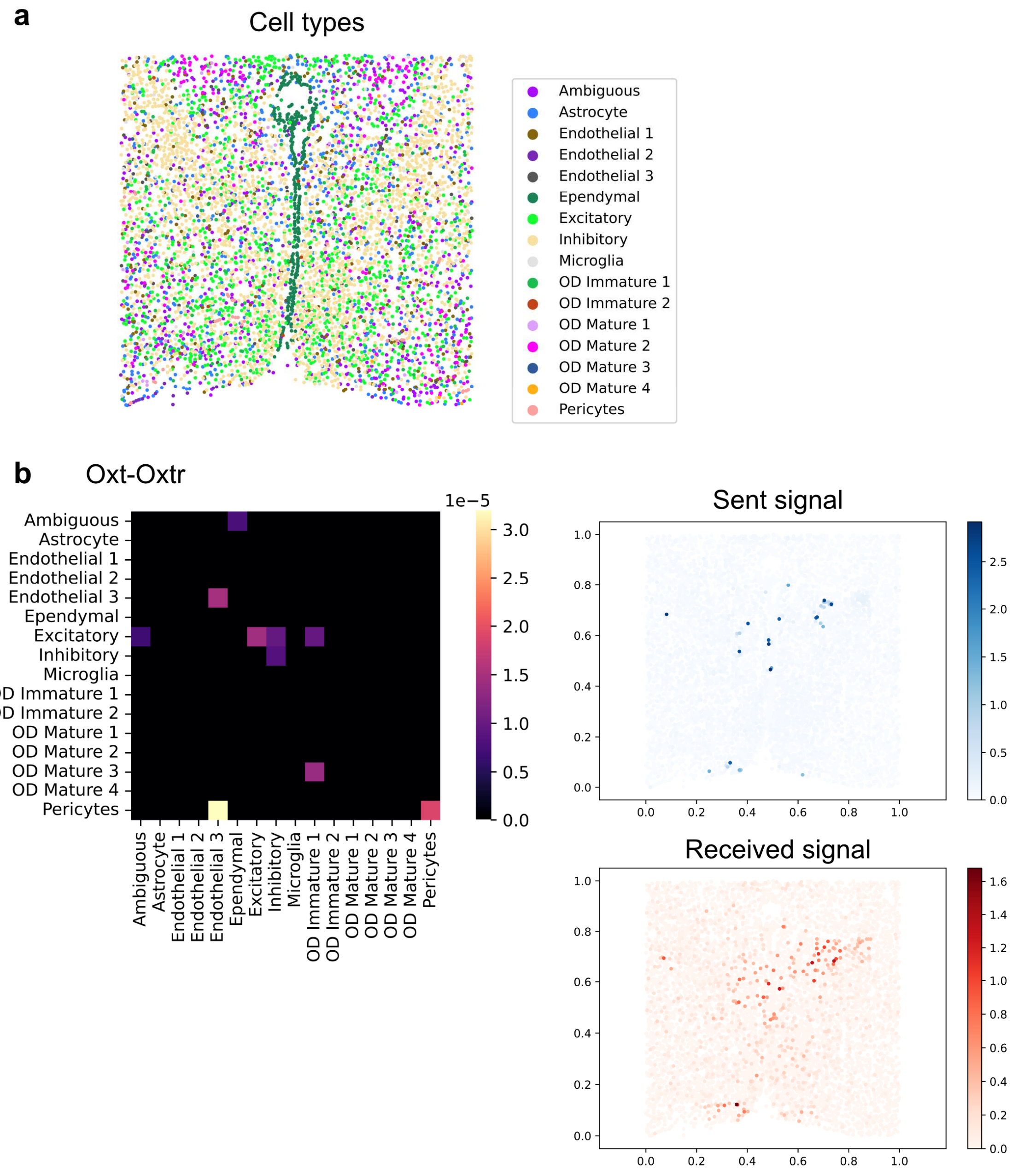

Supplementary Figure 17

OXT CCC in MERFISH mouse hypothalamic preoptic region

a Cell type plot of MERFISH mouse hypothalamic preoptic region data. **b** Heat map of cluster-level CCC where rows and columns correspond to senders and receivers, and the scatter plot of inferred sent and received signal through the ligand-receptor pair Oxt-Oxtr.

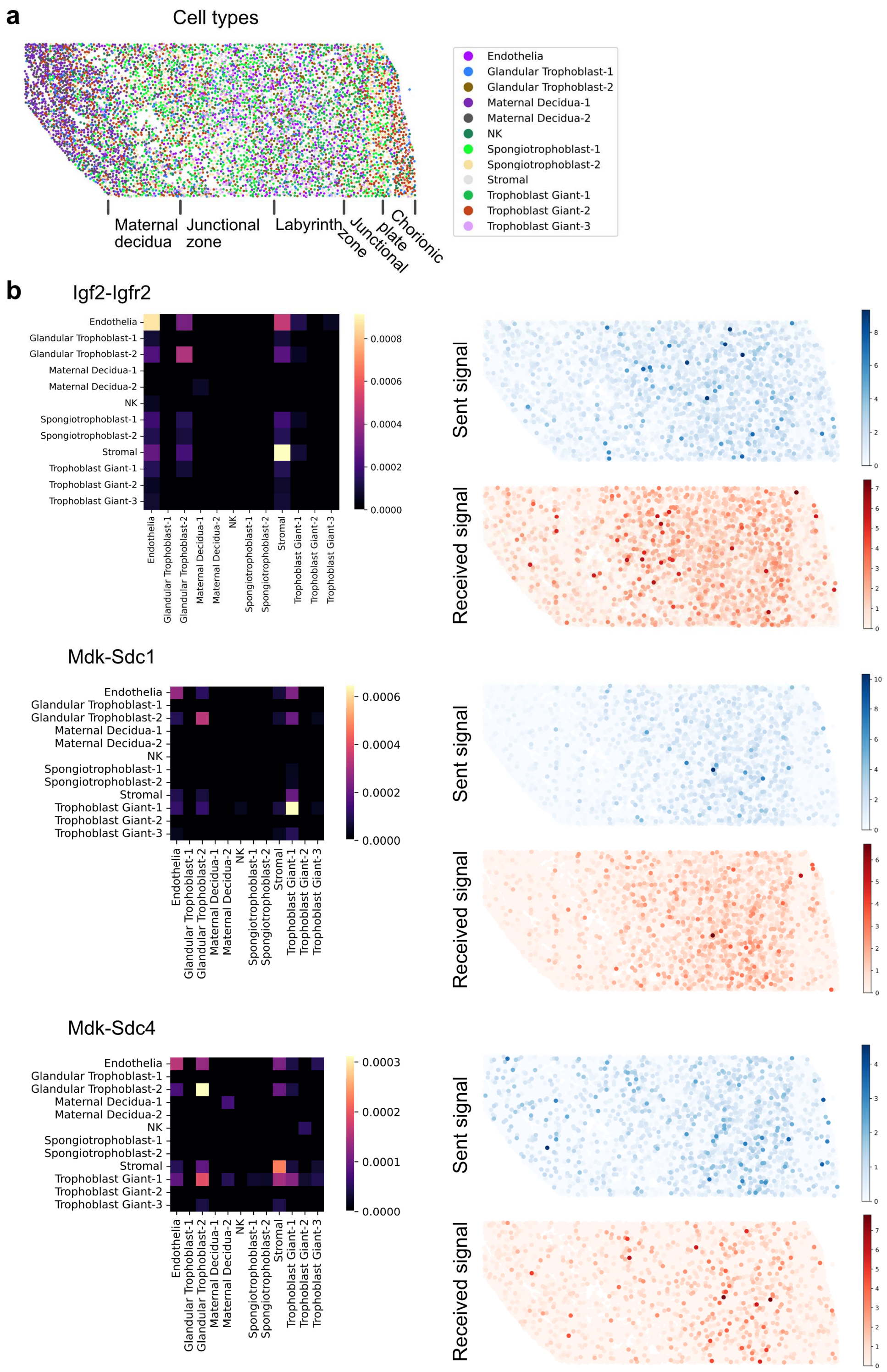

Supplementary Figure 18

CCC in STARmap mouse placenta data

a Cell type plot of the STARmap mouse placenta data. **b** Cluster-level CCC heatmaps where rows and columns correspond to signal senders and receivers respectively and the spatial distribution of sent and received signal through the ligand-receptor pairs Igf2-Igfr2, Mdk-Sdc1, and Mdk-Sdc4.

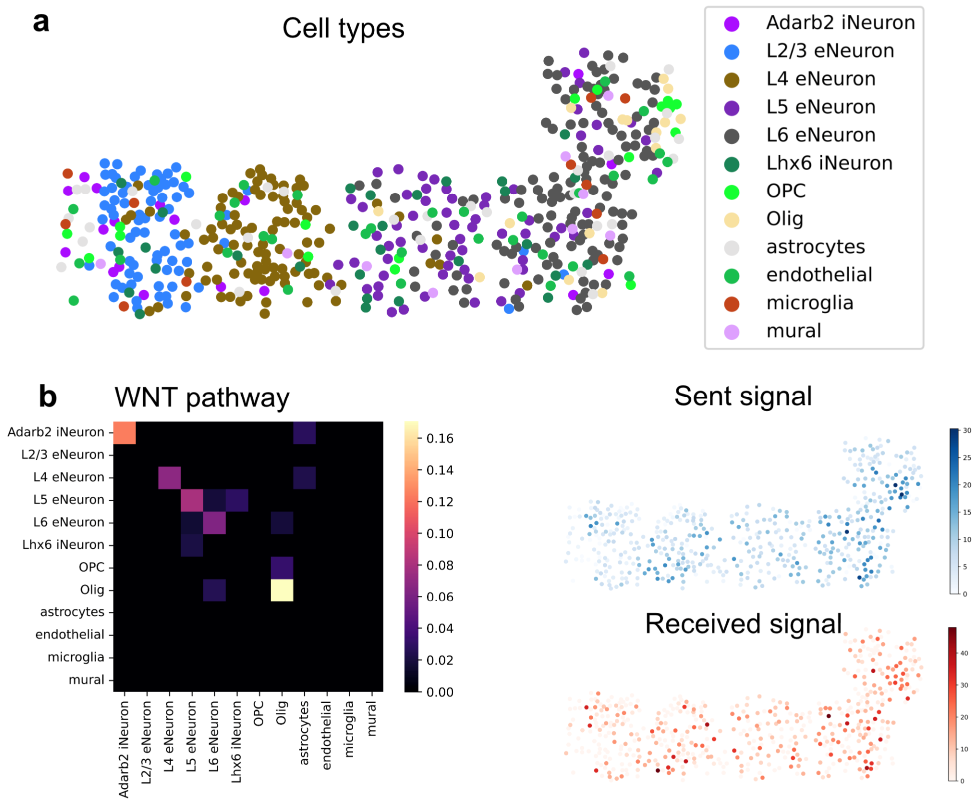

Supplementary Figure 19

WNT signaling in seqFISH+ mouse secondary somatosensory cortex

a Cell type plot of the seqFISH+ sscortex data. **b** Cluster-level CCC heatmaps where rows and columns correspond to signal senders and receivers respectively and the spatial distribution of sent and received signal through the ligand-receptor pairs from the WNT signaling pathway.

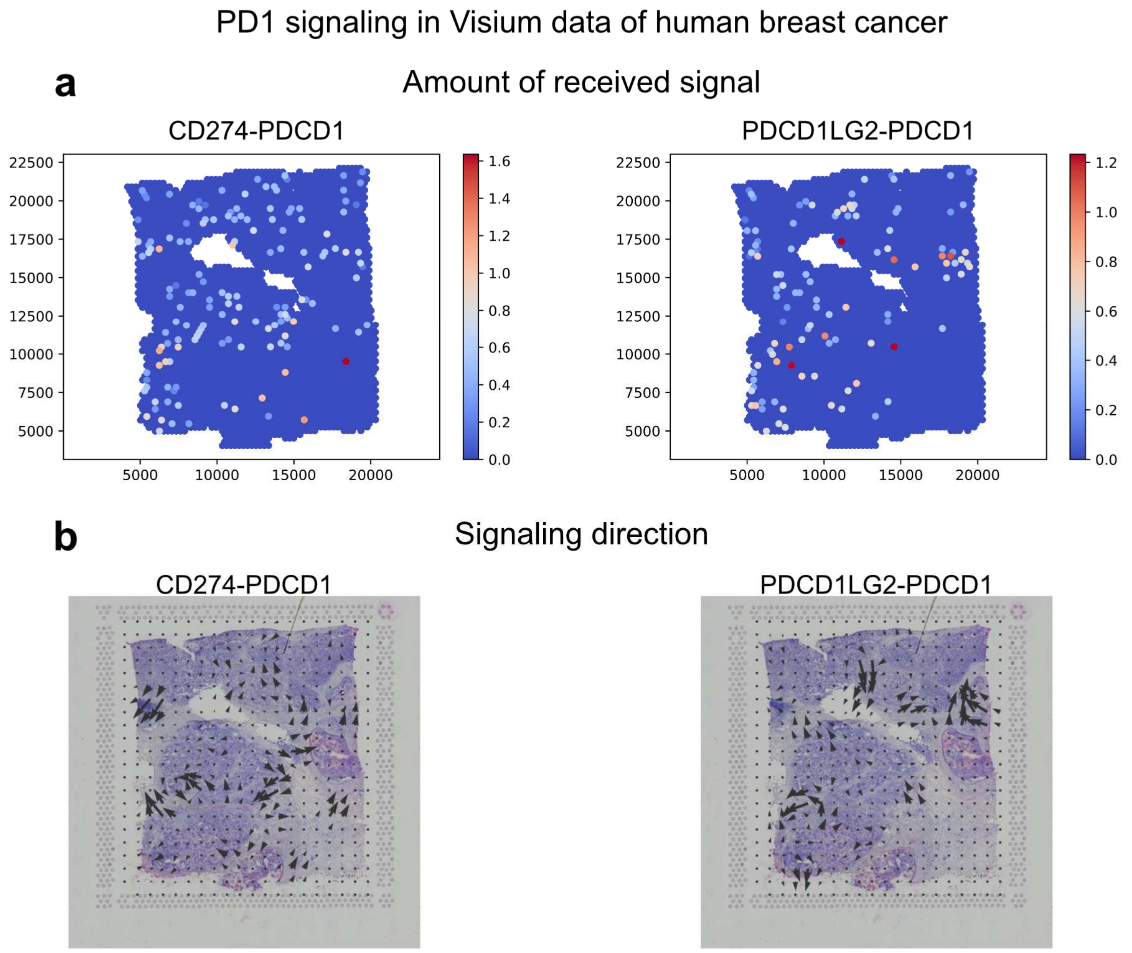

Supplementary Figure 20

PD1 signaling in the Visium data of human breast cancer.

a The amount of received signaling through two ligand-receptor pairs of PD1 signaling. **b** The signaling directions of the two ligand-receptor pairs.

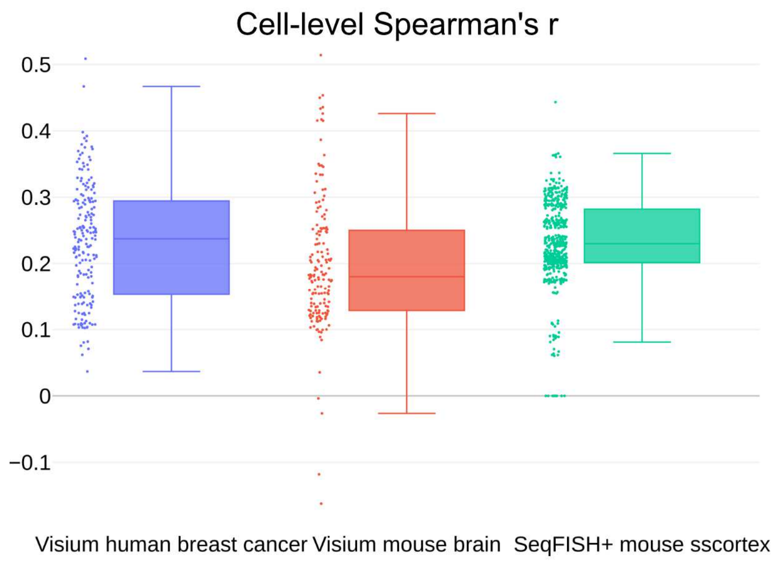

Supplementary Figure 21

Cell-level correlation between inferred signaling and expression of known downstream genes

Each dot represents a ligand-receptor pair. The Spearman's correlation coefficient is computed between the amount of received signal and the average expression of known downstream genes. The box plots show Q1 (25% quantile), Q2 (median), and Q3 (75% quantile). The upper whisker is the largest data point smaller than Q3+1.5IQR and the lower whisker is the smallest data point greater than Q1-1.5IQR. From left to right, n=192, 164, and 519 ligand-receptor pairs examined, respectively.

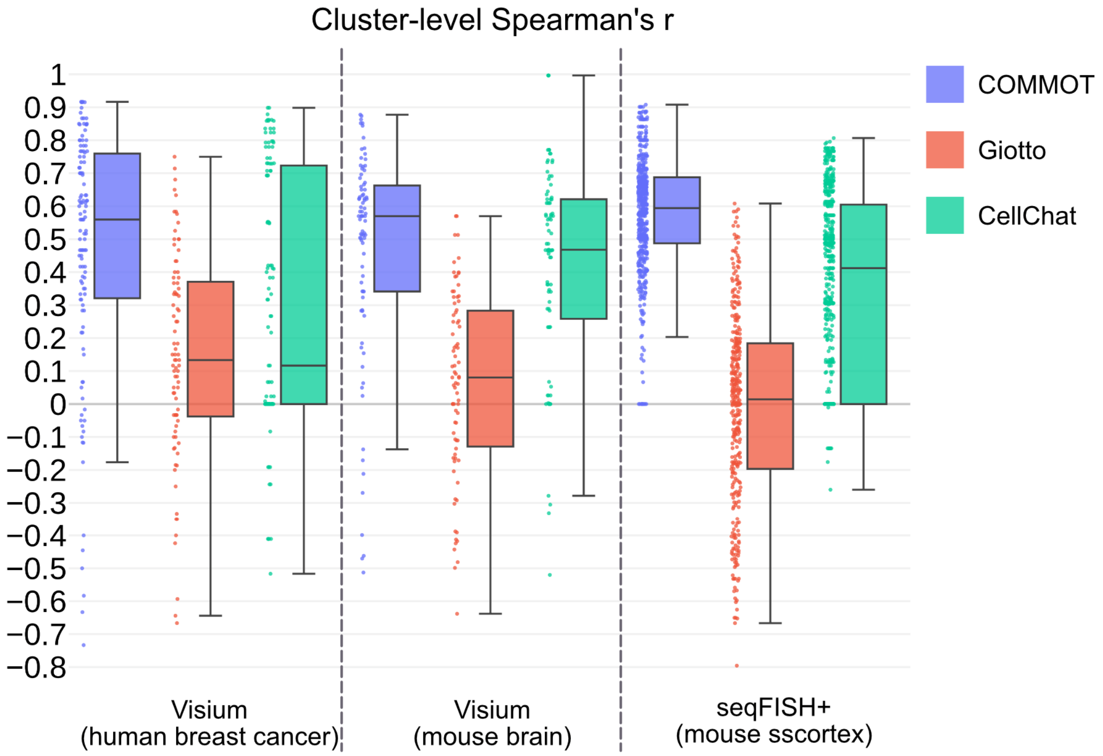

Supplementary Figure 22

Cluster-level correlation between inferred signaling and activity of known downstream genes (comparison with CellChat and Giotto)

Each dot represents a ligand-receptor pair. The activity of known downstream genes of a ligand-receptor pair is quantified for each cell cluster as the percentage of positive significant differentially expressed ones (p-value < 0.05) in that cluster. Spearman's correlation coefficients are computed between the amount of received signal and the downstream gene activity. For COMMOT, the amount of received signal is quantified by the average received signal of the cells in that cluster. For Giotto and CellChat which output a cluster-level signaling table, the maximum CCC score with each cluster as the receiver is used to quantify the amount of received signal. The box plots show Q1 (25% quantile), Q2 (median), and Q3 (75% quantile). The upper whisker is the largest data point smaller than Q3+1.5IQR and the lower whisker is the smallest data point greater than Q1-1.5IQR. For COMMOT and CellChat results, n=119, 84, and 518 ligand-receptor pairs for the Visium human breast cancer, Visium mouse brain, and seqFISH+ datasets, respectively. For Giotto, the number of pairs are n=85, 79, and 339, respectively. The heteromeric ligand and receptors are converted to single unit pairs for Giotto and thus the number of pairs is different from the numbers in COMMOT and CellChat.

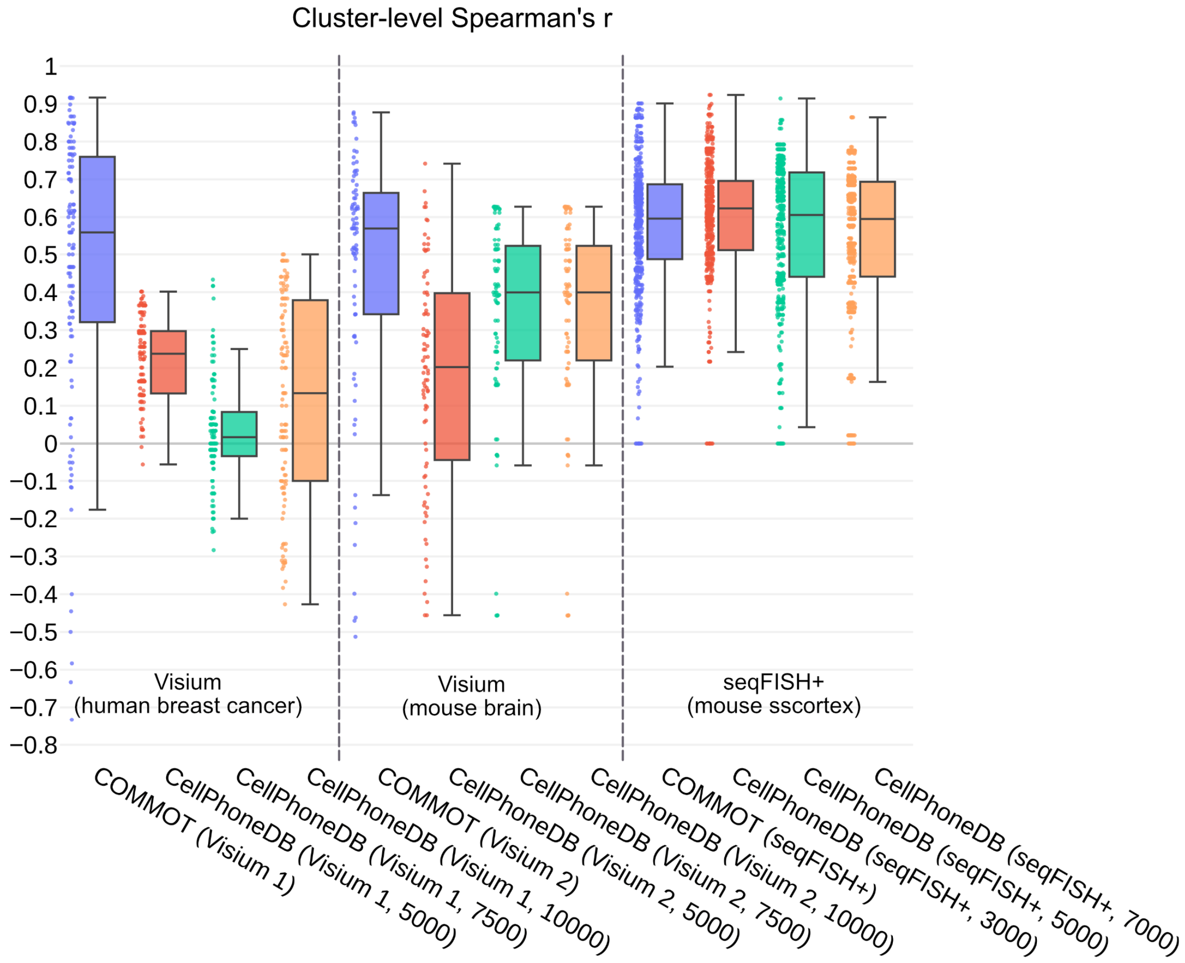

Supplementary Figure 23

Cluster-level correlation between inferred signaling and activity of known downstream genes (comparison with CellPhoneDB v3)

Each dot represents a ligand-receptor pair. The activity of known downstream genes of a ligand-receptor pair is quantified for each cell cluster as the percentage of positive significant differentially expressed ones (p-value < 0.05) in that cluster. Spearman's correlation coefficients are computed between the amount of received signal and the downstream gene activity. For COMMOT, the amount of received signal is quantified by the average received signal of the cells in that cluster. For CellPhoneDB, the total CCC score with each cluster as the receiver is used to quantify the amount of received signal. For COMMOT, a uniform 1000 μm (spot/cell level) distance cutoff is applied. For CellPhoneDB, the distance between clusters is first quantified as the average distance between spots/cells of the two clusters, and different cutoffs are tried (5000 μm, 7500 μm, and 10000 μm for Visium datasets and 3000 μm, 5000 μm, and 7000 μm for the seqFISH+ data). The box plots show Q1 (25% quantile), Q2 (median), and Q3 (75% quantile). The upper whisker is the largest data point smaller than Q3+1.5IQR and the lower whisker is the smallest data point greater than Q1-1.5IQR. For the Visium human breast cancer, Visium mouse brain, and seqFISH+ datasets, n=119, 83, and 499 ligand-receptor pairs, respectively.

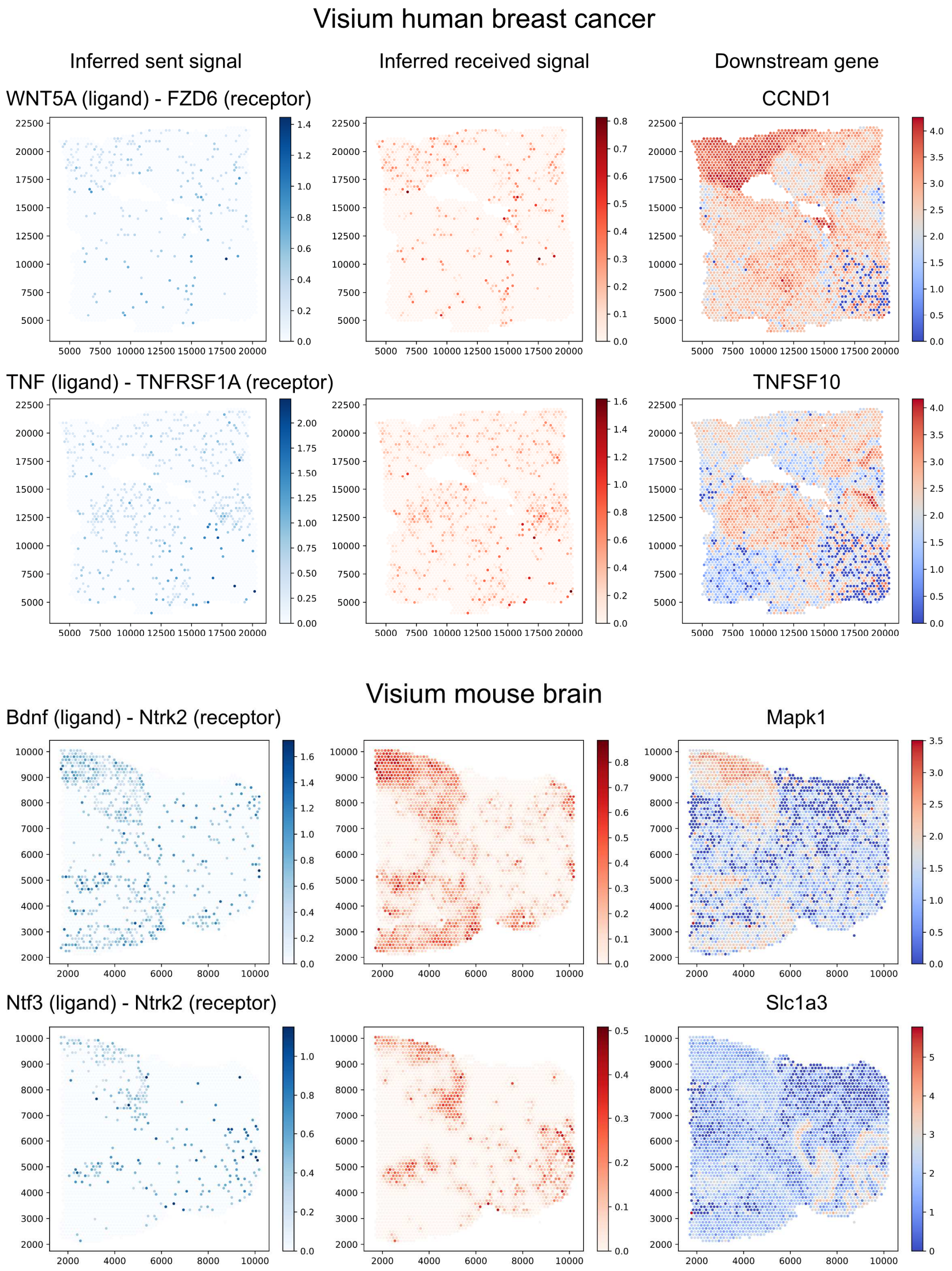

Supplementary Figure 24

Examples of inferred signaling and expression of known downstream genes.

The left column shows the ligands contributing to the inferred ligand-receptor complex. The middle column shows the inferred ligand-receptor complex. The right column shows the expression levels of known downstream target genes of the ligand-receptor pairs according to scSeqComm database.

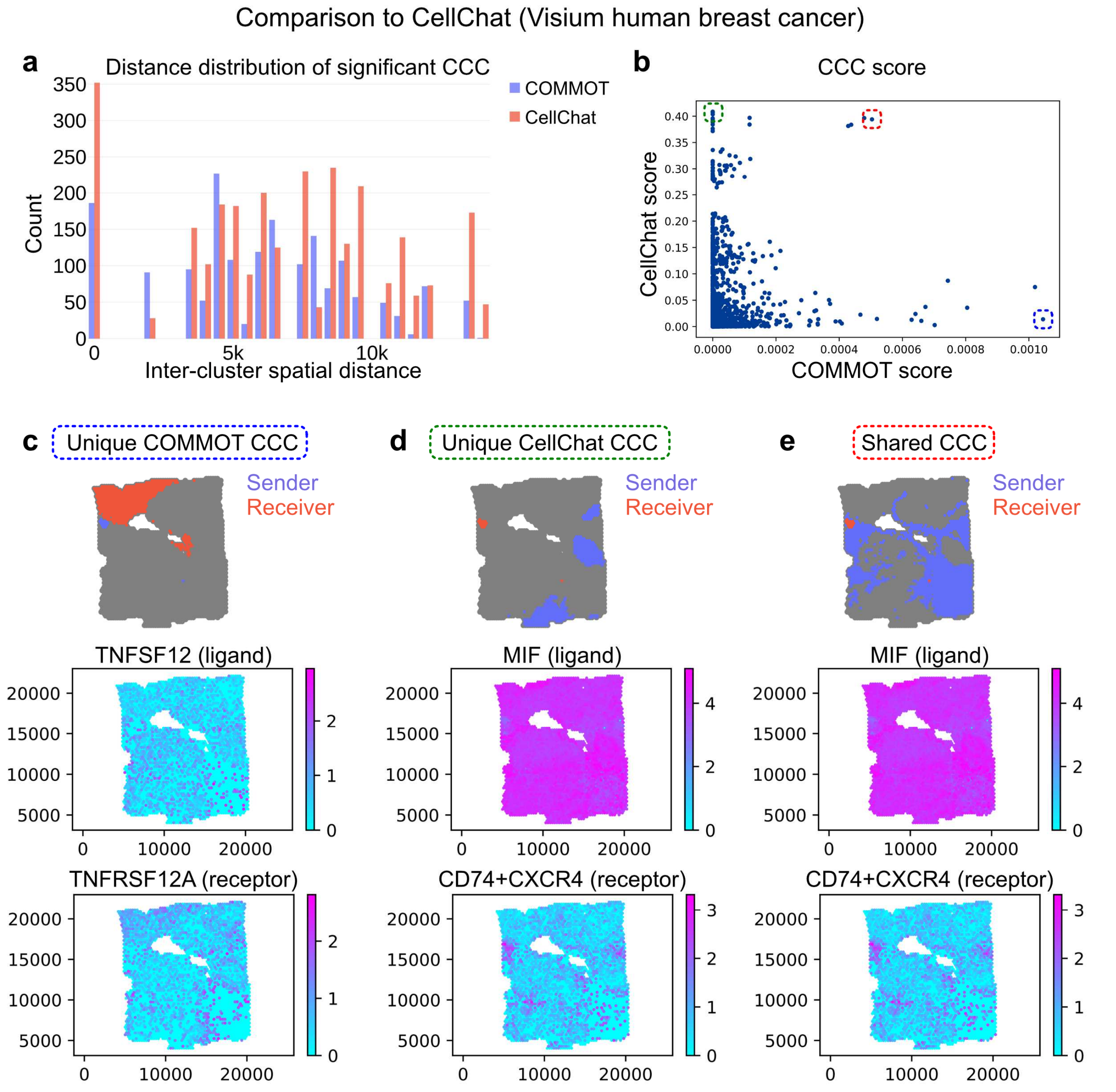

Supplementary Figure 25

Comparison with CellChat on a Visium human breast cancer dataset

a The distribution of inter-cluster distances (distance between geometric center of the clusters) of the significant CCC cluster pairs. **b** Each point represent a ligand-receptor pair and a cluster pair. **c-e** Example significant cluster-level CCC that are uniquely identified by COMMOT and CellChat, and by both methods. In comparison to CellChat, COMMOT uniquely detects CCC that are not differentially expressed in clusters but spatially significant.

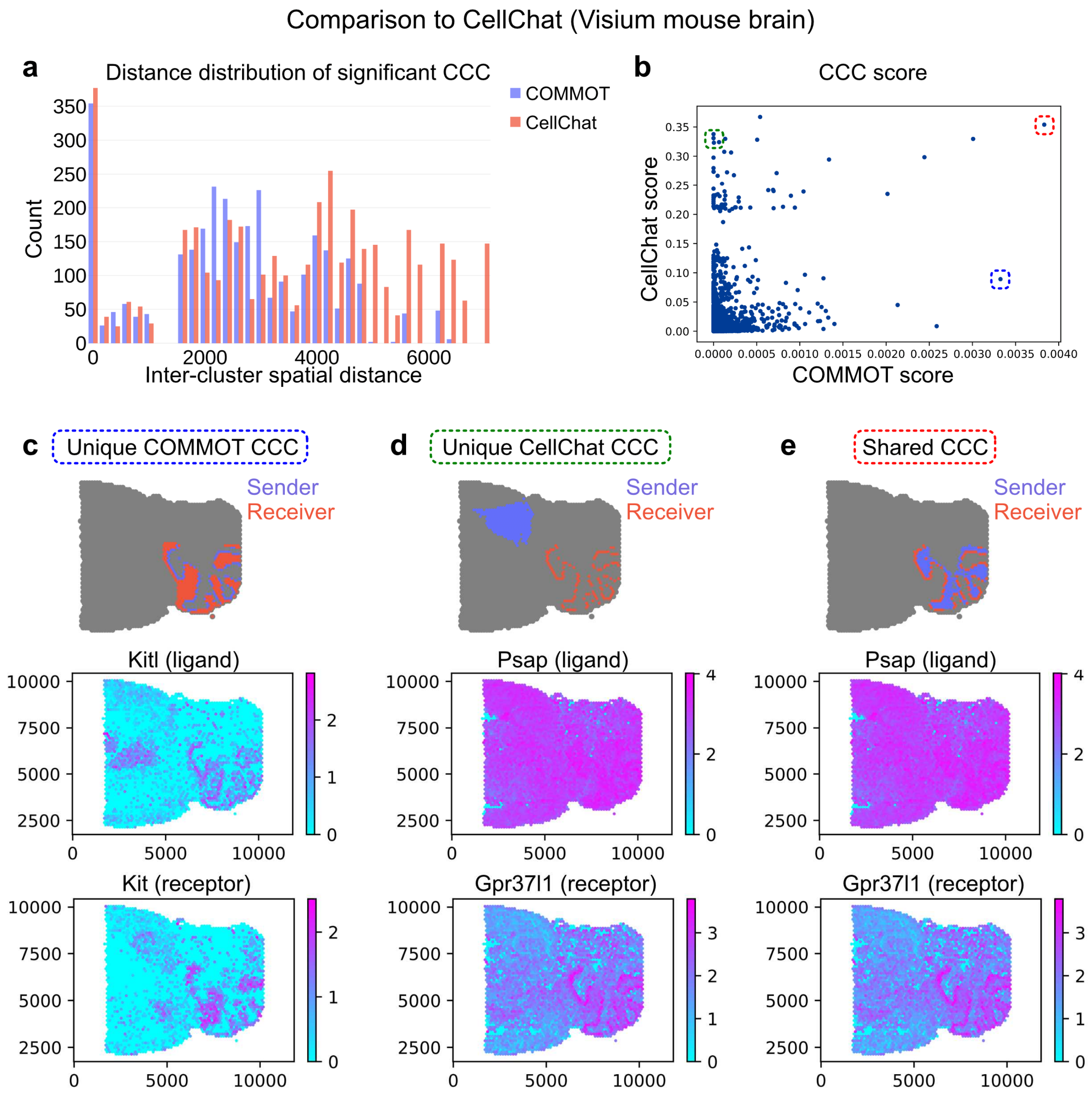

Supplementary Figure 26

Comparison with CellChat on a Visium mouse brain dataset

a The distribution of inter-cluster distances (distance between geometric center of the clusters) of the significant CCC cluster pairs. **b** Each point represent a ligand-receptor pair and a cluster pair. **c-e** Example significant cluster-level CCC that are uniquely identified by COMMOT and CellChat, and by both methods. In comparison to CellChat, COMMOT uniquely detects CCC that are not differentially expressed in clusters but spatially significant.

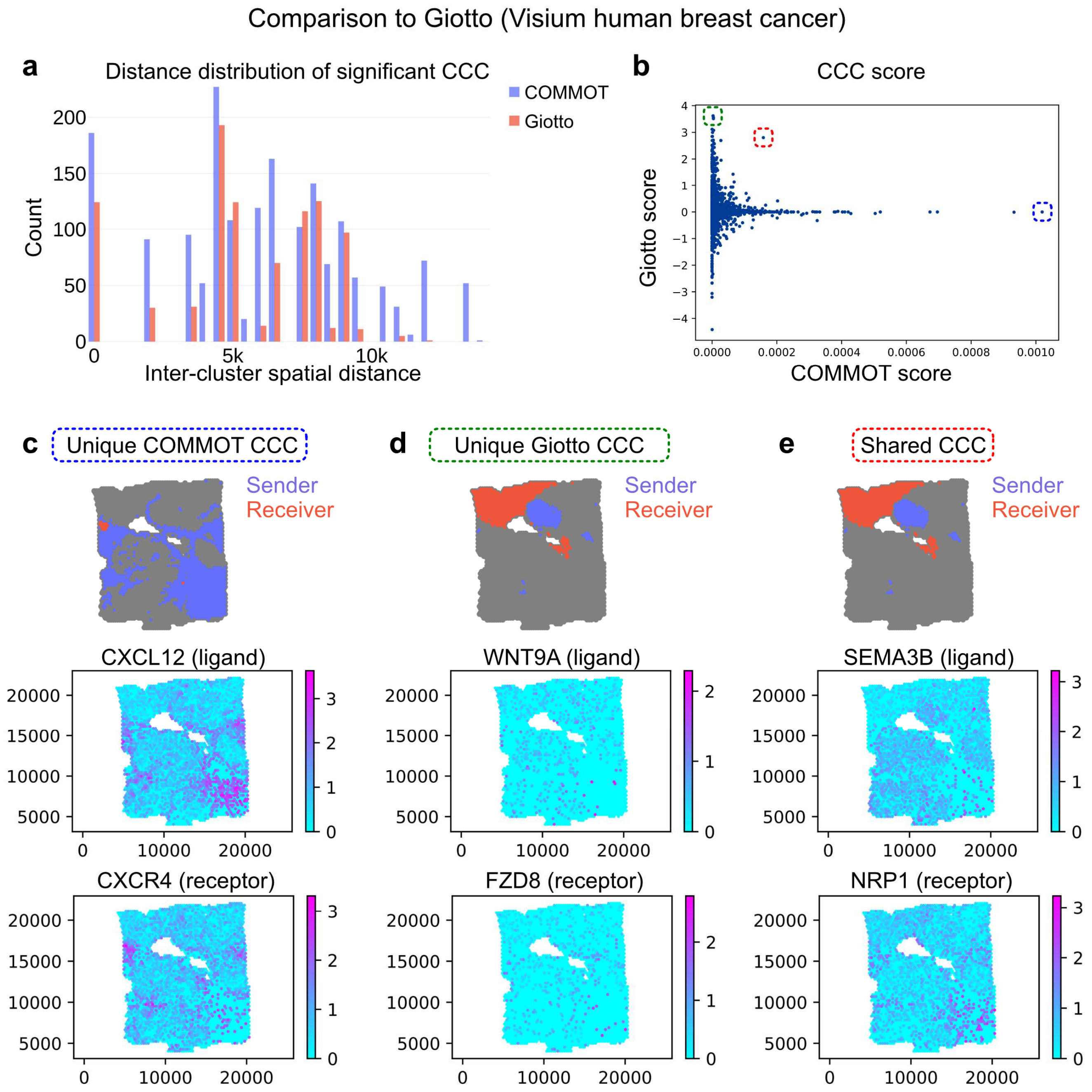

Supplementary Figure 27

Comparison with Giotto on a Visium human breast cancer dataset

a The distribution of inter-cluster distances (distance between geometric center of the clusters) of the significant CCC cluster pairs. **b** Each point represent a ligand-receptor pair and a cluster pair. **c-e** Example significant cluster-level CCC that are uniquely identified by COMMOT and Giotto, and by both methods. Both COMMOT and Giotto prioritize CCC between spatially close clusters while COMMOT identifies more CCC links that are beyond the neighboring cells because Giotto uses cell-contact graph that might limit the range of inferred CCC. Giotto characterizes the average expression of ligands and receptors of spatially neighboring spots without providing the role for each spot in CCC. COMMOT identifies each spot as a sender, receiver or both in CCC with interpretable results.

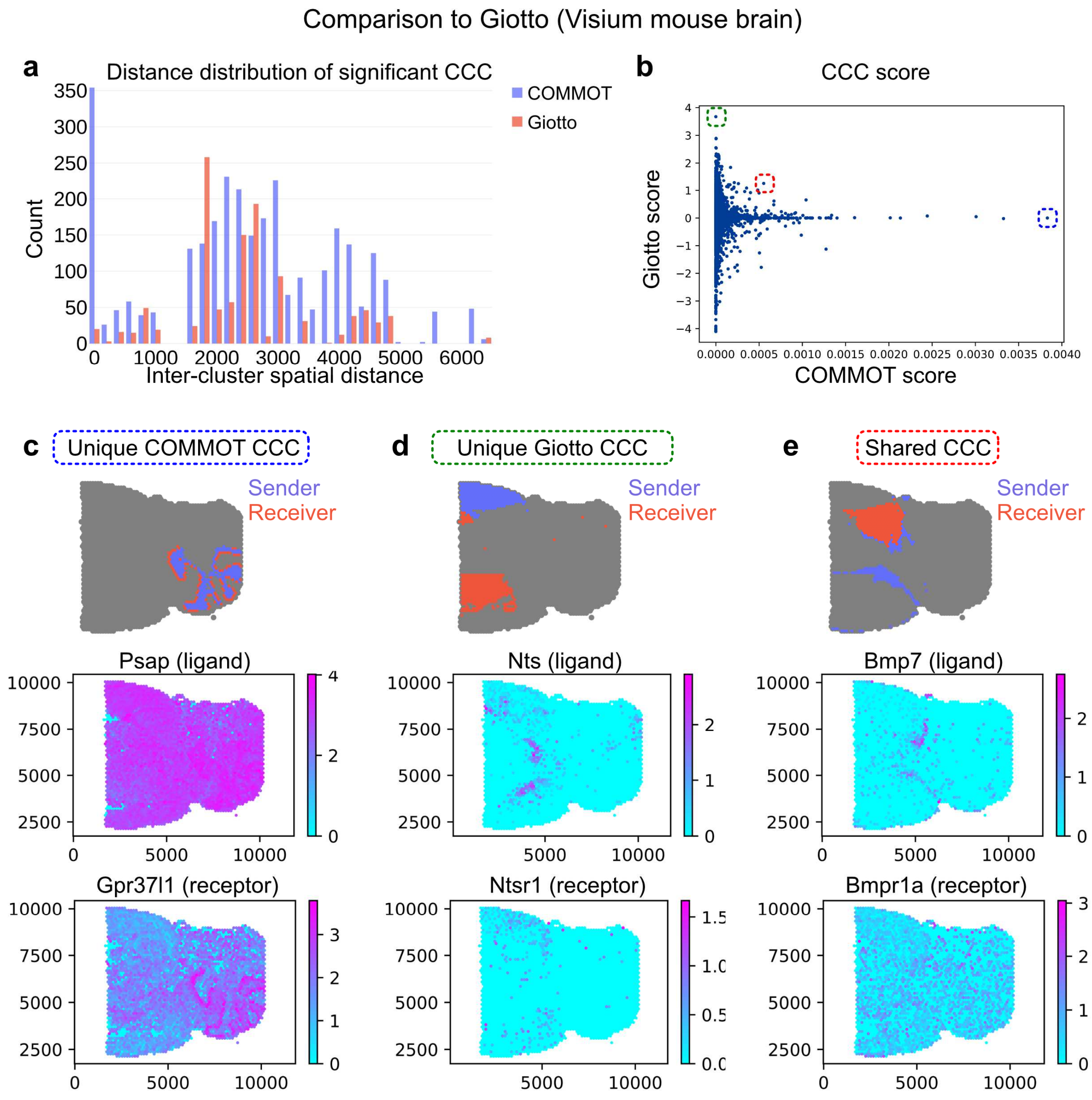

Supplementary Figure 28

Comparison with Giotto on a Visium mouse brain dataset

a The distribution of inter-cluster distances (distance between geometric center of the clusters) of the significant CCC cluster pairs. **b** Each point represent a ligand-receptor pair and a cluster pair. **c-e** Example significant cluster-level CCC that are uniquely identified by COMMOT and Giotto, and by both methods. Both COMMOT and Giotto prioritize CCC between spatially close clusters while COMMOT identifies more CCC links that are beyond the neighboring cells because Giotto uses cell-contact graph that might limit the range of inferred CCC. Giotto characterizes the average expression of ligands and receptors of spatially neighboring spots without providing the role for each spot in CCC. COMMOT identifies each spot as a sender, receiver or both in CCC with interpretable results.

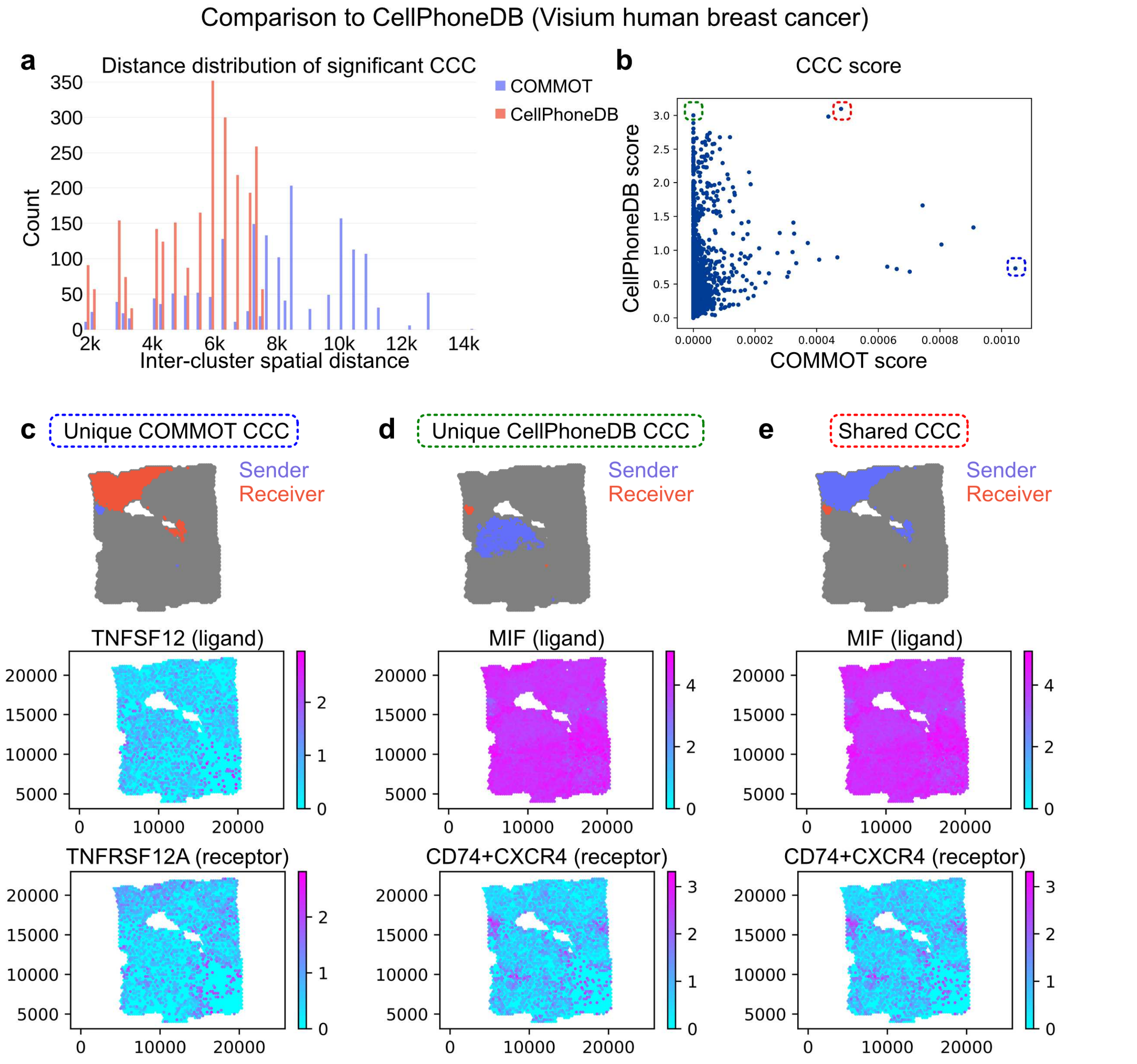

Supplementary Figure 29

Comparison with CellPhoneDB v3 on a Visium human breast cancer dataset

a The distribution of inter-cluster distances (distance between geometric center of the clusters) of the significant CCC cluster pairs. **b** Each point represent a ligand-receptor pair and a cluster pair. **c-e** Example significant cluster-level CCC that are uniquely identified by COMMOT and CellPhoneDB, and by both methods. CellPhoneDB v3 identifies more CCC links than COMMOT potentially due to its consideration of inter-cluster distances rather than distance among individual cells. COMMOT identifies CCC that is locally significant which CellPhoneDB v3 may neglect due to its consideration of expression level of entire clusters.

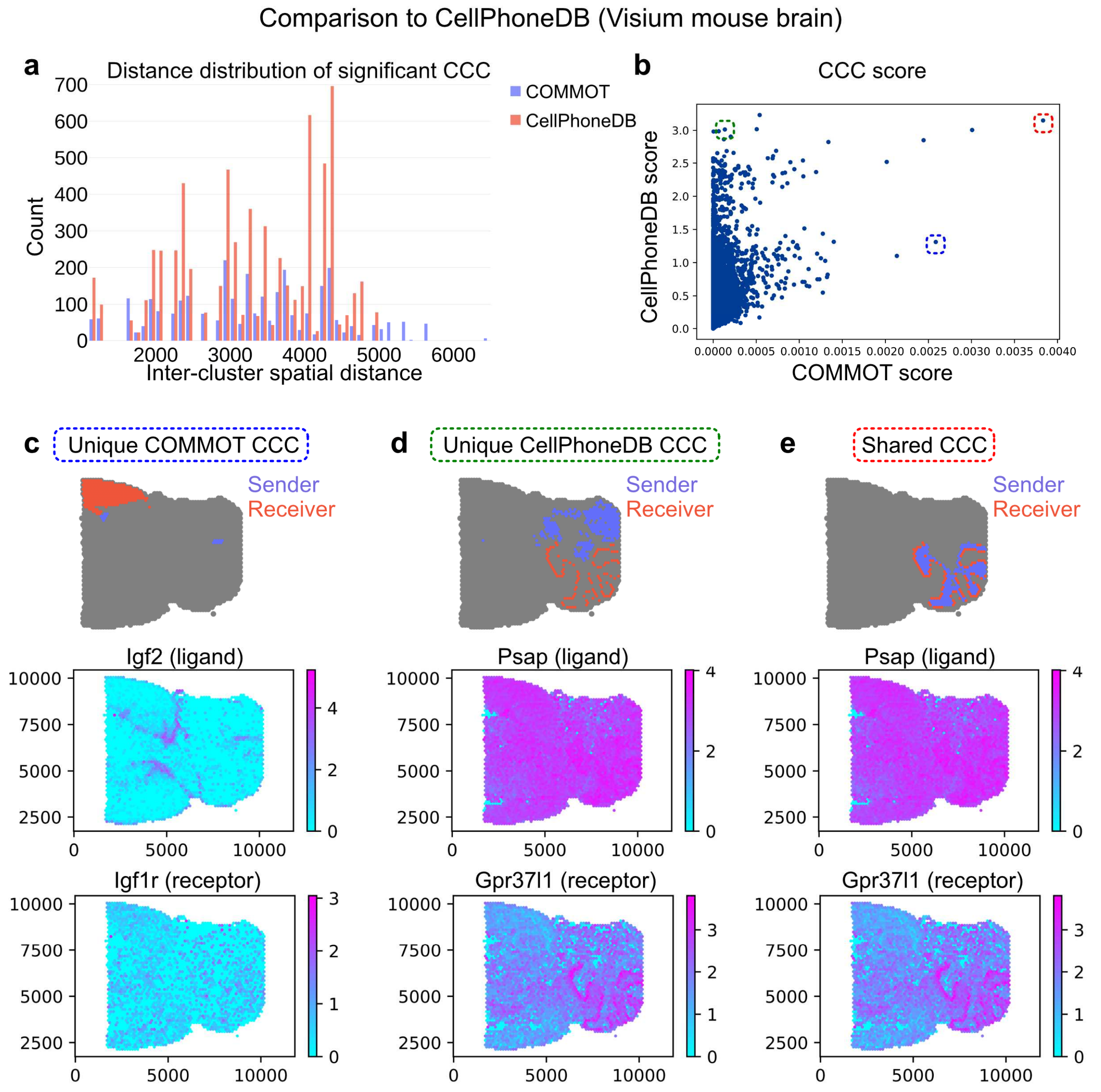

Supplementary Figure 30

Comparison with CellPhoneDB v3 on a Visium mouse brain dataset

a The distribution of inter-cluster distances (distance between geometric center of the clusters) of the significant CCC cluster pairs. **b** Each point represent a ligand-receptor pair and a cluster pair. **c-e** Example significant cluster-level CCC that are uniquely identified by COMMOT and CellPhoneDB, and by both methods. CellPhoneDB v3 identifies more CCC links than COMMOT potentially due to its consideration of inter-cluster distances rather than distance among individual cells. COMMOT identifies CCC that is locally significant which CellPhoneDB v3 may neglect due to its consideration of expression level of entire clusters.

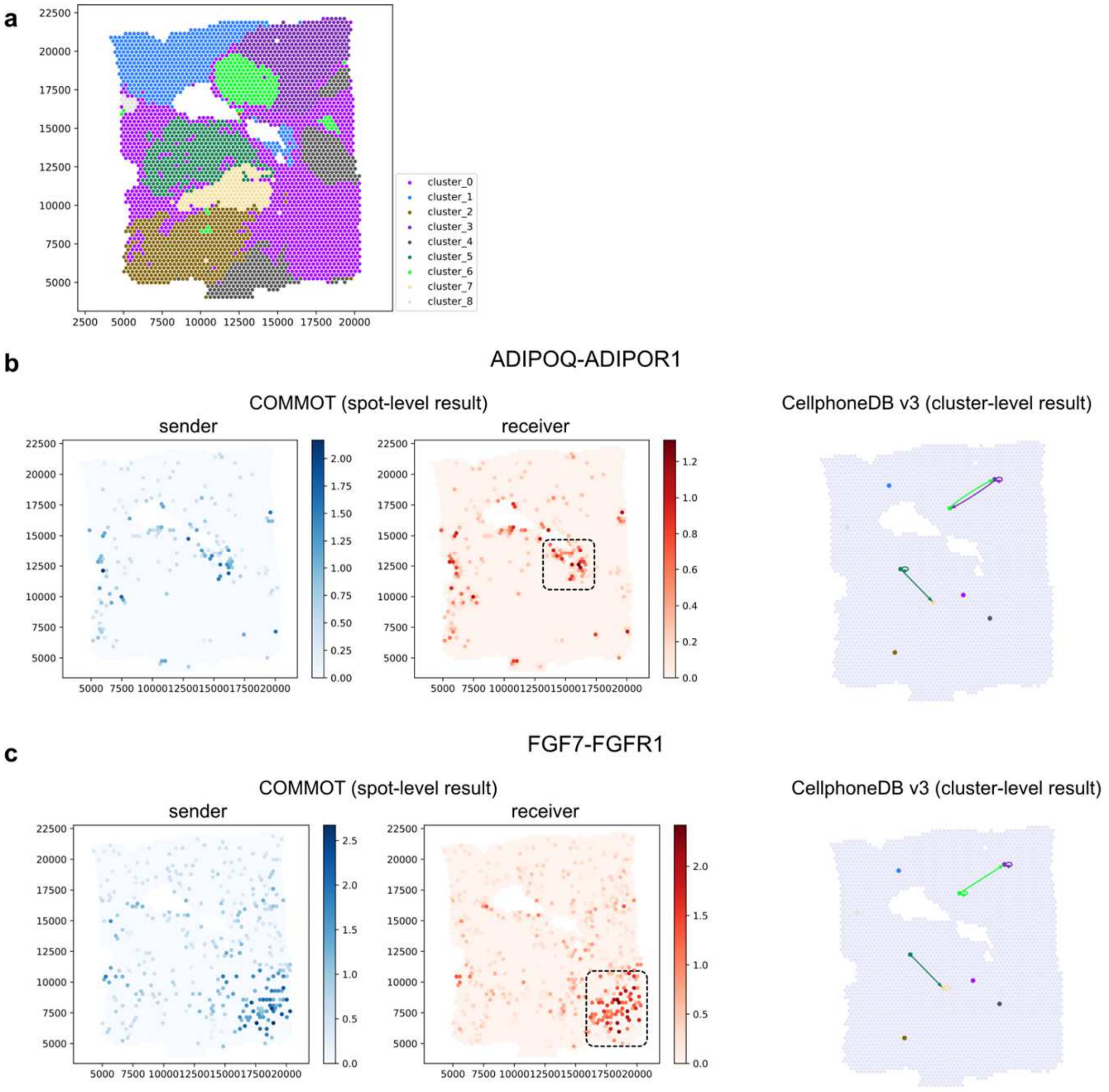

Supplementary Figure 31

Comparison of spot-level and cluster-level approaches on a Visium human breast cancer dataset

a Clustering of the spatial data. **b-c** Left two columns: The level of sent and received signals of the spots inferred by COMMOT. Right: The significant ($p \leq 0.05$) cluster-level signaling connections inferred by CellPhoneDB v3 with a cluster-level distance cutoff of 5000 μm. For the LR pairs ADIPOQ-ADIPOR1 and FGF7-FGFR1, COMMOT found local signaling hotspots (marked by dashed boxes) as subclusters of clusters 1 and 0, respectively, which were overlooked by the cluster-level inference.

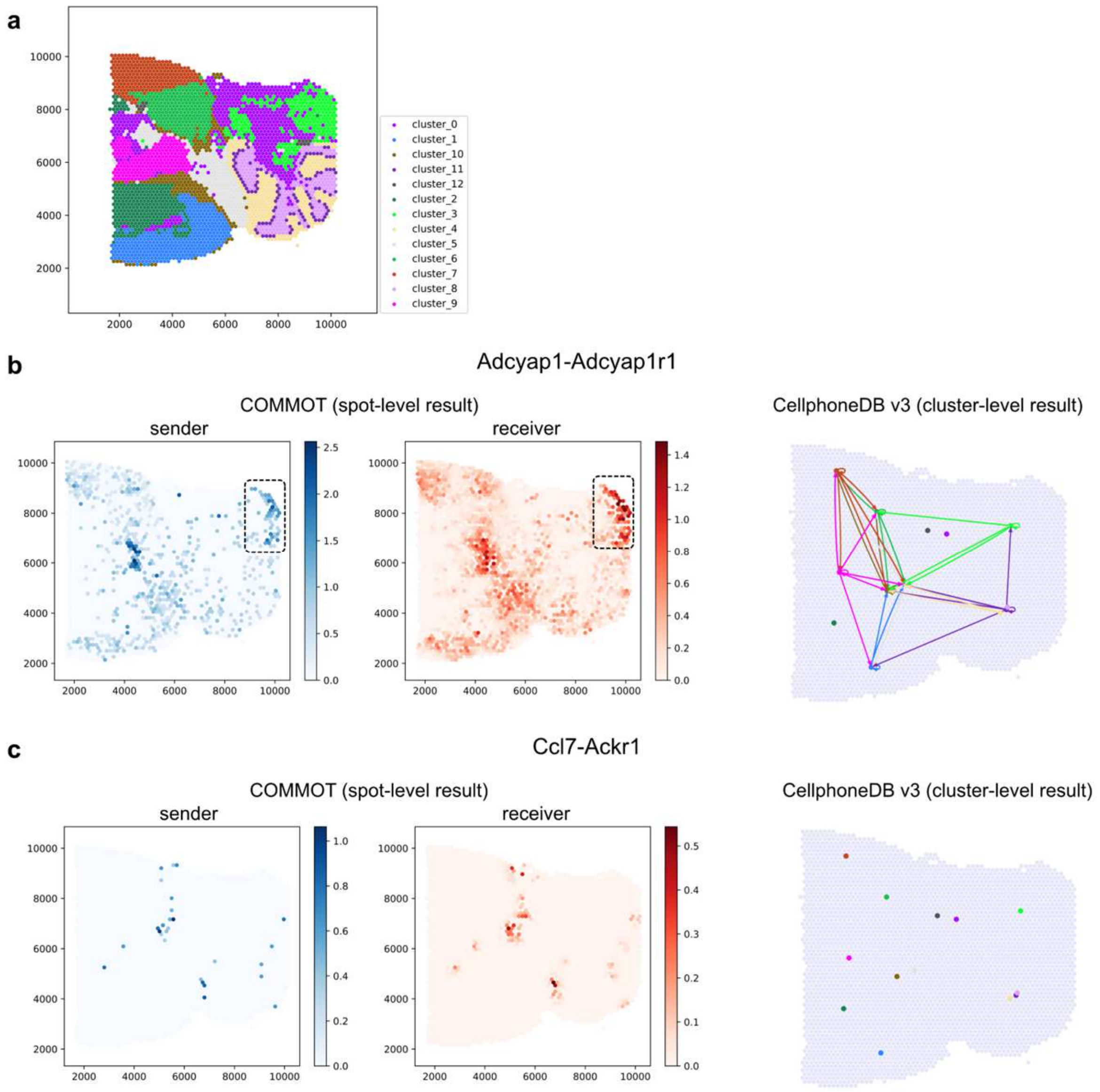

Supplementary Figure 32

Comparison of spot-level and cluster-level approaches on a Visium mouse brain dataset

a Clustering of the spatial data. **b-c** Left two columns: The level of sent and received signals of the spots inferred by COMMOT. Right: The significant ($p \leq 0.05$) cluster-level signaling connections inferred by CellPhoneDB v3 with a cluster-level distance cutoff of 5000 µm. For the LR pair Adcyap1-Adcyapr1, COMMOT found a localized signaling hotspot within cluster 3 which is far away from other significant sender and receiver populations while the cluster-level method still identified significant signaling connections between cluster 3 and others. For the LR pair FGF7-FGFR1, COMMOT found local signaling hotspots which were overlooked by the cluster-level inference.

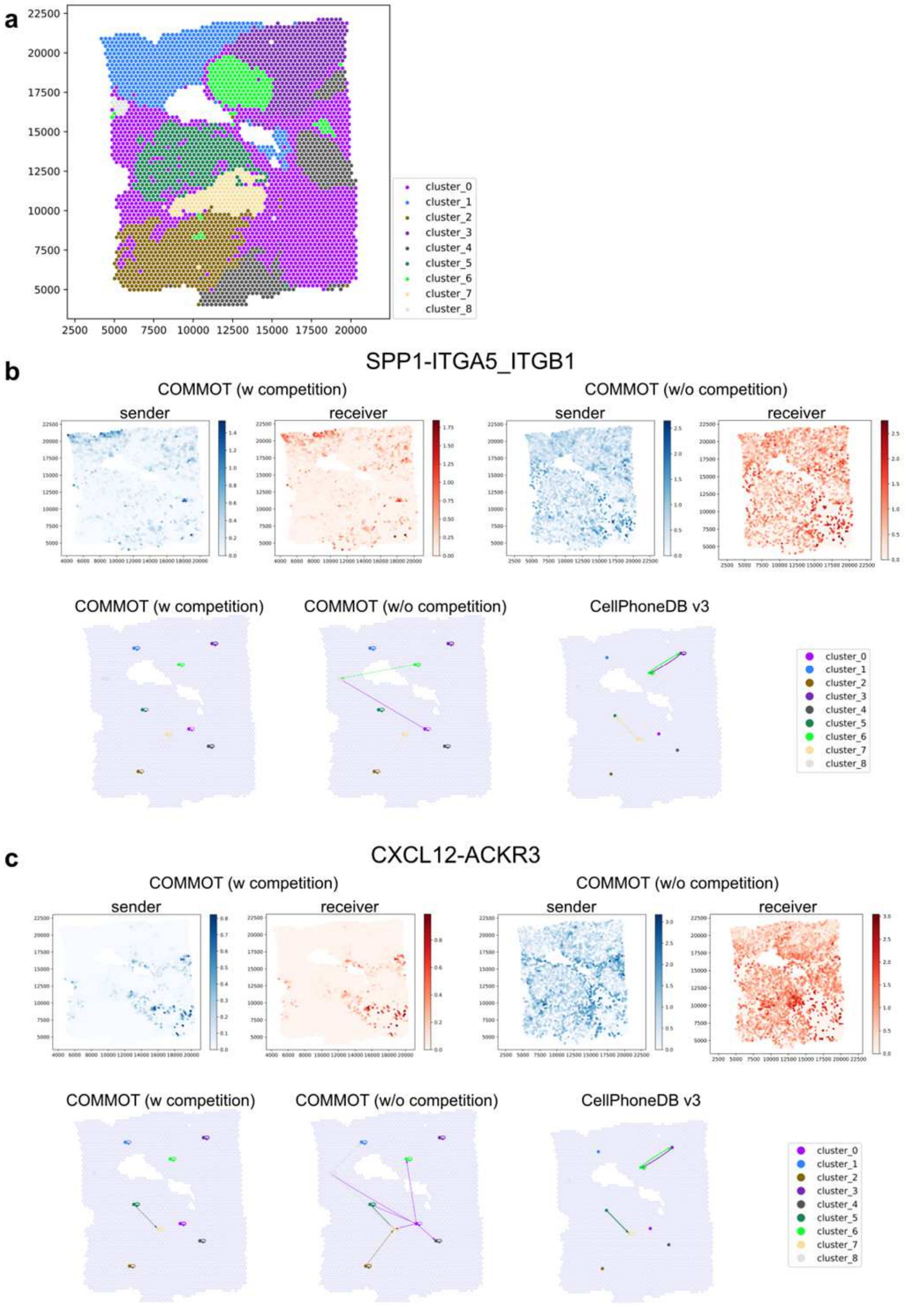

Supplementary Figure 33

Illustration of effect of competition on a Visium human breast cancer dataset

a Clustering of the spatial data. **b-c** Top: The level of sent and received signals of the spots inferred by COMMOT with or without considering competition between ligand and receptor species. Bottom: The significant ($p \leq 0.05$) cluster-level signaling connections inferred by COMMOT with or without considering competition and by CellPhoneDB v3 with a cluster-level distance cutoff of 5000 µm. For the LR pairs SPP1-ITGA5-ITGB1 and CXCL12-ACKR3, COMMOT with competition found localized signaling hotspots compared to the other two approaches.

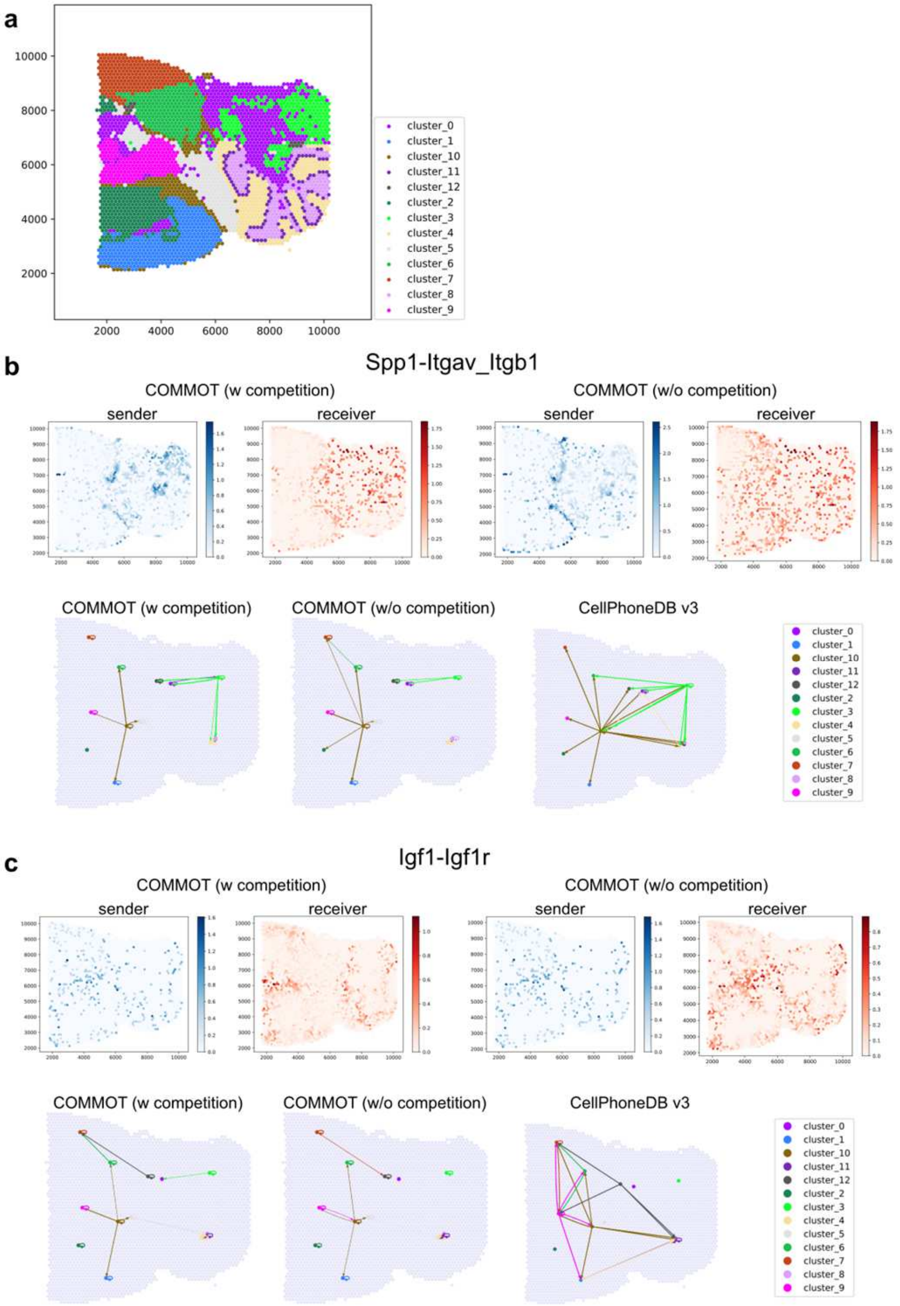

Supplementary Figure 34

Illustration of effect of competition on a Visium mouse brain dataset

a Clustering of the spatial data. **b-c** Top: The level of sent and received signals of the spots inferred by COMMOT with or without considering competition between ligand and receptor species. Bottom: The significant ($p \leq 0.05$) cluster-level signaling connections inferred by COMMOT with or without considering competition and by CellPhoneDB v3 with a cluster-level distance cutoff of 5000 µm. For the LR pair Spp1-Itgav_Itgb1, COMMOT with considering competition found higher signaling activity on the right side of the tissue while the other approaches inferred active signaling everywhere. For the LR pair Igf1-Igf1r, COMMOT with considering competition also found localized signaling hotspots compared to other approaches.

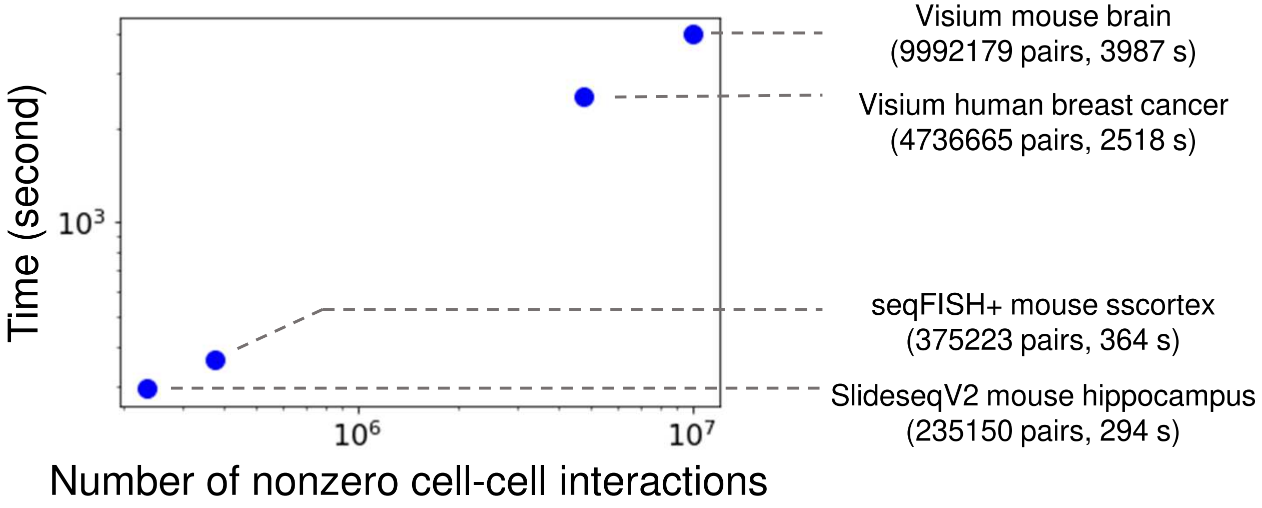

Supplementary Figure 35

CCC inference running time of COMMOT

The running time (tested on a desktop with i7-8700K running Ubuntu 20.04) of the collective optimal transport algorithm of COMMOT (the core CCC inference function) on several datasets. The wall time of computation is plotted against the number of nonzero elements in the interaction networks. Both axes are log transformed.

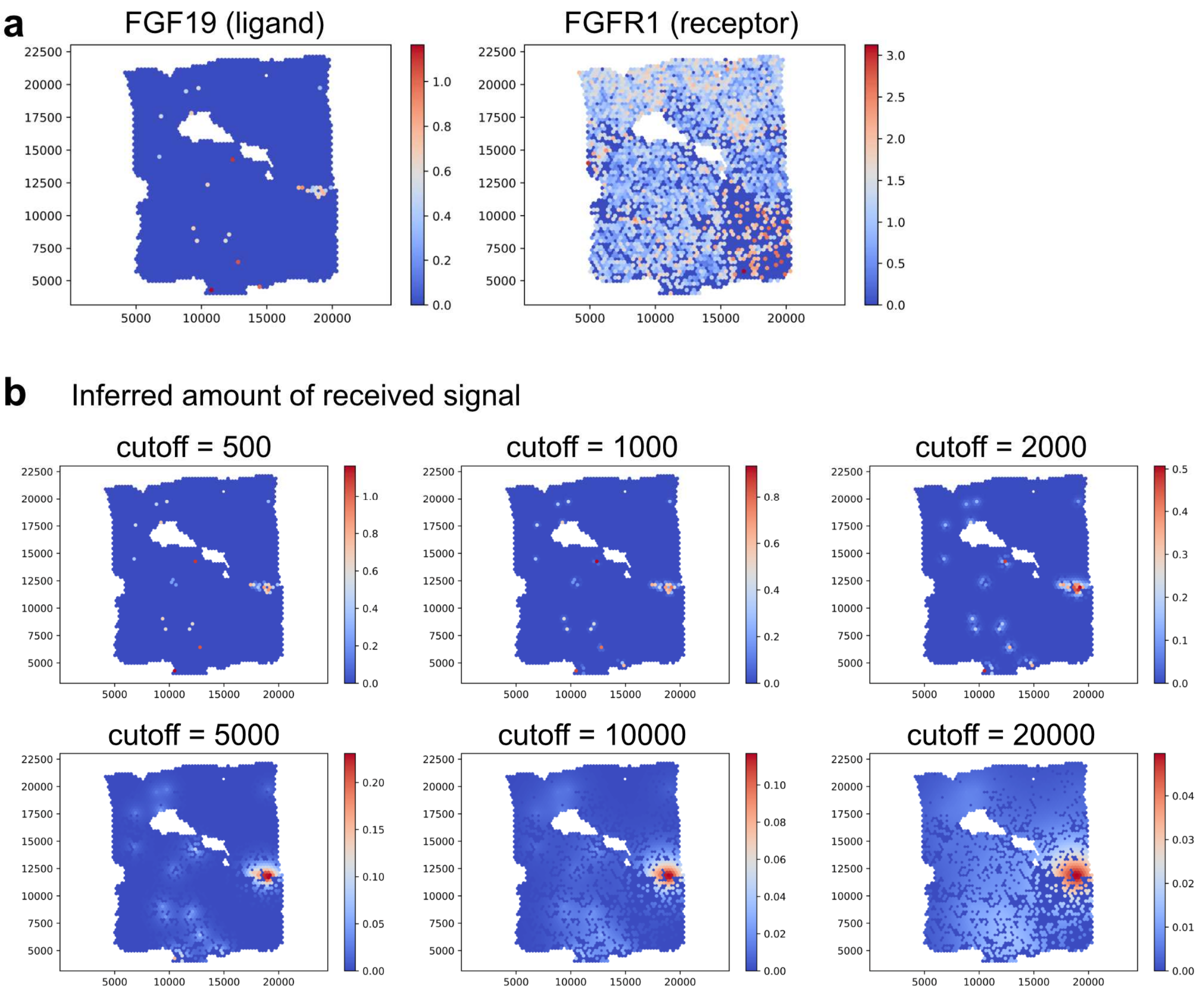

Supplementary Figure 36

Illustration of the effect of different distance cutoffs

An example ligand-receptor pair (FGF19-FGFR1) and the Visium data of human breast cancer is used to demonstrate the effect of using different distance cutoffs in COMMOT. **a** The spatial expression of FGF19 and FGFR1. **b** The inferred amount of received signal (spatial distribution of FGF19-FGFR1 complexes with different distance cutoffs in the unit of microns.

Supplementary Note

Contents

1 Collective optimal transport in COMMOT **1**
1.1 Collective optimal transport 1
1.2 Alternative formulation of COT 2
1.3 Numerical solutions 3

2 Benchmark and comparison with PDE-based simulation **5**
2.1 Model 6
2.2 Synthetic data generation 6
2.3 Benchmark and comparison result 7

3 Joint analysis of mouse cortex with different datasets **8**

4 Robustness evaluation of COMMOT on drosophila embryo data **8**

5 Comparison to CellChat, Giotto, and CellphoneDB **9**

1 Collective optimal transport in COMMOT

1.1 Collective optimal transport

Motivated by the biological application of analyzing interactions among multiple species of ligand and receptors, we introduce collective optimal transport (COT) which considers multiple source and target species, unlike traditional optimal transport [1, 2] that only couples two single species. Consider n_s points (cells of spots of cells) on which the discrete distributions are supported. Collective optimal transport couples n_l source species (ligands in the COMMOT application), $\boldsymbol{\alpha}_i \in \mathbb{R}_+^{n_s}, i = 1, \cdots, n_l$ and n_r target species (receptors in the COMMOT application), $\boldsymbol{\beta}_j \in \mathbb{R}_+^{n_s}, j = 1, \cdots, n_r$. Among these species, only part of the species pairs can interact and we use I to denote the indexes of iteracting species such that $(i,j) \in I$ indicates that source species i interacts with target species j. For each species pair $(i,j) \in I$, a precomputed cost matrix $\mathbf{C}_{(i,j)} \in \mathbb{R}_+^{n_s \times n_s}$ is given. An example of the cost matrix is the Euclidean distance between the n_s points where a distance entry is replaced by ∞ if it exceeds the spatial range of signaling. Collective optimal transport finds a collection of coupling plans $\mathbf{P}_{(i,j)} \in \mathbb{R}_+^{n_s \times n_s}, (i,j) \in I$ for each species pair in I that minimizes the total coupling cost over all pair.

The following optimization problem is formulated:

$$\begin{aligned}
&\min_{\{\mathbf{P}_{(i,j)}\}\in\mathbf{\Gamma}} \sum_{(i,j)\in I} \langle \mathbf{P}_{(i,j)}, \mathbf{C}_{(i,j)}\rangle_{\mathsf{F}} + \sum_i F(\boldsymbol{\mu}_i) + \sum_j F(\boldsymbol{\nu}_j),\\
&\mathbf{\Gamma} = \left\{ \{\mathbf{P}_{(i,j)}\} : \sum_{j \text{ if } (i,j)\in I} \sum_l \mathbf{P}_{(i,j)}(k,l) \le \boldsymbol{\alpha}_i(k), \sum_{i \text{ if } (i,j)\in I} \sum_k \mathbf{P}_{(i,j)}(k,l) \le \boldsymbol{\beta}_j(l), \mathbf{P}_{(i,j)}(k,l) \ge 0 \right\} \\
&\boldsymbol{\mu}_i(k) = \boldsymbol{\alpha}_i(k) - \sum_{j \text{ if } (i,j)\in I} \sum_l \mathbf{P}_{(i,j)}(k,l), \boldsymbol{\nu}_j(l) = \boldsymbol{\beta}_j(l) - \sum_{i \text{ if } (i,j)\in I} \sum_k \mathbf{P}_{(i,j)}(k,l),
\end{aligned} \tag{1}$$

where F penalizes the uncoupled masses $\boldsymbol{\mu}_i$ and $\boldsymbol{\nu}_j$.

In practice, there could be infinity entries in the cost matrices to block certain coupling according to specific interpretations in the applied domain. Also, $\boldsymbol{\alpha}_i$ and $\boldsymbol{\beta}_j$ are not required to be probability distributions. This property enables the application of collective optimal transport to problems where the mass distribution should not be normalized, for example, when the units need to be preserved. Here, the units are the expression levels of various ligand and receptor species and they need not to be normalized individually so that they stay comparable.

1.2 Alternative formulation of COT

To solve the collective optimal transport problem described above, we reformulate the problem. The multiple source and target species are combined to formulate an optimal transport problem of size $(n_s{*}n_l)\times(n_s{*}n_r)$. A new pair of distributions $\boldsymbol{\alpha} \in \mathbb{R}^{n_s * n_l}$ and $\boldsymbol{\beta} \in \mathbb{R}^{n_s * n_r}$ to be coupled is generated such that $\boldsymbol{\alpha}(i * n_s + k) = \boldsymbol{\alpha}_i(k)$ and $\boldsymbol{\beta}(j * n_s + l) = \boldsymbol{\beta}_j(l)$ The corresponding new cost matrix is then defined as

$$\mathbf{C}(i * n_l + 1 : (i+1) * n_l, j * n_l + 1 : (j+1) * n_l) = \begin{cases} \mathbf{C}_{(i,j)}, \text{ if } (i,j) \in I \\ \infty, \text{ otherwise} \end{cases} \tag{2}$$

In this setup, the discrete OT problem is formulated as the following,

$$\begin{aligned}
\min_{\mathbf{P},\boldsymbol{\mu},\boldsymbol{\nu}} \quad & \langle \mathbf{P}, \mathbf{C}\rangle_{\mathrm{F}} + F(\boldsymbol{\mu}) + G(\boldsymbol{\nu}) \\
\text{s.t.} \quad & \mathbf{P} \in \mathbb{R}_+^{m\times n}, \boldsymbol{\mu} \in \mathbb{R}_+^m, \boldsymbol{\nu} \in \mathbb{R}_+^n \\
& \mathbf{P}\mathbf{1}^n = \boldsymbol{\alpha} - \boldsymbol{\mu}, \\
& \mathbf{P}^T\mathbf{1}^m = \boldsymbol{\beta} - \boldsymbol{\nu},
\end{aligned} \tag{3}$$

where $m = n_s * n_l$, $n = n_s * n_r$, $\boldsymbol{\mu}$ and $\boldsymbol{\nu}$ are the unpaired masses, and F and G are penalty functions. In this work, we use two specific constructions with l_1 or l_2 penalty for the untransported mass.

COT with l_1 penalty

$$\begin{aligned}
\min_{\mathbf{P},\boldsymbol{\mu},\boldsymbol{\nu}} \quad & \langle \mathbf{P}, \mathbf{C}\rangle_{\mathrm{F}} + \epsilon_p H(\mathbf{P}) + \epsilon_\mu H(\boldsymbol{\mu}) + \epsilon_\nu H(\boldsymbol{\nu}) + \rho(\|\boldsymbol{\mu}\|_1 + \|\boldsymbol{\nu}\|_1) \\
\text{s.t.} \quad & \mathbf{P}\mathbf{1}^n = \boldsymbol{\alpha} - \boldsymbol{\mu}, \\
& \mathbf{P}^T\mathbf{1}^m = \boldsymbol{\beta} - \boldsymbol{\nu}.
\end{aligned} \tag{4}$$

COT with l_2 penalty

$$\begin{aligned} \min_{\mathbf{P},\boldsymbol{\mu},\boldsymbol{\nu}} \quad & \langle \mathbf{P}, \mathbf{C} \rangle_{\mathrm{F}} + \epsilon_p H(\mathbf{P}) + \epsilon_\mu H(\boldsymbol{\mu}) + \epsilon_\nu H(\boldsymbol{\nu}) + \frac{\rho}{2}(\|\boldsymbol{\mu}\|_2^2 + \|\boldsymbol{\nu}\|_2^2) \\ \text{s.t.} \quad & \mathbf{P}\mathbf{1}^n = \boldsymbol{\alpha} - \boldsymbol{\mu}, \\ & \mathbf{P}^T\mathbf{1}^m = \boldsymbol{\beta} - \boldsymbol{\nu}. \end{aligned} \tag{5}$$

In the equations above, $H(\mathbf{x}) = \sum_i \mathbf{x}_i(\ln(\mathbf{x}_i) - 1)$ is an entropy regularization function which is widely used to increase the computational efficiency [3]. The nonnegativity constraints in Eq. (3) are enforced here by the entropy barriers in Eqs. (4) and (5). The entropy terms also enforce a strong convexity which enables efficient numerical solutions.

1.3 Numerical solutions

In this section, we derive two kinds of numerical solutions to the optimization problems in Eqs. (4) and (5).

COT with l_1 penalty We begin with the Lagrangian associated with Eq. (4),

$$\begin{aligned} \mathcal{E}(\mathbf{P}, \boldsymbol{\mu}, \boldsymbol{\nu}; \mathbf{f}, \mathbf{g}) =& \langle \mathbf{P}, \mathbf{C} \rangle_{\mathrm{F}} + \epsilon_p H(\mathbf{P}) + \epsilon_\mu H(\boldsymbol{\mu}) + \epsilon_\nu H(\boldsymbol{\nu}) + \rho(\|\boldsymbol{\mu}\|_1 + \|\boldsymbol{\nu}\|_1) \\ & - \langle \mathbf{f}, \mathbf{P}\mathbf{1}^n - (\boldsymbol{\alpha} - \boldsymbol{\mu}) \rangle - \langle \mathbf{g}, \mathbf{P}^T\mathbf{1}^m - (\boldsymbol{\beta} - \boldsymbol{\nu}) \rangle \end{aligned} \tag{6}$$

The first order condition relates $\mathbf{P}$ with the multipliers as

$$\frac{\partial \mathcal{E}}{\partial \mathbf{P}_{ij}} = \mathbf{C}_{ij} + \epsilon_p \log \mathbf{P}_{ij} - \mathbf{f}_i - \mathbf{g}_j = 0 \quad \Rightarrow \quad \mathbf{P} = e^{\frac{\mathbf{f} \oplus \mathbf{g} - \mathbf{C}}{\epsilon_p}}. \tag{7}$$

With $(\epsilon H(\cdot) + \rho \|\cdot\|_1)^*(y) = \epsilon e^{\frac{y-\rho}{\epsilon}}$ and condition in Eq. (7), we have the following Lagrange dual function,

$$\begin{aligned} L(\mathbf{f}, \mathbf{g}) =& \langle \mathbf{f}, \boldsymbol{\alpha} \rangle + \langle \mathbf{g}, \boldsymbol{\beta} \rangle - \epsilon_p \langle e^{\frac{\mathbf{f} \oplus \mathbf{g} - \mathbf{C}}{\epsilon_p}}, \mathbf{1}^{m \times n} \rangle_{\mathrm{F}} \\ & - \epsilon_\mu \langle e^{\frac{\mathbf{f} - \rho}{\epsilon_\mu}}, \mathbf{1}^{m \times n} \rangle_{\mathrm{F}} - \epsilon_\nu \langle e^{\frac{\mathbf{g} - \rho}{\epsilon_\nu}}, \mathbf{1}^{m \times n} \rangle_{\mathrm{F}}, \end{aligned} \tag{8}$$

whose first order derivatives are

$$\begin{aligned} \frac{\partial L}{\partial \mathbf{f}} &= \boldsymbol{\alpha} - e^{\frac{\mathbf{f}}{\epsilon_p}} \odot (e^{-\frac{\mathbf{C}}{\epsilon_p}} e^{\frac{\mathbf{g}}{\epsilon_p}}) - e^{\frac{\mathbf{f} - \rho}{\epsilon_\mu}}, \\ \frac{\partial L}{\partial \mathbf{g}} &= \boldsymbol{\beta} - e^{\frac{\mathbf{g}}{\epsilon_p}} \odot (e^{-\frac{\mathbf{C}^T}{\epsilon_p}} e^{\frac{\mathbf{f}}{\epsilon_p}}) - e^{\frac{\mathbf{g} - \rho}{\epsilon_\nu}}. \end{aligned} \tag{9}$$

These immediately leads to gradient based methods, such as momentum gradient ascent and Nesterov's method [4].

If we set $\epsilon = \epsilon_p = \epsilon_\mu = \epsilon_\nu$, we can easily obtain the explicit first order conditions,

$$\begin{aligned} \mathbf{f} &= \epsilon \log \boldsymbol{\alpha} - \epsilon \log(e^{-\frac{\mathbf{C}}{\epsilon}} e^{\frac{\mathbf{g}}{\epsilon}} + e^{-\frac{\rho}{\epsilon}}), \\ \mathbf{g} &= \epsilon \log \boldsymbol{\beta} - \epsilon \log(e^{-\frac{\mathbf{C}^T}{\epsilon}} e^{\frac{\mathbf{f}}{\epsilon}} + e^{-\frac{\rho}{\epsilon}}). \end{aligned} \tag{10}$$

We now apply the log-sum-exp trick to stabilize computation for small value of ϵ. Note that

$$\log(e^{-\frac{\mathbf{C}}{\epsilon}}e^{\frac{\mathbf{g}}{\epsilon}}+e^{-\frac{\rho}{\epsilon}}) = -\frac{\mathbf{a}}{\epsilon}+\log(e^{\frac{\mathbf{a}}{\epsilon}}\odot e^{-\frac{\mathbf{C}}{\epsilon}}e^{\frac{\mathbf{g}}{\epsilon}}+e^{\frac{\mathbf{a}-\rho}{\epsilon}}) \tag{11}$$

for any $\mathbf{a}\in\mathbb{R}^m$. We now set $\mathbf{a}$ to $\mathbf{f}$ and $\mathbf{g}$ respectively and obtain a stabilized Sinkhorn iteration,

$$\begin{aligned}
\mathbf{f}^{(l+1)} &\leftarrow \epsilon\log\boldsymbol{\alpha}+\mathbf{f}^{(l)}-\epsilon\log(e^{\frac{\mathbf{f}^{(l)}}{\epsilon}}\odot e^{-\frac{\mathbf{C}}{\epsilon}}e^{\frac{\mathbf{g}^{(l)}}{\epsilon}}+e^{\frac{\mathbf{f}^{(l)}-\rho}{\epsilon}}),\\
\mathbf{g}^{(l+1)} &\leftarrow \epsilon\log\boldsymbol{\beta}+\mathbf{g}^{(l)}-\epsilon\log(e^{\frac{\mathbf{g}^{(l)}}{\epsilon}}\odot e^{-\frac{\mathbf{C}^T}{\epsilon}}e^{\frac{\mathbf{f}^{(l+1)}}{\epsilon}}+e^{\frac{\mathbf{g}^{(l)}-\rho}{\epsilon}}),
\end{aligned} \tag{12}$$

for $l\geq 0$ with arbitary $\mathbf{f}^{(0)}$ and $\mathbf{g}^{(0)}$.

COT with l_2 penalty The algorithms for the COT with l_2 penalty can be derived similarly. To avoid solving the highly nonlinear systems, we first introduce two primal variables $\tilde{\boldsymbol{\mu}}$ and $\tilde{\boldsymbol{\nu}}$ leading to the following minimization problem,

$$\begin{aligned}
\min_{\mathbf{P},\boldsymbol{\mu},\boldsymbol{\nu},\tilde{\boldsymbol{\mu}},\tilde{\boldsymbol{\nu}}} \quad & \langle\mathbf{P},\mathbf{C}\rangle_{\mathrm{F}}+\epsilon_p H(\mathbf{P})+\epsilon_\mu H(\boldsymbol{\mu})+\epsilon_\nu H(\boldsymbol{\nu})+\frac{\rho}{2}(\|\tilde{\boldsymbol{\mu}}\|^2+\|\tilde{\boldsymbol{\nu}}\|^2)\\
\text{s.t.} \quad & \mathbf{P}\mathbf{1}^n=\boldsymbol{\alpha}-\boldsymbol{\mu},\\
& \mathbf{P}^T\mathbf{1}^m=\boldsymbol{\beta}-\boldsymbol{\nu},\\
& \boldsymbol{\mu}=\tilde{\boldsymbol{\mu}},\quad \boldsymbol{\nu}=\tilde{\boldsymbol{\nu}}.
\end{aligned} \tag{13}$$

The associated Lagrangian then reads

$$\begin{aligned}
\mathcal{E}(\mathbf{P},\boldsymbol{\mu},\boldsymbol{\nu},\tilde{\boldsymbol{\mu}},\tilde{\boldsymbol{\nu}};\mathbf{f},\mathbf{g},\mathbf{r},\mathbf{s}) =& \langle\mathbf{P},\mathbf{C}\rangle_{\mathrm{F}}+\epsilon_p H(\mathbf{P})+\epsilon_\mu H(\boldsymbol{\mu})+\epsilon_\nu H(\boldsymbol{\nu})+\frac{\rho}{2}(\|\tilde{\boldsymbol{\mu}}\|^2+\|\tilde{\boldsymbol{\nu}}\|^2)\\
&-\langle\mathbf{f},\mathbf{P}\mathbf{1}^n-(\boldsymbol{\alpha}-\boldsymbol{\mu})\rangle-\langle\mathbf{g},\mathbf{P}^T\mathbf{1}^m-(\boldsymbol{\beta}-\boldsymbol{\nu})\rangle\\
&-\langle\mathbf{r},\boldsymbol{\mu}-\tilde{\boldsymbol{\mu}}\rangle-\langle\mathbf{s},\boldsymbol{\nu}-\tilde{\boldsymbol{\nu}}\rangle.
\end{aligned} \tag{14}$$

Taking the first order derivatives leads to the first order conditions,

$$\begin{aligned}
\frac{\partial\mathcal{E}}{\partial\mathbf{P}_{ij}} &= \mathbf{C}_{ij}+\epsilon_p\log\mathbf{P}_{ij}-\mathbf{f}_i-\mathbf{g}_j=0 && \Rightarrow \quad \mathbf{P}=e^{\frac{\mathbf{f}\oplus\mathbf{g}-\mathbf{C}}{\epsilon_p}},\\
\frac{\partial\mathcal{E}}{\partial\boldsymbol{\mu}_i} &= \epsilon_\mu\log\boldsymbol{\mu}_i-\mathbf{f}_i-\mathbf{r}_i=0 && \Rightarrow \quad \boldsymbol{\mu}=e^{\frac{\mathbf{f}+\mathbf{r}}{\epsilon_\mu}},\\
\frac{\partial\mathcal{E}}{\partial\boldsymbol{\nu}_i} &= \epsilon_\nu\log\boldsymbol{\nu}_i-\mathbf{g}_i-\mathbf{s}_i=0 && \Rightarrow \quad \boldsymbol{\mu}=e^{\frac{\mathbf{g}+\mathbf{s}}{\epsilon_\nu}},\\
\frac{\partial\mathcal{E}}{\partial\tilde{\boldsymbol{\mu}}_i} &= \rho\tilde{\boldsymbol{\mu}}_i+\mathbf{r}_i && \Rightarrow \quad \tilde{\boldsymbol{\mu}}=-\frac{\mathbf{r}}{\rho},\\
\frac{\partial\mathcal{E}}{\partial\tilde{\boldsymbol{\nu}}_i} &= \rho\tilde{\boldsymbol{\nu}}_i+\mathbf{s}_i && \Rightarrow \quad \tilde{\boldsymbol{\nu}}=-\frac{\mathbf{s}}{\rho}.
\end{aligned} \tag{15}$$

We now obtain the Lagrange dual function by plugging these conditions to the Lagrangian $\mathcal{E}$,

$$
\begin{aligned}
L(\mathbf{f},\mathbf{g},\mathbf{r},\mathbf{s}) = &\min_{\mathbf{P},\boldsymbol{\mu},\boldsymbol{\nu},\tilde{\boldsymbol{\mu}},\tilde{\boldsymbol{\nu}}} \mathcal{E}(\mathbf{P},\boldsymbol{\mu},\boldsymbol{\nu},\tilde{\boldsymbol{\mu}},\tilde{\boldsymbol{\nu}};\mathbf{f},\mathbf{g},\mathbf{r},\mathbf{s}) \\
=&\langle \mathbf{f},\boldsymbol{\alpha}\rangle + \langle \mathbf{g},\boldsymbol{\beta}\rangle - \epsilon_p \langle e^{\frac{\mathbf{f}\oplus\mathbf{g}-\mathbf{C}}{\epsilon_p}}, \mathbf{1}^{m\times n}\rangle_{\mathrm{F}} \\
&+ \min_{\boldsymbol{\mu}} \epsilon_\mu H(\boldsymbol{\mu}) - \langle \mathbf{f}+\mathbf{r},\boldsymbol{\mu}\rangle + \min_{\boldsymbol{\nu}} \epsilon_\nu H(\boldsymbol{\nu}) - \langle \mathbf{g}+\mathbf{s},\boldsymbol{\nu}\rangle \\
&+ \min_{\tilde{\boldsymbol{\mu}}} \frac{\rho}{2}\|\tilde{\boldsymbol{\mu}}\|^2 - \langle \mathbf{r},\tilde{\boldsymbol{\mu}}\rangle + \min_{\tilde{\boldsymbol{\nu}}} \frac{\rho}{2}\|\tilde{\boldsymbol{\nu}}\|^2 - \langle \mathbf{s},\tilde{\boldsymbol{\nu}}\rangle \\
=&\langle \mathbf{f},\boldsymbol{\alpha}\rangle + \langle \mathbf{g},\boldsymbol{\beta}\rangle - \epsilon_p \langle e^{\frac{\mathbf{f}\oplus\mathbf{g}-\mathbf{C}}{\epsilon_p}}, \mathbf{1}^{m\times n}\rangle_{\mathrm{F}} \\
&- (\epsilon_\mu H)^*(\mathbf{f}+\mathbf{r}) - (\epsilon_\nu H)^*(\mathbf{g}+\mathbf{s}) - (\frac{\rho}{2}\|\cdot\|^2)^*(-\mathbf{r}) - (\frac{\rho}{2}\|\cdot\|^2)^*(-\mathbf{s}),
\end{aligned}
\tag{16}
$$

where $(\epsilon H)^*(y) = \epsilon e^{\frac{y}{\epsilon}}$ and $(\frac{\rho}{2}\|\cdot\|^2)^*(\mathbf{y}) = \frac{\|\mathbf{y}\|^2}{2\rho}$. Similar to the l_1 case, gradient based optimization methods can be used given the first order derivatives,

$$
\begin{aligned}
\frac{\partial L}{\partial \mathbf{f}} &= \boldsymbol{\alpha} - e^{\frac{\mathbf{f}}{\epsilon_p}} \odot (e^{-\frac{\mathbf{C}}{\epsilon_p}} e^{\frac{\mathbf{g}}{\epsilon_p}}) - e^{\frac{\mathbf{f}+\mathbf{r}}{\epsilon_\mu}}, \\
\frac{\partial L}{\partial \mathbf{g}} &= \boldsymbol{\beta} - e^{\frac{\mathbf{g}}{\epsilon_p}} \odot (e^{-\frac{\mathbf{C}^T}{\epsilon_p}} e^{\frac{\mathbf{f}}{\epsilon_p}}) - e^{\frac{\mathbf{g}+\mathbf{s}}{\epsilon_\nu}}, \\
\frac{\partial L}{\partial \mathbf{r}} &= -e^{\frac{\mathbf{f}+\mathbf{r}}{\epsilon_\mu}} - \frac{\mathbf{r}}{\rho}, \\
\frac{\partial L}{\partial \mathbf{s}} &= -e^{\frac{\mathbf{g}+\mathbf{s}}{\epsilon_\nu}} - \frac{\mathbf{s}}{\rho}.
\end{aligned}
\tag{17}
$$

Setting $\epsilon = \epsilon_p = \epsilon_\mu = \epsilon_\nu$, using the first order condition, and applying the log-sum-exp trick, we have the following Sinkhorn iteration,

$$
\begin{aligned}
\mathbf{f}^{(l+1)} &\leftarrow \epsilon \log \boldsymbol{\alpha} + \mathbf{f}^{(l)} - \epsilon \log(e^{\frac{\mathbf{f}^{(l)}}{\epsilon}} \odot e^{-\frac{\mathbf{C}}{\epsilon}} e^{\frac{\mathbf{g}^{(l)}}{\epsilon}} + e^{\frac{\mathbf{f}^{(l)}+\mathbf{r}}{\epsilon}}), \\
\mathbf{g}^{(l+1)} &\leftarrow \epsilon \log \boldsymbol{\beta} + \mathbf{g}^{(l)} - \epsilon \log(e^{\frac{\mathbf{g}^{(l)}}{\epsilon}} \odot e^{-\frac{\mathbf{C}^T}{\epsilon}} e^{\frac{\mathbf{f}^{(l+1)}}{\epsilon}} + e^{\frac{\mathbf{g}^{(l)}+\mathbf{s}}{\epsilon}}), \\
\mathbf{r}^{(l+1)} &\leftarrow -\epsilon\omega(\frac{\mathbf{f}^{(l+1)}}{\epsilon} - \log\frac{\epsilon}{\rho}), \\
\mathbf{s}^{(l+1)} &\leftarrow -\epsilon\omega(\frac{\mathbf{g}^{(l+1)}}{\epsilon} - \log\frac{\epsilon}{\rho}),
\end{aligned}
\tag{18}
$$

where ω is the Wright omega function.

2 Benchmark and comparison with PDE-based simulation

Ideally, CCC inference methods for spatial data should be validated by examining the spatial co-localization of ligand and receptor proteins. However, such data is often lacking. We therefore built partial differential equation (PDE) models to simulate CCC in space. The PDE model describes the essential physical processes in ligand-receptor interaction

including the diffusion of ligands, binding and dissociation of ligand-receptor complexes, and degradation of molecules [5] in 1-dimensional (1D) or 2-dimensional (2D) spaces (Extended Data Fig. 1a).

As an example, in the case of two ligand species and one receptor species (Extended Data Fig. 1a, b), the two ligands could be Wnt2 and Wnt5a and the receptor could be Fzd5 [6]. Then $[L_1]$ and $[L_2]$ describe the spatial distribution of free Wnt2 and Wnt5a whose production rates are observed from spatial data. Similarly, $[R]$ describes the spatial distribution of unoccupied Fzd5 whose initial distribution is taken from spatial data. Then $[L_1R]$ and $[L_2R]$ describe the spatial distribution of Wnt2-Fzd5 and Wnt5a-Fzd5 complexes, respectively, which reconstruct the CCC activity in space. The quality of the inferred CCC will be assessed based on how accurately it reproduces the simulated spatial distributions of the ligand-receptor complexes.

2.1 Model

Assume there are n_l ligand and n_r receptor species where each ligand or receptor species might bind to multiple others. Let $[L_i], [R_j], [L_iR_j] : \mathbb{R}^{n_{\text{grid}} \times 2} \to \mathbb{R}$ denote the spatial distributions of ligand i, receptor j and the complex formed by their binding, the 1) diffusion and degradation of ligand and 2) binding and dissociation of ligand-receptor complex are modeled in the following equations,

$$\begin{aligned}\frac{\partial [L_i]}{\partial t} &= D_i \nabla^2 [L_i] - \sum_{j:(i,j)\in I} a_{(i,j)}[L_i][R_j] + \sum_{j:(i,j)\in I} b_{(i,j)}[L_iR_j] - c_i[L_i],\\ \frac{\partial [L_iR_j]}{\partial t} &= a_{(i,j)}[L_i][R_j] - b_{(i,j)}[L_iR_j],\\ \frac{\partial [R_j]}{\partial t} &= - \sum_{i:(i,j)\in I} a_{(i,j)}[L_i][R_j] + \sum_{i:(i,j)\in I} b_{(i,j)}[L_iR_j],\end{aligned} \tag{19}$$

for $(i, j) \in I$, the index set of ligand and receptor that can bind. For each ligand-receptor pair, ligand i and receptor j that can bind, $a_{(i,j)}$ and $b_{(i,j)}$ are the association rate and dissociation rate between them. For ligand i, c_i is its degradation rate and D_i is its diffusivity in space.

2.2 Synthetic data generation

Synthetic benchmark data for validating the collective optimal transport method is generated by simulation of the PDE models described in Eq. (19). Various cases of ligand-receptor binding are considered which have varying numbers of ligand species, receptor species, and ligand-receptor complexes, to account for different complexities and levels of competition among species (Supplementary Figs. 1-4). For each case, ten independent simulations were carried out. For each simulation, the initial distribution of ligands and receptors were generated by using multiple Gaussian distributions with random bandwidths and random locations in a square domain. All ligand-receptor complexes were initiated to be zero.

For all simulations, a natural boundary condition (no-flux) was assumed and a 2D 50-by-50 grid was used. The simulations were implemented using the py-pde package [7]. The solver was set to "scipy" which uses the "scipy.integrate.solve_ivp" utility in SciPy [8] that implements the explicit Runge-Kutta method of order 5(4). The simulation was run until the concentrations of the ligand-receptor complexes reach (numerical) equilibrium. The concentrations of the ligand-receptor complexes depict the spatial distributions of each receptor species occupied by each ligand species. The data analysis methods (optimal transport based method) are then benchmarked based on their performance of reconstructing these spatial distributions.

2.3 Benchmark and comparison result

We first illustrate COMMOT in two simulated examples in 1D and 2D. Specifically, the inferred spatial distribution of bound ligand-receptor complexes is compared with the simulated one. In the 1D physical model, the two ligand species are located on the two sides of the location of receptor, respectively and will compete to bind to the receptor with a limit spatial range of interactions. The effects of symmetric ligand locations, biased ligand locations, and different ligand diffusivities are correctly captured by collective optimal transport (Extended Data Fig. 1a). In the 2D physical model, five interacting species are modeled, and the interaction patterns are also recapitulated (Extended Data Fig. 1b). In addition to revealing ligand-receptor complexes, collective optimal transport also annotates the source of ligands that contributed to the ligand-receptor complexes (Extended Data Fig. 1a,b).

Further quantitative comparison is carried out using simulations with randomly generated spatial expression of ligands and receptors in various cases of ligand-receptor binding (Extended Data Fig. 1c). To account to different ligand-receptor binding situations, ten different cases are considered with different numbers of ligand and receptor species and different levels of competition (the ratio of interacting pairs over total number of species) among the species (Extended Data Fig. 1d). For each case, ten random initializations are generated. The levels of bound ligand-receptor complex inferred by collective optimal transport are compared to the PDE simulation results using the root mean square error and Spearman's correlation coefficient. Our results show that collective optimal transport consistently derives accurate predictions across the different cases and outperforms the results by evaluating each ligand-receptor pair independently using traditional optimal transport. The improvement in the results from the pairwise approach is especially significant when competition among species is high (Extended Data Fig. 1d, Supplementary Figs. 1-4), for example, cases 4, 5 and 10. A comparison with simulated data demonstrates that collective optimal transport used by COMMOT accurately reconstructs CCC in space. The ability to handle non-probability distribution is especially useful when the total levels of ligand and receptor expressions are significantly different (Supplementary Fig. 10). Also, incorporating spatial information helps prioritizing interactions among cells that are spatially close (Supplementary Fig. 11). Moreover, our method outperforms two other variants of optimal transport that also do not require the input data to be probability distributions: unbalanced optimal transport and partial optimal transport (Supplementary Figs. 5-6). In unbalanced optimal transport, the commonly used KL divergence is adapted in this comparison while other divergence forms may be incorporated as well. The differences among

these optimal transport variants are further illustrated by both a one-dimensional example (Supplementary Fig. 7) and with real ST data (Supplementary Fig. 8-9), showing again the advantages in using COMMOT.

3 Joint analysis of mouse cortex with different datasets

We jointly analyzed CCC in mouse cortex datasets generated with three different technologies including Visium, seqFISH+, and STARmap (Extended Data Figs. 3-5).

Across these three datasets, AGT signaling was identified in neurons. Spatially, neurons in the L2-3 region were identified as strong receivers of AGT ligands across the three datasets. Interestingly, a striped signaling pattern was observed, wherein strong signals within individual layers form stripes, while weak signals form inter-stripe regions. Strong AGT signaling activity among oligodendrocytes was also identified in both STARmap and seqFISH+ datasets. (Extended Data Fig. 3).

In both Visium and seqFISH+ cortex datasets, we inferred WNT signaling to be active across different cortical layers. In both datasets, we identified WNT signaling to be relatively low in layer 5, compared to other layers. This result is in line with previous study showing that disruption to WNT signaling in Lrp6 mutant mice leads to cortical hypoplasia and reduced proliferation in neurons in layers 2-4 and 6, with neurons in layer 5 remaining relatively unchanged [9] (Extended Data Fig. 4).

TAC (tachykinin neuropeptide family) signaling activity was consistently found in both Visium and STARmap cortex datasets to be active in non-neuronal cells and in inhibitory neurons, especially in somatostatin-expressing neurons (Sst), because of the high expression of tachykinin receptor-3 in Sst neurons [10] (Extended Data Fig. 5).

4 Robustness evaluation of COMMOT on drosophila embryo data

For the robustness study, we utilized the stage 6 Drosophila embryo, an extensively studied system, which contains approximately 6000 cells that have been extensively studied with in situ databases available that present systematically annotated spatial gene expression [11, 12]. The paired data consists of scRNA-seq of 1297 cells and 8925 genes along with the spatial single-cell resolution data of 3039 cells and 84 genes [13], with both good depth and spatial organization. We first integrated them using SpaOTsc [14] to generate two imputed datasets, the imputed scRNA-seq with predicted spatial coordinates of cells and the imputed spatial data with predicted expression of the 8925 genes from the scRNA-seq data. Using subsampling as a test for robustness, we randomly subsampled the data and compared them to the results from the full dataset. When decreasing the subsample size, the cosine distance (for spatial signaling direction) and Jaccard distance (for cluster level signaling) were found to remain relatively small showing that both signaling directions and cluster level signaling are robustly captured in the subsampled data (Extended Data Fig. 6). The differentially expressed signaling genes were then identified by using the inferred amount of received signal as the co-factor, analogous to the approach for pseudotime [15].

The DE genes identified from subsampled datasets were found to be comparable to the ones identified from the full dataset (Extended Data Fig. 6).

The spatial signaling direction is described by a vector field defined on n grid points discretizing the tissue space, and is represented by an array $\mathbf{V} \in \mathbb{R}^{n\times 2}$. Cosine distance is used to compare the vector field $\mathbf{V}_{\text{sub}}$ from subsampled data to the one from the full data $\mathbf{V}_{\text{full}}$ and is defined as

$$d_{cos}(\mathbf{V}_{\text{full}}, \mathbf{V}_{\text{sub}}) = \sum_i \|\mathbf{V}_{\text{full}}(i)\|[1 - \mathbf{V}_{\text{full}}(i) \cdot \mathbf{V}_{\text{sub}}(i)/(\|\mathbf{V}_{\text{full}}(i)\|\|\mathbf{V}_{\text{sub}}(i)\|)]/\sum_i \|\mathbf{V}_{\text{full}}(i)\|.$$

To compare two cluster level CCC networks $\mathbf{S}_1^{cl}$ and $\mathbf{S}_2^{cl}$, we first binarize them such that the edges with p-value smaller than 0.05 are kept in the edge sets $\bar{\mathbf{S}}_1^{cl}$ and $\bar{\mathbf{S}}_2^{cl}$. Then, the Jaccard distance is used for quantitative comparison,

$$d_{Jaccard}(\bar{\mathbf{S}}_1^{cl}, \bar{\mathbf{S}}_2^{cl}) = 1 - |\bar{\mathbf{S}}_1^{cl} \cap \bar{\mathbf{S}}_2^{cl}|/|\bar{\mathbf{S}}_1^{cl} \cup \bar{\mathbf{S}}_2^{cl}|.$$

5 Comparison to CellChat, Giotto, and CellphoneDB

Using Visium human breast cancer and mouse brain datasets, we studied the difference between COMMOT and the three other methods. When assessing the distributions of inter-cluster spatial distance of the identified significant CCCs, the significant CellChat interactions have a relative uniform distribution of the spatial distances. This is expected since CellChat was designed for scRNA-seq data and spatial organization is not considered in the method. Both COMMOT and Giotto prioritize CCC between spatially close clusters while COMMOT identifies more CCC links that are beyond the neighboring cells because Giotto uses cell-contact graph that might limit the range of inferred CCC. CellPhoneDB v3 identifies more CCC links than COMMOT potentially due to its consideration of inter-cluster distances rather than distance among individual cells (Supplementary Figs. 31-36). In comparison to CellChat, COMMOT uniquely detects CCC that are not differentially expressed in clusters but spatially significant (Supplementary Figs. 31-32). Giotto characterizes the average expression of ligands and receptors of spatially neighboring spots without providing the role for each spot in CCC. COMMOT identifies each spot as a sender, receiver or both in CCC with interpretable results (Supplementary Figs. 33-34). COMMOT identifies CCC that is locally significant which CellPhoneDB v3 may neglect due to its consideration of expression level of entire clusters (Supplementary Figs. 35-36).

Furthermore, COMMOT can identify localized signaling hotspots compared to cluster-level approaches (Supplementary Figs. 37-38). For a specific ligand-receptor pair, COMMOT further prioritizes regions containing its high signaling activity with low competitions from other pairs (Supplementary Figs. 39-40).

References

[1] Cédric Villani. *Optimal transport: old and new*, volume 338. Springer, 2009.

[2] Gabriel Peyré, Marco Cuturi, et al. Computational optimal transport: With applications to data science. *Foundations and Trends® in Machine Learning*, 11(5-6):355–607, 2019.

[3] Marco Cuturi. Sinkhorn distances: Lightspeed computation of optimal transport. *Advances in neural information processing systems*, 26:2292–2300, 2013.

[4] Yurii Nesterov. *Introductory lectures on convex optimization: A basic course*, volume 87. Springer Science & Business Media, 2003.

[5] ArthuráD Lander. Pattern, growth, and control. *Cell*, 144(6):955–969, 2011.

[6] Jordan A Ramilowski, Tatyana Goldberg, Jayson Harshbarger, Edda Kloppmann, Marina Lizio, Venkata P Satagopam, Masayoshi Itoh, Hideya Kawaji, Piero Carninci, Burkhard Rost, et al. A draft network of ligand–receptor-mediated multicellular signalling in human. *Nature communications*, 6(1):1–12, 2015.

[7] David Zwicker. py-pde: A python package for solving partial differential equations. *Journal of Open Source Software*, 5(48):2158, 2020.

[8] Pauli Virtanen, Ralf Gommers, Travis E. Oliphant, Matt Haberland, Tyler Reddy, David Cournapeau, Evgeni Burovski, Pearu Peterson, Warren Weckesser, Jonathan Bright, Stéfan J. van der Walt, Matthew Brett, Joshua Wilson, K. Jarrod Millman, Nikolay Mayorov, Andrew R. J. Nelson, Eric Jones, Robert Kern, Eric Larson, C J Carey, İlhan Polat, Yu Feng, Eric W. Moore, Jake VanderPlas, Denis Laxalde, Josef Perktold, Robert Cimrman, Ian Henriksen, E. A. Quintero, Charles R. Harris, Anne M. Archibald, Antônio H. Ribeiro, Fabian Pedregosa, Paul van Mulbregt, and SciPy 1.0 Contributors. SciPy 1.0: Fundamental Algorithms for Scientific Computing in Python. *Nature Methods*, 17:261–272, 2020.

[9] C-J Zhou, U Borello, JLR Rubenstein, and SJ Pleasure. Neuronal production and precursor proliferation defects in the neocortex of mice with loss of function in the canonical wnt signaling pathway. *Neuroscience*, 142(4):1119–1131, 2006.

[10] Stephen J Smith, Uygar Sümbül, Lucas T Graybuck, Forrest Collman, Sharmishtaa Seshamani, Rohan Gala, Olga Gliko, Leila Elabbady, Jeremy A Miller, Trygve E Bakken, et al. Single-cell transcriptomic evidence for dense intracortical neuropeptide networks. *elife*, 8:e47889, 2019.

[11] Eric Lécuyer, Hideki Yoshida, Neela Parthasarathy, Christina Alm, Tomas Babak, Tanja Cerovina, Timothy R Hughes, Pavel Tomancak, and Henry M Krause. Global analysis of mrna localization reveals a prominent role in organizing cellular architecture and function. *Cell*, 131(1):174–187, 2007.

[12] Pavel Tomancak, Benjamin P Berman, Amy Beaton, Richard Weiszmann, Elaine Kwan, Volker Hartenstein, Susan E Celniker, and Gerald M Rubin. Global analysis of patterns of gene expression during drosophilaembryogenesis. *Genome biology*, 8(7):1–24, 2007.

[13] Nikos Karaiskos, Philipp Wahle, Jonathan Alles, Anastasiya Boltengagen, Salah Ayoub, Claudia Kipar, Christine Kocks, Nikolaus Rajewsky, and Robert P Zinzen. The drosophila embryo at single-cell transcriptome resolution. *Science*, 358(6360):194–199, 2017.

[14] Zixuan Cang and Qing Nie. Inferring spatial and signaling relationships between cells from single cell transcriptomic data. *Nature communications*, 11(1):1–13, 2020.

[15] Koen Van den Berge, Hector Roux de Bézieux, Kelly Street, Wouter Saelens, Robrecht Cannoodt, Yvan Saeys, Sandrine Dudoit, and Lieven Clement. Trajectory-based differential expression analysis for single-cell sequencing data. *Nature communications*, 11(1):1–13, 2020.
