# Supplementary material for: Screening cell–cell communication in spatial transcriptomics via collective optimal transport
Source: Nat Methods. 2023 Jan 23;20(2):218–28. doi: 10.1038/s41592-022-01728-4 (PMC9911355; doi:10.1038/s41592-022-01728-4)
Supplement: Supplementary file 2 — Reporting Summary [file 41592_2022_1728_MOESM2_ESM.pdf]

nature portfolio

Corresponding author(s): Qing Nie

Last updated by author(s): Oct 31, 2022

Reporting Summary

Nature Portfolio wishes to improve the reproducibility of the work that we publish. This form provides structure for consistency and transparency in reporting. For further information on Nature Portfolio policies, see our Editorial Policies and the Editorial Policy Checklist.

Statistics

For all statistical analyses, confirm that the following items are present in the figure legend, table legend, main text, or Methods section.

n/a	Confirmed	
☐	☒	The exact sample size (*n*) for each experimental group/condition, given as a discrete number and unit of measurement
☐	☒	A statement on whether measurements were taken from distinct samples or whether the same sample was measured repeatedly
☒	☐	The statistical test(s) used AND whether they are one- or two-sided<br>*Only common tests should be described solely by name; describe more complex techniques in the Methods section.*
☒	☐	A description of all covariates tested
☒	☐	A description of any assumptions or corrections, such as tests of normality and adjustment for multiple comparisons
☐	☒	A full description of the statistical parameters including central tendency (e.g. means) or other basic estimates (e.g. regression coefficient) AND variation (e.g. standard deviation) or associated estimates of uncertainty (e.g. confidence intervals)
☐	☒	For null hypothesis testing, the test statistic (e.g. *F*, *t*, *r*) with confidence intervals, effect sizes, degrees of freedom and *P* value noted<br>*Give P values as exact values whenever suitable.*
☒	☐	For Bayesian analysis, information on the choice of priors and Markov chain Monte Carlo settings
☒	☐	For hierarchical and complex designs, identification of the appropriate level for tests and full reporting of outcomes
☐	☒	Estimates of effect sizes (e.g. Cohen's *d*, Pearson's *r*), indicating how they were calculated

Our web collection on statistics for biologists contains articles on many of the points above.

Software and code

Policy information about availability of computer code

Data collection	No software was used for data collection.
Data analysis	The open-source software has been uploaded to Github (https://github.com/zcang/COMMOT) and analysis code and results will be uploaded to Zenodo upon publication. The following versions were used for the software mentioned in the manuscript: CellChat version 1.1.3, Giotto version 1.0.3, CellPhoneDB version 3.1.0, tradeSeq version 1.0.1, scikit-learn version 1.0.2.

For manuscripts utilizing custom algorithms or software that are central to the research but not yet described in published literature, software must be made available to editors and reviewers. We strongly encourage code deposition in a community repository (e.g. GitHub). See the Nature Portfolio guidelines for submitting code & software for further information.

Data

Policy information about availability of data

All manuscripts must include a data availability statement. This statement should provide the following information, where applicable:

- Accession codes, unique identifiers, or web links for publicly available datasets
- A description of any restrictions on data availability
- For clinical datasets or third party data, please ensure that the statement adheres to our policy

The original public data used in this work can be accessed through the following links: (1) Drosophila embryo spatial and scRNA-seq data: Dream Single cell Transcriptomics Challenge through Synapse ID (syn15665609) [Karaiskos, N. et al., Science, 2017]. (2) Human epidermal scRNA-seq data [Wang, S. et al., Nat.

Commun., 2020]: GEO assession codes, GSE147482. (3) Mouse hypothalamic preoptic region MERFISH data [Moffitt, J. R. et al., Science, 2018]: original data available at Dryad[Moffitt, J. R. et al., Dryad, Dataset, 2018] through the link https://doi.org/10.5061/dryad.8t8s248. This work used the preprocessed data through the Squidpy package [Palla, G. et al., Nat. Methods, 2022] with the utility squidpy.datasets.merfish. (4) Mouse placenta STARmap data [He, Y. et al., Nat. Commun., 2021]: downloaded from Code Ocean (https://codeocean.com/capsule/9820099/tree/v1) with DOI: 10.24433/CO.6072400.v1. (5) Mouse brain STARmap data [Wang, X. et al., Science, 2018]: the processed data was downloaded from the same repository as the mouse placenta STARmap data. (6) Mouse somatosensory cortex seqFISH+ data [Eng, C.-H. L. et al., Nature, 2019]: downloaded through Giotto package [Dries, R. et al., Genome Biol., 2021]. (7) Mouse hippocampus Slide-seqV2 data [Stickels, R. R. et al., Nat. Biotechnol., 2020]: downloaded from Broad Institute Single Cell Portal (https://singlecell.broadinstitute.org/single_cell/study/SCP815/sensitive-spatial-genome-wide-expression-profiling-at-cellular-resolution#study-summary). (8) Breast cancer Visium data: downloaded from 10X Genomics website (https://www.10xgenomics.com/resources/datasets/human-breast-cancer-block-a-section-1-1-standard-1-1-0). (9) Mouse brain (sagittal posterior) Visium data: downloaded from 10X Genomics website (https://www.10xgenomics.com/resources/datasets/mouse-brain-serial-section-1-sagittal-anterior-1-standard-1-1-0).

The ligand-receptor pairs with secreted ligand based on CellChatDB database [Jin, S. et al., Nat. Commun., 2021] were used and can be accessed at http://www.cellchat.org/cellchatdb/. The downstream target genes were taken from scSeqComm [Baruzzo, G. et a., Bioinformatics, 2022] and the target gene libraries named TF_TG_TRRUSTv2 and TF_TG_TRRUSTv2_RegNetwork_High_mouse were used for human and mouse respectively.

Human research participants

Policy information about studies involving human research participants and Sex and Gender in Research.

Reporting on sex and gender	All tissue samples were archived discarded and de-identified neonatal foreskins not collected for the purpose of this study. All tissues are from males due to the nature of the tissue. No other covariants were used in the collection of the tissues.
Population characteristics	All human samples were from archived tissue not collected for the purpose of this study and our group was blinded to all characteristics of human subjects.
Recruitment	Not applicable. Tissue was collected as discarded and de-identified samples from available newborns.
Ethics oversight	Institutional Review Board of the University of California, Irvine.

Note that full information on the approval of the study protocol must also be provided in the manuscript.

Field-specific reporting

Please select the one below that is the best fit for your research. If you are not sure, read the appropriate sections before making your selection.

☒ Life sciences ☐ Behavioural & social sciences ☐ Ecological, evolutionary & environmental sciences

For a reference copy of the document with all sections, see nature.com/documents/nr-reporting-summary-flat.pdf

Life sciences study design

All studies must disclose on these points even when the disclosure is negative.

Sample size	No sample size calculations were performed for experiments. Discarded and de-identified human foreskin samples were used for immunohistochemical analysis with at least 3 biological replicates. Biological replicate sample size and size sufficiency was chosen due to the similarity of immunohistochemical staining.
Data exclusions	No data was excluded from the analysis.
Replication	All experiments were reproduced a minimum of three times.
Randomization	No randomization was necessary for immunohistochemical analysis because all samples were used to describe pathway status.
Blinding	Single cell RNA-seq and spatial transcriptomic analyses were unbiased. All cells were analyzed using computational algorithms that were not biased to recognize any particular cell types. All available tissue were used for immunohistochemical analysis. As only wild-type tissue was used with no manipulations, no blinding was necessary.

Reporting for specific materials, systems and methods

We require information from authors about some types of materials, experimental systems and methods used in many studies. Here, indicate whether each material, system or method listed is relevant to your study. If you are not sure if a list item applies to your research, read the appropriate section before selecting a response.

Materials & experimental systems

n/a	Involved in the study
☐	☒ Antibodies
☒	☐ Eukaryotic cell lines
☒	☐ Palaeontology and archaeology
☒	☐ Animals and other organisms
☒	☐ Clinical data
☒	☐ Dual use research of concern

Methods

n/a	Involved in the study
☒	☐ ChIP-seq
☒	☐ Flow cytometry
☒	☐ MRI-based neuroimaging

Antibodies

Antibodies used

The following antibodies were used: mouse anti-KRT5 (1:100; Santa Cruz Biotechnilogy; sc-32721), mouse anti-KRT15 (1:100; Santa Cruz Biotechnology; sc-47697), mouse anti-BCAM (1:100; Santa Cruz Biotechnology; sc-365191), mouse anti-FGF7 (1:100; Santa Cruz Biotechnology; sc-365440), mouse anti-STMN1 (1:100; Santa Cruz Biotechnology; sc-48362); mouse anti-IGFBP6 (1:500; Abgent; AP6764b); mouse anti-PMAIP1 (1:100; Santa Cruz Biotechnology; sc-56169), mouse anti-POSTN (1:100; Santa Cruz Biotechnology; sc-398631); mouse anti-FLG (1:100; Santa Cruz Biotechnology; sc-66192); rabbit anti-LOR (1:1000; abcam; ab85679); mouse anti-TYRO3 (1:100; LSBio; LS-C114523-100); rabbit anti-GAS6 (1:100; abcam; ab227174); and rabbit anti-PROS1 (1:100; Proteintech; 16910-1-AP). Secondary antibodies include Cy3 AffiniPure (1:500; Jackson ImmunoResearch; 711-165-152, 111-165-003).

Validation

mouse anti-KRT5 (1:100; Santa Cruz Biotechnilogy; sc-32721): Sung JS et al. 2020. Oncogene. 39(3):664-676.
mouse anti-KRT15 (1:100; Santa Cruz Biotechnology; sc-47697): Busslinger GA et al. 2021. Cell Rep. 34(10):108819.
mouse anti-BCAM (1:100; Santa Cruz Biotechnology; sc-365191): Zhao J et al. 2022. Clin Epigenetics. 14(1):99.
mouse anti-FGF7 (1:100; Santa Cruz Biotechnology; sc-365440): Chen X et al. 2022. Br J Pharmacol. 179(5):1102-1121.
mouse anti-STMN1 (1:100; Santa Cruz Biotechnology; sc-48362): Hu Z et al. 2020. Cancer Cell. 37(2):226-242.e7.
mouse anti-IGFBP6 (1:500; Abgent; AP6764b): manufacturer validated with human samples via western blot and IHC. Synthetic peptide of human IFGBP6 used as an antigen.
mouse anti-PMAIP1 (1:100; Santa Cruz Biotechnology; sc-56169): Palanikumar L et al. 2021. Nat Commun. 12(1):3962.
mouse anti-POSTN (1:100; Santa Cruz Biotechnology; sc-398631): Mircea M et al. 2021. Genome Biol. 23(1):18.
mouse anti-FLG (1:100; Santa Cruz Biotechnology; sc-66192): Dai X et al. 2022. J Invest Dermatol. 142:136-144.e3.
rabbit anti-LOR (1:1000; abcam; ab85679): Zhou Q et al. 2021. J Invest Dermatol. 141:152-163.
mouse anti-TYRO3 (1:100; LSBio; LS-C114523-100): manufacturer validated with human samples via western blot and IHC. Full length recombinant protein of human TYRO3 used as an antigen.
rabbit anti-GAS6 (1:100; abcam; ab227174): manufacturer validated with human samples via western blot and IHC. Recombinant fragment protein of human GAS6 used as an antigen.
rabbit anti-PROS1 (1:100; Proteintech; 16910-1-AP): Wang ZH et al. 2015. Mol Med Rep. 12(3):3279-3284.
