# Supplementary material for: Screening cell–cell communication in spatial transcriptomics via collective optimal transport
Source: Nat Methods. 2023 Jan 23;20(2):218–28. doi: 10.1038/s41592-022-01728-4 (PMC9911355; doi:10.1038/s41592-022-01728-4)
Supplement: Supplementary file 3 — Peer Review File [file 41592_2022_1728_MOESM3_ESM.pdf]

natureresearch

Peer Review Information

Journal: Nature Methods
Manuscript Title: Screening cell-cell communication in spatial transcriptomics via collective optimal transport
Corresponding author name(s): Qing Nie

Editorial Notes:

Reviewer Comments & Decisions:

Decision Letter, initial version:

Dear Dr Nie,

Your Article entitled "Screening cell-cell communication in spatial transcriptomics via collective optimal transport" has now been seen by 3 reviewers, whose comments are attached. While they find your work of potential interest, they have raised serious concerns which in our view are sufficiently important that they preclude publication of the work in Nature Methods, at least in its present form.

As you will see, the reviewers raise serious concerns about the lack of comparisons and ask for additional benchmarking studies using simulation and real datasets.

Should further experimental data allow you to fully address these criticisms we would be willing to look at a revised manuscript (unless, of course, something similar has by then been accepted at Nature Methods or appeared elsewhere). This includes submission or publication of a portion of this work somewhere else. We hope you understand that until we have read the revised paper in its entirety we cannot promise that it will be sent back for peer-review.

If you are interested in revising this manuscript for submission to Nature Methods in the future, please contact me to discuss your appeal before making any revisions. Otherwise, we hope that you find the reviewers' comments helpful when preparing your paper for submission elsewhere.

best regards and happy holidays,

Lei

Lei Tang, Ph.D.
Senior Editor
Nature Methods

Reviewers' Comments:

Reviewer #1:
Remarks to the Author:
Cang et al present COMMOT, a computational method used on optimal transport (OT) to study cell0-cell communication from spatially resolved transcriptomics data.

This type of data is becoming increasingly available and it enables to look at cell-communication with the additional insight of where cells are located, and hence computational methods that leverage this knowledge are timely. COMMOT presents a number of novel features. The method seems mathematically sound. The authors introduce a variant of optimal transport, called collective optimal transport (COT) which considers multiple source and target species simultaneously. They provide two iterative algorithms based on stabilized Sinkhorn iterations to find near optimal solutions. COMMOT is available as a package for use by the community.

While we welcome COMMOT to the palette of methods in the field, we have the following questions and concerns:

1 - The authors make a strong case of how their formulation takes into account the absolute values of ligands and receptors instead of normalised values, as well as the competition for receptor by ligands. However, these are phenomena that occur at the level of proteins (ligands and receptors are protein, not RNA molecules), and hence is not clear what a real impact this has at the level or RNA, given the limited correlation of proteins and RNA, plus all modifications that happen to proteins, etc.
1.1. Could authors not apply their method to spatially resolved protein data?

2- The simulated data is interesting and a helpful control, but not a 'validation' as it is labeled in Figure 2. The model uses the same concepts as COMMOT, based on modeling diffusion as a function of abundance; so it is reassuring but not surprising that COMMOT works there. Perhaps should be a supplementary note.
Also related to the previous point, it is based on in silicon data that represents proteins (ligands and receptors), not transcripts.

3 - Also, the data generated with SpaOTsc is built generating OT as well, so is in that senes a bit redundant: SpaOTsc uses the assumption that cell co-localize with similarity of expression, that also implies that structure is driven by diffusion. Actually, why generating from suspension data spatial data to try COMMOT, when there are so many spatial data sets?

4- - Computational efficiency and scalability of the proposed algorithms: It is not clear from their experiments how the algorithms perform in terms of computation time, and their scalability to large instances should be investigated. The model with l1 norm penalty on the slacks can be easily cast as a linear program (transportation problem). This formulation permits setting the coupling pairs with infinity cost to zero, and thus removing them from the LP. This results in an LP with significantly fewer variables, and possibly easy to solve, either using a solver or using tailored combinatorial algorithms. A comparison in terms of scalability of these formulation would be helpful.

5 - it is very challenging to know if this (or any other related method) is ‘working’, as we lack true experimental ground truths of cellular communication. Authors should clarify or soften statements such as ‘succesfully applied’. When is successfully applied? When the results seem to agree with our current knowledge from literature? That is very hard to asses and prone to biases.
5.1. - While not real validations, helpful analysis that the authors should perform include diverse tests of the statistical significance of their solutions. For example, by random permutation of cells in each of the sample(slide). And if possible also consider some ‘prediction task’ and show how COMMOT helps to predict unseen data.

Minor:
1- Authors take the ligand-receptor pairs from Fantom database, which is relatively old. there are many others after that one and they provide different results, so authors should explore this.

2 - with the random forest model, there is a danger of diluting the importance over all correlated variables because they are also correlated with each other by transition.

3 - Notational consistency: the mathematical notations in the body of the paper and those employed in the supplementary appendix are inconsistent in some cases. For instance, n_s is used for referring to points (cells/spots) in the main body of the paper, whereas in the appendix, it seems that the notation is n_p, while n_s is used for source species. These inconsistencies become confusing in some points, making it harder to follow than necessary.

Reviewer #2:

Remarks to the Author:
In this manuscript, the authors developed a novel computational framework COMMOT to infer cell-cell communications based on optimal transport. COMMOT is capable of 1) preserving the original units without the need for normalization, 2) enforcing spatial range limits of signaling, and 3) handling the cases of multiple competing species. Overall, the method is novel in concept. It is also of immediate interest to the field. However, several issues must be addressed before publication.

At a high level, most of the commentary is around some figures lacking quantitative support and a lack of robust comparisons against many baselines and ground truth.

Major:
1. Why do authors only compare with pairwise traditional optimal transport?
2. Can the failure modes of not doing normalization or considering spatial information be demonstrated with your method? By normalizing the distributions and showing inferior results? By making an identity distance matrix and showing false positives?
3. The authors only showed signaling pathways super families e.g., WNT, TGFb. Is this cherry-picking? Or are these the most obvious ones? The authors need to show COMMOT is generalizable to more examples of ligand-receptor interactions when interactions are more specific such as PD1-PDL1 (if data available).
4. In Fig 4, the authors showed signaling DE genes from imputed scRNA-seq data. The immunostaining didn't directly validate the L-R interactions. The authors need to show at least spatial validation of L-R expression in situ using either smFISH or immunostaining. Same for Figs 3, 5, 6.
5. The authors need to benchmark COMMOT with other methods across different datasets and demonstrate superior performance.
6. Mentions that interactions do not exist outside of a certain spatial radius: What is the cutoff for a certain LR pair (please provide the logic behind it) and why use this cutoff/infinity method?
7. In Figure 4a, the authors used 208 cells and 12 genes to integrate with scRNA-seq data containing 4264 cells and 33694 genes. I am concerned about the integration given the small number of genes (12!). Thus, all the downstream analysis.

Minor:

1. There is a difference in the naming of the method in the text and some of the figures (collective optimal transport vs supervised optimal transport);
2. Can the authors further demonstrate COMMOT is better than spatial proximity graph with known biological L-R interactions?
3. There are other spatial transcriptomics data available such as MERFISH, STARmap. The authors need to demonstrate the performance on more datasets.

Reviewer #3:
Remarks to the Author:
Review of "Screening cell-cell communication in spatial transcriptomics via collective optimal transport" (NMETH-A47293)

Summary

With COMMOT, Cang et al., present an elegant mathematical solution to the problem of inferring cell-cell communication (CCC) from spatial transcriptomics data based on a variant of optimal transport (OT). Their method considers competition among different ligand-receptor species, includes spatial constraints and does not require the input measures (ligand/receptor expression) to be normalized, thus preserving the original measurement units. The method is applied to spatial datasets of different sizes (~1k to ~50k spots or cells) and technologies, including both imaging (seqFISH+) and sequencing (Visium, slide-seqV2) based approaches and robustness of results is shown. Further, the authors show how their method can be used in different biological contexts, including mouse breast cancer and the brain. The concept figure gives a clear overview of their method and subsequent figures are well-organized. Further, the software package is well organized and documented, includes a tutorial and interfaces with standard single-cell packages such as AnnData and Scanpy.

However, I have concerns about the relationship between their method ("collective optimal transport") and previous OT extensions, in particular, multimarginal, partial and unbalanced OT, which individually solve the same problems the authors outline. These connections should be made explicit, cited accordingly and it needs to be clearly demonstrated why a new OT extension is necessary to clearly delineate novelty. Further, the authors do not benchmark their method against any other established method for CCC inference, they only consider a simple OT approach as baseline. This makes me wonder what the added benefit of their method is in practice. This needs to be clearly demonstrated using both their simulation study as well as one of their real data examples.

As it stands, the authors present an elegant mathematical concept with a good implementation, but fall short of demonstrating how their method gives practical advantages over existing approaches when applied to real biological settings. Are there new biological questions that can be studied with this approach, which could not have been studied equally well with existing approaches? I give details about my concerns in major and minor comments below.

Major comments

Introduction, paragraph starting in line 63, the authors motivate the need for developing a new optimal transport (OT) method. They do so in line 71 by claiming

“However, when utilizing existing optimal transport methods, ligand and receptor expression are normalized as probability distributions, and therefore, the original units of expression levels are not preserved making the distributions of ligand and receptor incomparable.”

From my point of view, this claim is incorrect. While it is correct that the standard OT problem is formulated for probability distributions (hence requiring normalization of ligand and receptor expression), unbalanced OT resolves this problem, as outlined in chapter 10.2 of ref. (Peyré and Cuturi 2019), which the authors cite in the same sentence. I quote from ref. (Peyré and Cuturi 2019):

“In practice, unbalanced OT techniques seem to outperform classical OT for applications (such as in imaging or machine learning) where the input data is noisy or not perfectly known. They are also crucial when the signal strength of a measure, as measured by its total mass, must be accounted for, or when normalization is not meaningful. This was the original motivation of Frogner et al. [2015], whose goal was to compare sets of word labels used to describe images. Unbalanced OT and the corresponding Sinkhorn iterations have also been used for applications to the dynamics of cells in [Schiebinger et al., 2017].”

As correctly noted by the authors of ref. (Peyré and Cuturi 2019), Schiebinger et al. (Cell 2019) already make use of unbalanced OT to account for cellular growth and death - another work that is cited by Cang et al. in the same sentence. Another work the authors cite in the same sentence, ref. (Chizat et al. 2018) introduced an efficient algorithm to solve unbalanced OT problems. For a simple illustration of how unbalanced OT can be applied to discrete measures that are not probability distributions (don’t sum to one), see https://pythonot.github.io/auto_examples/unbalanced-partial/plot_UOT_1D.html# which is taken from the docs of Python Optimal Transport (POT), the most common framework for Optimal Transport analysis in Python. Hence, the motivation outlined by the authors here to develop a new computational method is factually incorrect - the problem of computing a coupling between discrete measures that are not probability distributions is solved by unbalanced optimal transport, as outlined in many of the works the authors cite themselves.

This problem continues in the next paragraph, line 82. The authors write

“To address these two problems, we present a novel method called collective optimal transport, which is capable of preserving the original units without the need for
normalization, enforcing spatial range limits of signaling, and handling the cases of multiple competing species. “

From the previous paragraph, I conclude that the two problems the authors set out to tackle are
OT cannot be applied to input measures which are not probability distributions

Competition among ligand and receptor species, which is not taken into account by existing CCC methods which perform pairwise comparisons.

As the authors themselves explain, OT solves the second problem. The first problem, however, is invalid since unbalanced optimal transport, an established framework with efficient computational algorithms already existing, solves this problem. Thus, from this description, I do not see the need to develop a new OT extension. Moreover, since an efficient solution to this problem exists which is used by others both within (Schiebinger et al. 2019) and outside the single cell field, I think the authors should consider basing their method on unbalanced optimal transport rather than developing their own algorithm. The advantages of using unbalanced optimal transport are clear: it's an established method with mathematical guarantees and efficient, numerically robust and scalable algorithms to solve it (Peyré and Cuturi 2019; Chizat et al. 2018).

Results section, line 112, the authors write:

"This method has two important properties: 1) it couples multi-species distributions as opposed to traditional optimal transport that couple single-species distributions and 2) it allows infinity entries in the cost matrix to enforce spatial distance limits of CCC to avoid connecting cells that are too far apart spatially, while traditional optimal transport can only deal with finite entries"

I have two problems with this statement. First, their method description changed significantly from what it was in the introduction, see my comment above. Second, I'm again unsure whether there's a need for a new computational method here. The authors stress that their method has two important properties:

It couples several species at once, instead of just a single, as in classical OT
It allows for infinite entries in the transition matrix

From my understanding, problem 1 is classically solved using multimarginal OT, see Chapter 10.1 in ref. (Peyré and Cuturi 2019) and the following example from POT: https://pythonot.github.io/auto_examples/domain-adaptation/plot_otda_jcpot.html# Problem 2 is classically solved using partial OT, see ref. (Figalli 2010) and https://pythonot.github.io/auto_examples/unbalanced-partial/plot_partial_wass_and_gromov.html# from POT. If that is not the case, i.e. if there is some reason that prevents the authors from using these standard techniques, they should clearly state this and explain why this is the case.

In their methods supplement, the authors give more details and show how their problem can be broken down to equation Suppl(3), which to me, this looks like a multimarginal unbalanced OT problem, and connections to these earlier methods need to be made obvious and cited accordingly. IMHO, the

authors should pay special attention to infinite entries in the cost matrix, which could also be addressed using partial OT, and again, connections need to be made clear. Also, the algorithms they derive reduce to Sinkhorn iterations (the derivations are very nice), which yet again makes me wonder whether this is just a version of unbalanced OT. This problem is also apparent in the discussion, starting in line 413, where the authors write:

“While traditional optimal transport is powerful at integrating one pair of datasets, collective optimal transport can be powerful for multi-omics data integration as it couples multiple datasets at the same time, so that coupling of one dataset pair provides information about integration of other datasets. Moreover, the ability to preseve the original units of mass distributions allows collective optimal transport to better integrate unbalanced datasets, which is a common challenge in data integration, due to different limitations of different technologies.”

Again, this gives the impression that there exist no established OT extensions that can deal with multiple marginals or an unbalanced setting, which is not true. These extensions exist, they are widely used and implemented in standard python packages. The authors should avoid giving the impression of “reinventing the wheel” by making connections of their method to previous approaches explicitly, citing these appropriately and clearly stating why there is a need for a new OT extension in light of existing approaches.

What’s the difference between supervised optimal transport (SOT), collective optimal transport and COMMOT? The authors use all three terms and it’s unclear what their relationship is. This needs to be made explicit, it’s currently confusing.

Fig. 2, the authors should benchmark their method against other methods that can infer CCC, currently, they only compare to standard OT, applied independently to each ligand-receptor species pair. They should compare against both methods that make use of spatial information (like Squidpy, see the efficient cellphone db reimplementation) and those that do not when inferring CCC (the authors cite many of these methods in the introduction). The simulation example should be used to demonstrate how COMMOT improves over previous approaches, and which limitations to the approach exist.

Fig. 3 and corresponding text, similar to my above comment, it’s not clear from text or figure whether the recovered signalling direction could not have been equally well recovered using existing approaches. The authors could place more emphasis on highlighting shortcomings of previous approaches, and how their method improves upon them, not just in terms of theoretical analysis, but also in practical applications. If, for example, other computational approaches cannot be used to find partial DE genes (Fig. 3f), then this needs to be stated and shown in an example. The advantages of COMMOT over previous approaches need to be made much more concrete.

Fig. 3, I don’t find the evaluation of identified genes convincing. First, it would be good to see that these genes cannot be inferred equally well with existing, simpler approaches, see above comment. Second, the metric in Fig. 3e is not convincing: simply looking at the percentage of known genes identified does not take false positives into account, i.e. genes identified by COMMOT’s differential expression test which are known not to play a role downstream of DPP signalling.

Methods section, starting line 434, the authors write:

“The collective optimal transport results in a large-scale optimization problem for which efficient algorithms are developed.”

While it is in fact true that the authors reduce their algorithm to Sinkhorn iterations, they do not demonstrate that their algorithm is efficient, neigher in theory nor practice. In fact, standard sinkhorn iterations are known to scale quadratically in sample number (Peyré and Cuturi 2019), which, by modern ML standards, is not considered to be efficient. This does not rule out using OT, in fact, OT is used in many applications in data science, however, for the Sinkhorn updates developed in the methods supplementary for the L1 and L2 cases, it should be stated explicitly how compute time and memory complexity scale with the number of cells, ligand and receptor species to evaluate scalability of the proposed model. Ideally, this would also be demonstrated in a practical example where peak memory usage and compute time are recorded for test problems of increasing size (cell number, ligand/receptor pairs).

Code: the code is well-organized and documented. However, it’s unclear to me why the authors have to re-implement the sinkhorn algorithm since efficient implementations exist, e.g. in POT. Of course, sinkhorn is very simple to implement, nevertheless, by making use of existing implementations, the authors could benefit from more recent accelerations of sinkhorn, see the many solvers implemented in POT: https://pythonot.github.io/all.html Further, making use of existing OT packages as building blogs will likely result in fewer bugs since an entire community uses POT and fixes bugs. Furthermore, it’s unclear to me why the authors re-implement KNN graph construction in `_clustering.py`. They should make use of existing efficient implementations, such as `HNSW` or `pynndescent`. This is a general point, wherever efficient solutions are available in the community already, they should be used, rather than reinventing the wheel. Especially when it comes to KNN construction - this is a very well researched area and highly optimized solutions exist.

Code: there are no tests. For a software to be published in a high impact journal like Nature methods, I expect to see a minimal number of unit & regression tests that ensure the software is stable, mostly bug-free and can be maintained in the future to serve the community. Please include tests and set up CI.

Minor comments

In the abstract, I find the description of datasets used for application confusing. The authors write “We validate the method with simulation data and one spatially annotated scRNA-seq dataset. We show that COMMOT effectively infers spatial CCC using datasets by three popular spatial transcriptomic technologies.” After reading this, it’s unclear to me whether the authors used one or three datasets to apply their method. This should be made much more clear in the abstract, it’s essential information.

Introduction, line 41, the authors write “The majority of these methods rely on the expression levels of ligand and receptor pairs and predefined empirical formulas.” Unclear to me what exactly they mean by “predefined empirical formulas”. In the end, mathematical models define a way to process inputs to outputs and at some stage, they all contain “formulas”, don’t they?

Intro, line 55, the authors write “Spatial transcriptomics technologies provide distance information among cells.” Many current technologies do not achieve single-cell resolution, e.g. visium, so I don’t think this claim is formulated a bit too broadly.

Same paragraph as above, the authors open with this sentence about spatial transcriptomics technologies but proceed to introduce computational methods; this is confusing - I expect to learn about spatial technologies after this opening sentence.

There are spelling and grammar mistakes in the text. I will just give an example from the intro, line 65, the authors write “ there is competition between cells and therefore systematically examine CCC among all cells requires a global perspective”.

Introduction, line 93, the authors write “infers CCC in spatial transcriptomics data or spatially annotated scRNA-seq”. They used these terms a few times before, without ever explaining what the difference between them is. The terms need to be defined before they are used. After reading the entire text, I understand the latter refers to spatially imputed data, however, this could be explained earlier on.

Introduction, line 95, the authors write “identifies potential downstream impacts on gene expressions by CCC using machine learning models” - what are these “machine learning models”? Inflationary usage of the term “machine learning model” should be avoided, rather state which model was used here (Random Forests, see Line 353)

Line 304, the authors write “In this case, the CCC-induced clusters are mixed in space suggesting that the CCC activity is similar across the different tissue regions.” - is that expected in this experimental system? Is there anything known about this in the literature? At the moment, it’s unclear whether this result is biologically meaningful

Line 341, why is tradeSeq employed here and not in earlier analysis to identify DE genes as a function of signal reception? Should be motivated somehow.

Fig. 4c,d, it’s unclear to me after looking at the panels, reading the legend and the corresponding text to what extent their computational prediction was validated using the stainings. This explanation is given in the discussion, starting line 391. Consider moving this.

References

Chizat, L., G. Peyré, B. Schmitzer, and F. X. Vialard. 2018. “Scaling Algorithms for Unbalanced Optimal Transport Problems.” Mathematics of Computation. https://www.ams.org/mcom/0000-000-00/S0025-5718-2018-03303-8/.
Figalli, Alessio. 2010. “The Optimal Partial Transport Problem.” Archive for Rational Mechanics and Analysis 195 (2): 533–60.
Peyré, Gabriel, and Marco Cuturi. 2019. “Computational Optimal Transport: With Applications to Data Science.” Foundations and Trends® in Machine Learning 11 (5-6): 355–607.
Schiebinger, Geoffrey, Jian Shu, Marcin Tabaka, Brian Cleary, Vidya Subramanian, Aryeh Solomon, Joshua Gould, et al. 2019. “Optimal-Transport Analysis of Single-Cell Gene Expression Identifies Developmental Trajectories in Reprogramming.” Cell 176 (6): 1517.

Author Rebuttal to Initial comments

Reviewer #1:

Cang et al present COMMOT, a computational method used on optimal transport (OT) to study cell-cell communication from spatially resolved transcriptomics data.

This type of data is becoming increasingly available and it enables to look at cell-communication with the additional insight of where cells are located, and hence computational methods that leverage this knowledge are timely. COMMOT presents a number of novel features. The method seems mathematically sound. The authors introduce a variant of optimal transport, called collective optimal transport (COT) which considers multiple source and target species simultaneously. They provide two iterative algorithms based on stabilized Sinkhorn iterations to find near optimal solutions. COMMOT is available as a package for use by the community.

While we welcome COMMOT to the palette of methods in the field, we have the following questions and concerns:

Major points

1 - The authors make a strong case of how their formulation takes into account the absolute values of ligands and receptors instead of normalised values, as well as the competition for receptor by ligands. However, these are phenomena that occur at the level of proteins (ligands and receptors are protein, not RNA molecules), and hence is not clear what a real impact this has at the level or RNA, given the limited correlation of proteins and RNA, plus all modifications that happen to proteins, etc.

Response: We agree with the reviewer on this critical point, which is probably true for nearly all current computational methods for inferring cell-cell communication based on scRNA-seq or spatial transcriptomics data. While RNA abundance may have limited correlation with the level of proteins, many studies, including ours, on cell-cell communication using RNA abundancy information show that it is a valuable way of generating hypothesis-driven questions when protein information is lacking. Such methods have been broadly used in scRNA-seq data analysis. For example, the popular CellPhoneDB and CellChat methods have led to important biological insights. In principle, methods on inferring cell-cell communication using RNA information can be applied to single-cell or spatial protein measurements as they become available.

Although there is a lack of the ground-truth of protein-level cell-cell communication data to benchmark our method, we have added various assessments, including new experimental data, in the revised manuscript in addition to the ones in the original manuscript. These assessments are summarized here:

1) (**New experimental validation of RNA and protein abundance**) In the revised manuscript, we measured the spatial distributions of RNA using RNASscope and the corresponding protein via immunostaining for two pairs of ligand and receptors (GAS6-TYRO3, PROS1-TYRO3) for the human epidermis system. The pairs with high inferred CCC activity are chosen to be examined. The experimentally determined spatial distributions of RNA and protein agree well, and both support the inferred CCC (new Fig. 3b-e). The new result has been added in Paragraph 2 of section "The roles of CCC in human epidermal development". The new experimental result is added to new Fig. 3d,e. The related part in new Fig. 3 is shown below:

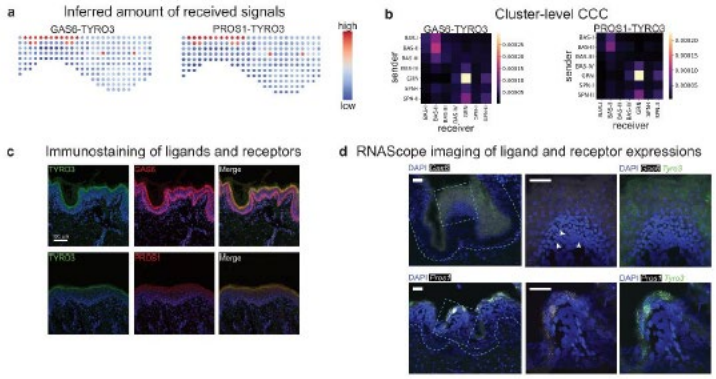

Figure 3 (partial) ***a-b*** *The inferred amount of received signals of two example ligand-receptor pairs, GAS6-TYRO3 and PROS1-TYRO3 at cell level and cluster level.* ***c*** *Immunostaining of proteins for GAS6, TYRO3, and PROS1.* ***d*** *RNAScope examination of GAS6, TYRO3, and PROS1.*

2) (**Findings consistent with prior knowledge**) In the original manuscript, we mainly analyzed two well-studied classical signaling pathways, WNT and TGF-β pathways, on the seqFISH+ data of mouse somatosensory cortex (sscortex), and Visium data of mouse brain and found that inference results are supported by biological knowledge. In the seqFISH+ example, we identified known downstream genes of Wnt signaling pathway and

in the Visium data, the recovered signaling directions of WNT and TGF-β pathways were consistent with prior knowledge.

In the revised manuscript, we have used a more recent and comprehensive ligand-receptor database (CellChatDB) and analyzed all available pathways. We have added two datasets, a MERFISH data on mouse hypothalamic preoptic region and a STARmap data on mouse placenta. a) In the MERFISH example, we consistently found OXT signaling patterns among excitatory and inhibitory neurons recapitulating known biological functions. b) In the STARmap data, we found opposite signaling directions between MK and IGF pathways indicating a known feedback loop between the two. c) In the Visium mouse brain data, after switching to the new LR database, we also found observations of signaling directions of PSAP and FGF pathways consistent with biological knowledge. While we are not able to survey the literature for all analyzed pathways, we have generated the signaling directions and cluster-level signaling for all pathways in the reproducible code for readers to assess. The analysis of MERFISH and STARmap data is added as new Fig. 4 in the revised manuscript.

3) (**Robustness assessed based on subsampling**) In the original manuscript, using a well-studied system of drosophila embryo, we demonstrated the robustness of COMMOT by showing the consistency between cell-cell communication inference results from subsampled data and full data.
4) (**Correlation to known target genes**) In the revised manuscript, we added an analysis of the correlation between the inferred cell-cell communication activity and known target genes using the downstream gene database (Baruzzo, G., Cesaro, G., & Di Camillo, B. (2022). Identify, quantify and characterize cellular communication from single-cell RNA sequencing data with scSeqComm. Bioinformatics.) The analyses were conducted on three datasets that have transcriptome or near transcriptome coverage of genes: Visium data on human breast cancer and mouse brain, and seqFISH+ data on mouse sscortex. We demonstrate the correlations between the inferred cell-cell communication activity and the expression of known target genes. This new analysis result is discussed in Paragraph 4 of section "Robust identification of spatial signaling direction and downstream targets" in the revised manuscript and new Supplementary Figs. 20 and 21.
5) (**Comparison with other methods**) In the revised manuscript, we have added comparisons to two other methods: CellChat for inferring CCC from scRNA-seq and Giotto for inferring CCC from ST data. Compared to CellChat, we show that COMMOT identifies CCC that are spatially significant but lowly ranked when all cluster pairs are considered

as in CellChat. Compared to Giotto, we show that COMMOT provides interpretable results and identifies signaling through secreted ligands over spatial range beyond cell-cell contact. These comparisons demonstrate the unique properties of COMMOT and clarify for users about what types of CCC are expected. The new comparison analysis is added as new Supplementary Figs. 22-25 and discussed in Paragraph 5 of section "Robust identification of spatial signaling direction and downstream targets". Related to item (3) above, we also compared the three methods in terms of correlation between inferred CCC and expression of known target genes where a stronger correlation is found for COMMOT (new Supplementary Fig. 21).

6) (**Continuous change over slices**) In the revised manuscript, the newly added MERFISH data is composed of 12 stacked slices (new Fig. 4a). This allows us to evaluate the continuity of inferred CCC activity across adjacent slices based on the assumption that CCC activities are more likely to change gradually across the spatially adjacent slices. We show that both gradual changes of the inferred signaling directions and cluster-level CCC are observed (new Fig. 4b,c and new Supplementary Figs. 16-17).
7) (**Label and spatial permutations**) In the original manuscript, we performed label permutations for deriving the significance of the identified cluster-level CCC. In the revised manuscript (also related to the reviewer's comment 5.1), we have also included spatial permutations which are more directly related to the spatial significance. These different permutations will be useful for users to prioritize the inferred CCC. The spatial permutation is added as function cluster_communication_spatial_permutation to the COMMOT package and the description has been added to section "Cluster level CCC" of Methods.

1.1. Could authors not apply their method to spatially resolved protein data?

Response: COMMOT can be used directly for protein data if the spatially resolved protein data is available. In our study of human skin, we've provided a set of protein staining data to partially test our method based on scRNA-seq data. We first integrated scRNA-seq data with spatial data to obtain "in silico" ST data. We then analyzed CCC in this data using COMMOT and identified the CCC induced DE genes. We found that the staining of DE genes agrees with our findings. For example, WNT signaling was found to be active in the basal layer (new Fig. 3f) and KRT5 was identified as a positive DE gene of the inferred WNT signaling (new Fig. 3g). In the staining data, KRT5 was identified in the basal layer colocalized with the region of high WNT signaling activity (new Fig. 3h). We have also conducted immunostaining of ligand and receptor proteins for several LR pairs which support our CCC inference results (new Fig. 3d).

We have searched current publicly available datasets and found no measured known ligand-receptor pairs (from a comprehensive ligand-receptor database, CellChatDB that we use in the revision). We anticipate spatial proteomics data to be available soon and COMMOT can be used directly to analyze those datasets.

To better address this point in the revision, we added a point in the Discussion:

"Of course, false positives in our inferred CCC are possible as the spatial transcriptomics data may not directly represent protein abundancy and our method cannot capture some CCC mechanisms such as phosphorylation. The reliability of CCC prediction is expected to be improved when the emerging spatial proteomics data is used with COMMOT."

2- The simulated data is interesting and a helpful control, but not a 'validation' as it is labeled in Figure 2. The model uses the same concepts as COMMOT, based on modeling diffusion as a function of abundance; so it is reassuring but not surprising that COMMOT works there. Perhaps should be a supplementary note.

Also related to the previous point, it is based on in silicon data that represents proteins (ligands and receptors), not transcripts.

Response: We agree that the spatiotemporal PDE model uses the same concepts as COMMOT and therefore COMMOT is expected to work here. Such spatiotemporal models have been widely used as an effective way to simulate ligand-receptor interactions for testing biological hypotheses (Lander, A. (2011). Pattern, growth, and control. Cell, 144(6), 955-969.). We agree that "validation" is inappropriate for such a case; however, it's still a valuable benchmark for the method. In the revision, we replaced the word "validation" with "comparison to simulated data" as often done for method testing when ground-truth is lacking. We would be willing to move this section into a supplementary note if the reviewer deems it necessary.

This simulation assumes that protein abundancy is proportional to RNA abundancy. We thus directly compare the COMMOT inference to the simulation results. In addition to this control, in the revision, we have added a systematic evaluation of the correlation between inferred signaling and activity of downstream genes to demonstrate the method and compare with other methods (details about this evaluation are discussed in the reply to point 5).

3 - Also, the data generated with SpaOTsc is built generating OT as well, so is in that senes a bit redundant: SpaOTsc uses the assumption that cell co-localize with similarity of expression, that also implies that structure is driven by diffusion. Actually, why generating from suspension data spatial data to try COMMOT, when there are so many spatial data sets?

Response: We thank the reviewer for pointing this out. In our method, the integration of scRNA-seq and spatial data is still mainly based on the expression similarity between cells in scRNA-seq data and spots in spatial data. The assumption of co-localization of cells with similar expression profiles is only used as a secondary tool to potentially improve the integration if it's needed. The quality of the two integrated dataset is justified. Specifically, the drosophila data has been extensively analyzed and tested in our previous works and the integrated human skin data agrees well with prior knowledge of the spatial arrangement of cell types.

Despite of availability of various spatial transcriptomics data, scRNA-seq data still have superior sequencing depth and better gene expression detection. Currently, scRNA-seq data and spatial data complement each other so including examples of such integrated data would be helpful.

To analyze more original spatial transcriptomics datasets as suggested by the reviewer, in the revision we have added two more examples of MERFISH and STARmap datasets where we also found the inferred signaling activity to be consistent with biological knowledge. Furthermore, the MERFISH dataset contains a sequence of adjacent two-dimensional tissue slices, and we also assessed the robustness of COMMOT by comparing the inferred cell-cell communication across the spatially adjacent slices. The new analysis results have been added as new Fig. 4 and described in Paragraphs 1 and 2 in the section "Signaling analysis in high spatial resolution ST data".

4- - Computational efficiency and scalability of the proposed algorithms: It is not clear from their experiments how the algorithms perform in terms of computation time, and their scalability to large instances should be investigated. The model with l1 norm penalty on the slacks can be easily cast as a linear program (transportation problem). This formulation permits setting the coupling pairs with infinity cost to zero, and thus removing them from the LP. This results in an LP with significantly fewer variables, and possibly easy to solve, either using a solver or using tailored combinatorial algorithms. A comparison in terms of scalability of these formulation would be helpful.

Response: We thank the reviewer for this insightful suggestion. In the current implementation, the sparsity of the transport plan has been used such that both the cost matrix and the transport plan are sparse matrices. With the l_1 norm, this formulation can be cast into a linear programming problem. This is still a large-scale problem due to the simultaneous incorporation of all ligand-receptor pairs. In the traditional optimal transport, the original problem can be solved as a linear programming problem; however, with significant speed acceleration using an approximation with an entropy regularization term to move the optimal solution to the interior of feasible sets. We follow the same strategy here to improve computational efficiency by introducing the entropy regularization term.

In the revision, we have tested the computational efficiency of the CCC inference function of COMMOT (new Supplementary Fig. 26). We found that the running time scales linearly to the number of nonzero elements in the CCC network (the potential nonzero elements in the transport plan) and the method can be run in a reasonable time for typical ST data. This new efficiency and scalability result has been added in the last paragraph of the Results section in the revised manuscript.

5 - it is very challenging to know if this (or any other related method) is 'working', as we lack true experimental ground truths of cellular communication. Authors should clarify or soften statements such as 'successfully applied'. When is successfully applied? When the results seem to agree with our current knowledge from literature? That is very hard to assess and prone to biases.

Response: We agree with the reviewer that it is really challenging to directly verify the inferred cell-cell communications. In the revision, we have removed statements such as "successfully applied" to be more precise about what we've done on the data analysis. We have also added this point in Discussion to explicitly state the caveat of our method in Paragraph 2 of Discussion.

Following the reviewer's suggestion, in the revised manuscript we have added different permutation tests and performed a systematic evaluation of the method and comparison to other methods on a prediction task. The details of these additions are listed below when replying to Point 5.1.

5.1. - While not real validations, helpful analysis that the authors should perform include diverse tests of the statistical significance of their solutions. For example, by random permutation of cells

in each of the sample(slide). And if possible also consider some 'prediction task' and show how COMMOT helps to predict unseen data.

Response: Thank you for the suggestion, In the revision, we have added label permutations and spatial permutations to evaluate the significance of cluster-level CCC. The permutation tests are described in Methods - Cluster level CCC.

Utilizing a recent comprehensive database of known downstream target genes of various ligand-receptor pairs (the databases organized in the package scSeqComm), we evaluate the performance of the CCC inference using the correlation between the expression of known downstream genes and inferred CCC activity:

1) At individual-spot level, Spearman's correlation coefficient is computed for each ligand-receptor pair between the level of received signal and the average expression of the known downstream genes.
2) At cluster-level, we quantify the level of received signal by the averages among the spots of the clusters. The activity of the downstream genes of a ligand-receptor pair is quantified as the percentage of significant positive DE genes of a cluster.

It is shown that the inferred CCC activity is correlated with expression of known downstream genes (new Supplementary Fig. 20).

At the cluster level, we also compare COMMOT with two popular existing methods that infer cluster-level cell-cell communication networks, CellChat (Jin, Suoqin, et al. "Inference and analysis of cell-cell communication using CellChat." Nature communications 12.1 (2021): 1-20.) and Giotto (Dries, R., Zhu, Q., Dong, R., Eng, C. H. L., Li, H., Liu, K., ... & Yuan, G. C. (2021). Giotto: a toolbox for integrative analysis and visualization of spatial expression data. Genome

biology, 22(1), 1-31.) which are designed for scRNA-seq data and spatial transcriptomics data, respectively. In this comparison, COMMOT achieves stronger correlation than the other methods.

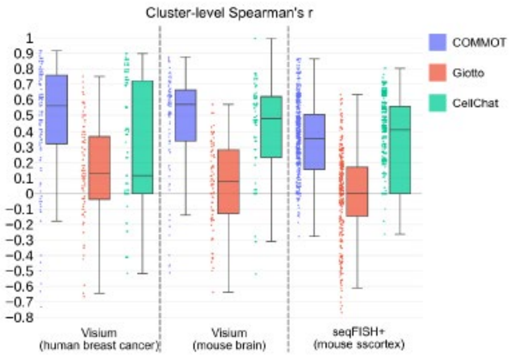

Supplementary Figure 21. Cluster-level correlation between inferred signaling and activity of known downstream genes. Each dot represents a ligand-receptor pair. The activity of known downstream genes of a ligand-receptor pair is quantified for each cell cluster as the percentage of positive significant differentially expressed ones (p-value < 0.05) in that cluster. The Spearman's correlation coefficients are computed between the amount of received signal and the downstream gene activity. For COMMOT, the amount of received signal is quantified by the average received signal of the cells in that cluster. For Giotto and CellChat that output a cluster-level signaling table, the maximum CCC score with each cluster as the receiver is used to quantify the amount of received signal.

Only the Visium and seqFISH+ datasets are used here due to their good coverage of genes, which is important when performing systematic analyses of downstream genes.

The new analysis results have been added as Paragraph 4 in Results "Robust identification of spatial signaling direction and downstream targets" and new Supplementary Figs. 20-21.

Minor points

1- Authors take the ligand-receptor pairs from Fantom database, which is relatively old. there are many others after that one and they provide different results, so authors should explore this.

Response: Thank you for the suggestion. In the revision, we have changed it to more updated and comprehensive databases in "CellChat" that contain manually curated ligand-receptor pairs of more than 2000 pairs for both mouse and human. CellChat (the paper and R package) was published in 2021 and has been highly used and cited.

2 - with the random forest model, there is a danger of diluting the importance over all correlated variables because they are also correlated with each other by transition.

Response: Thank you for pointing this out. To analyze how CCC impacts a target gene using random forest models, we have two options: 1) using the amounts of received signals through all the LR pairs of interest as features in a single random forest model ("tree_combined" set to True in the function "commot.tl.communication_impact") or 2) building an individual model for each LR pair. In those models, the "background genes" are also used as additional features to account for possible intra-cellular regulations of the target gene. In the first option, there is potentially dilution between the importance of different LR pairs; however, this problem can be avoided by the second option. For the dilution between the received signal and background genes, we can filter the LR pairs with unique impacts on the target genes, though this can also be weakened by setting the option "bg_genes" to a small number.

In the revision, we have added a description of how to choose these parameters for different analysis goals in the function's documentation and added a description in Methods - Downstream gene analysis which reads as the following:

"The inclusion of intracellular highly correlated genes as input features emphasizes the amount of information of potential target genes explained by inferred CCC which is unlikely explained by intracellular interactions. If such dilution of importance is not preferred, the users could choose a smaller number of highly correlated genes as input features."

3 - Notational consistency: the mathematical notations in the body of the paper and those employed in the supplementary appendix are inconsistent in some cases. For instance, n_s is used for referring to points (cells/spots) in the main body of the paper, whereas in the appendix, it seems that the notation is n_p, while n_s is used for source species. These inconsistencies become confusing in some points, making it harder to follow than necessary.

Response: In the revision, we have revised the supplementary file to ensure the notations consistent with the content of the main text.

Reviewer #2:

Remarks to the Author:

In this manuscript, the authors developed a novel computational framework COMMOT to infer cell-cell communications based on optimal transport. COMMOT is capable of 1) preserving the original units without the need for normalization, 2) enforcing spatial range limits of signaling, and 3) handling the cases of multiple competing species. Overall, the method is novel in concept. It is also of immediate interest to the field. However, several issues must be addressed before publication.

At a high level, most of the commentary is around some figures lacking quantitative support and a lack of robust comparisons against many baselines and ground truth.

Major points

1. Why do authors only compare with pairwise traditional optimal transport?

Response: Thank you for bringing up this point. In the revision, we have added comparisons to two other existing methods for CCC inference and several variants of optimal transport:

1) Comparisons to unbalanced optimal transport and partial optimal transport have been added. COMMOT is shown to outperform the other OT variants on the PDE simulation data. (Paragraph 4 of section "Accurate reconstruction of simulated spatial CCC", new Supplementary Figs. 7-9). The new Supplementary Figs. 7-9 showing the property differences among these methods and the performance differences on the simulated data are shown below

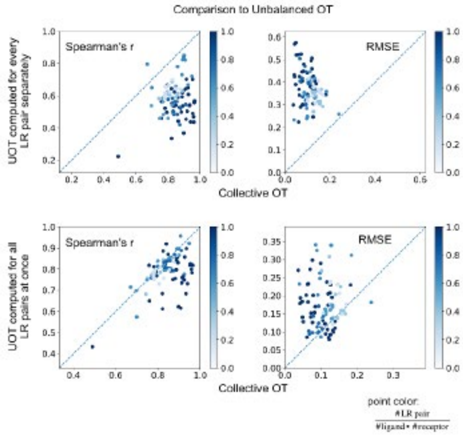

Supplementary Figure7. Comparison with unbalanced optimal transport that examines each ligand-receptor pair separately (top) or all pairs together (bottom). All analyses use the same spatial cutoff such that the transport cost is set to infinity if the distance between a location pair exceeds the distance cutoff. The optimal transport analysis results are evaluated by comparing the inferred amount of received signal to those obtained by simulation using Spearman's correlation coefficient and root-mean-squared error.

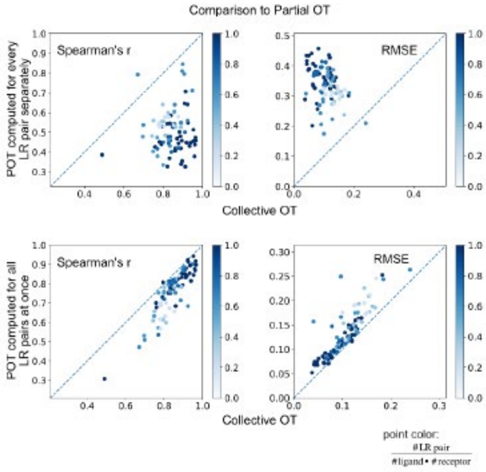

Supplementary Figure 8. Comparison with partial optimal that examines each ligand-receptor pair separately (top) or all pairs together (bottom). All analyses use the same spatial cutoff such that the transport cost is set to infinity

if the distance between a location pair exceeds the distance cutoff. The optimal transport analysis results are evaluated by comparing the inferred amount of received signal to those obtained by simulation using Spearman's correlation coefficient and root-mean-squared error.

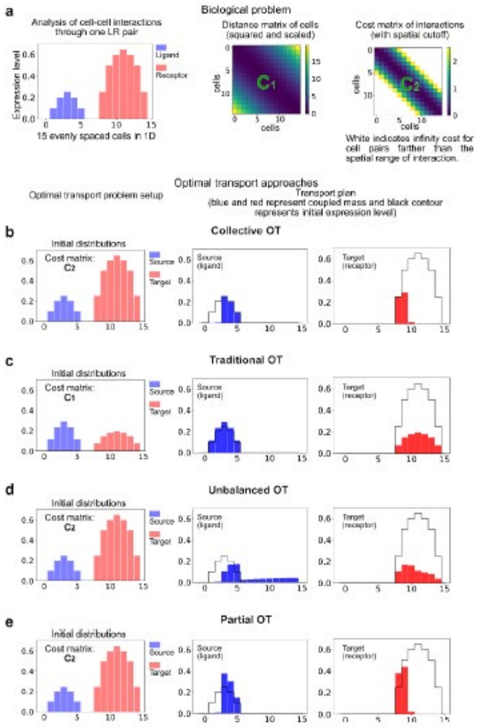

Supplementary Figure 9. Illustration of differences among several optimal transport variants. ***a.*** *The problem of fifteen cells placed one unit apart in 1D through one ligand-receptor pair with a spatial range cutoff of five units.* ***b-e.*** *The input distributions and cost matrices of each optimal transport approach and the marginal distributions of the optimal transport plans. All three variants (collective OT, unbalanced OT, and partial OT) can take the initial unnormalized distributions and the cost matrix with infinity entries. However, unbalanced OT and partial OT result in artificial extra amount of ligand and receptor exceeding their expression levels. Traditional OT also induces artificial expressions due to normalization and cannot enforce the spatial range of signaling. Collective OT controls the coupled mass by the initial distributions and enforces the spatial range of signaling.*

2) Comparisons to two existing CCC inference tools, CellChat and Giotto, on method properties are added. a) Compared to CellChat, COMMOT filters out communications between clusters that are spatially far apart and identifies localized CCC events that are overlooked by CellChat. (Paragraph 5 of section "Robust identification of spatial signaling direction and downstream targets", new Supplementary Figs. 22-23). b) Compared to Giotto, COMMOT treats spatial distance as a transport cost while Giotto creates contact graph with distance cutoff. As a result, Giotto prioritizes contact based CCC and COMMOT is suitable for model diffusive ligands. (Paragraph 5 of section "Robust identification of spatial signaling direction and downstream targets", new Supplementary Figs. 24-25). Two example new Supplementary Figs. 23 and 25 demonstrating the differences are shown below.

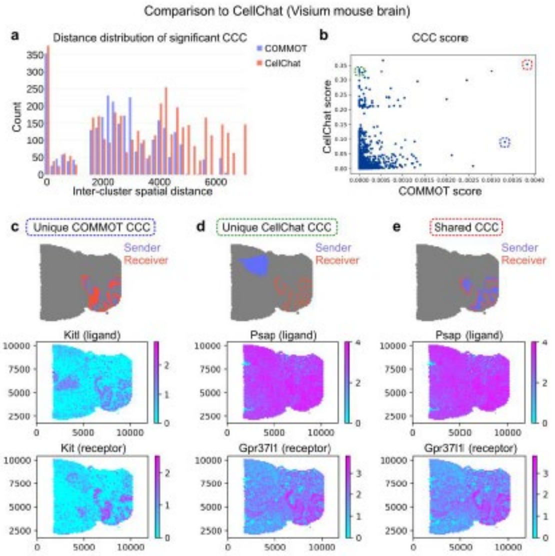

*Supplementary Figure 23. Comparison with CellChat on a Visium mouse brain dataset. **a** The distribution of inter-cluster distances (distance between geometric center of the clusters) of the significant CCC cluster pairs. **b** Each point represents a ligand-receptor pair and a cluster pair. **c-e** Example significant cluster-level CCC that are uniquely identified by COMMOT and CellChat, and by both methods.*

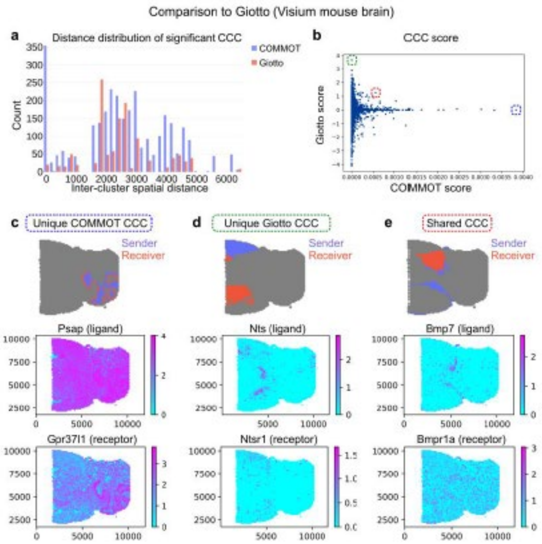

*Supplementary Figure 25. Comparison with Giotto on a Visium mouse brain dataset. **a** The distribution of inter-cluster distances (distance between geometric center of the clusters) of the significant CCC cluster pairs. **b** Each point represent a ligand-receptor pair and a cluster pair. **c-e** Example significant cluster-level CCC that are uniquely identified by COMMOT and Giotto, and by both methods.*

3) A comparison to CellChat and Giotto on downstream analysis has been added. Using a database of known target genes of each ligand-receptor pair (provided by scSeqComm package), we evaluate the correlation between inferred signaling activity and the activity of known target genes for each ligand-receptor pair. This systematic analysis shows that COMMOT achieves a higher correlation compared to the other two methods. The result is added in Paragraph 4 of section "Robust identification of spatial signaling direction and downstream targets" and new Supplementary Fig. 21.

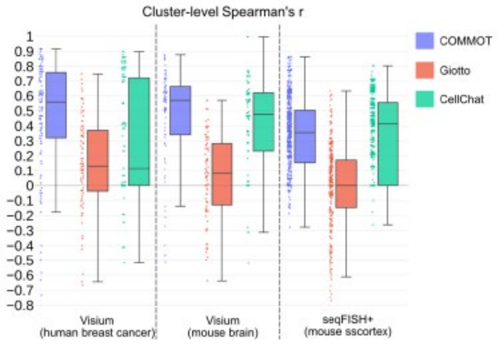

Supplementary Figure 21. Cluster-level correlation between inferred signaling and activity of known downstream genes. Each dot represents a ligand-receptor pair. The activity of known downstream genes of a ligand-receptor pair is quantified for each cell cluster as the percentage of positive significant differentially expressed ones (p-value < 0.05) in that cluster. The Spearman's correlation coefficients are computed between the amount of received signal and the downstream gene activity. For COMMOT, the amount of received signal is quantified by the average received signal of the cells in that cluster. For Giotto and CellChat that output a cluster-level signaling table, the maximum CCC score with each cluster as the receiver is used to quantify the amount of received signal.

2. Can the failure modes of not doing normalization or considering spatial information be demonstrated with your method? By normalizing the distributions and showing inferior results? By making an identity distance matrix and showing false positives?

Response: In the revision, we have added several demonstrations showing the advantages of 1) using spatial information and 2) without normalizing the expression levels to probability distributions. The additions are summarized as follows.

1) An illustration of the failure mode of normalizing expression levels to probability distributions is given in new Supplementary Fig. 5 shown below. When the total amounts of ligand and receptor expression levels are significantly different, normalizing them into probability distributions (which is required to apply traditional optimal transport) would result in false positives.

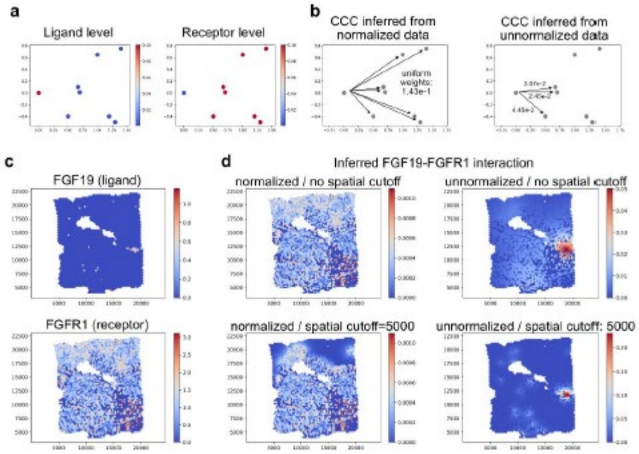

*Supplementary Figure 5. Comparison of whether to normalize expression to probability distributions. **a** An example with 8 cells where 1 cell expresses the ligand gene and the other 7 cells express the receptor gene. **b** The inferred cell-cell communication (CCC) with or without normalizing the expression levels to probability distributions. **c** An example ligand-receptor pair in a Visium human breast cancer data. **d** The inferred spatial distribution of received signals through the ligand-receptor pair FGF19-FGFR1 with or without normalization to probability distribution and with or without a spatial distance cutoff.*

2) We also show the advantage of collective optimal transport using expression levels as non-probability distributions in the comparisons to traditional optimal transport which requires normalizing expression levels to probability distributions (new Supplementary Fig. 9).
3) An illustration of the failure mode of not considering spatial information is given in new Supplementary Fig. 6 shown below. Incorporating spatial information will prioritize interactions among cells that are spatially close, and a spatial distance cutoff will further constrain the interactions in localized regions. The spatial distance cutoff is discussed in detail in the reply to comment 6.

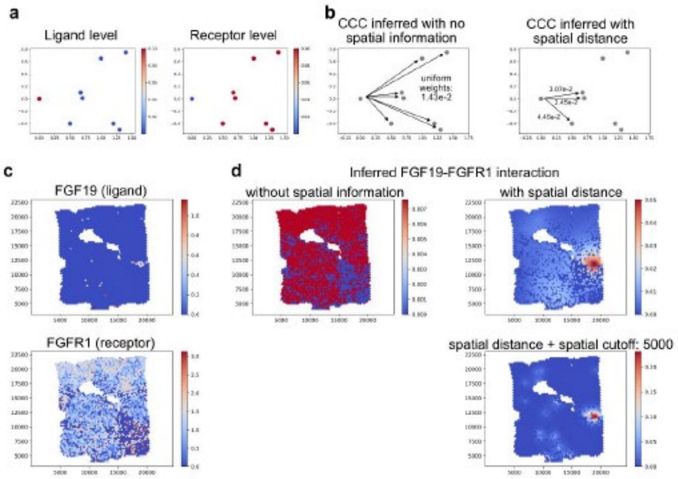

Supplementary Figure 6. Comparison of whether to incorporate spatial distance information. ***a*** *An example with 8 cells where 1 cell expresses the ligand gene and the other 7 cells express the receptor gene.* ***b*** *The inferred cell-cell communication (CCC) with or without normalizing the expression levels to probability distributions. Both results are from the spatial expression levels without normalizing to probability distributions.* ***c*** *An example ligand-receptor pair in a Visium human breast cancer data.* ***d*** *The inferred spatial distribution of received signals through the ligand-receptor pair FGF19-FGFR1 with or without incorporating spatial information and if spatial information is used, with or without a spatial distance cutoff.*

4) The advantage of incorporating spatial information is further demonstrated in comparison to another existing method, CellChat which infers cell-cell communication in single-cell data without spatial information. As expected, not using spatial information might lead to false positives (communications between clusters that are too spatially distant) (new Supplementary Figs. 22-23). We also show that the CCC activities inferred by COMMOT have higher correlations to the expression levels of known target genes of the ligand-receptor pairs (new Supplementary Fig. 21).

In the last paragraph of the section Accurate reconstruction of simulated spatial CCC, a description is added:

"The ability to handle non-probability distribution is especially useful when the total levels of ligand and receptor expressions are significantly different (Supplementary Fig. 5) and incorporating

spatial information helps prioritizing interactions among cells that are spatially close (Supplementary Fig. 6)."

3. The authors only showed signaling pathways super families e.g., WNT, TGFb. Is this cherry-picking? Or are these the most obvious ones? The authors need to show COMMOT is generalizable to more examples of ligand-receptor interactions when interactions are more specific such as PD1-PDL1 (if data available).

Response: In the revision, we have changed the ligand-receptor database to CellChatDB, a more recent database of ligand-receptor interactions containing around 2000 ligand-receptor pairs. With such a database, now we could examine tens of signaling pathways in detail. In this study, we quantify the activity level of each signaling pathway as the summation of the inferred cell-cell signaling score matrix and discuss the signaling pathways with high signaling activity. For example, we analyze in detail the MK signaling pathway for the Visium human breast cancer data and PSAP and FGF signaling pathways for the Visium mouse brain data, which are found to be the most active in the respective datasets according to the criteria described above (Figure 6). Generally, this approach could be applied to any ligand-receptor pairs based on specific biological interests. For example, we have followed the reviewer's suggestion and analyzed the PD1 signaling known to prevent T cells from killing other cells including cancer cells on the Visium data of human breast cancer. The signaling activity was found in several locations in the sample as shown below. The PD1 example is added as new Supplementary Fig. 18.

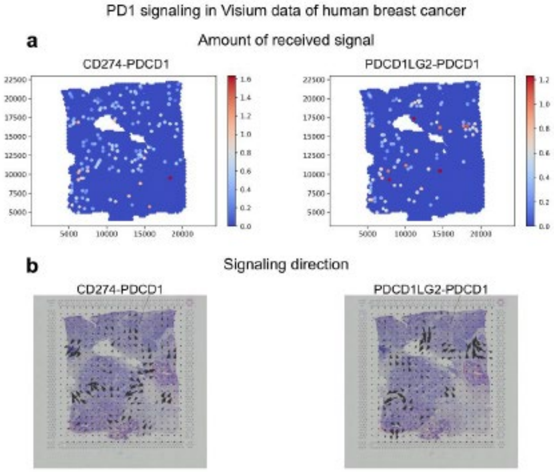

*Supplementary Figure 18. PD1 signaling in the Visium data of human breast cancer. **a** The amount of received signaling through two ligand-receptor pairs of PD1 signaling. **b** The signaling directions of the two ligand-receptor pairs.*

4. In Fig 4, the authors showed signaling DE genes from imputed scRNA-seq data. The immunostaining didn't directly validate the L-R interactions. The authors need to show at least spatial validation of L-R expression in situ using either smFISH or immunostaining. Same for Figs 3, 5, 6.

Response: In the revision, we have conducted new experiments on human epidermis. Namely, we have evaluated predicted cell-cell communication based on Gas6-Tyro3 and Pros1-Tyro3 LR interactions based on fluorescent *in situ* hybridization against ligand and receptor RNA molecules as well as the proteins. Indeed, RNAscope experiments show RNA molecules localize to the epidermal *stratum glanulosum* as predicted which is further confirmed by the immunostaining of proteins. The inference results and the experimental validation of protein and RNA are added to Figure 3 in the revised manuscript are also shown below.

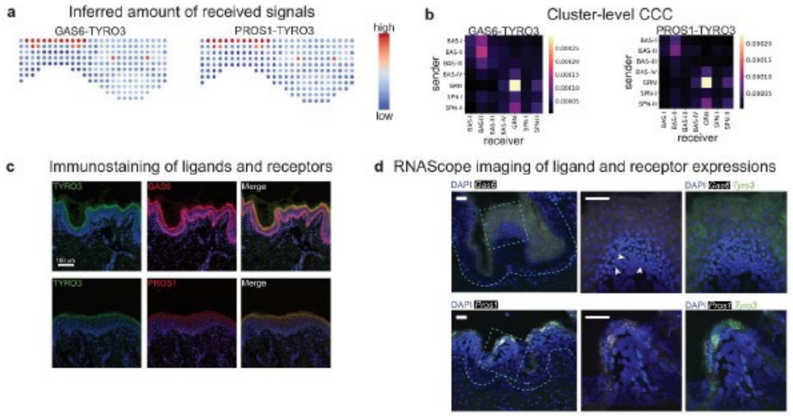

*Figure 3 (partial). **a-b** The inferred amount of received signals of two example ligand-receptor pairs, GAS6-TYRO3 and PROS1-TYRO3 at cell level and cluster level. **c** Immunostaining of proteins for GAS6, TYRO3, and PROS1. **d** RNAScope examination of GAS6, TYRO3, and PROS1.*

In addition to the new experimental results, we have also added several indirect validations in the revised manuscript:

1) (**Findings consistent with prior knowledge**) In the original manuscript, we mainly analyzed two well-studied classical signaling pathways, WNT and TGF-β pathways, on the seqFISH+ data of mouse sscortex, and Visium data of mouse brain and found the inference results are supported by biological knowledge. In the seqFISH+ example, we identified known downstream genes of Wnt signaling pathway and in the Visium data, the recovered signaling directions of WNT and TGF-β pathways were consistent with prior knowledge.

   In the revised manuscript, we have used a more recent and comprehensive ligand-receptor database (CellChatDB) and analyzed all available pathways. We have added two datasets, a MERFISH data on mouse hypothalamic preoptic region and a STARmap data on mouse placenta. a) in the MERFISH example, we consistently found OXT signaling patterns among excitatory and inhibitory neurons recapitulating the known biological functions. b) in the STARmap data, we found opposite signaling directions between MK and IGF pathways indicating a known feedback loop between the two. c) in the Visium mouse brain data, after switching to the new LR database, we also found knowledge-consistent observations of signaling directions of PSAP and FGF pathways. While we are

not able to survey the literature for all analyzed pathways, we have generated the signaling directions and cluster-level signaling for all pathways in the reproducible code for readers to assess. The analysis of MERFISH and STARmap data is added as the new Fig. 4 in the revised manuscript.

2) (**Correlation to known target genes**) In the revised manuscript, we added an analysis of the correlation between the inferred cell-cell communication activity and known target genes using the downstream gene database (Baruzzo, G., Cesaro, G., & Di Camillo, B. (2022). Identify, quantify and characterize cellular communication from single cell RNA sequencing data with scSeqComm. Bioinformatics.) The analyses were conducted on three datasets that have transcriptome or near transcriptome coverage of genes: Visium data on human breast cancer and mouse brain, and seqFISH+ data on mouse sscortex. We demonstrate the correlations between the inferred cell-cell communication activity and the expression of known target genes. This new analysis result is discussed in paragraph 4 of section "Robust identification of spatial signaling direction and downstream targets" in the revised manuscript and new Supplementary Figs. 20-21.
3) (**Continuous change over slices**) In the revised manuscript, the newly added MERFISH data is composed of 12 stacked slices (Figure 4a). This allows us to evaluate the continuity of inferred CCC activity across adjacent slices based on the assumption that CCC activities are more likely to change gradually across the spatially adjacent slices. We show that both gradual changes of the inferred signaling directions and cluster-level CCC are observed (Figure 4b,c and new Supplementary Figs. 16-17).

5. The authors need to benchmark COMMOT with other methods across different datasets and demonstrate superior performance.

Response: We thank the reviewer for pointing this out. In the revision, we have added benchmarks for comparisons to other related optimal transport variants and two existing methods: CellChat, a popular method for CCC inference on scRNA-seq data, and Giotto, a comprehensive package for spatial transcriptomics data analysis including a CCC inference module. The details about the comparisons are listed below.

1) On the simulated data from partial differential equation modeling of ligand diffusion and degradation and ligand-receptor binding, we benchmark the performance of COMMOT with related optimal transport variants including traditional optimal transport, unbalanced

optimal transport, and partial optimal transport. We show that COMMOT has superior performance in predicting the distributions of receptors bound to ligands. This benchmark is added as new Supplementary Figs. 7-8 and discussed in Paragraph 4 of section "Accurate reconstruction of simulated spatial CCC".

2) Using a database of known target genes of the ligand-receptor pairs (scSeqComm), we benchmarked COMMOT, CellChat, and Giotto by examining the correlation between the inferred CCC activity (quantified as the amount of received signal) and the expression of the corresponding target genes. This benchmark is added as new Supplementary Fig. 21 and discussed in Paragraph 4 of section "Robust identification of spatial signaling direction and downstream targets".

More details about the comparisons are listed in reply to Point 1.

6. Mentions that interactions do not exist outside of a certain spatial radius: What is the cutoff for a certain LR pair (please provide the logic behind it) and why use this cutoff/infinity method?

Response: In this work, we study diffusive ligands. Ligands diffuse within limited spatial ranges, so we incorporate a cutoff mechanism in our model to reflect this important physiological property. The diffusion range of a ligand depends on the diffusion coefficient of the ligand which is mainly determined by the a) the protein weight and b) the tortuosity of extra-cellular space (Lander, Arthur D., Qing Nie, and Frederic YM Wan. "Do morphogen gradients arise by diffusion?" Developmental Cell 2.6 (2002): 785-796.). For example, the diffusion distance of BMP is around 100 μm. In practice, it is hard to accurately compute this parameter for each of the analyzed ligand-receptor pairs but this parameter typically ranges from 50 to 150 μm. In the analyses conducted in this paper, relatively large spatial distance cutoffs are used to avoid overlooking certain interactions. In the revision, we have added an example to show the effect of different spatial cutoffs as new Supplementary Fig. 27 shown below. It demonstrates that the inferred cell-cell communication changes slightly when moderately increasing the spatial cutoff (e.g., from 500 μm to 2000 μm). And even when an unreasonably large cutoff is used (e.g., 10000 μm), the inferred local short-distance interactions are still much more significant than the long-distance ones.

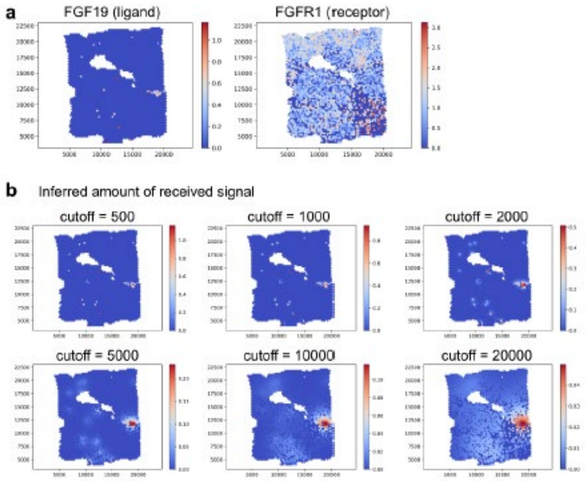

*Supplementary Figure 27. Illustration of effect of different distance cutoffs An example ligand-receptor pair (FGF19-FGFR1) and the Visium data of human breast cancer is used to demonstrate the effect of using different distance cutoffs in COMMOT. **a** The spatial expression of FGF20 and FGFR1. **b** The inferred amount of received signal (spatial distribution of FGF19-FGFR1 complexes with different distance cutoffs in the unit of microns.*

This implies that using a generic spatial distance cutoff (a relatively large cutoff to avoid missing interactions) is sufficient for screening over numerous ligand-receptor pairs. The inference result could be further improved if one narrows down to several important signaling pathways and use the accurately estimated spatial range limit with COMMOT.

We have added a paragraph in Discussion (Paragraph 3) to elaborate this important point:

"The spatial distance constraint in the model is used to capture the effect of ligand diffusivity, which is determined by several factors, including protein weight and tortuosity of extra-cellular space. When screening all ligand-receptor pairs, it is difficult to estimate this parameter for each pair accurately. In our model, the local short-range interactions are emphasized even when the spatial distance range is increased (Supplementary Fig. 27). Thus, when screening many ligand-receptor pairs, a uniform and relatively large spatial distance limit may be used in our model to avoid missing important interactions. Once the important interactions are identified, using an accurate estimation of this parameter would further refine the prediction to remove some false positive CCC links."

7. In Figure 4a, the authors used 208 cells and 12 genes to integrate with scRNA-seq data containing 4264 cells and 33694 genes. I am concerned about the integration given the small number of genes (12!). Thus, all the downstream analysis.

Response: Indeed, integrating spatial data and scRNA-seq data with 12 randomly picked genes would probably lead to unreliable results. However, in this work, the 12 genes were selected to be spatially measured in the original study because they are known to elucidate the spatial arrangement of different types of cells. Our mapped spatial locations of various cell types in the scRNA-seq data are consistent with prior knowledge as well as the spatially mapped pseudotime of scRNA-seq data. For example, the predicted spatial arrangements of the basal cell types and granular cell types reconstruct the known basal-suprabasal axis. The spatially mapped pseudotime also agrees with prior knowledge about the development from basal layer to suprabasal layer.

For further testing, In the revision, we have added a leave-one-out cross-validation. Specifically, in each round, the integration is done without one of the spatial genes and we compared the prediction of that spatial gene by the integrated data to the ground truth. In this cross-validation, most genes are accurately reconstructed supporting the quality of data integration. This cross-validation is added as new Supplementary Fig. 11.

In addition, in the inferred CCC for human epidermis system, we performed new experiments to provide support for two ligand-receptor pairs: 1) immunostaining of ligand and receptor proteins (new Fig. 3d), and 2) RNAScope measurements of ligand and receptor expressions (new Fig. 3e). This further enhances the indirect experimental support of immunostaining of identified DE genes of several major signaling pathways which we did in the original manuscript (new Fig. 3h).

Minor points

1. There is a difference in the naming of the method in the text and some of the figures (collective optimal transport vs supervised optimal transport);

Response: Sorry for the confusion. Collective optimal transport is the formulation for inferring the multispecies interactions in ST data and supervised optimal transport is a formulation for solving the collective optimal transport problem. Since this paper focuses on the biological application,

7. In Figure 4a, the authors used 208 cells and 12 genes to integrate with scRNA-seq data containing 4264 cells and 33694 genes. I am concerned about the integration given the small number of genes (12!). Thus, all the downstream analysis.

Response: Indeed, integrating spatial data and scRNA-seq data with 12 randomly picked genes would probably lead to unreliable results. However, in this work, the 12 genes were selected to be spatially measured in the original study because they are known to elucidate the spatial arrangement of different types of cells. Our mapped spatial locations of various cell types in the scRNA-seq data are consistent with prior knowledge as well as the spatially mapped pseudotime of scRNA-seq data. For example, the predicted spatial arrangements of the basal cell types and granular cell types reconstruct the known basal-suprabasal axis. The spatially mapped pseudotime also agrees with prior knowledge about the development from basal layer to suprabasal layer.

For further testing, In the revision, we have added a leave-one-out cross-validation. Specifically, in each round, the integration is done without one of the spatial genes and we compared the prediction of that spatial gene by the integrated data to the ground truth. In this cross-validation, most genes are accurately reconstructed supporting the quality of data integration. This cross-validation is added as new Supplementary Fig. 11.

In addition, in the inferred CCC for human epidermis system, we performed new experiments to provide support for two ligand-receptor pairs: 1) immunostaining of ligand and receptor proteins (new Fig. 3d), and 2) RNAScope measurements of ligand and receptor expressions (new Fig. 3e). This further enhances the indirect experimental support of immunostaining of identified DE genes of several major signaling pathways which we did in the original manuscript (new Fig. 3h).

Minor points

1. There is a difference in the naming of the method in the text and some of the figures (collective optimal transport vs supervised optimal transport);

Response: Sorry for the confusion. Collective optimal transport is the formulation for inferring the multispecies interactions in ST data and supervised optimal transport is a formulation for solving the collective optimal transport problem. Since this paper focuses on the biological application,

we have deleted the reference to supervised optimal transport in the manuscript and refer to the OT formulation solely as collective optimal transport.

2. Can the authors further demonstrate COMMOT is better than spatial proximity graph with known biological L-R interactions?

Response: In the revised manuscript, we have compared with Giotto, a method based on proximity graph (new Supplementary Figs. 21, 24-25).

1) We compared the properties of COMMOT and Giotto on two Visium datasets (new Supplementary Figs. 24-25. A spatial proximity graph is more suitable to analyze contact-based cell-cell communications while COMMOT can be applied to both contact-based and secreted ligand-mediated by using the spatial distance limit. It is especially suitable for analyzing secreted ligand-mediated cell-cell communications due to its ability to simultaneously a) incorporate an upper-bound of spatial distance for CCC and b) prioritize local interactions which may be much closer than the upper-bound. On the other hand, a spatial proximity graph does not distinguish interactions of different spatial distances if an unweighted graph is used and using a weighted graph will involve many additional parameters that might be hard to interpret.
2) To the best of our knowledge, there is no curated database of known biological L-R interactions. The comparison result will be unreliable if benchmarked on a few manually picked known. We thus use an indirect but systematic evaluation of the method by examining the correlation between inferred CCC activity and the expression level of the known target genes for each ligand-receptor pair using a comprehensive database of downstream target genes (the target gene database from the scSeqComm package). The comparison shows that COMMOT achieves a stronger correlation than Giotto (new Supplementary Fig. 21). This analysis was carried out on the Visium and seqFISH+ datasets due to their good coverage of gene expression which is crucial for systematic downstream analysis.

3. There are other spatial transcriptomics data available such as MERFISH, STARmap. The authors need to demonstrate the performance on more datasets.

Response: In the revision, we have added two analysis examples on a MERFISH data of mouse hypothalamic preoptic region and a STARmap data of mouse placenta. In the revision, the new

results are added as Paragraphs 1-2 in section "Signaling analysis in high spatial resolution ST data", and as new Fig. 4 and new Supplementary Figs. 16-17.

1) In the analysis of the MERFISH dataset, among the signaling pathways available in the data, oxytocin (OXT) signaling, an important pathway modulating social behaviors, was found to be the most active. Self-modulation of excitatory neurons and modulation of inhibitory neurons by excitatory neurons through OXT signaling were identified across all the slices (new Fig. 4b, new Supplementary Fig. 16) which is consistent with the known major functions of OXT signaling. Further analysis shows the local regions of high OXT signaling activity and the spatial direction of OXT signaling (new Fig. 4c). A gradual change of signaling direction and high-activity regions was observed through the adjacent slices (new Fig. 4c, new Supplementary Fig. 17).
2) In the analysis of the STARmap dataset, the midkine (MK) and insulin-like growth factor (IGF) signalings were found to be active in the same regions (new Fig. 4e). Interestingly, the inferred signaling directions of MK and IGF signaling are opposite which indicates a potential feedback loop between the two signaling pathways. We also found similar active regions and directions for Annexin and Angiopoietin (ANGPT) signaling suggesting that they might function cooperatively (new Fig. 4e).

Reviewer #3:

Remarks to the Author:

Review of "Screening cell-cell communication in spatial transcriptomics via collective optimal transport" (NMETH-A47293)

Summary

With COMMOT, Cang et al., present an elegant mathematical solution to the problem of inferring cell-cell communication (CCC) from spatial transcriptomics data based on a variant of optimal transport (OT). Their method considers competition among different ligand-receptor species, includes spatial constraints and does not require the input measures (ligand/receptor expression) to be normalized, thus preserving the original measurement units. The method is applied to spatial datasets of different sizes (~1k to ~50k spots or cells) and technologies, including both imaging (seqFISH+) and sequencing (Visium, slide-seqV2) based approaches and robustness of results is shown. Further, the authors show how their method can be used in different biological contexts, including mouse breast cancer and the brain. The concept figure gives a clear overview of their method and subsequent figures are well-organized. Further, the software package is well organized and documented, includes a tutorial and interfaces with standard single-cell packages such as AnnData and Scanpy.

However, I have concerns about the relationship between their method ("collective optimal transport") and previous OT extensions, in particular, multimarginal, partial and unbalanced OT, which individually solve the same problems the authors outline. These connections should be made explicit, cited accordingly and it needs to be clearly demonstrated why a new OT extension is necessary to clearly delineate novelty. Further, the authors do not benchmark their method against any other established method for CCC inference, they only consider a simple OT approach as baseline. This makes me wonder what the added benefit of their method is in practice. This needs to be clearly demonstrated using both their simulation study as well as one of their real data examples.

As it stands, the authors present an elegant mathematical concept with a good implementation, but fall short of demonstrating how their method gives practical advantages over existing approaches when applied to real biological settings. Are there new biological questions that can be studied with this approach, which could not have been studied equally well with existing approaches? I give details about my concerns in major and minor comments below.

Response: To address the reviewer's concerns on the lack of comparisons to other OT formulations and to existing CCC inference methods, in the revised manuscript, we have added 1) comparisons to unbalanced OT and partial OT on simulated data and real data and 2) comparisons to two existing CCC inference methods CellChat and Giotto on real data, to show the superior performance of COMMOT. Please see the reply to the points below for details.

Major points

1. Introduction, paragraph starting in line 63, the authors motivate the need for developing a new optimal transport (OT) method. They do so in line 71 by claiming

"However, when utilizing existing optimal transport methods, ligand and receptor expression are normalized as probability distributions, and therefore, the original units of expression levels are not preserved making the distributions of ligand and receptor incomparable."

From my point of view, this claim is incorrect. While it is correct that the standard OT problem is formulated for probability distributions (hence requiring normalization of ligand and receptor expression), unbalanced OT resolves this problem, as outlined in chapter 10.2 of ref. (Peyré and Cuturi 2019), which the authors cite in the same sentence. I quote from ref. (Peyré and Cuturi 2019):

"In practice, unbalanced OT techniques seem to outperform classical OT for applications (such as in imaging or machine learning) where the input data is noisy or not perfectly known. They are also crucial when the signal strength of a measure, as measured by its total mass, must be accounted for, or when normalization is not meaningful. This was the original motivation of Frogner et al. [2015], whose goal was to compare sets of word labels used to describe images. Unbalanced OT and the corresponding Sinkhorn iterations have also been used for applications to the dynamics of cells in [Schiebinger et al., 2017]."

As correctly noted by the authors of ref. (Peyré and Cuturi 2019), Schiebinger et al. (Cell 2019) already make use of unbalanced OT to account for cellular growth and death - another work that

is cited by Cang et al. in the same sentence. Another work the authors cite in the same sentence, ref. (Chizat et al. 2018) introduced an efficient algorithm to solve unbalanced OT problems. For a simple illustration of how unbalanced OT can be applied to discrete measures that are not probability distributions (don't sum to one), see https://pythonot.github.io/auto_examples/unbalanced-partial/plot_UOT_1D.html# which is taken from the docs of Python Optimal Transport (POT), the most common framework for Optimal Transport analysis in Python. Hence, the motivation outlined by the authors here to develop a new computational method is factually incorrect - the problem of computing a coupling between discrete measures that are not probability distributions is solved by unbalanced optimal transport, as outlined in many of the works the authors cite themselves.

This problem continues in the next paragraph, line 82. The authors write

"To address these two problems, we present a novel method called collective optimal transport, which is capable of preserving the original units without the need for normalization, enforcing spatial range limits of signaling, and handling the cases of multiple competing species. "

From the previous paragraph, I conclude that the two problems the authors set out to tackle are

OT cannot be applied to input measures which are not probability distributions

Competition among ligand and receptor species, which is not taken into account by existing CCC methods which perform pairwise comparisons.

As the authors themselves explain, OT solves the second problem. The first problem, however, is invalid since unbalanced optimal transport, an established framework with efficient computational algorithms already existing, solves this problem. Thus, from this description, I do not see the need to develop a new OT extension. Moreover, since an efficient solution to this problem exists which is used by others both within (Schiebinger et al. 2019) and outside the single cell field, I think the authors should consider basing their method on unbalanced optimal transport rather than developing their own algorithm. The advantages of using unbalanced optimal transport are clear: it's an established method with mathematical guarantees and efficient, numerically robust and scalable algorithms to solve it (Peyré and Cuturi 2019; Chizat et al. 2018).

Response: We thank the reviewer for the careful assessment. In summary, collective OT, unbalanced OT, and partial OT can all take unnormalized distributions but in different ways. Specifically:

- Unbalanced OT uses soft KL penalty terms, and the total inferred ligand-receptor complex (sum of transport plan) may exceed the total available ligand or receptor (initial distributions).
- Partial OT bounds the inferred ligand-receptor complex (marginal distributions of the transport plan) from the above by the ligand and receptor levels (initial distributions) and requires a *prescribed total mass* which is usually set to the smaller one between the total initial distributions. However, the choice of the prescribed total mass becomes non-trivial when the cost matrix contains infinity entries. Due to these infinity entries, the total possible transported mass may be strictly smaller than both the total mass of ligand and receptor and is unknown. When the prescribed total mass exceeds the total possible transported mass, partial OT will generate marginal distributions exceeding the initial distributions. In this case, a way to find the total mass is needed.
- Collective OT incorporates the total mass as a variable to be optimized together with the transport plan considering the infinity patterns in the cost matrix. It, therefore, prevents creating extra ligand-receptor interactions that exceed the available ligand and receptors and enforces the transport plan according to the infinity patterns in the cost matrix. In this way, no connections will be allowed between pairs of ligand-receptor that cannot interact or pairs of cells whose distance is greater than the spatial range of signaling.

We agree it is inaccurate to state that "using existing OT methods (including unbalanced OT), the distributions need to be probability distributions." However, as summarized above, they treat the unnormalized distributions in different ways. In the revised manuscript, we have added additional examples and results to demonstrate the connections and the many differences among the three OT variants. We show that collective OT is more suitable for the application of cell-cell communication inference.

Specifically, we made the following additions and modifications in the revised manuscript:

1) We have added a 1D example to illustrate the difference among the three OT variants. The unbalanced OT with KL divergence regulation of unmatched distributions will introduce artificial marginal distributions. For example, the row sum of the transport matrix may exceed the original source distribution. While this property works for some applications, in our studied systems, it will

introduce artificial extra ligand-receptor interactions when the total expressions of the ligand and receptor are significantly unbalanced. The partial OT takes the total coupled mass as an input parameter that needs to be given in advance. This total coupled mass is often set to the smaller one of the total masses of the two distributions if not given by the user. Unlike these two OT variants, the marginal distributions are guaranteed to not exceed the original distributions and the total coupled mass is inferred simultaneously with the transport plan. The differences are illustrated in detail in the following figure which is added as new Supplementary Fig. 9 in the revised manuscript.

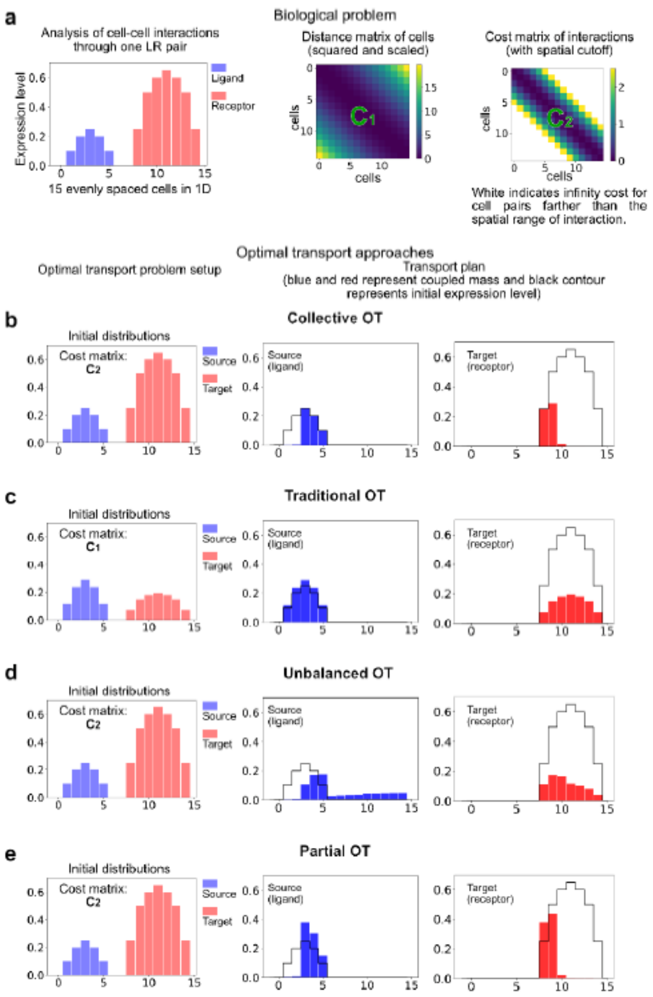

Supplementary Figure 9. Comparison of OT variants on an example ligand-receptor interaction analysis in 1D. a This example studies the interaction among 15 cells evenly placed in 1D through one pair of ligand and receptor with a spatial range of 5 units so that any pair of cells whose distance exceeds 5 cannot interact. b-e The initial distributions and cost matrices that each OT method can take and the marginal distributions of the optimal transport matrix are shown.

2) Next, we have compared the unbalanced OT and partial OT with collective OT on the PDE simulation data of cell-cell communications. The following two comparisons show that collective OT performs better than the other two OT methods on the PDE simulation data and further demonstrate that the three methods behave differently. These performance comparison results are added as new Supplementary Figs. 7-8 in the revised manuscript.

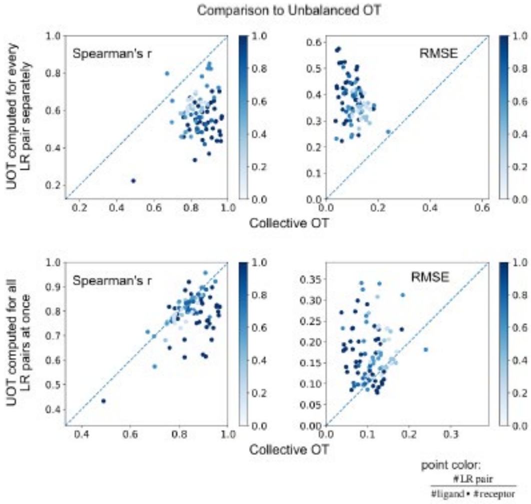

Supplementary Figure 7. Comparison of collective OT with unbalanced OT on simulated data. Each dot represents a simulated dataset and the dot color depicts the number of LR pairs divided by multiplication of numbers of ligand and receptor species which indicates the extend of competence. The performance is evaluated by comparing the inferred amounts of occupied receptors in each location with the simulated one. Spearman's correlation coefficient and root-mean-squared error are used.

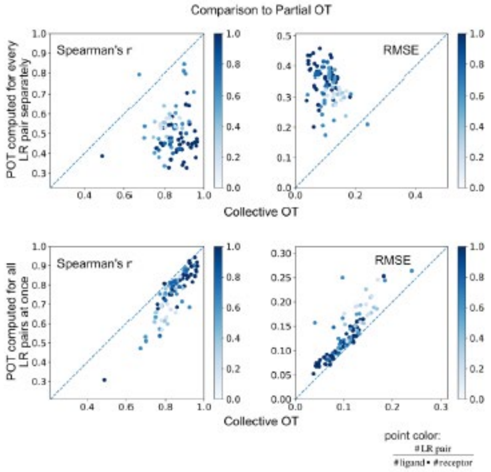

Supplementary Figure 8. Comparison of collective OT with partial OT on simulated data. The comparisons with partial optimal transport that examines each ligand-receptor pair separately (top) or all pairs together (bottom). All analyses use the same spatial cutoff such that the transport cost is set to infinity if the distance between a location pair exceeds the distance cutoff. The optimal transport analysis results are evaluated by comparing the inferred amount of received signal to those obtained by simulation using Spearman's correlation coefficient and root-mean-squared error.

3) Using Visium datasets, we further illustrate the difference between COMMOT and unbalanced OT and partial OT. a) When the total amount of ligand and receptor expressions are significantly different, unbalanced OT generates artificial connections due to the KL divergence regularization. b) When there are infinity entries in the cost matrix (e.g., induced by a limited spatial range of cell-cell communication), the maximum possible transported mass may be even smaller than the smallest total initial mass. This maximum possible transported mass is an unknown and is computed by COMMOT. In partial OT, if the total mass is not given, the smallest total initial mass is used as the total mass. In the case discussed above, this would result in the marginal distribution of the transport plan exceeding the initial distribution. In the context of CCC inference, this means the inferred sent or received signal would exceed the initial distribution of ligands and receptors. This demonstration is added as new Supplementary Fig. 10 and is shown below.

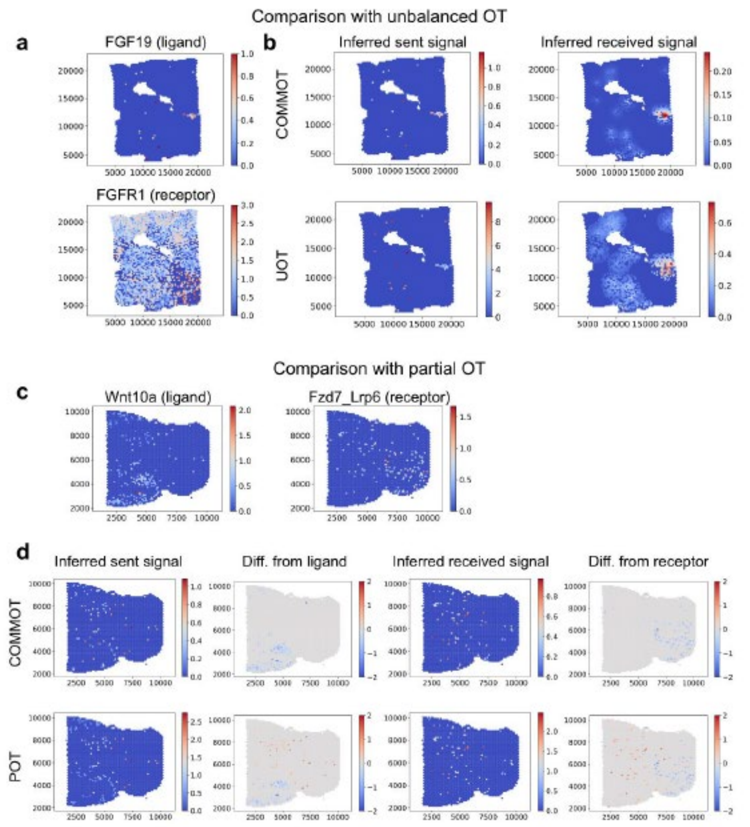

Supplementary Figure 10. Differences between OT variants on example Visium datasets **a** The difference between COMMOT and unbalanced OT is demonstrated with a Visium dataset of human breast cancer focusing on the ligand-receptor pair FGF19-FGFR1 whose total expression levels are significantly different. **b** The amount of sent and received signal is plotted. Compared to COMMOT, unbalanced OT overestimates the signaling activity. Specifically, the amount of sent signal inferred using unbalanced OT significantly exceeds the expression level of ligand. **c** The difference between COMMOT and partial OT is demonstrated with a Visium dataset of mouse brain focusing on the ligand-receptor pair Wnt10a-Fzd7_Lrp6 whose total expression levels are comparable but are expressed in spatially distinct regions. **d** The amounts of sent, received signal and their difference to the expression levels of ligand and receptor are plotted. Compared to partial OT, COMMOT further prioritizes potential signal senders and receivers. The difference plots show that the sent and received signal inferred using partial OT exceed ligand and receptor expressions in some regions leading to overestimation of signaling activity.

Also, on the real dataset with the comprehensive LR database, we are not able to examine all the LR pairs together with shared ligands and receptors among the pairs using the implementation of unbalanced or partial OT implemented as dense matrices in the Python Optimal Transport library.

4) We have revised the introduction to more accurately present the connection to unbalanced OT and partial OT and modified the description about handling unnormalized distributions.

"Naturally, one can view ligand and receptor expression as two distributions to be coupled with a cost based on spatial distance to form an Optimal Transport (OT) problem. However, using classical OT, when different species with significantly different expression levels are normalized to ensure the same total mass, it makes the units of distributions incomparable. While recent OT variants such as unbalanced OT and partial OT can deal with unnormalized distributions, they introduce some other issues. Specifically, unbalanced OT in its common form uses KL divergence as a soft constraint on marginal distribution preservation, and this may result in the total coupled species significantly exceeding the total amount of either ligand or receptor. On the other hand, partial OT, requires an additional parameter, the total coupled mass, which is usually difficult to estimate in the context of CCC inference."

And "By introducing an entropy regularization to enforce the inequalities for marginal distributions, the collective optimal transport can be reformulated as a special case of the general unbalanced optimal transport framework. An efficient algorithm is developed specifically for solving the collective optimal transport problem."

In short, the original formulation of the collective optimal transport problem (Eq. (1) in supplementary material) is motivated by the biological application. It can be reformulated into a slightly different form (Eq. (3) of supplementary material) by using entropy terms to enforce the inequality constraints in Eq. (1) of supplementary material). This form in Eq. (3) is indeed a special case of the general unbalanced optimal transport framework but differs from the commonly used one with KL divergence setup. We have modified the manuscript accordingly about the detailed connection to the related work.

2. Results section, line 112, the authors write:

"This method has two important properties: 1) it couples multi-species distributions as opposed to traditional optimal transport that couple single-species distributions and 2) it allows infinity

entries in the cost matrix to enforce spatial distance limits of CCC to avoid connecting cells that are too far apart spatially, while traditional optimal transport can only deal with finite entries"

I have two problems with this statement. First, their method description changed significantly from what it was in the introduction, see my comment above. Second, I'm again unsure whether there's a need for a new computational method here. The authors stress that their method has two important properties:

It couples several species at once, instead of just a single, as in classical OT

It allows for infinite entries in the transition matrix

From my understanding, problem 1 is classically solved using multimarginal OT, see Chapter 10.1 in ref. (Peyré and Cuturi 2019) and the following example from POT: https://pythonot.github.io/auto_examples/domain-adaptation/plot_otda_jcpot.html# Problem 2 is classically solved using partial OT, see ref. (Figalli 2010) and https://pythonot.github.io/auto_examples/unbalanced-partial/plot_partial_wass_and_gromov.html# from POT. If that is not the case, i.e. if there is some reason that prevents the authors from using these standard techniques, they should clearly state this and explain why this is the case.

In their methods supplement, the authors give more details and show how their problem can be broken down to equation Suppl(3), which to me, this looks like a multimarginal unbalanced OT problem, and connections to these earlier methods need to be made obvious and cited accordingly. IMHO, the authors should pay special attention to infinite entries in the cost matrix, which could also be addressed using partial OT, and again, connections need to be made clear. Also, the algorithms they derive reduce to Sinkhorn iterations (the derivations are very nice), which yet again makes me wonder whether this is just a version of unbalanced OT. This problem is also apparent in the discussion, starting in line 413, where the authors write:

"While traditional optimal transport is powerful at integrating one pair of datasets, collective optimal transport can be powerful for multi-omics data integration as it couples multiple datasets at the same time, so that coupling of one dataset pair provides information about integration of other datasets. Moreover, the ability to preserve the original units of mass distributions allows collective optimal transport to better integrate unbalanced datasets, which is a common challenge in data integration, due to different limitations of different technologies."

Again, this gives the impression that there exist no established OT extensions that can deal with multiple marginals or an unbalanced setting, which is not true. These extensions exist, they are

widely used and implemented in standard python packages. The authors should avoid giving the impression of "reinventing the wheel" by making connections of their method to previous approaches explicitly, citing these appropriately and clearly stating why there is a need for a new OT extension in light of existing approaches.

What's the difference between supervised optimal transport (SOT), collective optimal transport and COMMOT? The authors use all three terms and it's unclear what their relationship is. This needs to be made explicit, it's currently confusing.

Response: We thank the reviewer for further pointing out the relevant works and are sorry for the confusion. There are two major confusions: 1) the ability of multimarginal OT to handle multiple species and 2) the ability of unbalanced OT and partial OT to handle infinity entries. We clarify below in detail that 1) the interpretation of multimarginal OT is totally different from the purpose of our application and 2) like Point 1 about normalization, the three formulations handle the infinity entries in different ways and collective OT is more suitable for our application.

For the term "coupling multiple species", it is totally different in our application from the interpretation of multimarginal OT. In our application, for an example of two source distributions (ligand species) and one target distribution (receptor). The multimarginal OT would derive a 3D tensor as the transport plan and a nonzero element in the transport matrix means the coupling of three locations altogether (two locations in the two sources and one location in the target). In the interpretation of the CCC inference application under this setup, when we interpret sources as ligands and targets as receptors, an occupied receptor would need both ligands to contribute. However, our application focus is to capture the biological process in which one ligand is enough to occupy a receptor although the receptor may also interact with other ligand species.

For "allowing infinity entries" in the cost matrix, our description in the original manuscript was not totally accurate. Indeed, that unbalanced OT and potentially partial OT can deal with infinity entries. However, as in the response to Point 1, our method differs from the unbalanced OT in its common setup (with KL divergence). Specifically, collective optimal transport was formulated in its original form (Eq. (1) of supplementary material) motivated by the biological application and reformulated into a slightly different form (Eq. (3) of supplementary material) to derive efficient algorithms. The second form is indeed a special case of the general framework of unbalanced optimal transport studied in (Chizat, Lenaic, et al. "Scaling algorithms for unbalanced optimal transport problems." Mathematics of Computation 87.314 (2018): 2563-2609.). Collective optimal

transport is also different from partial optimal transport in that the former optimizes the total mass induced by infinity entries while the latter takes a predefined total mass.

For implementation, only the formulation of unbalanced optimal transport with the KL divergence is available in the popular Python Optimal Transport package. Our specific formulation of collective optimal transport is different from the existing specific formulation, and we couldn't find any existing implementations. We thus need to derive new algorithms and use our own implementations.

As for the last point in this comment about the claim in the last paragraph of discussion, the original description was inaccurate. But as stated above, although related, our method is different from unbalanced multimarginal optimal transport. In the revised manuscript, we have changed the last paragraph as the following to more accurately distinguish collective optimal transport from existing ones:

"While traditional optimal transport is powerful at integrating a pair of datasets and multimarginal optimal transport integrates multiple datasets, the collective optimal transport is able to effectively deal with competing species. Moreover, collective optimal transport provides a new way to preserve units and control coupled mass. The collective optimal transport in this work will be useful for a broad range of problems beyond the CCC inference."

Regarding the connections among collective OT, supervised OT, and COMMOT, we have revised the manuscript to clarify. Collective OT is the model motivated by the CCC inference application (Eq. (1) of main text). Supervised OT was the name for an equivalent form (Eq. (2) of main text) from which we derive the algorithms. In the revised manuscript, **we have removed the term supervised OT** and only refer to the OT formulation as collective OT. COMMOT is the name for the software package and the corresponding method for the collective OT's application to CCC analysis.

3. Fig. 2, the authors should benchmark their method against other methods that can infer CCC, currently, they only compare to standard OT, applied independently to each ligand-receptor species pair. They should compare against both methods that make use of spatial information (like Squidpy, see the efficient cellphone db reimplementation) and those that do not when inferring CCC (the authors cite many of these methods in the introduction). The simulation

example should be used to demonstrate how COMMOT improves over previous approaches, and which limitations to the approach exist.

Response: We thank the reviewer for this suggestion. In the revised manuscript, we have made comparisons to two existing CCC inference methods: 1) CellChat which is designed for scRNA-seq data and has been widely used and 2) Giotto which is a recent comprehensive package for analyzing spatial transcriptomics data. The comparisons show the different properties among these methods (new Supplementary Figs. 22-25). We have also compared the performance of these methods based on comparing the inferred signaling and the expression of known downstream genes (new Supplementary Fig. 21) where COMMOT achieves better performance.

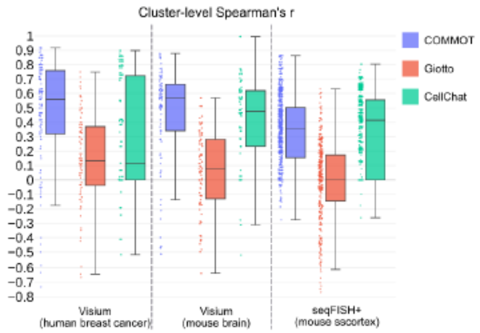

Supplementary Figure 21. Cluster-level correlations between the total amount of received signal from the identified cell-cell communication and the expression of known downstream genes. Each point represents one ligand-receptor pair.

We compared with Giotto instead of Squidpy since the CellPhoneDB implementation in Squidpy does not utilize spatial information. The simulation data is not applicable here because the existing methods output cluster-level cell-cell communication networks while the current simulation data does not have a notion of clusters. We thus compared them on real data. The Visium and seqFISH+ datasets are used here because of their good coverage of gene expression identification which is important for downstream analysis.

4. Fig. 3 and corresponding text, similar to my above comment, it's not clear from text or figure whether the recovered signalling direction could not have been equally well recovered using existing approaches. The authors could place more emphasis on highlighting shortcomings of previous approaches, and how their method improves upon them, not just in terms of theoretical

analysis, but also in practical applications. If, for example, other computational approaches cannot be used to find partial DE genes (Fig. 3f), then this needs to be stated and shown in an example. The advantages of COMMOT over previous approaches need to be made much more concrete.

Response: We have followed the reviewer's suggestion to demonstrate the improvement of our method over existing ones.

1) The signaling direction visualization as a detailed vector field is a unique feature of COMMOT which is not available in other existing packages. In the revised manuscript, we have pointed this out in Discussion.
2) Related to Point 3, we have added comparisons with two existing methods (CellChat and Giotto) in the revised manuscript (Paragraphs 4 and 5 of section "Robust identification of spatial signaling direction and downstream targets"). CellChat is a widely used tool for inferring CCC from scRNA-seq data and Giotto is a recent comprehensive package for spatial transcriptomics data analysis with a function for spatial CCC inference. In the revised manuscript, we have taken a more systematic approach for evaluation using a database of known downstream genes (scSeqComm) of various signaling pathways. All available ligand-receptor pairs with known downstream genes were evaluated for three datasets. This systematic comparison demonstrates that COMMOT performs better than the other two methods (see the figure in Point 3).
3) The differences between the identified cell-cell communication links among the three methods (new Supplementary Figs. 22-25) with examples show the spatially significant CCC that are uniquely identified by COMMOT.

The old Figure 3 (drosophila embryo example) in the original manuscript was mainly for robustness assessment and has been moved to supplementary as supplementary figure 18 to make place for the added example analysis of more spatial transcriptomics technologies in the revised manuscript (Figure 4). We believe the DE gene analysis in the drosophila embryo example is still a valuable assessment of robustness of the method. But because we only found database on downstream genes for the drosophila system with only a few signaling pathways, we used the more comprehensive database for human and mouse for comparison with existing methods.

5. Fig. 3, I don't find the evaluation of identified genes convincing. First, it would be good to see that these genes cannot be inferred equally well with existing, simpler approaches, see above

comment. Second, the metric in Fig. 3e is not convincing: simply looking at the percentage of known genes identified does not take false positives into account, i.e. genes identified by COMMOT's differential expression test which are known not to play a role downstream of DPP signalling.

Response: Related to Point 4, we have added a comparison with two existing approaches, Giotto and CellChat designed for spatial data and scRNA-seq data, respectively. A much more systematic approach for evaluation is used with hundreds of ligand-receptor pairs and a comprehensive database of known downstream genes (the database provided in the scSeqComm package). In the new evaluation, we changed the metric to the correlation between expression levels of known downstream genes and the inferred CCC activity through the corresponding ligand-receptor pair, to focus on genes known to play a role. Such evaluation based on correlation addresses the problem of not considering false positives when the evaluation was cast as a classification problem as in the original manuscript. We agree that also incorporating genes known not to play a role is ideal. However, such a "negative control" database is lacking, and it is difficult to manually curate such a database from literature as well.

The Giotto package uses a simpler approach by quantifying the average expression of ligand and receptor among spatially connected cells. In the comparison, it is demonstrated that the CCC inferred by COMMOT has a stronger correlation with the expression of known downstream genes.

The original Fig. 3 serves mainly as a robustness assessment of COMMOT and has been moved to supplementary in the revised manuscript. The added systematic evaluation and comparison with other methods should better demonstrate the performance of COMMOT.

6. Methods section, starting line 434, the authors write:

"The collective optimal transport results in a large-scale optimization problem for which efficient algorithms are developed."

While it is in fact true that the authors reduce their algorithm to Sinkhorn iterations, they do not demonstrate that their algorithm is efficient, neigher in theory nor practice. In fact, standard sinkhorn iterations are known to scale quadratically in sample number (Peyré and Cuturi 2019),

which, by modern ML standards, is not considered to be efficient. This does not rule out using OT, in fact, OT is used in many applications in data science, however, for the Sinkhorn updates developed in the methods supplementary for the L1 and L2 cases, it should be stated explicitly how compute time and memory complexity scale with the number of cells, ligand and receptor species to evaluate scalability of the proposed model. Ideally, this would also be demonstrated in a practical example where peak memory usage and compute time are recorded for test problems of increasing size (cell number, ligand/receptor pairs).

Response: We thank the reviewer for this insightful comment. Yes, our algorithm is reduced to Sinkhorn iterations, and it scales quadratically in sample number when the cost matrix has only finite entries, and linearly to the number of nonzero elements in the cell-cell communication matrices (transport plan) when the cost matrix has infinity entries. Due to the sparsity of our problem (the number of nonzero elements in the transport plan scales linearly to the number of samples since we are restricting the transport plan to be a distance network), the computational time and memory scale only linearly to sample size times the number of ligand-receptor pairs available. In the revised manuscript, we have included numerical experiments about the computation time (new Supplementary Fig. 26) confirming the scalability. For the largest problem (Visium data analysis due to the high level of detected genes and the large number of measured ligand and receptor genes), the algorithm can finish in about an hour on a regular desktop (with i7-8700k) which we believe is a reasonable running time. A description of the compute time and memory is added to the last paragraph of Results section. We have changed the statement to clarify that by "efficient", we mean the Sinkhorn iteration known as an efficient algorithm for optimal transport:

"The collective optimal transport results in a large-scale optimization problem for which algorithms are developed based on the efficient Sinkhorn iteration."

7. Code: the code is well-organized and documented. However, it's unclear to me why the authors have to re-implement the sinkhorn algorithm since efficient implementations exist, e.g. in POT. Of course, sinkhorn is very simple to implement, nevertheless, by making use of existing implementations, the authors could benefit from more recent accelerations of sinkhorn, see the many solvers implemented in POT: https://pythonot.github.io/all.html Further, making use of existing OT packages as building blogs will likely result in fewer bugs since an entire community uses POT and fixes bugs. Furthermore, it's unclear to me why the authors re-implement KNN

graph construction in `_clustering.py`. They should make use of existing efficient implementations, such as `HNSW` or `pynndescent`. This is a general point, wherever efficient solutions are available in the community already, they should be used, rather than reinventing the wheel. Especially when it comes to KNN construction - this is a very well researched area and highly optimized solutions exist.

Response: We thank the reviewer for this valuable suggestion.

1) There is no available implementation for the collective optimal transport algorithm (see discussions in major points 1 and 2). The Sinkhorn algorithms implemented in POT are for other formulations different from the collective optimal transport in this work. In addition, we have to implement collective optimal transport with *sparse matrices* due to the size of the CCC inference problem (e.g., the number of bins in the source distribution is the number of cells/spots multiplied by the number of ligand species). Implementations in POT use dense matrices and are computationally intractable for evaluating CCC by considering the relation among all different LR pairs.
2) In the revision, we have included the kneighbors_graph function from the scikit-learn package following the reviewer's suggestion. KNN graphs are used to cluster the LR pairs or the DE genes which usually have fewer than 1000 points. Thus, for clarity, reproducibility and to avoid potential dependency issues, we use exact implementations instead of the efficient approximations such as pynndecent. The KNN implementation with an input distance matrix is kept since non-standard metrics are used in some cases.

8. Code: there are no tests. For a software to be published in a high impact journal like Nature methods, I expect to see a minimal number of unit & regression tests that ensure the software is stable, mostly bug-free and can be maintained in the future to serve the community. Please include tests and set up CI.

Response: We thank the reviewer for this constructive suggestion. We have developed standard tests using pytest in the revised software package. We have also included test Jupiter notebooks to reproduce the functionalities of the package on example real datasets.

Minor points

In the abstract, I find the description of datasets used for application confusing. The authors write "We validate the method with simulation data and one spatially annotated scRNA-seq dataset. We show that COMMOT effectively infers spatial CCC using datasets by three popular spatial transcriptomic technologies." After reading this, it's unclear to me whether the authors used one or three datasets to apply their method. This should be made much more clear in the abstract, it's essential information.

Response: Sorry for the confusion. In the revised manuscript, we have changed this sentence to "We apply COMMOT to simulation data and eight spatial datasets acquired with five different technologies, showing its effectiveness and robustness in identifying spatial CCC in data with varying spatial resolutions and gene coverages." We added two datasets in the revised. Some datasets used are from the same ST technology and some are obtained by integrating scRNA-seq with spatial imaging data.

Introduction, line 41, the authors write "The majority of these methods rely on the expression levels of ligand and receptor pairs and predefined empirical formulas." Unclear to me what exactly they mean by "predefined empirical formulas". In the end, mathematical models define a way to process inputs to outputs and at some stage, they all contain "formulas", don't they?

Response: Sorry for the confusion. While all methods can be viewed as a function, they can take very different forms. By predefined formulas, we refer to methods that used an explicitly defined function. Other forms include machine learning models and the method in this paper that involves some optimization. We use this description to categorize the existing methods leading to organize the following description of relevant methods. In the revised manuscript, we have changed the wording from "predefined empirical formula" to "explicitly defined functions".

Intro, line 55, the authors write "Spatial transcriptomics technologies provide distance information among cells." Many current technologies do not achieve single-cell resolution, e.g. visium, so I don't think this claim is formulated a bit too broadly.

Response: Sorry for the confusion. This sentence has been changed to "Spatial transcriptomics technologies provide distance information among cells or spots containing multiple or fractions of cells."

Same paragraph as above, the authors open with this sentence about spatial transcriptomics technologies but proceed to introduce computational methods; this is confusing - I expect to learn about spatial technologies after this opening sentence.

Response: Sorry for the confusion. We have added a brief description of the typical output of these spatial transcriptomics technologies and cited a review article about ST technologies:

"With various cellular resolutions, these technologies measure spatial expression of hundreds to tens of thousands of genes in a 2-dimensional or a 3-dimensional tissue sample[21]."

21. Rao, A., Barkley, D., França, G. S. & Yanai, I. Exploring tissue architecture using spatial transcriptomics. Nat. 2021 5967871 596, 211–220 (2021).

There are spelling and grammar mistakes in the text. I will just give an example from the intro, line 65, the authors write " there is competition between cells and therefore systematically examine CCC among all cells requires a global perspective".

Response: Sorry for the mistakes. We have carefully proofread the manuscript and fixed several grammar mistakes.

Introduction, line 93, the authors write "infers CCC in spatial transcriptomics data or spatially annotated scRNA-seq". They used these terms a few times before, without ever explaining what the difference between them is. The terms need to be defined before they are used. After reading the entire text, I understand the latter refers to spatially imputed data, however, this could be explained earlier on.

Response: Sorry for the confusion. We have added the definition in the revised manuscript: "1) infers CCC in spatial transcriptomics data or spatially annotated scRNA-seq data (scRNA-seq data with estimated spatial distance between cells based on paired spatial imaging data) by simultaneously considering numerous ligand-receptor pairs;"

Introduction, line 95, the authors write "identifies potential downstream impacts on gene expressions by CCC using machine learning models" - what are these "machine learning

models"? Inflationary usage of the term "machine learning model" should be avoided, rather state which model was used here (Random Forests, see Line 353)

Response: Sorry for the confusion. We have changed "machine learning models" to "ensemble of trees models" since they will work under the same principal as the random forest used in COMMOT that the feature importance of the trained models is used as indicators of CCC impact.

Line 304, the authors write "In this case, the CCC-induced clusters are mixed in space suggesting that the CCC activity is similar across the different tissue regions." - is that expected in this experimental system? Is there anything known about this in the literature? At the moment, it's unclear whether this result is biologically meaningful

Response: Thank you for pointing this out. In this example, we intend to illustrate the difference between the CCC-induced clustering and cell-type based clustering and an example. In the revised manuscript, we have clarified that this is an example observation from the computational results and the subsequent hypothesis. The sentence is changed to "As opposed to the spatial separation of cell types, we observe the CCC-induced clusters to be mixed in space suggesting that different cell types might have similar signaling activities through multiple signaling pathways.".

Line 341, why is tradeSeq employed here and not in earlier analysis to identify DE genes as a function of signal reception? Should be motivated somehow.

Response: Sorry for the confusion. Different examples are used to emphasize different functions of the package. We are not able to list full analysis for all examples in the paper. Specifically, DE gene analysis with tradeSeq was performed for the integrated single-cell data and the visium data because of their good gene coverage and relatively large number of cells/spots compared to other technologies. In the revised manuscript, we have added a clarification to where analysis of signaling DE genes is first introduced (Paragraph 1 of section "Signaling analysis in multi-cell resolution ST data").

"The signaling DE gene analysis in ST data is similar to the temporal DE gene analysis in scRNA-seq data. Therefore, ST data with good coverage of genes and large number of cells or spots is preferred for such analysis."

Fig. 4c,d, it's unclear to me after looking at the panels, reading the legend and the corresponding text to what extent their computational prediction was validated using the stainings. This explanation is given in the discussion, starting line 391. Consider moving this.

Response: Sorry for the confusion. In the figure caption, we have added description of why these staining images are listed: "d Immunofluorescence staining images of the identified signaling DE genes supporting the identified correlation between WNT signaling and expression of these genes."

References

Chizat, L., G. Peyré, B. Schmitzer, and F. X. Vialard. 2018. "Scaling Algorithms for Unbalanced Optimal Transport Problems." Mathematics of Computation. https://www.ams.org/mcom/0000-000-00/S0025-5718-2018-03303-8/.

Figalli, Alessio. 2010. "The Optimal Partial Transport Problem." Archive for Rational Mechanics and Analysis 195 (2): 533–60.

Peyré, Gabriel, and Marco Cuturi. 2019. "Computational Optimal Transport: With Applications to Data Science." Foundations and Trends® in Machine Learning 11 (5-6): 355–607.

Schiebinger, Geoffrey, Jian Shu, Marcin Tabaka, Brian Cleary, Vidya Subramanian, Aryeh Solomon, Joshua Gould, et al. 2019. "Optimal-Transport Analysis of Single-Cell Gene Expression Identifies Developmental Trajectories in Reprogramming." Cell 176 (6): 1517.

Decision Letter, first revision:

Dear Qing,

Your revised Article, "Screening cell-cell communication in spatial transcriptomics via collective optimal transport", has now been seen by the three original reviewers again. As you will see from their comments below, although the reviewers find your revised manuscript has been substantially improved, they still have a number of concerns. We are interested in the possibility of publishing your paper in Nature Methods, but would like to consider your response to these concerns before we reach a final decision on publication.

We therefore invite you to revise your manuscript to address these concerns. Please bear in mind that we do not require benchmarking comparison against tools published after the manuscript was submitted to us. Please focus your revision on the technical concerns raised by the reviewers.

We are committed to providing a fair and constructive peer-review process. Do not hesitate to contact us if there are specific requests from the reviewers that you believe are technically impossible or unlikely to yield a meaningful outcome.

When revising your paper:

* include a point-by-point response to the reviewers and to any editorial suggestions

* please underline/highlight any additions to the text or areas with other significant changes to facilitate review of the revised manuscript

* address the points listed described below to conform to our open science requirements

* ensure it complies with our general format requirements as set out in our guide to authors at www.nature.com/naturemethods

* resubmit all the necessary files electronically by using the link below to access your home page

[Redacted] This URL links to your confidential home page and associated information about manuscripts you may have submitted, or that you are reviewing for us. If you wish to forward this email to co-authors, please delete the link to your homepage.

We hope to receive your revised paper within 6 weeks. If you cannot send it within this time, please let us know. In this event, we will still be happy to reconsider your paper at a later date so long as nothing similar has been accepted for publication at Nature Methods or published elsewhere.

OPEN SCIENCE REQUIREMENTS

REPORTING SUMMARY AND EDITORIAL POLICY CHECKLISTS
When revising your manuscript, please update your reporting summary and editorial policy checklists.

Reporting summary: https://www.nature.com/documents/nr-reporting-summary.zip
Editorial policy checklist: https://www.nature.com/documents/nr-editorial-policy-checklist.zip

If your paper includes custom software, we also ask you to complete a supplemental reporting summary.

Software supplement: https://www.nature.com/documents/nr-software-policy.pdf

Please submit these with your revised manuscript. They will be available to reviewers to aid in their evaluation if the paper is re-reviewed. If you have any questions about the checklist, please see http://www.nature.com/authors/policies/availability.html or contact me.

Please note that these forms are dynamic 'smart pdfs' and must therefore be downloaded and completed in Adobe Reader. We will then flatten them for ease of use by the reviewers. If you would like to reference the guidance text as you complete the template, please access these flattened versions at http://www.nature.com/authors/policies/availability.html.

IMAGE INTEGRITY
When submitting the revised version of your manuscript, please pay close attention to our Digital Image Integrity Guidelines and to the following points below:

-- that unprocessed scans are clearly labelled and match the gels and western blots presented in figures.
-- that control panels for gels and western blots are appropriately described as loading on sample processing controls
-- all images in the paper are checked for duplication of panels and for splicing of gel lanes.

Finally, please ensure that you retain unprocessed data and metadata files after publication, ideally archiving data in perpetuity, as these may be requested during the peer review and production process or after publication if any issues arise.

DATA AVAILABILITY
We strongly encourage you to deposit all new data associated with the paper in a persistent repository where they can be freely and enduringly accessed. We recommend submitting the data to discipline-specific and community-recognized repositories; a list of repositories is provided here: http://www.nature.com/sdata/policies/repositories

All novel DNA and RNA sequencing data, protein sequences, genetic polymorphisms, linked genotype and phenotype data, gene expression data, macromolecular structures, and proteomics data must be deposited in a publicly accessible database, and accession codes and associated hyperlinks must be provided in the “Data Availability” section.

Refer to our data policies here: https://www.nature.com/nature-research/editorial-policies/reporting-standards#availability-of-data

To further increase transparency, we encourage you to provide, in tabular form, the data underlying the graphical representations used in your figures. This is in addition to our data-deposition policy for specific types of experiments and large datasets. For readers, the source data will be made accessible directly from the figure legend. Spreadsheets can be submitted in .xls, .xlsx or .csv formats. Only one (1) file per figure is permitted: thus if there is a multi-paneled figure the source data for each panel should be clearly labeled in the csv/Excel file; alternately the data for a figure can be included in multiple, clearly labeled sheets in an Excel file. File sizes of up to 30 MB are permitted. When submitting source data files with your manuscript please select the Source Data file type and use the Title field in the File Description tab to indicate which figure the source data pertains to.

Please include a “Data availability” subsection in the Online Methods. This section should inform readers about the availability of the data used to support the conclusions of your study, including accession codes to public repositories, references to source data that may be published alongside the paper, unique identifiers such as URLs to data repository entries, or data set DOIs, and any other statement about data availability. At a minimum, you should include the following statement: “The data that support the findings of this study are available from the corresponding author upon request”, describing which data is available upon request and mentioning any restrictions on availability. If DOIs are provided, please include these in the Reference list (authors, title, publisher (repository name), identifier, year). For more guidance on how to write this section please see: http://www.nature.com/authors/policies/data/data-availability-statements-data-citations.pdf

CODE AVAILABILITY
Please include a “Code Availability” subsection in the Online Methods which details how your custom code is made available. Only in rare cases (where code is not central to the main conclusions of the paper) is the statement “available upon request” allowed (and reasons should be specified).

We request that you deposit code in a DOI-minting repository such as Zenodo, Gigantum or Code Ocean and cite the DOI in the Reference list. We also request that you use code versioning and provide a license.

For more information on our code sharing policy and requirements, please see:

https://www.nature.com/nature-research/editorial-policies/reporting-standards#availability-of-computer-code

SUPPLEMENTARY PROTOCOL
To help facilitate reproducibility and uptake of your method, we ask you to prepare a step-by-step Supplementary Protocol for the method described in this paper. We encourage authors to share their step-by-step experimental protocols on a protocol sharing platform of their choice and report the protocol DOI in the reference list. Nature Research's Protocol Exchange is a free-to-use and open resource for protocols; protocols deposited in Protocol Exchange are citable and can be linked from the published article. More details can found at www.nature.com/protocolexchange/about.

ORCID
Nature Methods is committed to improving transparency in authorship. As part of our efforts in this direction, we are now requesting that all authors identified as ‘corresponding author’ on published papers create and link their Open Researcher and Contributor Identifier (ORCID) with their account on the Manuscript Tracking System (MTS), prior to acceptance. This applies to primary research papers only. ORCID helps the scientific community achieve unambiguous attribution of all scholarly contributions. You can create and link your ORCID from the home page of the MTS by clicking on ‘Modify my Springer Nature account’. For more information please visit please visit www.springernature.com/orcid.

Please do not hesitate to contact me if you have any questions or would like to discuss these revisions further. We look forward to seeing the revised manuscript and thank you for the opportunity to consider your work.

Best regards,
Lei

Lei Tang, Ph.D.
Senior Editor
Nature Methods

Reviewers' Comments:

Reviewer #1:
Remarks to the Author:
We thank the authors for attempting to address our points.

While the authors have addressed some points, major limitations and concerns remain and we thus remain unconvinced of this approach. We outline the major points below:

1 - Comparison to other methods:
1.1 - only CellChat (which is from the authors themselves, and does not take into account space) and Giotto (a general-purpose toolbox for spatial data) are used. This is very limited given the large number of methods, increasingly with a focus on spatial data. By the way, CellPhoneDB does take into account space in its latest version (authors refer to the squidpy implementation, but there is an original one). And there are many other methods in particular published in the last months.

1.2 - In addition authors write "COMMOT achieves stronger correlation than the other methods" but rather it seems like a "comparable" correlation to the one of CellChat. This is rather a disappointing result, given that they apply CellChat on visium clusters and it doesn't take space into account (Supp Fig 21). So it is not convincing that there is added value of COMMOT.

2 - We were not able to run the code. So we could not truly test the tool. In this regard, it would be nice to have the package on e.g. pip, as trying to figure out why dependencies are not installing from .txt file is cumbersome and will also make that the tool is not broadly used.

3 - While authors have added some anecdotical data on proteins, this does not support enough their approach, which is based on the assumption of RNA is proportional to protein, and aspects such as localization or protein modification can be ignored. Given this major assumption, it seems too optimistic to expect that one can capture complex processes that happen at the protein level in a quantitative, spatially-localised, and dynamic manner such as competition.
This assumption is also the foundation for the simulated data used by the authors (‘This simulation assumes that protein abundance is proportional to RNA abundance.’); this justification is unconvincing; to show that their method can infer communication from gene expression data, their model ought to include explicitly the proteins besides the RNA, and the actual signaling as well.

4- some important aspects of COMMOT are unclear and should be better explained;
- How do they account for the competition between the ligands?
- How do they determine the distance above which the cost is infinite?

- How does COMMOT handle complexes? Authors write "The heteromeric ligand-receptor pairs in CellChatDB were converted to pairs of individual subunits" so it seems that it does not work with complexes?

Minor:
5- Authors used now CellChatDB, which is a natural choice for them given that is from the authors, but there are many databases, including meta-databases. The choice of database can significantly affect results.

6- why did the authors choose in particular scSeqComm, from the many resources/tools that include intracellular processes?

Reviewer #2:
Remarks to the Author:
In this revision, the authors addressed most of my comments and optimized COMMOT to infer cell-cell communications based on optimal transport. COMMOT is capable of 1) preserving the original units without the need for normalization, 2) enforcing spatial range limits of signaling, and 3) handling the cases of multiple competing species. Overall, the method is novel in concept. The biological "validation" is critical.

A few remaining points:
1. One major challenge is that many predictions do not have experimental and biological ground truth. While I appreciate the authors tried to test COMMOT on many different spatial datasets, it is hard to interpret the biological relevance. The authors claimed that these predictions are supported by prior knowledge. In most cases, the authors only cited the general functions of those ligand-receptor pairs and signaling pathways. The authors need to revise and connect prior knowledge to the cell type specific context in each tissue. It's difficult to interpret and judge whether the predictions make sense or cell type specific in each tissue. For example, the OXT pathway in MERFISH data, MK, ANNEXIN, IGF, ANGPT signaling in the STARmap placenta data, WNT in SeqFISH+. Are there reports supporting cell type specific interactions in these tissues predicted by COMMOT?
2. I appreciate the authors tested COMMOT in various tissues and I'm glad to see several examples. I suggest the authors focus on the brain because there are multiple spatial datasets available. For example, the new BRAIN Initiative datasets for benchmarking. In the brain, for the same ligand-receptor pairs and signaling pathways, 1) do we see similar results across datasets (MERFISH, STARmap, SeqFISH+, Slide-seqv2)? 2) does COMMOT agree or disagree with other methods for the same ligand-receptor pairs and signaling pathways? It's hard to interpret the STARmap placenta data. Why not test on STARmap brain data?

3. In Figure 3e, the spatial distance of the ligand-receptor pair GAS6 and TYRO3 doesn't seem close or "optimal".

Reviewer #3:
Remarks to the Author:
Review comments on NMETH-A47293A-Z (second round)

The authors improved the manuscript in many ways; in particular, they commented on my concerns regarding connections to earlier methods. These connections are made clear in their revised version and sufficient practical comparisons are added. They also addressed my comments regarding benchmarks with earlier methods. The new benchmarks are informative and improve the manuscript.

With the revised manuscript, COMMOT represents a useful addition to the world of single-cell tools. It is based on a sound mathematical formulation, connections to earlier methods are made clear, and advantages over competing methods are demonstrated. The implementation is scalable by exploiting sparsity.

Below, I comment on a few points that I think deserve some more attention; the remaining points have been addressed to my satisfaction.
Major comments
Re point 1: Comparison with unbalanced and partial OT. This is a nice investigation into alternative OT variants which I think improves the manuscript. The updated main text explains the problem much more precisely with respect to earlier OT variants. I just wonder why in line 88, the authors suddenly start to discuss competition; they should make it more clear whether this is also a problem with existing OT approaches or with prior CCC methods.

The new Supplementary Figs. 7-9 are convincing. However, the authors write in the response letter "When the total amount of ligand and receptor expressions are significantly different, unbalanced OT generates artificial connections due to the KL divergence regularization." As the authors correctly note in the main text, the choice of KL divergence is not a must for UOT, it's just a common choice. It's fine to use KL divergence here for comparison as it is very popular, however, it should just be made clear that there are other possible choices of divergences.

In Supplementary Fig. 10, the examples are nice, however, there should be one extra evaluation that shows that the LR pairs aren't cherrypicked and the phenomena are observed widely across many pairs. Some summary statistic that generalizes the result (for all three methods) to all/many LR pairs would be helpful.

Re point 2: Connection to multimarginal OT. The authors clarified this point and updated the manuscript accordingly; the updated paragraph in the discussion is more precise.

With respect to Equation (1) vs. (3) in the methods section, the authors should emphasize which formulation is the final one that was used to produce the results in the manuscript.

Re point 5: Benchmarks. Casting the problem in terms of correlation is fine; however, wouldn't it have been equally possible to stay within the classification setting and to look at AUC values, just defining false positives as all genes that are not in the database?

Re point 6: Scalability. The authors clarify this point, the linear scalability of their algorithm in the number of samples is impressive. This should be made more clear in the last paragraph of the results section by explicitly stating how compute time scales in terms of spatial locations.

I don't seem to have access to the new Supplementary Fig. 26 so I cannot comment on it.

Re point 7: Implementation. The authors have clarified why they need a custom implementation and cannot use POT. It is unreasonable to ask for this, but just as a general suggestion, the community would benefit more from implementing support for sparse matrices in POT and then interfacing to that from your own package, rather than implementing this in the first place in your own package. It's fine this way as well though.

Re point 8: Testing. The authors included tests in their software, this will make it more valuable to the community. Upon publication, they should also set up CI on GitHub using any available tool to enable the community to contribute while making sure core functionality does not change.

Author Rebuttal, first revision:

Reviewer #1:

We thank the authors for attempting to address our points.

While the authors have addressed some points, major limitations and concerns remain and we thus remain unconvinced of this approach. We outline the major points below:

Major points

1.1. Only CellChat (which is from the authors themselves, and does not take into account space) and Giotto (a general-purpose toolbox for spatial data) are used. This is very limited given the large number of methods, increasingly with a focus on spatial data. By the way, CellPhoneDB does take into account space in its latest version (authors refer to the squidpy implementation, but there is an original one). And there are many other methods in particular published in the last months.

Response: We thank the reviewer for pointing us to other spatial data analysis methods. CCC analysis in spatial data is a fast-evolving field and most of the mentioned methods are new, published after submission of this manuscript. Following the reviewer's suggestion, we have added comparison to the recent CellPhoneDB version that indeed takes into account spatial information. In the revised manuscript, we added the description of CellPhoneDB v3 to the third paragraph of the Introduction section: *"CellPhoneDB v3 restricts interactions to cell clusters within the same microenvironment defined based on spatial information."* The newly added comparison in downstream gene correlation has been added as new Supplementary Figure 29 and the corresponding illustrations have been added as new Supplementary Figures 35-36. For the result in new Supplementary Fig. 29, CellPhoneDB v3 takes cluster-level spatial proximity. In the numerical experiment, the distance between clusters was quantified as the average distance between cells/spots from two clusters. Three cluster-level distance cutoffs were then tested where only a few pairs of clusters, moderately many cluster pairs, or most of the cluster pairs are considered able to interact spatially.

1.2. In addition authors write "COMMOT achieves stronger correlation than the other methods" but rather it seems like a "comparable" correlation to the one of CellChat. This is rather a

disappointing result, given that they apply CellChat on visium clusters and it doesn't take space into account (Supp Fig 21). So it is not convincing that there is added value of COMMOT.

Response: The median correlations of COMMOT/CellChat on the three datasets we tested are 0.56/0.12, 0.57/0.47, and 0.59/0.41 which clearly indicates stronger correlations by COMMOT over CellChat. In the revised manuscript, we have added a comparison to the recent version of CellPhoneDB v3 which considers spatial information. We show that COMMOT achieves stronger correlations compared to CellPhoneDB on Visium datasets and comparable correlations on the seqFISH+ dataset. Altogether, these results show improved performance of COMMOT over the three other popular methods in the context of downstream gene correlations.

In addition to stronger correlations between downstream genes and inferred signaling, COMMOT offers many other added values. For example, in supplementary figures 35-36, we show the signaling activities uniquely identified by COMMOT which are not captured by the alternative method. COMMOT also provides unique utilities such as visualizing the spatial direction of signalings.

2. We were not able to run the code. So we could not truly test the tool. In this regard, it would be nice to have the package on e.g. pip, as trying to figure out why dependencies are not installing from .txt file is cumbersome and will also make that the tool is not broadly used.

Response: We have now published the package named "commot" to PyPI and made the source code available on GitHub (https://github.com/zcang/COMMOT). To further make sure the usability of the package, we have set up continuous integration pipelines on GitHub, which test the package in Linux and Mac environments with different Python versions.

3. While authors have added some anecdotical data on proteins, this does not support enough their approach, which is based on the assumption of RNA is proportional to protein, and aspects such as localization or protein modification can be ignored. Given this major assumption, it seems too optimistic to expect that one can capture complex processes that happen at the protein level in a quantitative, spatially-localized, and dynamic manner such as competition.

This assumption is also the foundation for the simulated data used by the authors ('This simulation assumes that protein abundance is proportional to RNA abundance.'); this justification is unconvincing; to show that their method can infer communication from gene expression data,

their model ought to include explicitly the proteins besides the RNA, and the actual signaling as well.

Response: We completely agree with the reviewer that the correlation between mRNA levels and protein levels has been a matter of debate for many years[1-3], and efforts to characterize and resolve transcriptome to proteome relationships are currently ongoing. Indeed, protein copy number and abundance in eukaryotic cells have been shown to be determined at the level of translation[4], and are further affected by post-translational modifications including glycosylation, addition of lipid groups, degradation, making linear correlations between transcript and protein levels difficult to establish. Further, proteomic analysis by HP/LC mass spectrometry, a popular and widely accessible method, is inherently noisy (especially when applied to single cells), compounding this problem. Recently, highly accurate proteomic techniques like Parallel Reaction Monitoring (PRM) have been developed, establishing new standards for protein concentration measurements with low error rates[5]. Indeed, PRM has recently been used to assess the correlation between mRNA and protein levels across multiple tissues and established cell lines[6], finding strong, reasonable correlations between mRNAs and their respective gene products (Pearson's $r \sim 0.60$ log-log scale) that becomes even stronger when using gene-specific transcript-to-protein conversion rates and is consistent with previous studies using less sensitive methods[7]. Indeed, our inference methods are not impervious to false positives, especially when considering post-translational modifications that may vary not only across tissues but also across cell types. However, we show that for an important and biologically-relevant number of signaling pathways such as WNT and FGF, COMMOT can be used as a reliable exploratory tool that will only improve with time as the fast-emerging spatial proteomics tools are being developed.

To further clarify this matter, we have dedicated a new paragraph in the Discussion section in the revised manuscript:

"Having said that, we acknowledge that false positives in our inferred CCC are inherently possible because spatial transcriptomics data does not directly represent protein abundancy and our method cannot capture protein-specific modifications, such as protein phosphorylation, glycosylation, proteolytic cleavage into fragments, dimerization, that certainly affect their signaling functions and, thus, CCC mechanisms, that COMMOT aims to infer. The reliability of CCC predictions is expected to significantly improve once the emerging spatial proteomics approaches will mature."

Lastly, we want to emphasize that the above limitation affects every RNA data-based computational inference methodology. Software predictions on cellular functions, including on their signaling functions, that derive from mRNA analysis should only serve as predictions (albeit highly valuable) and functional experiments including on protein products of mRNAs of interest are required to validate them.

1 Anderson, L. & Seilhamer, J. A comparison of selected mRNA and protein abundances in human liver. *Electrophoresis* **18**, 533-537, doi:10.1002/elps.1150180333 (1997).

2 Maier, T., Guell, M. & Serrano, L. Correlation of mRNA and protein in complex biological samples. *FEBS Lett* **583**, 3966-3973, doi:10.1016/j.febslet.2009.10.036 (2009).

3 Lawless, C. *et al.* Direct and Absolute Quantification of over 1800 Yeast Proteins via Selected Reaction Monitoring. *Mol Cell Proteomics* **15**, 1309-1322, doi:10.1074/mcp.M115.054288 (2016).

4 Schwanhausser, B. *et al.* Global quantification of mammalian gene expression control. *Nature* **473**, 337-342, doi:10.1038/nature10098 (2011).

5 Rauniyar, N. Parallel Reaction Monitoring: A Targeted Experiment Performed Using High Resolution and High Mass Accuracy Mass Spectrometry. *Int J Mol Sci* **16**, 28566-28581, doi:10.3390/ijms161226120 (2015).

6 Edfors, F. *et al.* Gene-specific correlation of RNA and protein levels in human cells and tissues. *Mol Syst Biol* **12**, 883, doi:10.15252/msb.20167144 (2016).

7 Wilhelm, M. *et al.* Mass-spectrometry-based draft of the human proteome. *Nature* **509**, 582-587, doi:10.1038/nature13319 (2014).

4. Some important aspects of COMMOT are unclear and should be better explained;

- How do they account for the competition between the ligands?

- How do they determine the distance above which the cost is infinite?

- How does COMMOT handle complexes? Authors write "The heteromeric ligand-receptor pairs in CellChatDB were converted to pairs of individual subunits" so it seems that it does not work with complexes?

Response: We thank the reviewer for the detailed suggestions.

1) In the revised manuscript, we have added the description of how competitions are accounted in the second paragraph of the section *"Results – Overview of COMMOT"* which reads *"The competitions between species and cells are addressed in the optimization problem that a receptor species or cell has a limited capacity of interaction, so a higher inferred interaction with one particular ligand species or cell will reduce the interaction score with other ligand species or cells."* This point is further illustrated in Figure

2c and the details in the formulation accounting for competition can be found in Equation (1).

2) In the revised manuscript, we have added a paragraph in Discussion about the choice of spatial distance cutoff: *"The spatial distance constraint in the model is used to capture the effect of ligand diffusivity, which is determined by several factors, including protein weight and tortuosity of extra-cellular space. When screening all ligand-receptor pairs, it is difficult to estimate this parameter for each pair accurately. In our model, the local short-range interactions are emphasized even when the spatial distance range is increased (Supplementary Fig. 27). Thus, when screening many ligand-receptor pairs, a uniform and relatively large spatial distance limit may be used in the model to avoid missing important interactions. Once the important interactions are identified, using an accurate estimation of this parameter would further refine the prediction to remove some false positive CCC links."* For example, we used a distance cutoff of 1000 μm for the Visium examples in this work, which is roughly equivalent to the size of 10 to 20 mammalian cells as an upper bound of effective range of diffusive ligands.

3) Sorry for the confusion. Similar to CellPhoneDB and CellChat, COMMOT uses the minimum of the units of complexes by default with the choice of using the average. In the main function of the package "commot.tl.spatial_communication()", the users can set the parameter "heteromeric_rule" to either "min" or "ave". In the revised manuscript, a clarification of this is added to the section *"Methods – COMMOT model"* which reads *"When the ligands or receptors contain heteromeric units, the minimum of units is used by default to represent the amount of ligand or receptor."* The sentence mentioned by the reviewer were in the section "Methods - CellChat and Giotto analysis" where we described how we used Giotto that does not consider heteromeric units with CellChatDB, a database considering multiple units.

Minor points

5. Authors used now CellChatDB, which is a natural choice for them given that is from the authors, but there are many databases, including meta-databases. The choice of database can significantly affect results.

Response: Indeed, the total CCC score across all ligand-receptor pairs between cells or clusters could be very different when different databases are used. However, the results for the same

individual ligand-receptor pairs should be similar for common ligand-receptor pairs in the different databases used.

In this revision we have added CellPhoneDB v4.0 as another choice for the built-in function "commot.pp.ligand_receptor_database()" that creates ligand-receptor lists. We also keep CellChatDB as a default choice, given it has been shown to work well in many case studies (>400 citations after 1.5 years of publication and selected as 2021 Top 25 Life and Biological Sciences Articles in Nature Communications), In practice, COMMOT can take any user-defined ligand-receptor lists in addition to the ones generated by the built-in function. The users could use any database suitable for their system of interest.

6. Why did the authors choose in particular scSeqComm, from the many resources/tools that include intracellular processes?

Response: There are indeed many resources on intracellular processes. We chose scSeqComm since it combines multiple major resources including KEGG, Reactome, TRRUST, HTRIdb, and RegNetwork. Also, scSeqComm was published April 2022, so it is one of the up-to-date meta databases. In the revised manuscript, we have added a sentence to clarify to paragraph 3 of *"Results - Robust identification of spatial signaling direction and downstream targets"* which reads *"scSeqComm is used because it combines multiple major resources including Reactome, TTRUST, and RegNetwork."*

Reviewer #2:

In this revision, the authors addressed most of my comments and optimized COMMOT to infer cell-cell communications based on optimal transport. COMMOT is capable of 1) preserving the original units without the need for normalization, 2) enforcing spatial range limits of signaling, and 3) handling the cases of multiple competing species. Overall, the method is novel in concept. The biological "validation" is critical.

Major points

1. One major challenge is that many predictions do not have experimental and biological ground truth. While I appreciate the authors tried to test COMMOT on many different spatial datasets, it is hard to interpret the biological relevance. The authors claimed that these predictions are supported by prior knowledge. In most cases, the authors only cited the general functions of those ligand-receptor pairs and signaling pathways. The authors need to revise and connect prior knowledge to the cell type specific context in each tissue. It's difficult to interpret and judge whether the predictions make sense or cell type specific in each tissue. For example, the OXT pathway in MERFISH data, MK, ANNEXIN, IGF, ANGPT signaling in the STARmap placenta data, WNT in SeqFISH+. Are there reports supporting cell type specific interactions in these tissues predicted by COMMOT?

Response: Thank you for the good point. In the revised manuscript, we have added cell type-specific descriptions and found several supporting biological studies. These added detailed descriptions are listed below:

1) (MERFISH mouse hpr data) *"Among the signaling pathways available in the data, oxytocin (OXT) signaling, an important pathway modulating social behaviors, was found to be most active. Self-modulation of excitatory neurons and modulation of inhibitory neurons by excitatory neurons through OXT signaling were identified across all the slices (Fig. 4b, Supplementary Fig. 17-18) a result consistent with the known major functions of OXT signaling[46]. Further analysis identifies the local regions of high OXT signaling activity and the spatial direction of OXT signaling (Fig. 4c), which corresponds with protein staining of OXT and its receptor[47].*

   46. Froemke, R. C. & Young, L. J. Oxytocin, Neural Plasticity, and Social Behavior. Annu. Rev. Neurosci. 44, 359–381 (2021).

47. Warfvinge, K., Krause, D. & Edvinsson, L. The distribution of oxytocin and the oxytocin receptor in rat brain: Relation to regions active in migraine. J. Headache Pain 21, 1–13 (2020)."

2) (STARmap mouse placenta) *"In addition, it was found that signaling through the ligand-receptor pair Igf2-Igf2r is active in the labyrinth region and in endothelial cells both of which are consistent with our prediction[50]. MK signaling was inferred to be active in trophoblast cells, consistent with the previous finding of SDC1 and SDC4 in trophoblast cells[51,52] (Supplementary Fig. 20).*

   50. Sandovici, I. et al. The imprinted Igf2-Igf2r axis is critical for matching placental microvasculature expansion to fetal growth. Dev. Cell 57, 63-79.e8 (2022).

   51. Marchese, M. J., Li, S., Liu, B., Zhang, J. J. & Feng, L. Perfluoroalkyl Substance Exposure and the BDNF Pathway in the Placental Trophoblast. Front. Endocrinol. (Lausanne). 12, 916 (2021).

   52. Jeyarajah, M. J., Jaju Bhattad, G., Kops, B. F. & Renaud, S. J. Syndecan-4 regulates extravillous trophoblast migration by coordinating protein kinase C activation. Sci. Reports 2019 91 9, 1–15 (2019)."

3) (seqFISH+ mouse sscortex) *"COMMOT predicted significant WNT signaling in neurons (Supplementary Fig. 21), correlating well with critical roles of WNT signaling in neuronal migration and activity in the somatosensory cortex[53].*

   53. Bocchi, R. et al. Perturbed Wnt signaling leads to neuronal migration delay, altered interhemispheric connections and impaired social behavior. Nat. Commun. 2017 81 8, 1–15 (2017)."

4) In the newly added analysis of three cortex datasets, we also discussed the identified cell-type specific CCCs supported by several previous biological studies. Please see reply to comment 2 below for details of this part.

Three new Supplementary Figures 18, 20, 21 have been added with these cell-type specific descriptions.

2. I appreciate the authors tested COMMOT in various tissues and I'm glad to see several examples. I suggest the authors focus on the brain because there are multiple spatial datasets available. For example, the new BRAIN Initiative datasets for benchmarking. In the brain, for the same ligand-receptor pairs and signaling pathways, 1) do we see similar results across datasets (MERFISH, STARmap, SeqFISH+, Slide-seqv2)? 2) does COMMOT agree or disagree with other

methods for the same ligand-receptor pairs and signaling pathways? It's hard to interpret the STARmap placenta data. Why not test on STARmap brain data?

Response: Thank you for the insightful suggestion.

1) We have added a joint analysis of Visium, STARmap, and seqFISH+ data of mouse cortex. The new analysis is added as Paragraph 5 to *"Results - Signaling analysis in high spatial resolution ST data"* and new Supplementary Figures 22-24 in the revised manuscript. The added results are shown below:
   "We further jointly analyzed mouse cortex examined by different technologies including Visium, seqFISH+, and STARmap. Across the three datasets, AGT signaling was identified in the neuron cells. Spatially, layers L2-3 region was identified as a strong receiver of AGT ligands across the three datasets. Interestingly, a striped pattern was observed where strong signals within individual layers form stripes, while weak signals between these layers form gaps. Strong AGT signaling activity among oligodendrocytes was also identified in both STARmap and seqFISH+ data. (Supplementary Fig. 22). In both Visium and seqFISH+ cortex datasets, we inferred WNT signaling to be active across different cortical layers. Among these layers, we identified WNT signaling to be relatively low, compared to other layers, in layer 5 in both datasets. This is in line with a previous study that disruptions to WNT signaling in Lrp6 mutant mice leads to cortical hypoplasia and reduced proliferation in neurons in layers 2-4 and layer 6, but with neurons in layer 5 remaining relatively unchanged[1] (Supplementary Fig. 23). TAC (tachykinin neuropeptide family) signaling was consistently found in both Visium and STARmap cortex data to be active in non-neuronal cells and inhibitory neurons, especially in somatostatin-expressing neurons (Sst), because of the high expression of tachykinin receptor-3 in Sst neurons[2] (Supplementary Fig. 24)."
   1. Zhou, C.J., Borello, U., Rubenstein, J.L.R. & Pleasure, S.J. Neuronal production and precursor proliferation defects in the neocortex of mice with loss of function in the canonical Wnt signaling pathway. Neuroscience 142, 1119-1131 (2006).
   2. Smith, S.J. et al. Single-cell transcriptomic evidence for dense intracortical neuropeptide networks. eLife 8, e47889 (2019).
2) We have compared the properties across the four methods COMMOT, CellChat, Giotto, and CellPhoneDB v3 (newly added in this revision) using two datasets and illustrated the different identified signaling in Supplementary Figures 31-36 and in paragraph 4 of

"Results - Robust identification of spatial signaling direction and downstream targets" which is listed below.

"Finally, using Visium human breast cancer and mouse brain datasets, we studied the difference between COMMOT and the three other methods. When assessing the distributions of inter-cluster spatial distance of the identified significant CCCs, the significant CellChat interactions have a relative uniform distribution of the spatial distances. This is expected since CellChat was designed for scRNA-seq data and spatial organization is not considered in the method. Both COMMOT and Giotto prioritize CCC between spatially close clusters while COMMOT identifies more CCC links that are beyond the neighboring cells because Giotto uses cell-contact graph that might limit the range of inferred CCC. CellPhoneDB v3 identifies more CCC links than COMMOT potentially due to its consideration of inter-cluster distances rather than distance among individual cells (Supplementary Figs. 31-36). In the comparison to CellChat, COMMOT uniquely detects CCC that are not differentially expressed in clusters but spatially significant (Supplementary Figs. 31-32). Giotto characterizes the average expression of ligands and receptors of spatially neighboring spots without providing the role for each spot in CCC. COMMOT identifies each spot as a sender, receiver or both in CCC with interpretable results (Supplementary Figs. 33-34). COMMOT identifies CCC that is locally significant which CellPhoneDB v3 may neglect due to its consideration of expression level of entire clusters (Supplementary Figs. 35-36)."

3. In Figure 3e, the spatial distance of the ligand-receptor pair GAS6 and TYRO3 doesn't seem close or "optimal".

Response: Thank you for bringing this to our attention and we apologize for this confusion. In an effort to highlight all detected signals in the GAS6-TYRO3 FISH experiment, we labeled only GAS6 mRNAs that may have been difficult to see, leaving others unlabeled, including those localized to the granular layer along with TYRO3. We have improved the figure for clarity (shown below), only labeling GAS6 mRNAs that spatially co-localize or that are within significant optimal distance to detected TYRO3 mRNAs in the granular layer as in the PROS1-TYRO3 figure panel.

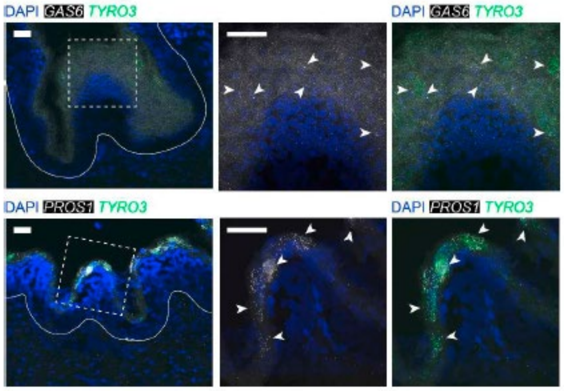

Updated Figure 3 Panel e. Fluorescent in situ hybridization against RNA molecules for predicted L-R interactions in human epidermis (solid white outline; regions of interest are marked by a white dashed square). Top row shows expression patterns of Gas6 (white) and Tyro3 (green); bottom row shows expression patterns for Pros1 (white) and Tyro3 (green). In both cases, L-R signals that localize to stratum granulosum and are in close proximity to each other are indicated with white arrowheads.

Reviewer #3:

The authors improved the manuscript in many ways; in particular, they commented on my concerns regarding connections to earlier methods. These connections are made clear in their revised version and sufficient practical comparisons are added. They also addressed my comments regarding benchmarks with earlier methods. The new benchmarks are informative and improve the manuscript.

With the revised manuscript, COMMOT represents a useful addition to the world of single-cell tools. It is based on a sound mathematical formulation, connections to earlier methods are made clear, and advantages over competing methods are demonstrated. The implementation is scalable by exploiting sparsity.

Below, I comment on a few points that I think deserve some more attention; the remaining points have been addressed to my satisfaction.

Major points

1. Re point 1: Comparison with unbalanced and partial OT. This is a nice investigation into alternative OT variants which I think improves the manuscript. The updated main text explains the problem much more precisely with respect to earlier OT variants. I just wonder why in line 88, the authors suddenly start to discuss competition; they should make it more clear whether this is also a problem with existing OT approaches or with prior CCC methods.

The new Supplementary Figs. 7-9 are convincing. However, the authors write in the response letter "When the total amount of ligand and receptor expressions are significantly different, unbalanced OT generates artificial connections due to the KL divergence regularization." As the authors correctly note in the main text, the choice of KL divergence is not a must for UOT, it's just a common choice. It's fine to use KL divergence here for comparison as it is very popular, however, it should just be made clear that there are other possible choices of divergences.

In Supplementary Fig. 10, the examples are nice, however, there should be one extra evaluation that shows that the LR pairs aren't cherrypicked and the phenomena are observed widely across many pairs. Some summary statistic that generalizes the result (for all three methods) to all/many LR pairs would be helpful.

Response: Thank you for detailed suggestions. We have modified the manuscript and performed new analyses to address these points.

1) In the revised manuscript, we have rearranged paragraph 4 in the Introduction section. Competition between different ligand and receptor species has not been addressed in the existing CCC methods. In the context of optimal transport, addressing this phenomenon requires consideration of multiple ligand and receptor species at the same time. Among these species, some pairs that do not interact introduce constraints on transport plans fixing the corresponding elements in the coupling matrix to zero. This constraint can be enforced by unbalanced or partial optimal transport by setting the transport cost to infinity at these locations. However, both methods lead to marginal distributions (bound ligand or receptor) exceeding the initial distributions, the available ligand or receptor. Currently, other CCC methods are unable to deal with such competition. In revision, we have clarified this point: *"Such competition between multiple species is ubiquitous, a critical physical process ignored in the existing CCC inference methods."* We have also clarified the limitations of unbalanced OT and partial OT in addressing this difficulty: "Specifically, unbalanced OT in its common form uses KL divergence as a soft constraint on marginal distribution preservation. this approach may result in the total coupled species significantly exceeding the total amount of either ligand or receptor initially available. On the other hand, partial OT, requires an additional parameter, the total coupled mass, which is usually difficult to estimate in the context of CCC inference."
2) We have added a sentence to clarify in the paragraph 4 of the *"Results - Accurate reconstruction of simulated spatial CCC"*: "In unbalanced optimal transport, the commonly used KL divergence is adapted in this comparison while other divergence forms may be incorporated as well."
3) In the revision, we have evaluated this in two datasets with all the ligand-receptor pairs in the CellChatDB database. The results have been added as new Supplementary Figure 11 which is also shown below.

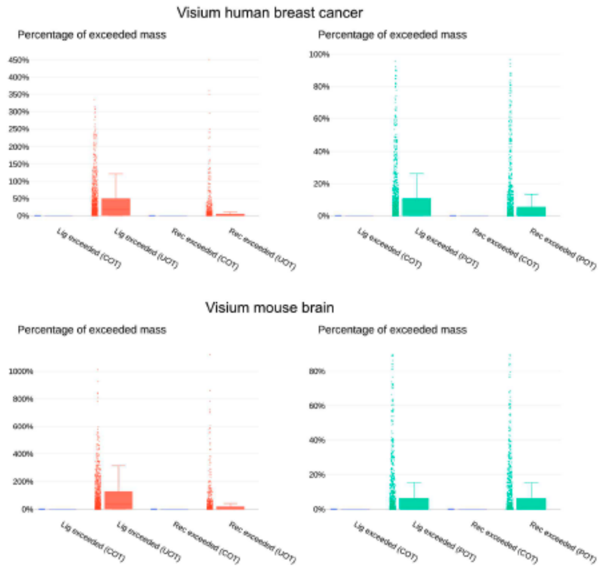

New Supplementary Figure 11 Inferred sent or received signal exceeding the amount of ligand or receptors. Each dot represents a ligand-receptor pair. The commonly used KL divergence-based unbalanced optimal transport is used.

2. Re point 2: Connection to multimarginal OT. The authors clarified this point and updated the manuscript accordingly; the updated paragraph in the discussion is more precise.

With respect to Equation (1) vs. (3) in the methods section, the authors should emphasize which formulation is the final one that was used to produce the results in the manuscript.

Response: Thank you for the suggestion. We have added the following sentence in the paragraph 2 of *"Methods – Collective optimal transport algorithm"*: *"The formulation in Eq. (2) solved by the algorithm in Eq. (3) was used to generate the results in this study."*

3. Re point 5: Benchmarks. Casting the problem in terms of correlation is fine; however, wouldn't it have been equally possible to stay within the classification setting and to look at AUC values, just defining false positives as all genes that are not in the database?

Response: This is a very good point. Ideally it would be nice to cast this into a classification problem. However, the biological processes of gene expression and its regulation are complex. For example, a gene that is not directly regulated by a ligand-receptor pair could still be highly correlated to the activity of the related signaling. Also, the database of gene expression regulation is still expanding, and our knowledge is far from complete. At this moment, it is more interpretable to use the correlation approach for comparisons. Thus, we only state that COMMOT achieves stronger correlation without mentioning this as a benchmark. Benchmarking CCC inference methods computationally and experimentally is still a challenging and ongoing research topic. In this revision, we have added a clarification in paragraph 3 of *"Results - Robust identification of spatial signaling direction and downstream targets"* which reads *"This evaluation can be further improved if more complete knowledge of gene regulations is available. With such list, one may also formulate the evaluation as a classification problem."*

4. Re point 6: Scalability. The authors clarify this point, the linear scalability of their algorithm in the number of samples is impressive. This should be made more clear in the last paragraph of the results section by explicitly stating how compute time scales in terms of spatial locations.

I don't seem to have access to the new Supplementary Fig. 26 so I cannot comment on it.

Response: In the revised manuscript, we have made this clearer by changing the last sentence of the Results section to say: "Thus, COMMOT can effectively handle the existing ST datasets since the computing time and the memory usage both scale linearly to the number of spatial locations." We have included the figure on Scalability below showing the linear scalability with respect to the number potential signaling cell pairs that is linearly proportional to the number of cells/spot given the same spatial distance cutoff.

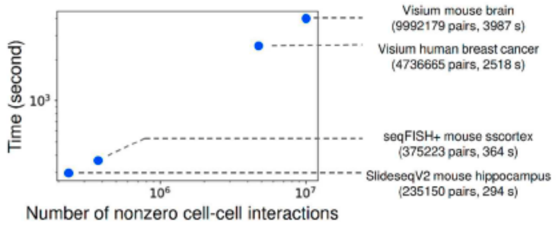

Supplementary Figure 37 CCC inference running time of COMMOT. The running time (tested on a desktop with i7-8700K running Ubuntu 20.04) of the collective optimal transport algorithm of COMMOT (the core CCC inference function) on several datasets. The wall time of computation is plotted against the number of nonzero elements in the interaction networks. Both axes are log transformed.

5. Re point 7: Implementation. The authors have clarified why they need a custom implementation and cannot use POT. It is unreasonable to ask for this, but just as a general suggestion, the community would benefit more from implementing support for sparse matrices in POT and then interfacing to that from your own package, rather than implementing this in the first place in your own package. It's fine this way as well though.

Response: We thank the reviewer for this suggestion. To further clarify, even in the dense form, the exact OT formulation and algorithm used in our study is not available in POT. If our algorithm becomes available on common libraries like POT, we will simplify our current COMMOT codes to call the function from POT. Meanwhile, we have released all the source codes to GitHub (https://github.com/zcang/COMMOT) and all the OT functions are in the folder "_optimal_transport" so readers interested in the OT part could directly use the functions.

6. Re point 8: Testing. The authors included tests in their software, this will make it more valuable to the community. Upon publication, they should also set up CI on GitHub using any available tool to enable the community to contribute while making sure core functionality does not change.

Response: Now we have set up CI on GitHub (https://github.com/zcang/COMMOT) for testing on MacOS and Ubuntu with Python 3.8 and 3.9. We have also published the package to PyPI for the ease of installation.

Decision Letter, second revision:

Dear Dr. Nie,

Thank you for submitting your revised manuscript "Screening cell-cell communication in spatial transcriptomics via collective optimal transport" (NMETH-A47293B). It has now been seen by the original referees again and their comments are attached below. The reviewers find that the paper has improved in revision, and therefore we'll be happy in principle to publish it in Nature Methods, pending

revisions to satisfy the referees' final requests and to comply with our editorial and formatting guidelines.

In particularly, we ask you to provide a rebuttal letter to discuss reviewer #1's concerns on benchmarking comparisons and to address the issue of the COMMOT installation. Please bear in mind that we don't request additional experiments for comparing COMMOT with methods that are published as preprints or in journals while your manuscript was under review or revision with Nature Methods.

We are now performing detailed checks on your paper and will send you a checklist detailing our editorial and formatting requirements in about a week. Please do not upload the final materials and make any revisions until you receive this additional information from us.

TRANSPARENT PEER REVIEW
Nature Methods offers a transparent peer review option for new original research manuscripts submitted from 17th February 2021. We encourage increased transparency in peer review by publishing the reviewer comments, author rebuttal letters and editorial decision letters if the authors agree. Such peer review material is made available as a supplementary peer review file. Please state in the cover letter 'I wish to participate in transparent peer review' if you want to opt in, or 'I do not wish to participate in transparent peer review' if you don't. Failure to state your preference will result in delays in accepting your manuscript for publication.
Please note: we allow redactions to authors' rebuttal and reviewer comments in the interest of confidentiality. If you are concerned about the release of confidential data, please let us know specifically what information you would like to have removed. Please note that we cannot incorporate redactions for any other reasons. Reviewer names will be published in the peer review files if the reviewer signed the comments to authors, or if reviewers explicitly agree to release their name. For more information, please refer to our FAQ page.

Thank you again for your interest in Nature Methods Please do not hesitate to contact me if you have any questions.

Best regards,
Lei

Lei Tang, Ph.D.
Senior Editor
Nature Methods

ORCID

IMPORTANT: Non-corresponding authors do not have to link their ORCIDs but are encouraged to do so. Please note that it will not be possible to add/modify ORCIDs at proof. Thus, please let your co-authors know that if they wish to have their ORCID added to the paper they must follow the procedure described in the following link prior to acceptance:
https://www.springernature.com/gp/researchers/orcid/orcid-for-nature-research

Reviewer #1 (Remarks to the Author):

Although, we agree that the proposed OT algorithm and its application to CCC are promising, to be publisheds, we believe that the manuscript should undergo further revisions. The current manuscript portrays the method as iterative over previous developments, and masks its potential and usability.

In particular, instead of presenting the repetitive applications of their method, the authors should address the lack of comparison to other relevant tools and propose at least a basic working tutorial for their method.

See point by point suggestions below:

● (As an answer to point 1.1) CellPhoneDB v3 simply restricts inference to microenvironments. So, to iterate the same point, unclear why the authors chose to compare their method to CellPhoneDB and CellChat, given that both work at the cluster level and neither takes spatial information into account. Furthemore, the reported performance gain is very underwhelming. Instead, the authors should demonstrate that their tool is superior to tools that work at the single-cell resolution (e.g. SCRIABIN https://www.biorxiv.org/content/10.1101/2022.02.04.479209v1.full) and/or such which take spatial into account (see below).
● Strangely the authors say “Computationally, only a small number of methods have been developed to analyze CCC specifically for spatial data”. However, this is simply incorrect, multiple methods exist which take spatial information into account, including SpatialDM (https://www.biorxiv.org/content/10.1101/2022.08.19.504616v1.full), stMLnet (https://www.biorxiv.org/content/10.1101/2022.06.27.497696v1.abstract, in which the corresponding author is a co-author), HoloNet (https://www.biorxiv.org/content/10.1101/2022.06.22.496105v2.full), SpaTalk (https://www.nature.com/articles/s41467-022-32111-8), DeepLinc (https://genomebiology.biomedcentral.com/articles/10.1186/s13059-022-02692-0), among others. The authors should compare their tool to the methods they perceive as most similar, and clarify the advantages of their method and the rest.
● The authors also mention that their method is “efficient”, though little is presented to back their claim in terms of being efficient relative to what, besides Supplementary Figure 37.
● Addressing these points would allow the authors to emphasise on the major advantages of their tool - i.e. that it works at the spot/cell level and takes spatial information into account.

● Applications of the method seem repetitive, and it's unclear why so many datasets are included if there are few changes in their analyses.
● In particular, unclear why Slide-seqV2 paragraph (Fig 5g-i) is included in the main text - it seems as a confirmatory result at best. Even more unclear for Mouse Visium in Fig 6. Unless clarified why these analyses are done, they should be moved as Supp Notes.

"Similar to CellPhoneDB and CellChat, COMMOT uses the minimum of the units of complexes by default with the choice of using the average"
● Then it still is unclear how does COMMOT deal with competition when working with complexes. Say a ligand (L1) binds both R1_R2 and R1_R3, where R1-3 are receptor subunits, would it compute the competition between the complexes? Or would it instead compute the competition between the minimum subunit? For example, if expression of R1 > R2 > R3, then is the competition for L1 computed using the values of R2 and R3, even if R1 is shared between the two complexes? This should be clarified.

● Besides the comparison to pairwise OT, we couldn't notice anything, in terms of unique CCC insights, to support the benefit of taking competition into account, excluding the presumed basic biological logic. The authors should address this, if this is a major selling point of their method.

● Unclear what is the benefit of original units/unnormalized distributions? Also, the authors log-normalise the data in their tutorial prior to running COMMOT, why?

● The authors should focus on the applicability of their tool by others. The 5 lines included as an example to the GitHub landing page are simply insufficient.
● Finally, the authors should make sure that the code works on any system, and any environment, not theirs alone. I still cannot install COMMOT, attempted now on 3 different Python versions, and two fresh conda environments, from both local path and pip…

Reviewer #2 (Remarks to the Author):

In this revision, the major points have been addressed and the authors revised the texts. The authors added sufficient benchmark analysis and supporting reports of the "validation".

One minor point:
Updated Figure 3 Panel e: In an effort to highlight all detected signals in the GAS6-TYRO3 FISH experiment, we labeled only GAS6 mRNAs that may have been difficult to see, leaving others unlabeled,

including those localized to the granular layer along with TYRO3. Please make sure the manipulation of the figure follows Nature Method Figure Policy.

Reviewer #3 (Remarks to the Author):

Re the COMMOT paper review (third round), the authors now addressed all of my concerns/comments sufficiently. I think this deserves to be published.

Author Rebuttal, second revision:

Response to Reviewer 1

Although, we agree that the proposed OT algorithm and its application to CCC are promising, to be published, we believe that the manuscript should undergo further revisions. The current manuscript portrays the method as iterative over previous developments, and masks its potential and usability.

In particular, instead of presenting the repetitive applications of their method, the authors should address the lack of comparison to other relevant tools and propose at least a basic working tutorial for their method.

Response: We thank the reviewer for the suggestions. In this revision, we have greatly improved the documentation of the package, including a) the addition of full tutorials and b) more comprehensive testing. We clarify in the following point-by-point response that we have compared COMMOT extensively with the methods that were available at the time when the manuscript was submitted. We have also followed the reviewer's suggestions to further clarify the unique features of COMMOT: a) its capability of handling competition between ligands or/and receptors and b) its functionality for dealing with spot and cell resolution in space.

Major points:

1. (As an answer to point 1.1) CellPhoneDB v3 simply restricts inference to microenvironments. So, to iterate the same point, unclear why the authors chose to compare their method to CellPhoneDB and CellChat, given that both work at the cluster level and neither takes spatial information into account. Furthemore, the reported performance gain is very underwhelming. Instead, the authors should demonstrate that their tool is superior to tools that work at the single-cell resolution (e.g. SCRIABIN https://www.biorxiv.org/content/10.1101/2022.02.04.479209v1.full) and/or such which take spatial into account (see below).

Response: We thank the reviewer for pointing out these tools. Those tools mentioned in points 1 and 2 only became available in public after our original submission of this manuscript. We therefore compared our method with a) CellChat – a popular CCC inference tool for scRNA-seq data, and b) two methods Giotto and CellPhoneDB v3 – spatial CCC inference methods that we could find before the original submission of this paper. Here, we summarize the first date of the methods becoming available: our method COMMOT: submitted on 10/08/2021; SCRIABIN: first on bioRxiv 02/07/2022; SpatialDM: first on bioRxiv 08/22/2022; stMLnet: first on bioRxiv 06/29/2022; HoloNet: first on bioRxiv 06/26/2022; SpaTalk: first on bioRxiv 04/13/2022; DeepLinc: not found on bioRxiv, published 06/03/2022.

Analyzing cell-cell communication from scRNA-seq data is a relatively new topic, and CCC analysis with spatial transcriptomics data is an even more recent topic as all these aforementioned methods were released or published in the past half year. It is difficult to quantify the performance comparison at this moment with those methods that have not been fully reviewed. We also found it difficult to use some of the aforementioned methods due to a lack of lucid documentation.

To highlight our method, in the paper we pointed out 1) it is one of the very first few CCC methods designed specifically for spatial transcriptomics data; 2) by the submission date of the paper, this is the first tool that models CCC in ST data at spot and cell resolution, enabling high-resolution downstream analyses; 3) it is the first method that considers competitions between multiple ligand and receptor species; and 4) a novel representation of signaling in space is provided by showing the directions of inferred spatial signaling.

2. Strangely the authors say "Computationally, only a small number of methods have been developed to analyze CCC specifically for spatial data". However, this is simply incorrect, multiple methods exist which take spatial information into account, including SpatialDM (https://www.biorxiv.org/content/10.1101/2022.08.19.504616v1.full), stMLnet (https://www.biorxiv.org/content/10.1101/2022.06.27.497696v1.abstract, in which the corresponding author is a co-author), HoloNet (https://www.biorxiv.org/content/10.1101/2022.06.22.496105v2.full), SpaTalk (https://www.nature.com/articles/s41467-022-32111-8), DeepLinc (https://genomebiology.biomedcentral.com/articles/10.1186/s13059-022-02692-0), among others. The authors should compare their tool to the methods they perceive as most similar, and clarify the advantages of their method and the rest.

Response: We thank the reviewer for the careful review. As discussed in the previous point, at the time of original submission of our paper, only a couple of tools were available in this area, and all the listed tools above only became available online as preprints or other forms after this paper was submitted (see the dates in reply to Comment 1). While we do not compare our method directly to these methods according to the journal policy as pointed out by the editor, we have added references to these methods to a new paragraph in Discussion:

"Most recently, several methods and packages have been introduced to study cell-cell communication with ST data. SpatialDM evaluates the co-expression of ligand receptor genes; SpaTalk and stMLnet are focused on signaling target genes; HoloNet studies the joint impact from different combinations of cell-cell communication events; DeepLinc constructs de novo cell-cell interaction landscapes without the need for annotated ligand and receptor genes. While COMMOT has a different focus, these methods arguably complement each other when studying different aspects of CCC."

3. The authors also mention that their method is "efficient", though little is presented to back their claim in terms of being efficient relative to what, besides Supplementary Figure 37. Addressing these points would allow the authors to emphasise on the major advantages of their tool - i.e. that it works at the spot/cell level and takes spatial information into account.

Response: In the last paragraph of Results section, we have described the efficiency in the following sentences:

"We found COMMOT running time scales linearly with the number of nonzero elements in the CCC (Supplementary Fig. 35). The number of CCC scales linearly with the number of locations in ST data due to the spatial range constraint, and the memory usage also scales linearly with the number of locations since only the finite values of the cost matrix and the nonzero values of the CCC matrix need to be stored."

In the current Supplementary Figure 35, we tested the running time for cases of various sizes, ranging from ~200000 to ~10000000 potential cell pairs for three major ST data types. We hope this is a good representation of computational efficiency on currently available ST data.

In this round of revisions, we have added new Supplementary Figures 31-32 to illustrate the advantages of the spot/cell level approach. Specifically, we found 1) some subcluster signaling hotspots that the cluster-level approach overlooks and 2) more accurate spatial signaling prediction to reduce the number of false positives in cluster-level approaches. The new Supplementary Figures 32 is shown below as an example.

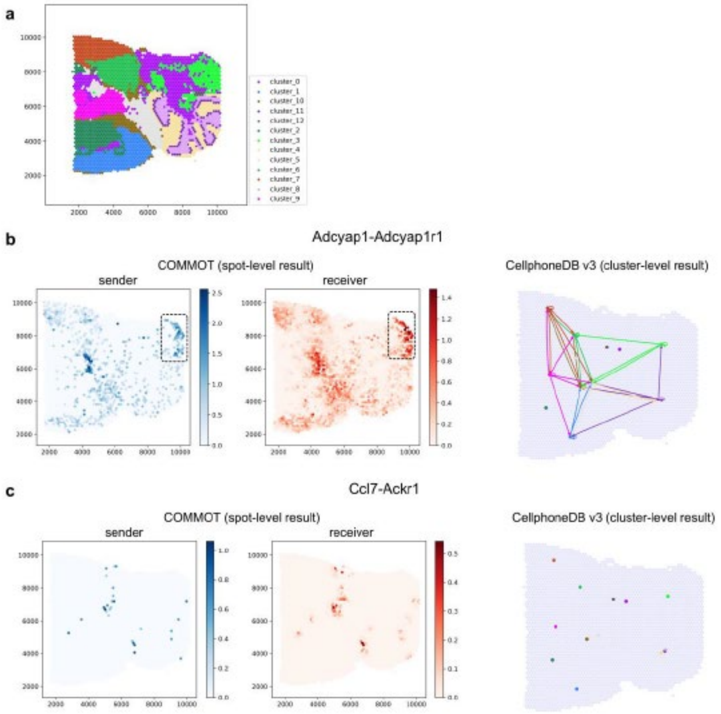

Supplementary Figure 32

Comparison of spot-level and cluster-level approaches on a Visium mouse brain dataset

*a Clustering of the spatial data. **b-c** Left two columns: The level of sent and received signals of the spots inferred by COMMOT. Right: The significant ($p \leq 0.05$) cluster-level signaling connections inferred by CellPhoneDB v3 with a cluster-level distance cutoff of 5000 µm. For the LR pair Adcyap1-Adcyapr1, COMMOT found a localized signaling hotspot within cluster 3 which is far away from other significant sender and receiver populations while the cluster-level method still identified significant signaling connections between cluster 3 and others. For the LR pair FGF7-FGFR1, COMMOT found local signaling hotspots which were overlooked by the cluster-level inference.*

4. Applications of the method seem repetitive, and it's unclear why so many datasets are included if there are few changes in their analyses.

In particular, unclear why Slide-seqV2 paragraph (Fig 5g-i) is included in the main text - it seems as a confirmatory result at best. Even more unclear for Mouse Visium in Fig 6. Unless clarified why these analyses are done, they should be moved as Supp Notes.

Response: We apologize for not being clear enough in our presentation. The different numerical experiments presented in the paper are meant to 1) emphasize the different utilities of COMMOT and 2) demonstrate its applicability to different types of data, which was also suggested by another reviewer. Specifically, Fig. 3 (previously Fig. 4) focuses on data of high spatial resolution and small gene number and the utility of spatial direction of signaling; Fig.4 (previously Fig. 5) focuses on data of high spatial resolution and a large gene number, which illustrates the utility of CCC-based clustering of data; Fig. 5 (previously Fig. 6) focuses on Visium data, which has a relatively low spatial resolution but great gene coverage, and the utility of identifying signaling DE genes. Indeed, both Slide-seq and Visium mouse brain examples are confirmatory results. However, since it is impossible to systematically and directly validate CCC inference results, these confirmatory results are valuable supportive information. Also, the Slide-seq data is an example of ST data containing a large number of spots, and it is useful in the main text to show COMMOT's capacity to handling data of such size.

5. "Similar to CellPhoneDB and CellChat, COMMOT uses the minimum of the units of complexes by default with the choice of using the average"

Then it still is unclear how does COMMOT deal with competition when working with complexes. Say a ligand (L1) binds both R1_R2 and R1_R3, where R1-3 are receptor subunits, would it compute the competition between the complexes? Or would it instead compute the competition between the minimum subunit? For example, if expression of R1 > R2 > R3, then is the competition for L1 computed using the values of R2 and R3, even if R1 is shared between the two complexes? This should be clarified.

Response: We apologize for the confusion. For the example of R1_R2 and R1_R3, COMMOT uses min(R1,R2) to represent the level of R1_R2 and min(R1,R3) to represent the level of R1_R3. Then, in the model, R1_R2 and R1_R3 are considered as two different receptor species that can both bind to L1. In the revision, we have clarified this in the Methods section "When the ligands or receptors contain heteromeric units, the minimum of units is used by default in the package to represent the amount of ligand or receptor. For example, if receptor species j is composed of two subunits, the minimum of them is used to represent the level of this receptor species X_(j,·)^R"

We agree that ideally, we would also consider the competition between R2 and R3 for the common subunit R1 within cells. We chose our current approach for the purpose of avoiding overlooking important CCC connections. We appreciate the reviewer's nice suggestion, and will consider modeling such detailed mechanisms within cells in the future by including more known molecular details.

6. Besides the comparison to pairwise OT, we couldn't notice anything, in terms of unique CCC insights, to support the benefit of taking competition into account, excluding the presumed basic biological logic. The authors should address this, if this is a major selling point of their method.

Response: Thank you for the good point. In this revision, we have added new Supplementary Figures 33 and 34 to demonstrate the impact of considering competitions between multiple ligands and receptors. Specifically, including the competition between ligand and receptor species in the model could help identify the most probable high-CCC-activity regions of a ligand-receptor. In the fourth paragraph of "Results – Robust identification of spatial signaling direction and downstream targets", we have added: "For a specific ligand-receptor pair, COMMOT further prioritizes regions containing its high signaling activity with low competitions from other pairs (Supplementary Figs. 33-34)." Here we include the new Supplementary Figure 34 as an example below.

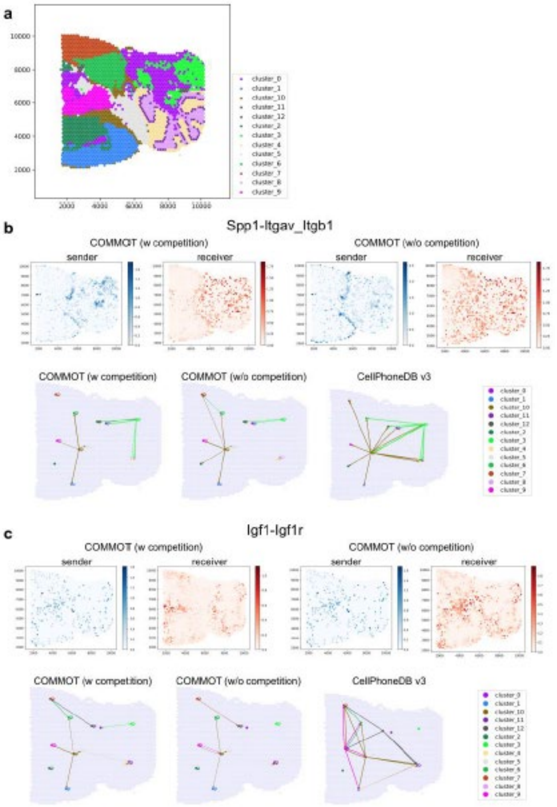

Supplementary Figure 34

Illustration of effect of competition on a Visium mouse brain dataset

a Clustering of the spatial data. ***b-c*** *Top: The level of sent and received signals of the spots inferred by COMMOT with or without considering competition between ligand and receptor species. Bottom: The significant ($p \leq 0.05$) cluster-level signaling connections inferred by COMMOT with or without considering competition and by CellPhoneDB v3 with a cluster-level distance cutoff of 5000 μm. For the LR pair Spp1-Itgav_Itgb1, COMMOT with considering competition found higher signaling activity on the right side of the tissue while the other approaches inferred active signaling everywhere. For the LR pair Igf1-Igf1r, COMMOT with considering competition also found localized signaling hotspots compared to other approaches.*

7. Unclear what is the benefit of original units/unnormalized distributions? Also, the authors log-normalise the data in their tutorial prior to running COMMOT, why?

Response: We are sorry for the confusion. The data is log-normalized to avoid outliers and preserve the variation. Here, "by unnormalized" we mean that the distributions do not need to be normalized as probability distributions, which is different from the normalizing total count approach. Here, we focus on maintaining comparability between different species. For example, when a gene is detected in just a few spots, normalizing its expression distribution into a probability distribution may result in overestimation in CCC inferences. This situation is illustrated in Supplementary Figure 5 using both a toy example and a real example.

To clarify this confusion, in the revision we have changed the description accordingly. Specifically, 1) In the first paragraph of "Results – Overview of COMMOT": We changed the original statement to "…taking non-probability mass distributions and controlling the marginals of the transport plan by these distributions to maintain the comparability between species"; and 2) In the fifth paragraph of "Introduction": we changed it to "preserving the comparability between distributions".

8. The authors should focus on the applicability of their tool by others. The 5 lines included as an example to the GitHub landing page are simply insufficient.

Response: We thank the reviewer for this suggestion. Now we have set up documentation pages on readthedocs.org: https://commot.readthedocs.io/en/latest/. A full detailed tutorial, API reference, and installation instructions are now provided on this site for more comprehensive documentation.

9. Finally, the authors should make sure that the code works on any system, and any environment, not theirs alone. I still cannot install COMMOT, attempted now on 3 different Python versions, and two fresh conda environments, from both local path and pip…

Response: Sorry for the inconvenience. COMMOT has several key dependencies, for example scanpy>=1.8 which requires Python 3.6 or later and pandas>=1.3 which requires Python 3.7 or later. It is therefore not practical to have the code work on any Python versions.

While we do not know the specific setups and issues the reviewer had when testing the package, in the revision we have made major effort to improve the installation process in addition to better documentation. In the revision, 1) we have changed to the up-to-date pyproject.toml + setup.cfg combination for installation configuration from the old setup.py approach; 2) we have relaxed the version requirements for some dependencies; 3) we have changed some dependencies (which require available R installation) as optional; and 4) we have implemented automatic tests of installation and core functions on Github for multiple python versions 3.7, 3.8, and 3.9 and in different operating systems MacOS and Ubuntu (see the screenshot below for the successful installation tests).

In addition, we asked five other colleagues who have different OS and computational environment setups to test installation from local path and from pip. Since the package was released online, we have heard from several external research groups who are interested in using our method: they have all successfully installed the package and used our package for their data analysis,

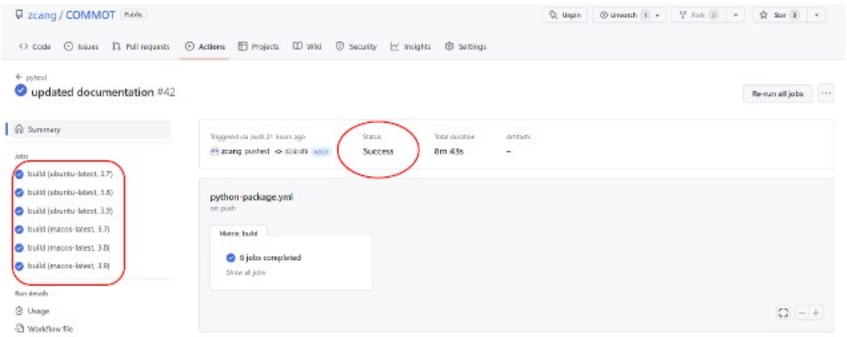

During the testing, we did notice that some additional efforts to install the dependencies are needed for Windows users. We have included details of these additional setups in the Installation documentation page (https://commot.readthedocs.io/en/latest/installation.html). If the reviewer still has issues with installation of the package, we would greatly appreciate it if the reviewer could provide more information about the installation failure so that we can further improve the installation process.

Response to Reviewer 2

In this revision, the major points have been addressed and the authors revised the texts. The authors added sufficient benchmark analysis and supporting reports of the "validation".

Response: We thank the reviewer for the valuable comments that have greatly improved the manuscript.

One minor point:

Updated Figure 3 Panel e: In an effort to highlight all detected signals in the GAS6-TYRO3 FISH experiment, we labeled only GAS6 mRNAs that may have been difficult to see, leaving others unlabeled, including those localized to the granular layer along with TYRO3. Please make sure the manipulation of the figure follows Nature Method Figure Policy.

Response: Thank you for bringing this up to our attention. We have included labels for all channels in the figure and, in the case of triple overlayed channels only, retained brightness increase for the GAS6 Channel (Gray) to improve clarity among the abundant TYRO3 signal (Green). The original brightness is retained in the middle panel. This complies with Nature Figure Policy.

Response to Reviewer 3

Re the COMMOT paper review (third round), the authors now addressed all of my concerns/comments sufficiently. I think this deserves to be published.

Response: We thank the reviewer for the valuable comments that have greatly improved the manuscript.

Final Decision Letter:

Dear Qing,

My apologies for the slight delay! I am pleased to inform you that your Article, "Screening cell-cell communication in spatial transcriptomics via collective optimal transport", has now been accepted for publication in Nature Methods. Your paper is tentatively scheduled for publication in our February print issue, and will be published online prior to that. The received and accepted dates will be 8 October 2021 and 21 November 2022. This note is intended to let you know what to expect from us over the next month or so, and to let you know where to address any further questions.

Acceptance is conditional on the data in the manuscript not being published elsewhere, or announced in the print or electronic media, until the embargo/publication date. These restrictions are not intended to deter you from presenting your data at academic meetings and conferences, but any enquiries from the media about papers not yet scheduled for publication should be referred to us.

Once your paper is typeset, you will receive an email with a link to choose the appropriate publishing options for your paper and our Author Services team will be in touch regarding any additional information that may be required.

Please note that <i>Nature Methods</i> is a Transformative Journal (TJ). Authors may publish their research with us through the traditional subscription access route or make their paper immediately open access through payment of an article-processing charge (APC). Authors will not be required to make a final decision about access to their article until it has been accepted. Find out more about Transformative Journals

Authors may need to take specific actions to achieve compliance with funder and institutional open access mandates. If your research is supported by a funder that requires immediate open access (e.g. according to Plan S principles) then you should select the gold OA route, and we will direct you to the compliant route where possible. For authors selecting the subscription publication route, the journal's standard licensing terms will need to be accepted, including self-archiving policies. Those licensing terms will supersede any other terms that the author or any third party may assert apply to any version of the manuscript.

You will not receive your proofs until the publishing agreement has been received through our system.

If you have any questions about our publishing options, costs, Open Access requirements, or our legal forms, please contact ASJournals@springernature.com

Your paper will now be copyedited to ensure that it conforms to Nature Methods style. Once proofs are generated, they will be sent to you electronically and you will be asked to send a corrected version within 24 hours. It is extremely important that you let us know now whether you will be difficult to contact over the next month. If this is the case, we ask that you send us the contact information (email, phone and fax) of someone who will be able to check the proofs and deal with any last-minute problems.

If, when you receive your proof, you cannot meet the deadline, please inform us at rjsproduction@springernature.com immediately.

Once your manuscript is typeset and you have completed the appropriate grant of rights, you will receive a link to your electronic proof via email with a request to make any corrections within 48 hours. If, when you receive your proof, you cannot meet this deadline, please inform us at rjsproduction@springernature.com immediately.

Once your paper has been scheduled for online publication, the Nature press office will be in touch to confirm the details.

If you have posted a preprint on any preprint server, please ensure that the preprint details are updated with a publication reference, including the DOI and a URL to the published version of the article on the journal website.

Content is published online weekly on Mondays and Thursdays, and the embargo is set at 16:00 London time (GMT)/11:00 am US Eastern time (EST) on the day of publication. If you need to know the exact publication date or when the news embargo will be lifted, please contact our press office after you have submitted your proof corrections. Now is the time to inform your Public Relations or Press Office about your paper, as they might be interested in promoting its publication. This will allow them time to prepare an accurate and satisfactory press release. Include your manuscript tracking number NMETH-A47293C and the name of the journal, which they will need when they contact our office.

About one week before your paper is published online, we shall be distributing a press release to news organizations worldwide, which may include details of your work. We are happy for your institution or funding agency to prepare its own press release, but it must mention the embargo date and Nature Methods. Our Press Office will contact you closer to the time of publication, but if you or your Press Office have any inquiries in the meantime, please contact press@nature.com.

To assist our authors in disseminating their research to the broader community, our SharedIt initiative provides you with a unique shareable link that will allow anyone (with or without a subscription) to read

the published article. Recipients of the link with a subscription will also be able to download and print the PDF.
As soon as your article is published, you will receive an automated email with your shareable link.

You can now use a single sign-on for all your accounts, view the status of all your manuscript submissions and reviews, access usage statistics for your published articles and download a record of your refereeing activity for the Nature journals.

Nature Portfolio journals encourage authors to share their step-by-step experimental protocols on a protocol sharing platform of their choice. Nature Portfolio 's Protocol Exchange is a free-to-use and open resource for protocols; protocols deposited in Protocol Exchange are citable and can be linked from the published article. More details can found at www.nature.com/protocolexchange/about.

Please note that you and any of your coauthors will be able to order reprints and single copies of the issue containing your article through Nature Portfolio 's reprint website, which is located at http://www.nature.com/reprints/author-reprints.html. If there are any questions about reprints please send an email to author-reprints@nature.com and someone will assist you.

Please feel free to contact me if you have questions about any of these points.

Best regards,
Allison

Allison Doerr, Ph.D.
Chief Editor
Nature Methods
